# First comprehensive catalogue of hibernating Darwin wasps in the Western Palaearctic (Hymenoptera, Ichneumonidae)

**DOI:** 10.3897/BDJ.13.e176441

**Published:** 2025-12-29

**Authors:** Fons Verheyde, Augustijn De Ketelaere, Geir Ørsnes, William Pénigot, Malcolm Storey, Ika Österblad, Al Cameron, Gunnar Engan, Miroslav Fiala, Jonas Lutz, Maksym Parkhomenko, Vladimir E Gokhman, Wouter Dekoninck, Stijn Cooleman, Jan Mees

**Affiliations:** 1 Flanders Marine Institute (VLIZ), Ostend, Belgium Flanders Marine Institute (VLIZ) Ostend Belgium; 2 Aculea (Natuurpunt), Mechelen, Belgium Aculea (Natuurpunt) Mechelen Belgium; 3 Scientific collaborator at Royal Belgian Institute of Natural Sciences, Brussels, Belgium Scientific collaborator at Royal Belgian Institute of Natural Sciences Brussels Belgium; 4 Unaffiliated, Bodø, Norway Unaffiliated Bodø Norway; 5 Unaffiliated, Occitanie, France Unaffiliated Occitanie France; 6 Unaffiliated, Wimborne, United Kingdom Unaffiliated Wimborne United Kingdom; 7 Unaffiliated, Jungsund, Finland Unaffiliated Jungsund Finland; 8 Unaffiliated, Wester Ross, United Kingdom Unaffiliated Wester Ross United Kingdom; 9 Unaffiliated, Oslo, Norway Unaffiliated Oslo Norway; 10 Unaffiliated, Přerov, Czech Republic Unaffiliated Přerov Czech Republic; 11 Unaffiliated, Borup, Denmark Unaffiliated Borup Denmark; 12 Unaffiliated, Kharkiv, Ukraine Unaffiliated Kharkiv Ukraine; 13 Russian Entomological Society, Moscow, Russia Russian Entomological Society Moscow Russia; 14 Royal Belgian Institute of Natural Sciences, Brussels, Belgium Royal Belgian Institute of Natural Sciences Brussels Belgium; 15 Belgian Biodiversity Platform, Brussels, Belgium Belgian Biodiversity Platform Brussels Belgium

**Keywords:** Ichneumonid wasps, diapause, overwintering, ecology, faunistics, citizen-science data, Europe

## Abstract

**Background:**

In the Western Palaearctic, many species of Darwin wasps exhibit a form of diapause known as free-living adult diapause, similar to hibernation in certain beetle, bumblebee and butterfly species. This study provides a first comprehensive overview of all known hibernating species and aims to improve the current ecological knowledge.

**New information:**

We reviewed 439 species, confirming free-living adult diapause in 340; 81 remain unverified and 18 are excluded, which have been incorrectly reported as hibernators in the past. The validated dataset includes 7443 records from 27567 specimens, spanning over 235 years of both published and unpublished observations. We report 29 species as hibernators for the first time. Amongst the records, 388 provide the first evidence of hibernation for a species in a given country, with 67 also representing the species' first national record. We highlight the value of field-based data and caution against relying solely on collection dates to study diapause. The observed variability in diapause strategies and hibernacula underscores the importance of nature management for biodiversity conservation, especially preservation of microhabitats.

## Introduction

The concept of diapause is used to designate a dynamic process of several successive phases of low metabolic activity, ofted used by organisms to bridge unfavourable environmental conditions (Denlinger 2022). The best known examples of adult diapause in insects include vespid wasps, bumbleblees, some beetles and butterflies. In this paper, we discuss free-living adult diapause in the Western Palaearctic (defined as 'Greater Western Palaearctic'; Flanders Marine Institute (2025)) for the Hymenoptera family of Darwin wasps or Ichneumonidae.

In this catalogue, we describe the hibernation preferences for 439 species occurring in 37 countries in the Western Palaearctic. A total number of 7443 records and 27567 female specimens were integrated, originating from unpublished field data collected by the authors, public and private collections, citizen-science data and all published literature from 1790 and the subsequent 235 years. Analysing the ecology and behaviour of these species, which may even differ at population level (Verheyde and Quicke 2022; Discussion), it is shown how a case-by-case evaluation is necessary for proper judgement of the species' general biology. This results in 340 confirmed species using free-living adult diapause.

Unlike various other insects - such as some certain butterflies and true bugs (Denlinger 2022) - only female Darwin wasps hibernate. Their life cycle typically proceeds as follows. An (often univoltine) species mates during the year and feeds on different kinds of flowers or honeydew of sap-feeding insects to build up fat reserves. From autumn onwards, the female starts searching for a hibernation spot (*hibernaculum*) during the prediapause phase. In the subsequent weeks, the deepest phase of diapause is triggered and the individual remains largely immobilised. At the postdiapause phase, the metabolism is activated again and shortly after, the diapause ends. Egg maturation is now resumed (generally, hibernators are thus synovigenic) and shortly after, fertilised eggs can be deposited on or in the host (Hinz 1983). Usually the diapause lasts until March or May, depending on latitude and altitude. Hibernation typically can be extended in colder or snowy regions, for example in Scandinavia or mountain ranges in Central Europe. The mechanism of diapause also occurs during summer, which is called aestivation (Tougeron 2019).

Several types of diapause occur amongst parasitoid wasps. Diapause may take place during the larval or pupal stages (Quicke 2015). In some species, adults enter hibernation at the developmental site or within the host cocoon. In such cases, the individual (a pharate adult) has not been able to mate or fly freely yet. This overwintering strategy is common amongst various species of Banchinae (e.g. *Lissonota*), Cryptinae and Phygadeuontinae (e.g. *Bathythrix*) (Schwarz and Shaw 1999, Schwarz and Shaw 2000, Schwarz and Shaw 2010, Shaw 2017). Beyond these developmental stages, diapause can also vary in its dependence on physiological and environmental cues. Some species undergo facultative diapause, entering dormancy only under certain conditions, while others experience obligate diapause. In obligate diapause, the development is arrested regardless of any environmental conditions (Hahn and Denlinger 2007).

Generally, the key factor initiating diapause in Western Palaearctic insects is photoperiodicity or the length of daylight (Tougeron 2019, Denlinger 2022). Temperature comes second, although it possibly plays a more important role in summer diapause. Other factors can be humidity, food availability, the life phase of the host or even competition between species or populations (Denlinger 2022, Shen et al. 2025).

## Materials and methods

### Data collection

**Figure 1.**
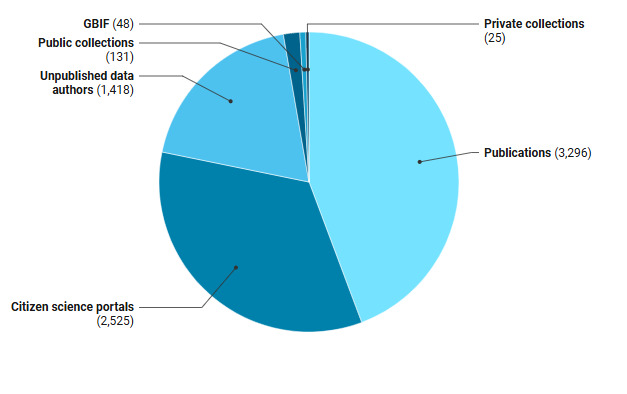
Origin of the data used in the dataset and catalogue.

To compile our dataset, integrated in the catalogue, a variety of sources was used (Fig. 1). A first set (19%) consists of unpublished records from the authors in this paper. A second set (3%) are unpublished records from both public and private collections. A third set (34%) are records from citizen-science portals. The final set (44%) contains all published records. We included external data before 30/04/2024 and authors' data before 30/04/2025. However, exceptions were made, especially in the case of new country records. A record is defined as an observation of one or more individuals at a fixed moment of time.

Records from the authors were compiled from excursions and inventories in their countries of origin. Such took place mainly in Belgium (Fig. 2) and to a much lesser extent the Netherlands (2018-2025), Czechia (2010-2011 and 2024), France (2004-2022), Norway (2010-2021), Russia (1983-2013), the United Kingdom (2018-2025) and Ukraine (2020-2025).

In our second set, an exhaustive approach was not made. However, the collection of Royal Belgian Institute of Natural Sciences (RBINS; Brussels) was completely checked and included and, where possible, additional museum catalogues were looked for example via online resources such as GBIF. It is important to emphasise records were only included when there was an explicit statement of hibernation on the specimen labels (Fig. 3). Exceptionally, records were also included ad hoc when there was contextual evidence of the species being found in hibernaculum (i.e. an entomologist who has been known for searching hibernating beetles under bark). We did not include specimens on the sole base of date, for example, as explicitly mentioned in Sebald et al. (2000), as this is not a reliable criterion for hibernation (Discussion). Finally, some specimens from private collections were integrated.

Our third set is rooted in the recent emergence of citizen science. It is our second largest dataset, based on the observations of many hobbyists throughout Europe (Table 1). Only records with photographic evidence and a clear indication of hibernation were included in the dataset. Every record was carefully checked by at least one of the authors to confirm the identification. We decided to use species complexes when identification to species level was not possible. They were not used in the catalogue; however, some are discussed in the species’ notes (Catalogue), especially when it would have yielded new country records.

**Figure 4.**
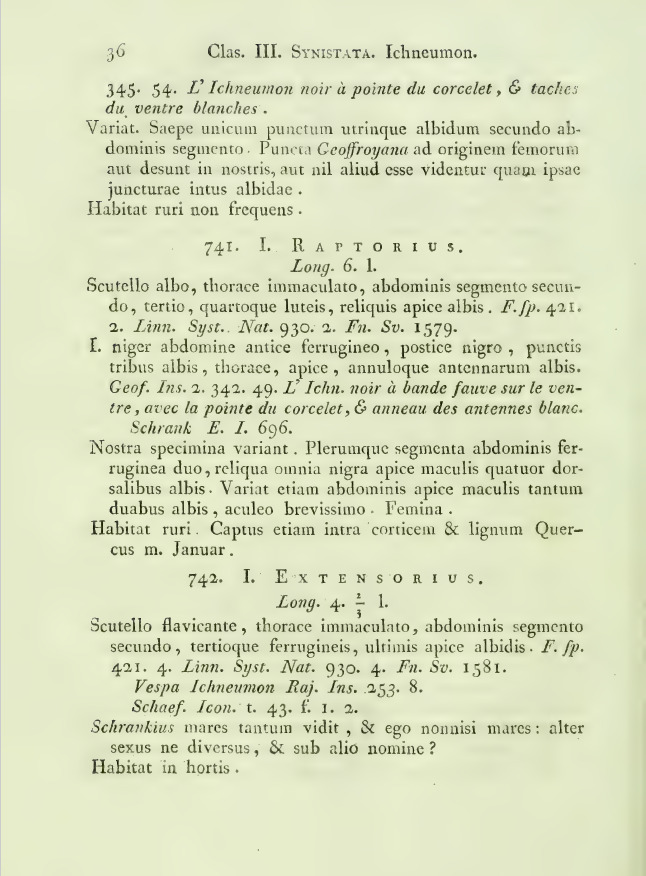
Extract of Rossius (1790), with the first description of a hibernating ichneumonid wasp, *Diphyus raptorius*.

For the fourth set, all existing literature known to us was checked, going back to the first report by Rossius (1790) (Fig. 4). Although we made a lot of effort in finding literature, it is possible we missed smaller remarks on species in local faunistic journals. During our research, several issues arose when consulting older literature. Older nomenclature and shifting country borders (e.g. Germany and Poland, Hungary and Romania) were a first issue encountered. Furthermore, it was not always clear if the data were original, especially when cross-references were missing. A fair share of examples are found in the works of Tischbein (Tischbein (1863), Tischbein (1871), Tischbein (1873a), Tischbein (1873b), Tischbein (1874a), Tischbein (1874b), Tischbein (1876), Tischbein (1879), Tischbein (1881)), Berthoumieu (Berthoumieu (1894), Berthoumieu (1895a), Berthoumieu (1895b), Berthoumieu (1896)) and Heinrich (Heinrich (1926), Heinrich (1927), Heinrich (1928), Heinrich (1930), Heinrich (1935), Heinrich (1936), Heinrich (1949a), Heinrich (1949b), Heinrich (1951)), where findings were often recited. On another occasion, the context of a finding was unclear; when and where exactly was the specimen found? How did the authors decide to report a given species as hibernating? Was this decided based on the date, additional labels, notes or rather information via personal networks? This is especially worrisome in some previous catalogues (e.g. Berthoumieu (1894), Rasnitsyn (1964), Sebald et al. (2000)). Similar to all other literature, we checked the original source case-by-case and added our assessment for dubious records in the species’ Notes. A final issue is that some researchers reported in bulk, meaning they reported a total number of occurrences per species, collected at different occasions. In this case, it was included as only one record in our dataset, while these records probably constitute different occurences in reality. This is important to bear in mind when studying species aggregates, population density etc. We tried to split and break down the bulk reports to the level of the occurrence, whenever possible. For example, while some of the data of Tereshkin are published in several papers (e.g. Tereshkin (2002), Tereshkin (2003), Tereshkin (2004), Tereshkin (2005)), we generally used his unpublished datasets (Tereshkin 2014) as they were more complete and as the records were available to the level of occurrences here.

All literature was uploaded to the platform TaxonWorks and is available upon request. The correlation graph shown in Fig. 9 was generated using *R* (R Core Team 2024) in *RStudio* (Posit Team 2025). Visualisation of the results was performed using the ggplot2 package (version 3.5.2; Wickham (2016)).

### Catalogue structure and terminology

We structured our results consistently; when certain fields are missing (e.g. hibernaculum), it means they are not available based on our source material.

In '**Status**', three classifications are used: Confirmed, Unverified and Incorrect. For ‘Confirmed’, we have valid records of specimens found in hibernacula and/or very strong indications the species is likely to hibernate (e.g. obligatory diapause at the genus level or author reliability). When the hibernaculum or the identification was unclear, we used the status 'Unverified'. To confirm these, more data would be needed. Lastly, for ‘Incorrect’, there are strong indications free-living adult hibernation is unlikely. Three species have an unclear status taxonomically and received a fourth status 'Taxon requiring further verification'. We used '*' when a species is reported for the first time as a hibernator.

In '**Records**', the number of country records is specified between brackets. The symbol '*' was used at the level of the countries to highlight that this finding is reported as a hibernator for the first time in that given country. A double symbol '**' was used to state this species had not previously been reported in that country. We used Yu et al. (2016) as a baseline to check this, but also consulted more recent literature and several local experts.

'**Sources**' include all publications or unpublished data which was integrated directly into the dataset. As for personal observations by the authors of this paper, the abbreviation ‘Pers. obs.’ was used. Unpublished external personal observations were always specified between brackets. ‘**Also mentioned in**’ is mostly used for self- and recitations, often without obvious original record (e.g. Morley (1903), Constantineanu (1959)).

For the '**Hibernacula**' types, we used the typology as set out in Verheyde and Quicke (2022) (Table 2). Between brackets, we mention the absolute number of individuals reported at a specific hibernaculum.

More detailed information on all records and sources (dates, hibernacula, collector, identification etc.) can be found in our dataset (Verheyde et al. 2025b).

## Data resources

Two datasets were prepared to register into the Global Biodiversity Information Facility (GBIF). The first, Verheyde et al. (2025b), contains original data, mostly records collected by the authors, but also all other data previously unpublished on GBIF. The second dataset compiles all derivative data with references to their GBIF data publishers.

The new dataset was published as a standardised Darwin Core Archive. For each species listed in the Taxon Core, it provides available information on overwintering stage (in the Taxon Description Extension), hibernaculum type, country code, references, source and other relevant terms (in the Occurrence or Species Distribution Extension). To comply with privacy regulations, all observers other than the authors were anonymised. Obtaining permission to publish certain citizen-science records individually proved challenging at times, even when the authors had contributed substantially by identifying species or processing data. This difficulty was particularly pronounced when GBIF imports occurred only sporadically or involved a limited subset of records. Consequently, some entries were grouped or “clustered”, resulting in a reduced level of detail that could be shared.

## Checklists

### Catalogue of overwintering Ichneumonid wasps

#### Ichneumonidae

F2CE9DC3-DBB1-5B86-AC55-1AE456BD62C9

#### Banchinae

5397FEF0-1DEC-5E1A-8E36-1CFE9278EEFC

##### Notes

The subfamily of Banchinae consists of koinobiont endoparasitoids of Lepidoptera larvae (Broad et al. 2018). No certain records of adult free-living diapause exist; in the genus *Lissonota*, for example, there is evidence that hibernation occurs as pharate adult (Shaw 2017, Johansson 2024).

#### Lissonota digestor

(Thunberg, 1824)

7A844B9D-82FB-5629-917D-A5C715D6AB56

##### Notes

See our statements for the subfamily of Banchinae.

**Status**: Incorrect.

**Records**: Romania, 30.VI.1960.

**Hibernacula**: C (1).

**Sources**: Decou and Decou (1961) [*Echthrodoca hians* Thoms.].

#### Campopleginae

530C897D-9D22-5C26-AEEF-79743B16403F

##### Notes

Campopleginae are koinobiont endoparasitoids, mainly specialised on Lepidoptera, sometimes other orders too, like Coleoptera (see below). The vast majority overwinters as a young larva in an overwintering host larva or in the cocoon stage, either as a prepupa or, in some cases, a pharate or enclosed adult. However, one species has been reported as hibernating in Switzerland and there may be more species as several are known to emerge late in the year with no evident next host available (Broad et al. 2018).

#### Enytus montanus

(Ashmead, 1890)

F1BC5236-B5A7-5E42-90C6-D9768A1955A3

##### Notes

Holarctic species. It is reported as hibernating in North America in the forest soil (Baltensweiler 1958).

**Status**: Confirmed.

**Records**: Switzerland, 1954-1957.

**Hibernacula**: DV (1).

**Sources**: Baltensweiler (1958) [*Horogenes exareolatus* Ratz.].

#### Bathyplectes infernalis

(Gravenhorst, 1820)

36470DB6-E11D-59FF-9565-57926838C609

##### Notes

It is not entirely clear from the paper what the authors mean by ‘adults that emerge in the fall are capable of surviving the winter, more specifically in cracks and crevices in the soil and secluded places at the bases of the host plant’. This species is Holarctic and shows high degrees of synchronicity with its plurivoltine host, the beetle *Brachypera zoilus* (J. A. Scopoli, 1763). There are some records in the United States, early in the year, but it is unclear if these specimens were hibernating. Any data from the Western Palaearctic region are missing.

**Status**: Unverified.

**Sources**: Pottler and Coles (1962) [*Biolysia tristis* Grav.].

#### Hyposoter ebeninus

(Gravenhorst, 1829)

B5122007-6EBB-5ABD-A6ED-80DDC53D086A

##### Notes

Assumed to hibernate in Busch (1951). However, this is nothing, but an assumption, which seems unlikely knowing the ecology of species in the subfamily. More specifically, if diapause occurs, it will be as a pharate adult in the host cocoon (Shaw et al. 2016).

**Status**: Incorrect.

**Sources**: Busch (1951) [*Anilasta vulgaris* Tschek].

#### Cryptinae

1D272835-D7B8-5D8F-9CC6-C6EC790C1B62

##### Notes

This subfamily, similar to Phygadeuontinae, has a large diversity of idiobiont ectoparasitoids of more or less concealed hosts, which consist of a wide variety of host orders (Broad et al. 2018). Multiple hibernation strategies are used as demonstrated in the species below.

#### Aptesini

77F30935-1AFD-555C-B72A-3B59B51F8C8F

#### Aptesis nigrocincta

(Gravenhorst, 1815)

DD8F0540-2E65-5557-941F-B424668B57AC

##### Notes

*Aptesis nigrocincta* is specialised on sawfly hosts, for example, the apple sawfly *Hoplocampa testudinea* (Klug, 1816). This host association is more exceptional within hibernating Ichneumonidae, but it is possible that the diapause is facultative here. However, the species has been found in a wide variety of hibernacula and countries, with a slight preference for hibernation in vegetation, notably grass tussocks.

**Status**: Confirmed.

**Records**: Belgium (2), Denmark (1)*, France (1), Ireland (4), Norway (4), Russia (1), Sweden (1)*, Switzerland (1)*, The Netherlands (2)*, United Kingdom (13).

**First record**: 13.III.1904 (Morley (1907), United Kingdom).

**Hibernacula**: DT (4), DV (5), LV (15), M (5), S (3), U (1).

**Sources**: Morley (1907) [*Microcryptus nigrocinctus* Grav.], Anonymous (1909) [*M. nigrocinctus* Gr.], Johnson (1920) [*M. nigrocinctus* Gr.], Seyrig (1923) [*Aptesis nigrocinctus* Gr.], Hancock (1923) and Hancock (1925) [*M. nigrocinctus* Grav.], Rasnitsyn (1964) [*M. nigrocinctus* Grav.]; Unpublished: Artportalen, Naturbasen, Observation.org, Pers. obs.

#### Cubocephalus sperator

(Muller, 1776)

6C105CEE-5911-579A-BD6D-F931EA6BDB1E

##### Notes

Currently, no other species in this genus are known to hibernate and, as most species are associated with Coleoptera, hibernation is rather unlikely (Broad et al. 2018).

**Status**: Unverified.

**Records**: Belgium, 19.X.1906 (Anonymous 1909, Belgium).

**Hibernacula**: M (1).

**Sources**: Anonymous 1909 [*Microcryptus sperator* Gr.].

#### Oresbius galactinus

(Gravenhorst, 1829)

BE9B5FC1-BB4D-597A-A5F1-5EA5B4C93D1D

##### Notes

This genus needs to be revised taxonomically and no recent records are known. Moreover, Johnson (1920) reports this species in November, from moss, which could also be coincidental.

**Status**: Unverified.

**Records**: Ireland, 1890-1920.

**Hibernacula**: M (1).

**Sources**: Johnson (1920) [*Microcryptus galactinus* Gr.].

#### Parmortha parvula

(Gravenhorst, 1829)

C1BDFED8-A9CD-5A1E-9494-E3C559CB73C8

##### Notes

Hibernation is unclear. Both specimens were found in October near the soil, possibly still searching for host pupae (Symphyta).

**Status**: Unverified.

**Records**: Belgium (1), Norway (1).

**First record**: 19.X.1906 (Anonymous 1909, Belgium).

**Hibernacula**: DV (1), M (1).

**Sources**: Anonymous 1909 [*Cratocryptus parvulus* Gr.]; Unpublished: Pers. obs.

#### Parmortha pleuralis

(Thomson, 1873)

3BDF770F-D587-5BBA-B838-1732E41CDE37

##### Notes

Hibernation is unclear, see the species above.

**Status**: Unverified.

**Records**: Belgium, 15.X.1906.

**Hibernacula**: M (1).

**Sources**: Anonymous 1909 [*Cratocryptus pleuralis* Thoms.].

#### Rhembobius bifrons

(Gmelin, 1790)

97565FF6-0F7C-52F9-8DC5-EC2EAE6CB522

##### Notes

The first specimen was found in moss, in woods. Other specimens were found more recently in Belgium, by sieving and in Scotland, more specifically in an ant nest below a stone.

**Status**: Confirmed.

**Records**: Belgium (1)**, Ireland (1), United Kingdom (2)*.

**First record**: 1890-1920 (Johnson (1920), Ireland).

**Hibernacula**: DV (1), M (1), S (2).

**Sources**: Johnson (1920) [*Microcryptus bifrons*]; Unpublished: Coll. RBINS, Pers. obs.

#### Rhembobius perscrutator

(Thunberg, 1824)

E0B8F87B-6903-56BA-843F-D32E46FCB1D9

##### Notes

A more common *Rhembobius* species, which also appears to hibernate in vegetation, notably grass tussocks. Bauer (1984) list this specimen, exceptionally, as found ‘in Stubben’ (in tree stumps).

**Status**: Confirmed.

**Records**: France (2), Germany (1), United Kingdom (3)*.

**First record**: 1915-1922 (Seyrig (1923), France).

**Hibernacula**: DT (1), LV (9).

**Sources**: Seyrig (1923) [*Acanthocryptus nigritus* Gr.], Bauer (1984), Pénigot (2020); Unpublished: Pers. obs.

**Also mentioned in**: Rasnitsyn (1964) [*A. perscrutator* Thunb.], Schwarz and Shaw (2010).

#### Rhembobius quadrispinus

(Gravenhorst, 1829)

703495D3-77D2-5C39-95F0-F914E270838D

##### Notes

Most common in grass tussocks, but it also listed by Bauer (1984) as ‘in Stubben’ (tree stumps) and, more recently, a specimen was discovered in moss. Several uncertain records exist on citizen-science portals.

**Status**: Confirmed.

**Records**: Belgium (1), Denmark (1)*, France (1), Germany (1), Russia (2), United Kingdom (14).

**First record**: ca. 1904 (Morley (1907), United Kingdom).

**Hibernacula**: DT (1), LV (18), M (2), U (2).

**Sources**: Morley (1907) [*Acanthocryptus quadrispinus* Grav.], Anonymous 1909 [*A. quadrispinus* Grav.], Hancock (1923) [*A. quadrispinus* Grav.], Seyrig (1923) [*A. quadrispinus* Gr.], Rasnitsyn (1964) [*A. quadrispinus* Grav.], Bauer (1984); Unpublished: Naturbasen, Pers. obs.

**Also mentioned in**: Schwarz and Shaw (2010).

#### Cryptini

D4213C9D-63EC-5B97-8B45-BF80299F3E68

##### Notes

Several species have erroneously been reported to hibernate. Our findings provide additional evidence, however, on how *Ischnus* spp. do in fact hibernate, adding (evergreen) trees as an important hibernaculum, next to the known records from Cole (1979) in vegetation or dead litter.

#### Agrothereutes abbreviatus

(Fabricius, 1794)

5051EE12-67B0-5E09-B92A-9EA3EE7AED7D

##### Notes

One older record from Belgium below moss and one record from the Netherlands, where a female specimen was observed below the bark of a dead tree. There are no other indications from the ecology and phenology of the species (or genus) that hibernation could be likely. The individuals were probably searching for hosts or in a state of quiescence.

**Status**: Unverified.

**Records**: Belgium (1), the Netherlands (1).

**Hibernacula**: DT (1), M (1).

**Sources**: Anonymous (1909) [*Spilocryptus pygoleucus* Gr.], Verheyde and Quicke (2022).

#### Enclisis macilenta

(Gravenhorst, 1829)

5859FBB3-8281-5807-85C6-26FED4847A01

##### Notes

It is unclear under which circumstances this species was collected. However, this species hibernates as a pharate adult in the (aculeate) host nest (Schwarz and Shaw 1998) and was omitted in Rasnitsyn (1964).

**Status**: Incorrect.

**Records**: Russia, 1951-1958.

**Sources**: Rasnitsyn (1959) [*Coenocryptus laticrus* Thoms.].

#### Enclisis vindex

(Tschek, 1871)

47995CFF-94E8-54C6-8B5D-775E28083C07

##### Notes

See our statements on *E. macilenta*.

**Status**: Incorrect.

**Records**: Russia, 1951-1958 (2 records).

**Sources**: Rasnitsyn (1959) [*Coenocryptus striolatus* Thoms.].

**Also mentioned in**: Rasnitsyn (1964) [*C. striolatus* Thoms.].

#### Ischnus agitator

(Olivier, 1792)

FCFB95A5-5551-5814-8574-52C239BAFB48

##### Notes

One specimen was discovered by beating a fallen tree branch (*Pinus sylvestris*) at one metre height, the branch containing numerous dry needles.

**Status**: Confirmed*.

**Records**: The Netherlands, 25.XI.2023.

**Hibernacula**: LT (1).

**Sources**: Observation.org.

#### Ischnus alternator

(Gravenhorst, 1829)

25F99A3C-D999-5629-9618-E1FDA5E00BA9

##### Notes

One specimen was discovered hibernating deep in a rotting spruce stump.

**Status**: Confirmed*.

**Records**: Czechia, 26.X.2025.

**Hibernacula**: DV (1).

**Sources**: Unpublished: Pers. obs.

#### Ischnus inquisitorius

(Muller, 1776)

01E76CEF-2FE1-5486-A939-1A7ECA5D9586

##### Notes

Several specimens were collected on fallen oak branchs, after a storm passed the Netherlands in the winter of 2023-2024. Two specimens were collected on ivy. One uncertain record for France (iNaturalist) was not included.

**Status**: Confirmed.

**Records**: The Netherlands (5)*, United Kingdom (9).

**First record**: IV.1971 (, United Kingdom).

**Hibernacula**: DV (6), LT (8), LV (3).

**Sources**: Cole (1979); Unpublished: iNaturalist, Observation.org, Pers. obs.

**Also mentioned in**: Schwarz and Shaw (1998) [*Ischnus migrator* (Fabricius)].

#### Ctenopelmatinae

63D205FC-57EB-53A7-B5D4-5574FA2309A6

##### Notes

Koinobiont endoparasitoids of sawfly larvae. No certain records exist of adult free-living diapause, most adults probably hibernate in the host cocoon, sometimes as an adult (Broad et al. 2018).

#### Alexeter multicolor

(Gravenhorst, 1829)

9FD4970C-ED7E-56DA-B17F-74D7AF6353F6

##### Notes

Another odd record with little context. Although Bauer (1984) reports it as occasionally overwintering, the species is excluded from his more extensive list of species and hibernacula.

**Status**: Incorrect.

**Records**: Germany, ca. 1984.

**Sources**: Bauer (1984) [*Mesoleius multicolor*].

#### Ichneumoninae

1DAA5CB3-C240-5D67-B04F-1951E31FF8B6

##### Notes

The main part of adult hibernators consists of Ichneumoninae. We follow the classification from Santos et al. (2021). All species are endoparasitoids of Lepidoptera (Broad et al. 2018).

#### Ichneumonini

444F101C-5F99-504B-8617-DA6356ACCB84

##### Notes

Diapause seems obligate in several genera of Ichneumonini, notably *Aoplus*, *Chasmias*, *Diphyus*, *Exephanes* (specialised in caves), *Hoplismenus*, *Stenichneumon*, *Syspasis* and, of course, *Ichneumon* spp. Notwithstanding reports in older literature, it is clear that species of *Coelichneumon* do not hibernate as adults. Some species of *Cratichneumon* and *Virgichneumon* have been found hibernating with some certainty and some species of *Vulgichneumon* and *Ctenichneumon* with less certainty. Some others, often less species-rich genera, share this mixed situation (e.g. *Eutanyacra*, *Limerodops* and *Spilichneumon*). In these cases, more data are necessary to make statements on the character of the hibernation.

#### Achaius margineguttatus

(Gravenhorst, 1829)

7EB392A5-0671-5F13-9934-61C3BE1E6273

##### Notes

Several specimens were found in moss in Belgium (Athimus 1901b).

**Status**: Confirmed.

**Records**: Belgium, ca. 1900 (Athimus 1901b).

**Hibernacula**: M (1).

**Sources**: Athimus (1901b) [*Amblyteles margineguttatus* Gr.].

#### Achaius oratorius

(Fabricius, 1793)

81239F6B-2C8F-5367-B883-2F81096820D5

##### Notes

Remarkably, this species has over 1500 records on GBIF, but records of hibernating specimens are extremely rare. The species is included in Berthoumieu (1895b) as a hibernator, but this was only confirmed in 2014. This may demonstrate diapause is rather facultative and/or the hibernaculum is limited to tussocks or very specialised. In the Netherlands, one specimen was observed feeding on bait (used for moths) on 16.II.2023 (Observation.org). Additionally, in Austria, a specimen was collected on 15.XII.1927 (GBIF).

**Status**: Confirmed.

**Records**: Belgium (1)*, France (2), United Kingdom (1)*.

**First record**: Ca. 1895 (Berthoumieu (1895b), France).

**Hibernacula**: DT (2), LV (1), M (1).

**Sources**: Berthoumieu (1895b) [*Amblyteles oratorius* Fabr.]; Unpublished: Coll. RBINS, Pers. obs.

**Also mentioned in**: Morley (1903) [*A. oratorius* Fab.], Constantineanu (1959) [*A. oratorius* Fab.].

#### Amblyteles armatorius

(Forster, 1771)

ACD5C711-1FB4-561F-9261-634EEFBF4815

##### Notes

Together with *Diphyus quadripunctorius*, this is one of the few species known for summer diapause. Females form aggregations, often using buildings for that purpose. In Belgium, for example, up to 60 specimens were found in the crypt of St. Hubert on 26.IX.2023. In comparison to *D. quadripunctorius*, the usage of these alternative structures is higher, which may simply be due to the species being more common in an urban environment. There are also observations in natural caves, however, for example, in the limestone caves in the Netherlands (Vergoossen et al. In press), but the density of specimens tends to be lower here. An exception is the description of Seyrig (1926b), who mentions more than 500 individuals in a certain fissure on a height of 1,300 metres in the south of Spain. More rarely it has also been observed in litter or grass tussocks (Seyrig 1923 & Pers. obs.). The specific diapause rhythm for this species seems to be much more complex (and variable) compared to other species since active specimens have been observed at any time.

**Status**: Confirmed.

**Records**: Austria (1)*, Belgium (3), Bulgaria (1), Denmark (2)**, France (2), Germany (7), Grand Duchy of Luxembourg (2), Hungary (1), Italy (3)*, Poland (1)*, Romania (2), Slovenia (3), Spain (10), Switzerland (6), the Netherlands (16), Ukraine (1)*, United Kingdom (3)*.

**First record**: Ca. 1914 (Szilády (1914), Hungary).

**Hibernacula**: B (87), C (677), DV (1), LV (3), U (3).

**Sources**: Szilády (1914) [*Amblyteles fasciatorius* F.], Seyrig (1923) [*A. fasciatorius* L.], Seyrig (1926b), Heinrich (1936), Mohr (1938), Leruth (1939), Collart (1941), Aellen and Strinati (1962), Dobat (1975), Weber (1989), Selfa and Escolà (1991), Novak (2005), Schmidt and Zmudzinski (2005), Ortiz-Sánchez and Fernández (2008), Novak et al. (2010), Sebald and Weber (2013), Fernández et al. (2013) and Fernández and Ruiz (2014), Verheyde and Quicke (2022), Vergoossen et al. (In press); Unpublished: iNaturalist, iRecord, Naturbasen, Naturgucker, Observation.org, Pers. obs., Pers. obs. (G. Artmann).

**Also mentioned in**: Leruth (1939), Strinati (1966), Weber (2014).

#### Anisopygus pseudonymus

(Wesmael, 1845)

D0D82D0F-C251-5EA9-AFF8-2168EC863B83

##### Notes

This species was only recently discovered to be a hibernator in 2012 in northern France. It appears to be rare in Europe, mostly confined to Scandinavia nowadays (GBIF).

**Status**: Confirmed.

**Records**: France, 15.I.2012.

**Hibernacula**: DT (1).

**Sources**: Valemberg and Vago (2013).

#### Aoplus altercator

(Wesmael, 1855)

43EA291E-D682-5A93-81C3-27ABB6CD3582

##### Notes

None.

**Status**: Confirmed.

**Records**: France (2), Germany (1), Russia (1).

**First record**: 1924 (Seyrig (1926b), France).

**Hibernacula**: DT (7), M (1), U (2).

**Sources**: Seyrig (1926b) [*Cratichneumon altercator* Wsm.], Heinrich (1949b), Valemberg (1982), Riedel and Humala (2009).

#### Aoplus castaneus

(Gravenhorst, 1820)

B61D1D61-0EA6-50D6-85FD-7866A9C0ECE0

##### Notes

One uncertain hibernating record exists from Sweden (GBIF), on 3.II.1918. Most specimens were encountered by Rasnitsyn (1959) in Russia.

**Status**: Confirmed.

**Records**: Austria (1), Belarus (1), France (2), Germany (7), Russia (1).

**First record**: Ca. 1874 (Tischbein (1874a), Germany).

**Hibernacula**: DT (29), DTCL (1), M (2), U (2).

**Sources**: Tischbein (1874a) [*Ichneumon castaneus* Gr.], Lauterborn (1936) [*I. castaneus*], Heinrich (1949b), Rasnitsyn (1959) [*Stenichneumon castaneus* Grav.], Valemberg and Vago (1974a) and Valemberg (1976a), Bauer (1984), Hinz and Kreissl (1993), Tereshkin (2004), Schmidt and Zmudzinski (2005).

#### Aoplus defraudator

(Wesmael, 1845)

7D604B87-A33D-5CAE-901F-03AEADD62C7C

##### Notes

None.

**Status**: Confirmed.

**Records**: Austria (6), Belgium (4)*, Denmark (1)**, France (6), Germany (14), Norway (1)*, Poland (2), Russia (1), the Netherlands (3), United Kingdom (3)*.

**First record**: Ca. 1863 (Tischbein (1863), Germany).

**Hibernacula**: DT (46), DTCL (3), LT (1), M (4), U (7).

**Sources**: Tischbein (1863) [*Ichneumon angustus* Tischb.] and Tischbein (1871) [*I. defraudator* Koch], Berthoumieu (1895b) [*I. defraudator* Koch], Seyrig (1923) [*Barichneumon defraudator* Koch], Heinrich (1927) [*Stenichneumon defraudator* Wesm.], Heinrich (1949b)Heinrich (1949a)/Heinrich (1949b) and Heinrich (1951), Rasnitsyn (1964) [*S. defraudator* Kriechb.], Valemberg (1975), Valemberg (1976b) and Valemberg (1982), Bauer (1984), Hinz and Kreissl (1992) and Hinz and Kreissl (1993), Sebald et al. (2001), Schmidt and Zmudzinski (2005), Verheyde and Quicke (2022); Unpublished: iNaturalist, iRecord, Naturbasen, Observation.org, Pers. obs.

**Also mentioned in**: Tischbein (1874a) [*I. defraudator* Koch], Morley (1903) [*S. defraudator* Wesm.], Constantineanu (1959) [*S. defraudator* Wesm.].

#### Aoplus deletus

(Wesmael, 1845)

6367577E-89ED-5DF1-97E4-8D7FC59A4D49

##### Notes

None.

**Status**: Confirmed.

**Records**: Belarus (1), Belgium (1), Germany (1).

**First record**: Ca. 1900 (Athimus (1901b), Belgium).

**Hibernacula**: DT (1), DTCL (1), M (1).

**Sources**: Athimus (1901b) [*Ichneumon praestigiator*; *I. deletus* Wesm.], Bauer (1984), Tereshkin (2004).

**Also mentioned in**: Sebald et al. (2000).

#### Aoplus mustela

(Kriechbaumer, 1895)

FF3F2DF9-6C0C-590A-BB63-DA4DC41515EB

##### Notes

None.

**Status**: Confirmed.

**Records**: Belarus (1), France (1), Germany (2), Russia (1).

**First record**: March 1894 (Kriechbaumer (1895), Germany).

**Hibernacula**: DT (2), DTCL (1), M (2), U (1).

**Sources**: Berthoumieu (1895b) [*Ichneumon mustela* Kriech.], Kriechbaumer (1895) [*I. mustela*], Rasnitsyn (1964) [*Stenichneumon mustela* Kriechb.], Bauer (1984), Tereshkin (2004).

#### Aoplus ochropis

(Gmelin, 1790)

1A937FE5-E1C9-5EA0-83A3-FE5A673F165D

##### Notes

Reported to hibernate by Ulrich (2001); however, it is not clear on what basis. This survey undertaken near Göttingen (Germany) consisted of monitoring fauna with soil photo-eclectors. No context on the finding was otherwise given. In November 2024, one specimen was found under dead wood in France (iNaturalist). No other occurrences are known, including specimens found early or late in the year (GBIF). Hibernation could be facultative in this species.

**Status**: Confirmed*.

**Records**: France, 9.XI.2024.

**Hibernacula**: DT (1).

**Sources**: iNaturalist.

**Also mentioned in**: Ulrich (2001).

#### Aoplus theresae

(Berthoumieu, 1896)

946B125E-1895-599B-93C0-FAE8DD4DF835

##### Notes

None.

**Status**: Confirmed.

**Records**: Germany (2).

**First record**: May 1947 or 1948 (Heinrich (1949b), Germany).

**Hibernacula**: DTCL (1), U (1).

**Sources**: Heinrich (1949b) [*Aoplus sphinx*], Bauer (1984).

**Also mentioned in**: Sebald et al. (2000), Bauer (2001).

#### Aoplus torpidus

(Wesmael, 1857)

9D83F163-848D-5050-9E33-5F0E178E66FD

##### Notes

Similar to other *Aoplus* spp. in Sebald et al. (2000) and Bauer (2001), but the original data source is unclear.

**Status**: Confirmed.

**Records**: Belarus (1), Germany (1).

**First record**: May 1947 or 1948 (Heinrich (1949b), Germany).

**Hibernacula**: DT (1), U (1).

**Sources**: Heinrich (1949b), Tereshkin (2004).

**Also mentioned in**: Sebald et al. (2000), Bauer (2001).

#### Baranisobas ridibundus

(Gravenhorst, 1829)

3523B23A-25FA-50AE-BA43-9536EE707FAC

##### Notes

Not to be confused with *Apaeleticus* and *Crytea* spp. This species was found hibernating for the first time in a grass tussock of *Molinia caerulea*.

**Status**: Confirmed*.

**Records**: United Kingdom, 17.I.2020.

**Hibernacula**: LV (1).

**Sources**: Pers. obs.

#### Barichneumon albicaudatus

(Fonscolombe, 1847)

C5B810D1-F265-50A1-A004-EEF0B93D09EE

##### Notes

The observation from Schmidt and Zmudzinski (2005) in November is the only recent observation from this species. Not to be confused with *Ichneumon angustatus* sensu Tischbein 1863, which was synonymised with *Aoplus defraudator*.

**Status**: Confirmed.

**Records**: France (1), Germany (1).

**First record**: Ca. 1895 (Berthoumieu (1895a), France).

**Hibernacula**: M (1), U (1).

**Sources**: Berthoumieu (1895a) [*Ichneumon angustatus* Trent.], Schmidt and Zmudzinski (2005).

**Also mentioned in**: Morley (1903) [*Barichneumon angustatus* Wesm.].

#### Barichneumon bilunulatus

(Gravenhorst, 1829)

FA5351B9-645F-5EE9-B9E2-367A0647E4BA

##### Notes

Johnson's record is not to be confused with *Coelichneumon bilineatus* (Gmelin, 1790). All findings are associated with pine trees and so are most of the hosts, with *Bupalus piniaria* (Linnaeus, 1758) as the most widespread example (Yu et al. 2016). Seyrig (1923) found his specimen between pine needles, while Johnson (1920) mentions the species’ presence below pine bark. The specimen from Russia (iNaturalist) was found in a pine forest.

**Status**: Confirmed.

**Records**: France (1), Ireland (1), Russia (1)*.

**First record**: Ca. 1920 (Johnson (1920), Ireland).

**Hibernacula**: DT (2), DV (1).

**Sources**: Johnson (1920) [*Barichneumon bilineatus* Gr.], Seyrig (1923) [*Cratichneumon bilunulatus* Gr.]; Unpublished: iNaturalist.

#### Barichneumon chionomus

(Wesmael, 1845)

D421290A-F6B1-5891-B3A7-D0711D28E289

##### Notes

Only discovered in grass tussocks so far. The records from Heinrich may concern the same specimen.

**Status**: Confirmed.

**Records**: France (1), Germany (2), United Kingdom (7)*.

**First record**: Ca. 1895 (Berthoumieu (1895a), France).

**Hibernacula**: LV (11), M (1), U (1).

**Sources**: Berthoumieu (1895a) [*Ichneumon chionomus* Wesm.], Heinrich (1949a) and Heinrich (1951), Storey et al. (2025); Unpublished: Pers. obs.

**Also mentioned in**: Morley (1903), Constantineanu (1959).

#### Barichneumon derogator

(Wesmael, 1845)

F8DEDF7F-7E8C-5787-AEBD-C414A436DD0D

##### Notes

Found in the Netherlands, either by beating a pine tree or in the tussocks of *Molinia caerulea*.

**Status**: Confirmed*.

**Records**: The Netherlands, 5.II.2020.

**Hibernacula**: LT/LV (1).

**Sources**: Observation.org.

#### Barichneumon nigrifemur

(Tischbein, 1881)

8AE39FB1-01C8-5C9A-997B-55C2B6E88F23

##### Notes

The taxonomic status is unclear, partially due to having no type material (Hinz and Horstmann 2000). It is reported as hibernator by Aerts (1957), more specifically below bark in the region of Krefeld.

**Status**: Taxon requiring further verification.

**Records**: Germany, 1919-1957.

**Hibernacula**: DT (1).

**Sources**: Aerts (1957) [*Exephanes nigrifemur* Tischb.].

#### Barichneumon peregrinator

(Linnaeus, 1758)

C0BD5D48-503D-503D-96FD-1EEE611AFA16

##### Notes

Not to be confused with *Ichneumon peregrinator* sensu Thunberg, now *I. oblongus* (e.g. Hedwig (1927)). In the Netherlands, specimens of *B. peregrinator* were found on the wooden information panels of a nature reserve during two consecutive winter seasons (2022–2023 and 2023–2024) so far.

**Status**: Confirmed.

**Records**: Denmark (1)*, Finland (1)*, France (1), Poland (1), the Netherlands (7), United Kingdom (1)*.

**First record**: Ca. 1895 (Berthoumieu 1895a, France).

**Hibernacula**: DT (4), LT (3), M (1), O (13), U (1).

**Sources**: Berthoumieu (1895a) [*Ichneumon vaccilatorius* Grav.], Hedwig (1927) [*Barichneumon scriptorius* Thunb.], Van Dinther (1951); Unpublished: Laji.fi, Naturbasen, Observation.org, Pers. obs.

**Also mentioned in**: Morley (1903) [*B. vacillatorius* Grav.], Constantineanu (1959) [*B. scriptorius* Thunb.], Verheyde and Quicke (2022)

#### Barichneumon plagiarius

(Wesmael, 1848)

6EF36477-1D64-5B7F-B575-6273FF3115CA

##### Notes

Found at least twice in the region of Baden (Germany), in November and January of an undefined year. Most likely below bark, but this is not explicitly stated.

**Status**: Confirmed.

**Records**: Germany, 1980-2006 (2 records.).

**Hibernacula**: U (2).

**Sources**: Schmidt and Zmudzinski (2005).

#### Barichneumon praeceptor

(Thunberg, 1824)

B074C896-52BA-548C-8F43-64A23A4BD67E

##### Notes

Found at least twice in the region of Baden (Germany), in November and January of an undefined year. Most likely below bark, but this is not explicitly stated.

**Status**: Confirmed.

**Records**: Germany, 1980-2006 (2 recs.).

**Hibernacula**: U (2).

**Sources**: Schmidt and Zmudzinski (2005).

#### Bureschias subcylindricus

(Gravenhorst, 1829)

89BAA688-0D7E-556C-80A6-0115F32E253C

##### Notes

This species is very rare in Europe, but more common in highlands (> 1500 m), especially in the Balkan Region and Eastern Europe. Several females (including the holotype of *B. balcanicus*) were found hibernating on a dead tree in August (Heinrich 1936). In 1990, one more specimen was found at a lower height (600-900 metres) in the Rhodope mountains in Bulgaria, below the bark of a dead oak.

**Status**: Confirmed.

**Records**: Bulgaria (2).

**First record**: August 1935 (Heinrich (1936), Bulgaria).

**Hibernacula**: DT (2).

**Sources**: Heinrich (1936) [*Bureschias balcanicus*], Kolarov (1992) [*Bureschias subcilindricus* Grav.].

#### Chasmias motatorius

(Fabricius, 1775)

E691B8D3-6BD5-5533-A4F9-C09B9344A798

##### Notes

Similar to *C. paludator*. Differences are the colouration of the proximal flagellomeres (orange in *C. motatorius*) and the dorsal side of the hind tarsus (orange in *C. motatorius*). Around 16 observations could only be identified as belonging to this complex (*C. motatorius*/*paludator*), but none of these observations would constitute new country records. Common below bark, notably in Denmark and the United Kingdom.

**Status**: Confirmed.

**Records**: Austria (1)*, Belgium (11), Czechia (5)*, Denmark (28)*, Finland (1)*, France (32), Germany (20), Hungary (1)*, Latvia (2)*, Lithuania (1)*, Norway (2)*, Poland (3), Russia (5), Sweden (6)*, the Netherlands (18), Ukraine (1), United Kingdom (51).

**First record**: Ca. 1863 (Tischbein (1863), Germany).

**Hibernacula**: DT (228), DTCL (4), DV (5), LV (46), M (11), S (1), U (121).

**Sources**: Tischbein (1863) and Tischbein (1871) [*Chasmodes motatorius* Grav.], Berthoumieu (1894) [*C. motatorius* Grav.], Habermehl (1896) [*C. motatorius*], Morley (1903), Habermehl (1916) [*C. motatorius* Grav.], Pic (1917) [*C. motatorius* Grv.], Smits van Burgst (1918) [*C. motatorius* Grv.] Pic (1919) [*C. motatorius* Grv.], Hancock (1923) and Hancock (1925), Seyrig (1923) [*C. motatorius* F.], Seyrig (1926a), Hedwig (1927) [*C. motatorius* Grv.], Heinrich (1949a) and Heinrich (1951), Renken (1956) [*C. motatorius* Grav.], Starke (1956), Aerts (1957), Rasnitsyn (1959), Valemberg and Vago (1974a)/Valemberg and Vago (1974b), Valemberg and Vago (1975) and Valemberg and Vago (2013), Valemberg (1976a)/Valemberg (1976b), Valemberg (1978) and Valemberg (1982), Bauer (1984), Gokhman (1985), Hilpert (1989), Sebald et al. (2001), Schmidt and Zmudzinski (2005), Pénigot (2020), Varga et al. (2020), Verheyde and Quicke (2022); Unpublished: Arter DK, Artportalen, Artsobservasjoner, Coll. RBINS, Dabasdati, GBIF, Lajitietokeskus/FinBIF, iNaturalist, iRecord, Naturbasen, Naturgucker, Observation.org, Pers. obs.

**Also mentioned in**: Tischbein (1873a) [*C. motatorius* F.], Constantineanu (1959), Hinz and Horstmann (1999), Gokhman (2003).

#### Chasmias paludator

(Desvignes, 1854)

B3929C5B-EC8C-587D-8F6D-E8D5C0F70930

##### Notes

Similar to *C. motatorius*. Somewhat less common. One extraordinary finding was the presence of two females hibernating in the burrow of a *Megachile* sp., in a willow post. The ichneumonids must have entered the hole before the bee laid down the first of its cells (Boyd 1935).

**Status**: Confirmed.

**Records**: Belgium (14), Denmark (3)**, Finland (1)*, France (3), Germany (6), Lithuania (1)**, Poland (1)*, Russia (1), Sweden (3)**, the Netherlands (16), Ukraine (1)**, United Kingdom (8).

**First record**: Ca. 1915, March (Habermehl (1916), Germany).

**Hibernacula**: DT (63), DTCL (3), DV (2), O (2), U (5).

**Sources**: Habermehl (1916) [*Chasmodes paludicola* Wesm.], Seyrig (1923) [*C. paludicola* Wsm.], Boyd (1935) [*C. paludicola* Wesm.], Heinrich (1949a) [*C. paludicola* Wesm.], Rasnitsyn (1959) [*C. paludicola* Wesm.], Valemberg and Bosquit (1979), Bauer (1984) [*Chasmias paludicola* W.], Sebald et al. (2001), Schmidt and Zmudzinski (2005), Verheyde and Quicke (2022); Unpublished: Artportalen, Coll. RBINS, iNaturalist, Naturbasen, Observation.org.

**Also mentioned in**: Morley (1903), Hinz and Horstmann (1999).

#### Coelichneumon funebrator

Horstmann, 2006

1934434A-1FAF-569F-B5F7-DCDEC3B5F5C5

##### Notes

Supposedly, this species was discovered below the bark of a poplar.

**Status**: Incorrect.

**Records**: Romania, 8.XI.1926.

**Sources**: Constantineanu (1929a) [*Ichneumon funebris* F.].

**Hibernacula**: DT (1).

**Also mentioned in**: Constantineanu (1959).

#### Coelichneumon haemorrhoidalis

(Gravenhorst, 1820)

592625C0-B6F3-5150-82BB-9AA3843C9612

##### Notes

First mentioned in a paper from Weber and Weber (1986), later specified in Weber (1989). This species may have been in or near the cave by coincidence. Furthermore, it is unclear if the identification is correct as there have been some taxonomic changes (Riedel 2012).

**Status**: Unverified.

**Records**: Germany, ca. 1981.

**Hibernacula**: C (1).

**Sources**: Weber (1989) [*Ichneumon castaneiventris* Gravenhorst].

#### Coelichneumon leucocerus

(Gravenhorst, 1820)

292019DA-F78C-5DA8-AE5E-3E8ECDE115E4

##### Notes

As with many of Berthoumieu’s records, the context is unclear. The species is reported as hibernating beneath mosses. While it is common throughout Europe, no recent hibernation records are known.

**Status**: Incorrect.

**Records**: France, ca. 1894.

**Hibernacula**: M (1).

**Sources**: Berthoumieu 1894 [*Ichneumon leucocerus* Gr.].

**Also mentioned in**: Morley (1903), Constantineanu (1959).

#### Coelichneumon nigerrimus

(Stephens, 1835)

9E5DF801-448F-591C-BBC5-6636D1ACA1B7

##### Notes

Although there is no original data record, this species is reported as a hibernator (below bark or in the galleries of xylophagous insects) in Constantineanu (1959).

**Status**: Incorrect.

**Records**: Romania, 1929-1959.

**Hibernacula**: DT (1).

**Sources**: Constantineanu (1959) [*Coelichneumon derasus* Wesm.].

#### Coelichneumon sugillatorius

(Linnaeus, 1758)

E6CD04A1-5FEB-551C-946C-D400BB9F7ECB

##### Notes

As with many of Berthoumieu’s records, the context is unclear. There is one additional record from Austria in November 1984 (GBIF), but it is unclear if this specimen was hibernating.

**Status**: Incorrect.

**Records**: France, ca. 1894.

**Hibernacula**: M (1).

**Sources**: Berthoumieu (1894) [*Ichneumon sugillatorius* L.].

**Also mentioned in**: Morley (1903), Constantineanu (1959).

#### Cratichneumon coruscator

(Linnaeus, 1758)

431A5B7D-8BE0-5F77-A4E7-EDAA49C18CC2

##### Notes

It was included as hibernating in the list of Schmidt and Zmudzinski (2005), but no additional context was given here. This is a very common species and has over 500 occurrences on GBIF, but none of them indicates hibernation.

**Status**: Unverified.

**Records**: Germany, 1980-2006.

**Sources**: Schmidt and Zmudzinski (2005).

#### Cratichneumon culex

(Muller, 1776)

678B6FF1-9998-58ED-8F1A-137476E31F94

##### Notes

Despite many occurrences on GBIF, there have been no records of hibernation for over a century.

**Status**: Unverified.

**Records**: France, ca. 1895 (Berthoumieu 1895a).

**Sources**: Berthoumieu (1895a) [*Ichneumon annulator* Fabr.].

**Also mentioned in**: Morley (1903) [*Cratichneumon annulator* Fab.], Constantineanu (1959) [*C. annulator* Fabr.].

#### Cratichneumon sicarius

(Gravenhorst, 1829)

932116A7-810A-501E-8607-24581D0C8D01

##### Notes

Within the genus *Cratichneumon*, this species has the best documented cases of hibernation. In Belgium, it was found twice in a grass tussock (Observation.org) and in Germany, multiple times (not specified) below moss on a dead tree (Bauer 1984).

**Status**: Confirmed.

**Records**: Belgium (2)*, Germany (1).

**First record**: 1984 (Bauer (1984), Germany).

**Hibernacula**: DT (1), LV (2).

**Sources**: Bauer (1984); Unpublished: Observation.org.

**Also mentioned in**: Sebald et al. (2000).

#### Cratichneumon versator

(Thunberg, 1824)

8EA339E7-D697-53E8-B4EA-256A40D95435

##### Notes

There are indications based on phenology (GBIF) that this species hibernates, but more data are needed, bearing in mind the ecology of other species in the genus.

**Status**: Unverified.

**Records**: Romania, ca. 1959.

**Hibernacula**: DT (1).

**Sources**: Constantineanu (1959).

#### Cratichneumon viator

(Scopoli, 1763)

19F9A744-4523-51E5-A4AA-0E09E0943522

##### Notes

None.

**Status**: Confirmed.

**Records**: Germany (3).

**First record**: Ca. 1871 (Tischbein (1871), Germany).

**Hibernacula**: DT (2), U (1).

**Sources**: Tischbein (1871) [*Ichneumon nigritarius* W.], Bauer (1984) [*Cratichneumon nigritarius* Grav.], Schmidt and Zmudzinski (2005).

**Also mentioned in**: Berthoumieu (1895a) [*I. nigritarius* Grav.], Morley (1903) [*C. nigritarius* Grav.].

#### Crytea erythraea

(Gravenhorst, 1820)

AAF61E18-B42C-5E6F-A0B3-3C33F71B06A9

##### Notes

This species has become very common over the last decade in Belgium and to a lesser extent in the Netherlands. It most probably followed its most used host, the lepidopteran *Pechipogo plumigeralis* Hübner (Erebidae), which is associated with ivy. Systematic field research on several localities in Belgium seems to demonstrate how only some adults within a population hibernate in the evergreen ivies. Additionally, there are also observations of adults in grass tussocks and litter.

**Status**: Confirmed.

**Records**: Belgium (10)*, France (2), the Netherlands (3)*.

**First record**: France, ca. 1895 (Berthoumieu 1895a).

**Hibernacula**: DV (1), LV (14), U (1).

**Sources**: Berthoumieu (1895a) [*Ichneumon erythraeus* Grav.]; Unpublished: Insecte.org, Observation.org.

**Also mentioned in**: Morley (1903) [*Melanichneumon erythraeus* Grav.].

#### Crytea sanguinator

(Rossi, 1794)

4021CD76-1F5C-547B-99CF-9BF66A5F4C57

##### Notes

Closely resembles *C. erythraea*; however, in *C. sanguinator*, the white orbits are narrower, the first tergite is dark and the temples are less narrowed.

**Status**: Confirmed.

**Records**: Belgium (2)*, France (1), Germany (1), Hungary (1)*.

**First record**: 1915-1922 (Seyrig (1923), France).

**Hibernacula**: DT (1), DV (2), LV (3), U (1).

**Sources**: Seyrig (1923) [*Melanichneumon sanguinator* Rossi], Schmidt and Zmudzinski (2005); Unpublished: Coll. RBINS, iNaturalist.

#### Ctenichneumon circulator

(Thomson, 1894)

2188E136-9CA5-5AF7-BE47-16DF5C68B411

##### Notes

In Bauer's list, it is included as hibernating, but no further details are given.

**Status**: Unverified.

**Records**: Germany, ca. 2001.

**Sources**: Bauer (2001) [*Ctenichneumon migratus circulator* Thoms.].

#### Ctenichneumon devylderi

(Holmgren, 1871)

C699FDC1-CBD4-5515-9C8A-B76D36E083B0

##### Notes

One specimen was found under the thick bark of a decaying tree in Romania, but bearing in mind the ecology of the genus, more data are needed.

**Status**: Unverified.

**Records**: Romania, 2006-2010.

**Hibernacula**: DT (1).

**Sources**: Lungu-Constantineanu and Constantineanu (2014).

#### Ctenichneumon funereus

(Geoffroy, 1785)

9FA69939-250B-59D2-8EF4-FE4D9AFEAE7C

##### Notes

None.

**Status**: Unverified.

**Records**: France, ca. 1895.

**Sources**: Berthoumieu (1895b) [*Amblyteles funereus* Fourcr.].

**Also mentioned in**: Morley (1903), Constantineanu (1959) [*Amblyteles funereus* Fourcr.].

#### Ctenichneumon inspector

(Wesmael, 1845)

F9E104C3-896C-5B40-A64E-18292FE46869

##### Notes

None.

**Status**: Unverified.

**Records**: France, ca. 1895.

**Sources**: Berthoumieu (1895b) [*Amblyteles inspector* Wesm.].

**Also mentioned in**: Morley (1903), Constantineanu (1959) [*Amblyteles inspector* Wesm.].

#### Ctenichneumon melanocastanus

(Gravenhorst, 1820)

635D4AEE-B626-5D5C-AE4A-54EA620CD455

##### Notes

None.

**Status**: Unverified.

**Records**: France, ca. 1895.

**Sources**: Berthoumieu (1895b) [*Amblyteles melanocastanus* Grav.].

**Also mentioned in**: Morley (1903), Constantineanu (1959) [*A. melanocastanus* Grav.].

#### Ctenichneumon nitens

(Christ, 1791)

9D1A56F1-24A5-50B2-98A4-968BF169FC30

##### Notes

None.

**Status**: Unverified.

**Records**: France, ca. 1895.

**Sources**: Berthoumieu (1895b) [*Amblyteles mesocastanus* Grav.].

**Also mentioned in**: Morley (1903) [*Ctenichneumon mesocastanus* Grav.], Constantineanu (1959) [*A. nitens* Christ].

#### Ctenichneumon repentinus

(Gravenhorst, 1820)

221CB48E-13DB-542C-859C-7214A40A5102

##### Notes

None.

**Status**: Unverified.

**Records**: France, ca. 1895.

**Sources**: Berthoumieu (1895b) [*Amblyteles repentinus* Grav.].

**Also mentioned in**: Morley (1903), Constantineanu (1959) [*A. repentinus* Grav.].

#### Diphyus amatorius

(Muller, 1776)

74846E90-E940-5B6B-B85A-83D6BEE23E8E

##### Notes

Seems to be associated with larger open grasslands and heathlands, where it can be found in a rather wide variety of hibernacula.

**Status**: Confirmed.

**Records**: Belgium (1)*, France (4), Germany (2), the Netherlands (2).

**First record**: 12.IX.1913 (Jeannel 1926, France).

**Hibernacula**: C (1), DT (2), DV (1), M (3), S (1), U (2).

**Sources**: Seyrig (1923) [*Amblyteles amatorius* L.], Jeannel (1926) [*A. amatorius* Müll.], Heinrich (1949a) [*A. amatorius* Müll.], Bauer (1984), Valemberg and Vago (2013), Verheyde and Quicke (2022); Unpublished: Coll. RBINS, iNaturalist, Observation.org.

#### Diphyus bicingulatus

(Gravenhorst, 1829)

46C2A53D-60AF-523B-8200-0C3741D33431

##### Notes

Unfortunately the finding context of the specimen mentioned in Bauer (2001) is unclear. This species is mostly confined to mountainous regions.

**Status**: Confirmed.

**Records**: Germany, 1980-2001.

**Sources**: Bauer (2001).

#### Diphyus castanopyga

(Stephens, 1835)

76A9E479-2A74-59BC-8F25-19FF947933BA

##### Notes

In limestone caves in Spain and the Netherlands, this species has been observed consistently, although often in low numbers between other more common species (Selfa and Escolà 1991, Vergoossen et al. In press). Exceptionally, there is one observation under tree bark from Luxembourg, but the area consisted of a former slag dump with many little niches between the stones. This is similar to one observation we have of *D. quadripunctorius* under bark, which normally also prefers caves exclusively. These specimens were possibly disrupted or exceptionally choose other hibernacula in the direct vicinity of the caves.

**Status**: Confirmed.

**Records**: Belgium (2)*, France (1)*, Germany (1), Grand Duchy of Luxembourg (1)**, Spain (8), the Netherlands (11).

**First record**: May 1947 or 1948 (Heinrich (1949b), Germany).

**Hibernacula**: B (1), C (33), DT (1), U (1).

**Sources**: Heinrich (1949b) [*Pseudamblyteles castanopygus* Steph.], Selfa and Escolà (1991), Ortiz-Sánchez and Fernández (2008), Fernández and Ruiz (2014), Verheyde and Quicke (2022), Vergoossen et al. (In press); Unpublished: iNaturalist, Observation.org, Pers. obs.

#### Diphyus catagraphus

(Kokujev, 1904)

59C0C92C-BF10-58FD-922D-17746682DABA

##### Notes

Unfortunately, the exact context of this finding is also unclear. The Russian specimens are reported from two regions: Moscow and Altai (Lake Teletskoye), bordering the Eastern Palaearctic Region.

**Status**: Confirmed.

**Records**: Russia, 1959-1964 (2 records).

**Sources**: Rasnitsyn (1964).

#### Diphyus fossorius

(Linnaeus, 1758)

CB5053E8-2108-52DC-B38E-2C7BD5281086

##### Notes

Similar to *D. quadripunctorius*, but typically it has stronger metasomal bands apically and the hind femora are completely orange. Due to the quite uncertain morphological and taxonomic status, it is difficult to say which older reports we can trust or if this is a valid species at all. One record from Sudetenland in Czechia (Hedwig 1943) - the original source 'Pax 1937' could not be found - and one uncertain record from Poland (Skalski 1973) were not included. Older synonyms should not be confused with *Ctenichneumon edictorius*, which does not hibernate.

**Status**: Confirmed.

**Records**: France (2), Germany (1), Poland (1), Romania (1), Spain (11).

**First record**: Ca. 1895 (Berthoumieu (1895b), France).

**Hibernacula**: C (27), M (1).

**Sources**: Berthoumieu (1895b) [*Amblyteles fossorius* Müll.], Arndt (1921) [*A. atratorius* F.], Seyrig (1926b) [*A. atratorius* F.], Collart (1941) [*A. fossorius* Lin.], Weber and Weber (1986) [*A. fossorius* L.], Selfa and Escolà (1991).

**Also mentioned in**: Morley (1903) [*Ctenichneumon fossorius* Grav.], Hedwig (1943) [*A. atratorius* Pz.], Constantineanu (1959) [*A. fossorius* Lin.], Skalski (1973), Skalski (1981), Kocot-Zalewska and Domagała (2020).

#### Diphyus gradatorius

(Thunberg, 1824)

BD01C32B-F564-5A9D-9FE6-3F705D1A75A9

##### Notes

This species is mostly confined to mountainous regions. Together with *D. palliatorius* and *D. trifasciatus*, it belongs to a complex of species where the females are hard to distinguish.

**Status**: Confirmed.

**Records**: Germany (2).

**First record**: Ca. 1947-1949 (Heinrich (1949a), Germany).

**Sources**: Heinrich (1949a) [*Amblyteles gradatorius* Thunb.], Bauer (2001).

#### Diphyus latebricola

(Wesmael, 1845)

4537D2FE-EEB2-5E1D-B15C-E69A46BD0CA7

##### Notes

One of the oldest hibernation records we have. It was described by Wesmael ‘inter arbustorum radices sub terra degens’ near Brussels - this could either mean between the roots of bushes in the soil, but also, more likely, below the decaying roots of a tree which fell over. This interpretation also matches Bauer (1984) (‘in Wurzeltellern’). A third specimen is reported from a cave in France. On several citizen-science portals, specimens are reported in April near dead wood, in the Baltic States and Russia.

**Status**: Confirmed.

**Records**: Belgium (1), France (1)*, Germany (1).

**First record**: Ca. 1844 (Wesmael (1844), Belgium).

**Hibernacula**: C (1), DTCL (2).

**Sources**: Wesmael (1844) [*Amblyteles latebricola*], Bauer (1984); Unpublished: Pers. obs.

**Also mentioned in**: Constantineanu (1959) [*Amblyteles latebricola* Wesm.].

#### Diphyus longigena

(Thomson, 1888)

38400B8A-566F-5FAE-BAA8-995E798ABEFA

##### Notes

Unfortunately, the specific context of this finding is unclear.

**Status**: Confirmed.

**Records**: Germany, 1980-2001.

**Sources**: Bauer (2001).

#### Diphyus longimanus

(Wesmael, 1857)

0CB528B5-A812-574F-B5E8-3B4AE0A68C27

##### Notes

Reported from a cave in Spain, the cova Palomera de Taús.

**Status**: Confirmed.

**Records**: Spain, 20.III.1895.

**Hibernacula**: C (1).

**Sources**: Selfa and Bordera (1995).

#### Diphyus luctatorius

(Linnaeus, 1758)

3488B5BD-3E47-597A-AD84-5B6791F1BED6

##### Notes

None.

**Status**: Confirmed.

**Records**: France (1), Germany (4).

**First record**: Ca. 1850-1871 (Tischbein (1863), Germany).

**Hibernacula**: C (1), DT (3), U (1).

**Sources**: Tischbein (1863), Tischbein (1871) and Tischbein (1873b) [*Ichneumon luctatorius* L.], Heinrich (1949a) [*Amblyteles culpatorius* Grav.], Dobat (1963a) [*Amblyteles culpatorius* Grav.].

#### Diphyus mercatorius

(Fabricius, 1793)

2F59827B-93AF-546D-8E6C-B0D9829035DE

##### Notes

No details on the observation in Sebald et al. (2000). Uncertain records exist for Austria (iNaturalist).

**Status**: Confirmed.

**Records**: Germany (2), Spain (5), Switzerland (1).

**First record**: 1949-1962 (Aellen and Strinati (1962), Switzerland).

**Hibernacula**: C (9), U (1).

**Sources**: Aellen and Strinati (1962) [*Amblyteles infractorius* L.], Dobat (1975) [*A. infractorius* Panz.], Selfa and Escolà (1991), Sebald et al. (2000).

**Also mentioned in**: Strinati (1966) [*A. infractorius*].

#### Diphyus monitorius

(Panzer, 1801)

53FC67E7-077C-5940-8FE2-855350B1AE86

##### Notes

One additional uncertain record for Spain, mentioned in Selfa and Bordera (1995). They reported Diphyus cf. monitorius ex *Miniopterus schreibersi*, common bent-wing bat.

**Status**: Confirmed.

**Records**: Poland (1), Spain (1).

**First record**: 5.VI.1968 (Skalski (1973), Poland).

**Hibernacula**: C (3).

**Sources**: Skalski (1973) [*Diphyus manitorius* Pz.], Selfa and Escolà (1991)

**Also mentioned in**: Skalski (1981) [*Diphyus manitorius* Pz.], Selfa and Bordera (1995), Kocot-Zalewska and Domagała (2020).

#### Diphyus ochromelas

(Gmelin, 1790)

0E3F2EC6-4CBD-5FAC-8974-5977C2CCD675

##### Notes

Seyrig (1923) reports several individuals at a height of more than 800 metres, in grass tussocks.

**Status**: Confirmed.

**Records**: France (2).

**First record**: Ca. 1895 (Berthoumieu (1895b), France).

**Hibernacula**: M (1), LV (2).

**Sources**: Berthoumieu (1895b) [*Amblyteles negatorius* Fabr.], Seyrig (1923) [*Spiloteles negatorius* F.].

**Also mentioned in**: Morley (1903) [*Spilichneumon negatorius* Fab.].

#### Diphyus palliatorius

(Gravenhorst, 1829)

1D3A3AAC-E406-5A94-AEFE-4D3AC7937E2B

##### Notes

Similar to *D. gradatorius* and *D. trifasciatus*, this species forms a complex of species mostly specialised in cave hibernation. Of the six records not integrated in the paper from this complex, there are multiple (first) records for Italy (iNaturalist) which most likely consist of *D. palliatorius*. Northern specimens of *D. palliatorius* are more darkly coloured.

**Status**: Confirmed.

**Records**: Czechia (2), France (1)*, Germany (4), Grand Duchy of Luxembourg (5), Romania (1), Russia (1), Spain (6), the Netherlands (3), Ukraine (1)*, United Kingdom (3).

**First record**: May 1947 or 1948 (Heinrich (1949b), Germany).

**Hibernacula**: B (1), C (29), U (3).

**Sources**: Heinrich (1949b) [*Pseudamblyteles palliatorius* Gr.], Decou and Decou (1961) [*Amblyteles palliatorius* Grav.], Rasnitsyn (1964) [*A. palliatorius* Grav.], Weber (1989), Selfa and Escolà (1991), Bauer (2001), Šedivý and Dvořák (2002), Sebald and Weber (2013), Baird and Shaw (2019), Verheyde and Quicke (2022), Vergoossen et al. (In press); Unpublished: GBIF, iNaturalist, Pers. obs.

**Also mentioned in**: Šedivý (2001), Weber (2014).

#### Diphyus quadripunctorius

(Müller, 1776)

F53320E0-0F6D-5550-B686-7B370D7C3D61

##### Notes

Similar to *D. fossorius*. The most common species with winter and summer diapause throughout Europe, specialised in natural caves, but also present in buildings and fortifications. Enormous aggregations can be reached. A group of 791 individuals was counted at one locality in 2022, which is probably the largest cluster of ichneumonids ever measured. Long-term research in the Netherlands shows how mortality can become as high as 50%, which is remarkable in comparison to the hibernating species associated with other hibernacula (Vergoossen et al. In press). One third of the specimens reported in this catalogue belong to this species.

**Status**: Confirmed.

**Records**: Albania (1), Austria (13), Belgium (165), Bosnia and Herzegovina (1), Bulgaria (2), Croatia (2), Czechia (48), Denmark (1)**, France (27), Germany (63), Grand Duchy of Luxembourg (9), Hungary (5), Italy (27), Norway (10)*, Poland (9), Portugal (2), Romania (49), Serbia (5), Slovenia (5), Spain (16), Sweden (1)*, Switzerland (16), the Netherlands (154), Ukraine (7)*, United Kingdom (14).

**First record**: Ca. 1865 (Frivaldszky 1865, Hungary).

**Hibernacula**: B (409), C (9250), DT (3), S (1), U (5).

**Sources**: Frivaldszky (1865) [*Ichneumon natatorius*], Mocsáry (1877) [*Amblyteles natatorius* Fabr.], Szilády (1914) [*A. quadripunctorius* Müll.], Jeannel (1926) [*A. quadripunctorius* Müll.], Buresch (1934) [*A. bipunctatus*], Leruth (1939) [*A. quadripunctorius* Müll.], Collart (1941) [*A. quadripunctorius* Müll.], Franciscolo (1955) [*A. quadripunctorius* Müll.], Kowalski (1955) [*A. quadripunctorius* Müll.], Decou and Decou (1961) [*A. quadripunctorius* Müll.], Guéorguiev and Beron (1962) [*A. bipunctatus*], Aellen and Strinati (1962) [*A. quadripunctorius* Müll.], Dobat (1975) [*A. quadripunctorius* Müll.], Strouhal and Vornatscher (1975) [*A. quadripunctorius*], Weber and Weber (1986) [*A. quadripunctorius* Müll.], Weber (1989), Selfa and Escolà (1991), Bertolani et al. (1994), Šedivý (2001), Šedivý and Dvořák (2002), Ozimec (2002), Parenzan and Belmonte (2002), Rathgeber (2004), Kubátová and Dvořák (2005), Novak (2005) [*A. quadripunctorius* Müll.], Dethier (2007), Lana et al. (2008) [*A. quadripunctorius*], Ortiz-Sánchez and Fernández (2008), Novak et al. (2010), Fernández and Ortíz-Sánchez (2013), Penado et al. (2013), Sebald and Weber (2013), Weber (2014), Fabbri and Poletti (2015), Lana and Sella 2016, Presetnik et al. (2016), Dundarova et al. (2018), Baird and Shaw (2019), Žikić et al. (2020), Lenart et al. (2022), Verheyde and Quicke (2022), Canciani and Cechini (2023), Vesović et al. (2024); Unpublished: Artsobservasjoner, BioPortal, iNaturalist, Insecte.org, Naturbasen, Naturgucker, Observation.org, Pers. obs., Pers. obs. (G. Artmann), UKRbin.

**Also mentioned in**: Bokor (1921) [*A. quadripunctorius* Müll.], Strinati (1966) [*A. quadripunctorius*], Dvořák (2002), Weber (2014), Beron (2016), Vas and Kutasi (2016), Kocot-Zalewska and Domagała (2020), Lana et al. (2021), Vergoossen et al. (In press).

#### Diphyus raptorius

(Linnaeus, 1758)

98F35CAA-BEDF-58F3-8AB6-C39F8A6808BE

##### Notes

Similar to *D. septemguttatus*. In this latter species, there is no white spot on the fourth metasomal segment or it is much smaller than the spot on the fifth tergite (both are of equal size in *D. raptorius*). *D. raptorius* was the first ichneumonid wasp to be reported hibernating. Rossius (1790) mentions a specimen he found below oak bark in his monograph: “Captus etiam intra corticem & lignum Quercus m. Januar”. The species is widespread throughout Europe. In Denmark, it appears to be more common than in other areas.

**Status**: Confirmed.

**Records**: Belarus (4), Belgium (1), Bulgaria (3), Czechia (5)*, Denmark (17)*, France (12), Germany (16), Grand Duchy of Luxembourg (1)**, Hungary (1)*, Italy (1), Lithuania (2)**, Romania (5), Russia (5), Serbia (1)**, Spain (1)*, Sweden (4), United Kingdom (3).

**First record**: Ca. 1790 (Rossius 1790, Italy).

**Hibernacula**: DT (148), DTCL (1), M (5), S (20), U (1).

**Sources**: Rossius (1790) [*Ichneumon raptorius*], Wesmael (1844) [*Amblyteles gravenhorstii*], Tischbein (1863), Tischbein (1871) and Tischbein (1873b) [*I. raptorius* Gr.; *A. gravenhorstii* W.], Holmgren (1864) [*A. Gravenhorsti*], Berthoumieu (1895b) [*A. Gravenhorsti* Wesm.], Pic (1916) and Pic (1917) [*I. raptorius* Grav.; *A. Gravenhorsti* Wesm.], Seyrig (1923) and Seyrig (1926b) [*Spiloteles gravenhorsti* Wsm.; *S. quadriguttorius* Thunb.], Hancock (1923) and Hancock (1925) [*Spilichneumon gravenhorsti* Wesm.], Constantineanu (1929a) [*A. Gravenhorsti* Wesm.], Heinrich (1949a) [*A. gravenhorsti* Wesm.], Constantineanu (1959) [*Amblyteles quadriguttorius* Thunb.], Rasnitsyn (1959) [*A. quadriguttorius* Thunb.], Bauer (1984) [*Spilichneumon raptorius* L.], Kolarov (1992), Valemberg and Vago (2013); Unpublished: Arter DK, Artportalen, Facebook (European Ichneumonidae), iNaturalist, Insecte.org, Naturbasen, Naturgucker, Pers. obs., Tereshkin 2014.

**Also mentioned in**: Morley (1903) [*Ichneumon raptorius* Grav.].

#### Diphyus restitutor

(Wesmael, 1859)

C53809B8-EE91-5244-9539-C5801D419EF9

##### Notes

Superficially resembles *D. fossorius* and *D. quadripunctorius*, but the hind femora are black and apically, there is a white spot on the metasoma. It appears to be connected to open grasslands and/or heathlands, similar to *D. amatorius*. There is one record from in a small building in the Netherlands, but so far, all other specimens were found below tree bark.

**Status**: Confirmed.

**Records**: Belarus (1), Belgium (1)*, Germany (1), the Netherlands (2).

**First record**: 29.XI.1986 (Tereshkin (2014), Belarus).

**Hibernacula**: B (1), DT (3), U (1).

**Sources**: Sebald et al. (2000), Verheyde and Quicke (2022); Unpublished: Observation.org, Pers. obs., Tereshkin (2014).

**Also mentioned in**: Tereshkin (2011).

#### Diphyus salicatorius

(Gravenhorst, 1820)

3809F1BB-7C58-5EBA-AAB6-AA0EEFECEB44

##### Notes

There is one record from Sweden, December 1911, but hibernation is unsure (GBIF). Tischbein (1863) reports a remarkable aggregation of females in an old, abandoned ant nest of *Formica flava*.

**Status**: Confirmed.

**Records**: Austria (1), Belarus (1), Belgium (9), Czechia (2)*, France (3), Germany (5), Lithuania (1)**, Norway (1)*, Romania (1), Russia (3), the Netherlands (1)*, United Kingdom (4).

**First record**: Ca. 1863 (Tischbein (1863), Germany).

**Hibernacula**: C (1), DT (49), M (5), O (1), U (4).

**Sources**: Tischbein (1863) [*Amblyteles indocilis* W.], Berthoumieu (1895b) [*A. indocilis* Wesm.], Athimus (1901b) [*A. indocilus* Wesm.], Seyrig (1923) [*Spiloteles indocilis* Wsm.], Collart (1941) [*A. indocilis* Wesm.], Heinrich (1949a) [*A. indocilis* Wesm.], Aerts (1957) [*A. indocilis* Wesm.], Rasnitsyn (1959) [*A. indocilis* Wesm.], Valemberg (1975) [*A. indocilis* Wesm.], Bauer (1984) [*Diphyus indocilis* W.], Hinz and Kreissl (1993) [*D. indocilis* Wesm.], Verheyde (2023); Unpublished: Artsobservasjoner, iNaturalist, Observation.org, Pers. obs., Tereshkin (2014).

**Also mentioned in**: Tischbein (1871) and Tischbein (1874b) [*Amblyteles indocilis* W.], Morley (1903) [*A. indocilis* Wesm.], Heinrich (1951) [*Pseudamblyteles indocilis* Wesm.], Bauer (1961) [*A. indocilis* Wesm.], Tereshkin (2011).

#### Diphyus septemguttatus

(Gravenhorst, 1829)

DB73CCD5-D2F5-5B67-BCCE-2BAB962C82EF

##### Notes

Similar to *D. raptorius*. *D. septemguttatus* seems to be slightly more common in mountainous regions (Seyrig 1923, Bauer 1984, Artsobservasjoner). One uncertain hibernation record exists from February 1914 in Sweden (GBIF).

**Status**: Confirmed.

**Records**: Austria (1), Belgium (1), France (3), Germany (2), Norway (1)*, Russia (1)*.

**First record**: Ca. 1900 (Athimus (1901b), Belgium).

**Hibernacula**: DT (4), M (6), S (1).

**Sources**: Athimus (1901b) [*Amblyteles septemguttatus* Gr.], Seyrig (1923) [*Spiloteles 7 guttatus* Gr.], Aerts (1957) [*A. septemguttatus* Gr.], Bauer (1984) [*Spilichneumon septemguttatus* Grav.], Hinz and Kreissl (1992); Unpublished: Artsobservasjoner, iNaturalist.

#### Diphyus tricolor

(Kim, 1955)

BFA89F32-B3BC-5D80-964B-EC6444F17D03

##### Notes

The sole hibernation record is of one found in June at 1200 metres altitude in the Alps (Heinrich 1949b).

**Status**: Confirmed.

**Records**: Germany, IV.1947-1948.

**Sources**: Heinrich (1949b).

#### Diphyus trifasciatus

(Gravenhorst, 1829)

944F2DC2-2A20-5A3E-BC19-F9C6107D5D46

##### Notes

Similar to *D. gradatorius* and *D. palliatorius*. The finding context of Bauer (2001) is unclear.

**Status**: Confirmed.

**Records**: Belgium (1), Germany (1), Grand Duchy of Luxembourg (1).

**First record**: Ca. 1900 (Athimus (1901b), Belgium).

**Hibernacula**: C (1), M (2), U (1).

**Sources**: Athimus (1901b) [*Amblyteles trifasciatus* Gr.], Bauer (2001), Sebald and Weber (2013).

#### Eutanyacra crispatoria

(Linnaeus, 1758)

EE24206B-738F-5763-BFFD-1DE62D7DC83D

##### Notes

This species uses a remarkably wide range of hibernacula. It has been found on/below stones and tree bark, but also on at least four different species of plants (tussocks).

**Status**: Confirmed.

**Records**: Belgium (1), France (2), Germany (3), Grand Duchy of Luxembourg (1)**, Russia (1), the Netherlands (1)*, United Kingdom (5).

**First record**: Ca. 1871 (Tischbein (1871), Germany).

**Hibernacula**: DT (5), LV (5), M (2), S (2).

**Sources**: Tischbein (1871) [*Amblyteles crispatorius* L.], Berthoumieu (1895b) [*A. crispatorius* Lin.], Athimus (1901b) [*A. crispatorius* Lin.], Rasnitsyn (1959) [*A. crispatorius* L.], Bauer (1984); Unpublished: iNaturalist, Insecte.org, Naturgucker, Observation.org, Pers. obs.

**Also mentioned in**: Morley (1903) [*A. crispatorius* Lin.], Constantineanu (1959) [*A. crispatorius* Lin.].

#### Eutanyacra glaucatoria

(Fabricius, 1793)

16409C16-9DF3-54EF-B1D5-FAB62C93AC81

##### Notes

Rasnitsyn (1964) explicitly mentions it as a new hibernator from the region of Moscow, but it was later reported by Dobat (1978) with an earlier observation date. Weber and Weber (1986) also report the species, but their identification was later corrected to *D. quadripunctorius* (Weber 1989).

**Status**: Confirmed.

**Records**: Germany (1), Russia (1).

**First record**: 13.VIII.1934 (Germany, Dobat (1978)).

**Hibernacula**: C (1), U (1).

**Sources**: Rasnitsyn (1964) [*Amblyteles obesus* Berth.], Dobat (1978) [*A. glaucatorius* Fabr.].

#### Eutanyacra jucunda

(Kriechbaumer, 1882)

9884AFC9-64AA-5D69-A776-6ADA4D04AD6F

##### Notes

Although this species was caught with pitfall traps and not directly observed in hibernacula, we decided to integrate this record. The traps were placed directly near the hibernacula in the caves and other hibernating species in the genus are known. This species is very rare in Europe. It is only known from the Carpathian Basin (Vas and Kutasi 2016).

**Status**: Confirmed.

**Records**: Hungary, 5.VII.2012-13.XI.2012.

**Sources**: Vas and Kutasi (2016).

#### Eutanyacra picta

(Schrank, 1776)

6D171C2F-6A38-5B3E-9883-6FFBB7BE9721

##### Notes

It took nearly 130 years until the first report of Berthoumieu (1895b) was confirmed. In September 2024, a female of *E. picta* was observed in Italy below large heaps of dung (mainly horse, cow and donkey). According to the observer, the species was in a state of quiescence and did not show any signs of flying away after moving the dung.

**Status**: Confirmed.

**Records**: France (1), Italy (1)*.

**First record**: Ca. 1895 (Berthoumieu (1895b), France).

**Hibernacula**: DV (1), M (1).

**Sources**: Berthoumieu (1895b) [*Ichneumon pictus* Grav.]; Unpublished: iNaturalist.

**Also mentioned in**: Morley (1903) [*Amblyteles vadatorius* Illig.], Constantineanu (1959) [*A. vadatorius* Ill.].

#### Exephanes ischioxanthus

(Gravenhorst, 1829)

E4A14BE8-3165-5BF5-870E-BEA45982661D

##### Notes

*E. ischioxanthus* is the most common species in the genus and easily recognised. It is usually found in smaller numbers in caves than *Amblyteles* or *Diphyus* species. The highest reported aggregation is 39, in the limestone caves in the Netherlands (Vergoossen et al. In press). One record from Sudetenland in Czechia was not included as the source is unclear and possibly refers to integrated sources (Hedwig 1943).

**Status**: Confirmed.

**Records**: Belgium (4), Czechia (5), France (2), Germany (19), Grand Duchy of Luxembourg (6), Poland (7), Russia (1)*, Slovakia (1), Slovenia (1), Spain (1), the Netherlands (21), United Kingdom (3).

**First record**: Ca. 1895 (Berthoumieu (1895b), France).

**Hibernacula**: B (1), C (355), LV (1), M (1), S (1), U (2).

**Sources**: Berthoumieu (1895b) [*Exephanes hilaris* Grav.], Morley (1903) [*E. hilaris* Grav.], Seyrig (1923) [*E. hilaris* Gr.], Hancock (1925) [*E. hilaris* Grav.], Griepenburg (1933), Griepenburg (1934) and Griepenburg (1935) [*E. hilaris* Grav.], Pax and Maschke (1935) [*E. hilaris* Grav.], Leruth (1939) [*E. hilaris* Grav.], Kowalski (1955) [*E. hilaris* Grav.], Dobat (1963b) [*E. hilaris* Grav.], Skalski (1969), Hadjuk and Orłoszek (1970) [*E. hilaris* Grav.], Dobat (1975), Selfa and Escolà (1991), Šedivý and Dvořák (2002), Novak (2005) [*E. hilaris* Grav.], Schmidt and Zmudzinski (2005), Dethier (2007) [*E. hilaris* Grav.], Sebald and Weber (2013), Kováč (2019), Verheyde and Quicke (2022), Vergoossen et al. (In press); Unpublished: GBIF, iNaturalist, iRecord, Obervation.org

**Also mentioned in**: Hedwig (1943) [*E. hilaris* Grv.], Constantineanu (1959) [*E. hilaris* Grav.], Skalski (1981), Weber (2014), Kocot-Zalewska and Domagała (2020).

#### Exephanes occupator

(Gravenhorst, 1829)

8AE25835-C8F4-50D7-93C1-8D20E8E0981D

##### Notes

Very similar to *E. venustus*, collecting a specimen is necessary (Hinz and Horstmann 2000). A more thorough study on the ecology of this species was made by Zwolfer (1962).

**Status**: Confirmed.

**Records**: Germany (2), United Kingdom (1).

**First record**: 1926 (Büttner (1932), Germany).

**Hibernacula**: C (3).

**Sources**: Büttner (1932), Baird and Shaw (2019); Unpublished: GBIF.

**Also mentioned in**: Zwolfer (1962).

#### Exephanes rhenanus

Habermehl, 1918

D57B68EE-A920-5851-AA1E-59BF2991642F

##### Notes

Similar to *E. riesei*, which also has a yellow hind tibia and a similar spot on the metasoma. However, in most specimens of *E. rhenanus*, the proximal flagellomeres are yellow to brown. The first record of two females hibernating remains without location (Hinz and Horstmann 2000).

**Status**: Confirmed.

**Records**: Czechia (1), Germany (1)*, Grand Duchy of Luxembourg (2), Russia (2)*.

**First record**: Ca. 2000 (Hinz and Horstmann (2000), no location).

**Hibernacula**: C (8).

**Sources**: Šedivý and Dvořák (2002), Sebald and Weber (2013); Unpublished: GBIF, iNaturalist.

**Also mentioned in**: Hinz and Horstmann (2000), Weber (2014).

#### Exephanes riesei

(Habermehl, 1916)

2C737DA2-DF6C-5584-9106-BF145094C92B

##### Notes

Similar to *E. rhenanus*; however, in most specimens, the proximal flagellomeres are blackish (Hinz and Horstmann 2000). It is rather uncommon in limestone caves (Vergoossen et al. In press).

**Status**: Confirmed.

**Records**: Belgium (1)*, Czechia(7), France (1)**, Germany (6), Grand Duchy of Luxembourg (1), Slovakia (2), the Netherlands (5).

**First record**: 14.VII.1985 (Weber (1989), Germany).

**Hibernacula**: C (38).

**Sources**: Weber (1989) [*Exephanes amabilis* Kriechb.], Šedivý (2001) [*E. amabilis* Kriechb.], Kovác et al. (2002) [*E. amabilis* Kriechb.], Šedivý and Dvořák (2002), Sebald and Weber (2013), Verheyde and Quicke (2022); Unpublished: GBIF, iNaturalist, Observation.org.

**Also mentioned in**: Bauer (2001) [*Exephanes hoerhammeri* Heinr.], Weber (2014), Vergoossen et al. (In press).

#### Exephanes tauricus

Hinz, 2000

3195E155-FFA7-55DA-AF73-95EB32CBABDB

##### Notes

Reported from the Middle East, in the west of Iran in the Barezard Cave.

**Status**: Confirmed.

**Records**: Iran, 28.III.2015.

**Sources**: Darvishnia et al. (2018).

**Hibernacula**: C (8).

#### Exephanes venustus

(Tischbein, 1876)

568080BB-F61C-541D-BC46-E5CFF346887B

##### Notes

Similar to *E. occupator*. Two records exist of four and ten specimens from caves in Czechia.

**Status**: Confirmed.

**Records**: Czech Republic, 29.I.1998 (2 records).

**Hibernacula**: C (14).

**Sources**: Šedivý (2001) [*Exephanes caelebs* Kriechb.].

#### Gareila nivata

(Gravenhorst, 1820)

55CBB18E-9D23-5102-95A1-88BFBC1169C9

##### Notes

The report by Berthoumieu (1896) probably concerns the same specimen as Athimus (1901b), as this specimen has been reported as found in Belgium at more or less the same period in time.

**Status**: Confirmed.

**Records**: Belgium, ca. 1896.

**Hibernacula**: M (1).

**Sources**: Athimus (1901b) [*Ichneumon Tosquineti* Kriechb.].

**Also mentioned in**: Berthoumieu (1896) [*I. Tosquineti* Kriechb.], Rasnitsyn (1964) [*Barichneumon tosquineti* Kriechb.].

#### Hepiopelmus variegatorius

(Panzer, 1800)

C9575399-8782-59B7-959A-010F89FA9A51

##### Notes

Very easily recognised and not uncommon throughout Europe, so more data are necessary. The records of Lauterborn consist of specimens hibernating below bark together with other ichneumonid wasps. Should not be confused with *I. variegatorius* Holmgr. (= *Ichneumon albiornatus* Tischbein, 1879).

**Status**: Unverified.

**Records**: Germany (2).

**First record**: 30.IX.1925 (Lauterborn (1926), Germany).

**Hibernacula**: DT (2).

**Sources**: Lauterborn (1926) and Lauterborn (1936) [*Ichneumon variegatorius* Panz.]

#### Homotherus locutor

(Thunberg, 1824)

7CCC0576-18C0-5BCF-8F4F-4ACF4B83A130

##### Notes

A species which prefers to hibernate in smaller hibernacula, like grass tussocks and evergreen trees.

**Status**: Confirmed.

**Records**: France (2), Germany (1), Ireland (1), the Netherlands (1)*, United Kingdom (6).

**First record**: Ca. 1895 (Berthoumieu (1895b), France).

**Hibernacula**: DV (1), LT (1), LV (7), M (3), U (2).

**Sources**: Berthoumieu (1895b) [*Ichneumon albicinctus* Grav.], Morley (1903) [*Barichneumon albicinctus* Grav.], Johnson (1920) [*B. albicinctus* Gr.], Schmidt and Zmudzinski (2005), Pénigot (2020); Unpublished: Observation.org, Pers. obs.

**Also mentioned in**: Constantineanu (1959) [*Barichneumon locutor* Thunb.].

#### Homotherus varipes

(Gravenhorst, 1829)

3D903411-D930-5883-BE70-602B8B5F02EA

##### Notes

Similar in ecology and phenology to *Homotherus locutor*, but with fewer records. There is no information on the context of the observation in Germany, but the specimen in the Netherlands was found hiding behind outdoor lamps on a building. Some additional field records exist from Denmark and the Netherlands in November (GBIF), but it is unclear if the specimens were hibernating.

**Status**: Confirmed.

**Records**: Germany (1), the Netherlands (1)*.

**First record**: 1980-2006 (Schmidt and Zmudzinski (2005), Germany).

**Hibernacula**: B (1), U (1).

**Sources**: Schmidt and Zmudzinski (2005); Unpublished: Observation.org.

#### Hoplismenus bidentatus

(Gmelin, 1790)

73E86FDF-E433-5426-B50F-53AF5EB8B931

##### Notes

Similar to *H. bispinatorius*, but it seems to be more common. This complex is one of the more problematic ones as both species are very hard to distinguish. Specimens of *H. bidentatus* are usually dark in the United Kingdom or northern European countries, but on the continent, the only notable difference is the number of flagellomeres and size (Valemberg 1975, Riedel 2021b). Therefore, all recently collected or photographed specimens were carefully checked. In the end, 87 records of 245 specimens could not be included. These records mostly include reports from Belgium and the Netherlands, but also from France, Germany and the United Kingdom. There are also reports from Italy and Norway, where neither species have been reported hibernating. Some specimens have been found in rather odd places, like fence posts, flower pots or bee hotels.

**Status**: Confirmed

**Records**: Belgium (6), Germany (2), the Netherlands (12)*, United Kingdom (6)*.

**First record**: 1985 (Hilpert (1987a), Germany).

**Hibernacula**: DT (41), O (17), U (1).

**Sources**: Hilpert (1987a), Verheyde and Quicke (2022); Unpublished: iNaturalist, iRecord, Observation.org, Pers. obs., Pers. obs. (P.-N. Libert).

**Also mentioned in**: Hilpert (1987b), Schmidt and Zmudzinski (2005).

#### Hoplismenus bispinatorius

(Thunberg, 1824)

E5FDBCBE-89FC-5826-BE7C-2F48E5647706

##### Notes

Similar to *H. bidentatus*.

**Status**: Confirmed.

**Records**: Belgium (1)*, France (4), Germany (3), Hungary (1), Russia (1), Sweden (1)*, the Netherlands (1)*.

**First record**: Ca. 2.XI.1885 (Mocsáry (1885), Hungary).

**Hibernacula**: DT (30), M (11), O (1), U (1).

**Sources**: Mocsáry (1885) [*Hoplismenus perniciosus* Grav.], Berthoumieu (1894) [*H. perniciosus* Grav.], Pic (1917) [*H. perniciosus* Grav.], Seyrig (1923) [*H. perniciosus*] and 1926, Hilpert (1987a) [*H. albifrons* Grav.], Schmidt and Zmudzinski (2005) [*H. axillatorius* Thunb.]; Unpublished: Artportalen, iNaturalist, Observation.org.

**Also mentioned in**: Constantineanu (1959), Verheyde and Quicke (2022).

#### Hoplismenus krapinensis

Hensch, 1928

AAB72952-5526-5A47-85BD-0BDEB235D8F2

##### Notes

All records consist of specimens found below bark in mountainous regions.

**Status**: Confirmed.

**Records**: Bulgaria (1), Germany (5).

**First record**: 1985-1986 (Hilpert 1987a).

**Hibernacula**: DT (54), DTCL (1).

**Sources**: Hilpert (1987a) [*Hoplismenus flavitarsis* Clém.], Tsankov and Mirchev (1999) [*Rhysaspis flavitarsis* Clem.].

**Also mentioned in**: Schmidt and Zmudzinski (2005).

#### Hoplismenus lamprolabus

Wesmael, 1857

48E02993-B272-52B9-AD2E-F34C23DB4574

##### Notes

Another species occurring in mountainous regions, all findings being made above 1000 metres. It was first reported as *Docyteles homocerus* (= now *Pseudoamblyteles homocerus*) by Seyrig (1923) in the Vosges mountain range, but this was corrected by himself in Seyrig (1926b). Lauterborn (1936) found the species in the south of Germany (Schwarzwald).

**Status**: Confirmed.

**Records**: France (1), Germany (1).

**First record**: 1915-1922 (Seyrig (1923), France).

**Hibernacula**: DT (4).

**Sources**: Seyrig (1923) [*Docyteles homocerus*] and Seyrig (1926b), Lauterborn (1936).

**Also mentioned in**: Seyrig (1926b).

#### Hoplismenus pica

Wesmael, 1855

2436C9D7-12BE-5498-B659-36C289A2148A

##### Notes

None.

**Status**: Confirmed.

**Records**: Austria (4), France (3), Germany (2), Italy (2)**, Lithuania (2), Poland (1)*, Romania (10), Russia (2).

**First record**: 18.XI.1925 (Constantineanu 1929b and Constantineanu 1959, Romania).

**Hibernacula**: DT (71), U (1).

**Sources**: Constantineanu (1959), Rasnitsyn (1959), Constantineanu (1969), Jonaitis (1982), Hinz and Kreissl (1992), Bauer (1999), Valemberg and Vago (2013), Gokhman (2014), Lungu-Constantineanu and Constantineanu (2014), Pénigot (2020); Unpublished: Facebook (Hymenopterists Forum), iNaturalist, Naturgucker.

**Also mentioned in**: Constantineanu (1929b), Rasnitsyn (1964).

#### Hoplismenus terrificus

Wesmael, 1848

51191A80-C82C-5D2F-AF2A-9174AF5ADB2A

##### Notes

The overwhelming majority of records are quite recent and mostly below bark, so this species may have become more common or was overlooked in the past.

**Status**: Confirmed.

**Records**: Belgium (16), Denmark (1)**, France (1)*, Germany (5), Hungary (1)*, Lithuania (3)*, Romania (1), Russia (6), the Netherlands (13), Ukraine (1)*.

**First record**: 1951-1958 (Rasnitsyn (1959), Russia).

**Hibernacula**: DT (146), DTCL (1), U (1).

**Sources**: Constantineanu (1959), Rasnitsyn (1959) and Rasnitsyn (1964), Hilpert (1987a), Schmidt and Zmudzinski (2005), Verheyde and Quicke (2022); Unpublished: iNaturalist, Naturbasen, Observation.org, UKRbin.

#### Ichneumon affector

Tischbein, 1879

FD3A5607-9355-574B-8A03-3FECF1496B3E

##### Notes

There are two additional uncertain records from Germany with no statement about hibernation, one from 29.XI.1986 (Schmidt and Zmudzinski 2005) and one from 17.II.2002 (GBIF).

**Status**: Confirmed.

**Records**: France (1), Germany (1), Romania (1).

**First record**: Ca. 1959 (Constantineanu (1959), Romania).

**Hibernacula**: DT (6), S (1).

**Sources**: Constantineanu (1959) [*Ichneumon steckii* Kriechb.], Bauer (1984) [*I. crassitarsis* Thoms.], Pénigot (2020).

**Also mentioned in**: Rasnitsyn (1964) [*I. stecki* Kriechb.], Schmidt and Zmudzinski (2005).

#### Ichneumon albiger

Wesmael, 1845

ACBF9402-F6FA-5569-AB92-23B4E11EB55D

##### Notes

In north-western Europe, this species belongs to a species complex consisting of species with reddish-brown hind tibia (although *I. albiger* can also have them yellow – in that case similar to *I. confusor*) and the two apical segments of the metasoma covered by a white spot. For the more common species, this complex also includes *I. extensorius* and *I. gracilentus*. The three species are difficult to distinguish and collecting specimens is necessary. Important characteristics are the number of flagellomeres, the presence of a scopa and sculpture of the hind coxae and the sculpture of the second tergite (Hilpert 1992). In total, 140 records of 370 specimens from this complex could not be included as a consequence, from a wide variety of countries including many possible first reports as hibernators. As this is a medium-sized species, both tree bark and tussocks are used as hibernacula. Sometimes high numbers of specimens in a cluster can be reached.

**Status**: Confirmed.

**Records**: Austria (3), Belarus (5), Belgium (12), Czechia (1)*, France (17), Germany (19), Ireland (3), Poland (1), Romania (10), Russia (1), Switzerland (2), United Kingdom (75).

**First record**: Ca. 1894 (Berthoumieu (1894), France).

**Hibernacula**: DT (374), DTCL (33), DV (2), LV (97), M (7), U (8).

**Sources**: Berthoumieu (1894), Meyer (1913) [*Ichneumon tempestivus* Holmgr.], Johnson (1920) [*I. tempestivus* Hlgr.], Hancock (1923) and Hancock (1925) [*I. tempestivus* Holmgr.], Hedwig (1927), Heinrich (1949a)/Heinrich (1949b), Heinrich (1951), Constantineanu (1959) [*I. tempestivus* Holmgr.], Constantineanu (1969), Valemberg and Vago (1974b) and Valemberg and Vago (1975), Valemberg (1976b) and Valemberg (1982) [some as *I. mordax* Kriech.], Bauer (1984), Gokhman (1990b), Hinz and Kreissl (1992) and Hinz and Kreissl (1993), Sebald et al. (2001), Schmidt and Zmudzinski (2005), Sebald (2007), Tschopp et al. (2012), Valemberg and Vago (2013), Lungu-Constantineanu and Constantineanu (2014) [*I. tempestivus* Holmgr.], Verheyde and Quicke (2022); Unpublished: Coll. RBINS, GBIF, iNaturalist, iRecord, Pers. obs., Pers. obs. (G. Artmann), Tereshkin (2014).

**Also mentioned in**: Tereshkin (2002), Gokhman (2003).

#### Ichneumon albiornatus

Tischbein, 1879

A969E0C8-38E8-59B2-A560-A3B4E5F349C3

##### Notes

No details exist for the two known records. Possibly more records on GBIF (notably in France and Germany) consist of hibernating specimens, but this is unclear.

**Status**: Confirmed.

**Records**: Germany (2).

**First record**: Ca. 1947-1949 (Heinrich (1949a), Germany).

**Sources**: Heinrich (1949a) [*Pterocormus variegatorius* Holmgr.], Sebald et al. (2000).

#### Ichneumon alius

Tischbein, 1879

0F7C0234-2B97-5F78-8F1E-DA88B54DA317

##### Notes

None.

**Status**: Confirmed.

**Records**: Belgium (1), Germany (2).

**First record**: Ca. 1900 (Athimus (1901a), Belgium).

**Hibernacula**: M (1), S (1), U (2).

**Sources**: Athimus (1901a) [*Ichneumon aries* Kr.], Bauer (1984); Unpublished: GBIF.

#### Ichneumon alpinator

Aubert, 1964

D20C9B83-99CC-5EEF-B3D0-52BA52FC770F

##### Notes

Confined to the Alps. This species was analysed in laboratory conditions by Hinz (1991) and obligatory diapause was demonstrated. It has not yet been found in a hibernaculum.

**Status**: Confirmed.

**Sources**: Hinz (1991) [*Ichneumon lautareti*].

#### Ichneumon altaicola

Heinrich, 1978

EE10B9CE-7302-563B-B78A-4D4D99340969

##### Notes

Sebald et al. (2000) include it, stating that the label in Bauer’s collection records the specimen as having been collected while overwintering in situ.

**Status**: Confirmed.

**Records**: Germany, 1980-2000.

**Sources**: Sebald et al. (2000).

#### Ichneumon amphibolus

Kriechbaumer, 1888

000EEFCE-6509-58CE-B36D-77728E4812D6

##### Notes

The holotype was found hibernating near Vienna, below the bark of a common pine. A variable species; the metasoma can vary from nearly entirely black to the second tergite coloured red-brown. Superficially resembles *Ichneumon inquinatus*, but the scutellum is completely black. Specimens with a black scutellum and T2(-3) darkened or black resemble darker variants of *I. boreellus* (and possibly *I. ignobilis*), which are very rare in lowlands, but microscopy is still necessary to check the sculpture of the first tergite and confirm the identification with certainty (Hilpert 1992). One specimen in Norway was found by sieving the soil. Uncertain records exist from France, Lithuania and Russia (iNaturalist).

**Status**: Confirmed.

**Records**: Austria (2), Belgium (2), Czechia (3), Germany (2), Norway (2)*, Russia (6).

**First record**: Ca. 1888 (Kriechbaumer (1888), Austria).

**Hibernacula**: DT (29), DV (1), U (4).

**Sources**: Kriechbaumer (1888), Rasnitsyn (1964), Hilpert (1987a), Hinz and Kreissl (1993), Sebald et al. (2001), Gokhman (1990a), Valemberg and Fiala (2009), Gokhman et al. (2014), Verheyde et al. (2025a); Unpublished: Observation.org, Pers. obs.

**Also mentioned in**: Gokhman (2003).

#### Ichneumon analis

Gravenhorst, 1829

4E0AC1A0-1DB9-5E06-8A82-A07A3FEF0D4F

##### Notes

Similar to *I. analisorius* (Hilpert 1992).

**Status**: Confirmed.

**Records**: France (1), Germany (1), United Kingdom (1).

**First record**: Ca. 1903 (Morley (1903), United Kingdom).

**Hibernacula**: LV (3), S (1).

**Sources**: Morley (1903), Seyrig (1923), Bauer (1984).

**Also mentioned in**: Constantineanu (1959).

#### Ichneumon analisorius

Heinrich, 1952

5DED0311-BA48-519D-9450-593500B3FA0E

##### Notes

Similar to *I. analis* (Hilpert 1992). Sebald et al. (2000) include it, stating that the label in Bauer’s collection records the specimen as having been collected while overwintering in situ.

**Status**: Confirmed.

**Records**: Germany, 1980-2000.

**Sources**: Sebald et al. (2000).

#### Ichneumon balteatus

Wesmael, 1845

A43C2989-EA9C-5AB2-AC77-E19E2A4A9348

##### Notes

In Berthoumieu (1894), this species is referred to with a cross-reference to Tischbein, which could not be found. More recently, a specimen was reported hibernating between the cracks of a living tree in Romania (Lungu-Constantineanu and Constantineanu 2014). Yet, in Timuș et al. (2014), one of the same authors presents an image identified as *I. balteatus* that appears to be a misidentification - likely depicting *Ichneumon eumerus* or a closely-related species (Hilpert 1992). Given this uncertainty and the difficulty in verifying the identification, this particular record has been excluded from consideration.

**Status**: Unverified.

**Records**: Romania, 2006-2010.

**Hibernacula**: LT (1).

**Sources**: Lungu-Constantineanu and Constantineanu (2014).

**Also mentioned in**: Berthoumieu (1894), Constantineanu (1959), Rasnitsyn (1964).

#### Ichneumon bellipes

Wesmael, 1845

BF78E8CD-2F2F-586D-8E38-EE9F6D207EAC

##### Notes

Discussed in detail by Athimus (1901a), several specimens were found in moss.

**Status**: Confirmed.

**Records**: Belgium (2), Germany (1).

**First record**: Ca. 1900 (Athimus (1901a), Belgium).

**Sources**: Athimus (1901a) [incl. *Ichneumon medialis*], Sebald et al. (2000).

**Hibernacula**: M (40), U (1).

#### Ichneumon bucculentus

Wesmael, 1845

DED6852E-6B30-5F85-86FB-46003DC66213

##### Notes

In north-western Europe, this is by far the most common species with red-brown hind tibia and the three apical segments covered with a white spot. However, there are many similar (rare) species which could be difficult to distinguish from pictures (Hilpert 1992). Therefore, only clear data were included. In total, 48 records of 69 specimens could not be integrated, most of them being from Belgium. These records would not yield any new reports for certain countries. *I. bucculentus* is specialised in hibernation on dead wood, although several specimens have been found in tussocks and one specimen in Greece was discovered inside the cracks of a marble quarry.

**Status**: Confirmed.

**Records**: Austria (5), Belarus (2)**, Belgium (145), Bulgaria (3), Czechia (12)*, Denmark (4)**, France (30), Germany (24), Grand Duchy of Luxembourg (1)**, Greece (1)*, Hungary (4)*, Lithuania (1)*, Poland (4)*, Romania (11), Russia (9), Switzerland (2)*, the Netherlands (31), Ukraine (20)*, United Kingdom (12)*.

**First record**: Ca. 1871 (Tischbein (1871), Germany).

**Hibernacula**: C (1), DT (534), DTCL (14), LV (4), M (8), U (28).

**Sources**: Tischbein (1871), Berthoumieu (1894), Pic (1919), Seyrig (1923) and Seyrig (1926a), Constantineanu (1929b) and Constantineanu (1959), Aerts (1957), Rasnitsyn (1959), Constantineanu (1969), Valemberg (1982), Bauer (1984), Hilpert (1989), Kolarov (1992), Gokhman (1993), Hinz and Kreissl (1993), Tsankov and Mirchev (1999), Sebald et al. (2001), Schmidt and Zmudzinski (2005), Valemberg and Vago (2013), Lungu-Constantineanu and Constantineanu (2014), Verheyde and Quicke (2022); Unpublished: Coll. RBINS, GBIF, iNaturalist, Insecte.org, iRecord, Naturgucker, Observation.org, Pers. obs., Pers. obs. (G. Artmann).

**Also mentioned in**: Tischbein (1873b), Morley (1903), Rasnitsyn (1964), Gokhman (2003).

#### Ichneumon buryas

Heinrich, 1949

05F4A84E-C3FD-5C8B-991A-9A9E99740701

##### Notes

The holotype of this species was found hibernating near Berchtesgaden.

**Status**: Confirmed.

**Records**: Germany, 5.V.1947.

**Sources**: Heinrich (1949b).

#### Ichneumon caedator

Gravenhorst, 1829

E3A7EE96-2D1A-5B6C-9E98-F25A7588339F

##### Notes

Reported by Rasnitsyn (1964) from the Moscow region. Recently confirmed by Varga et al. (2020).

**Status**: Confirmed.

**Records**: Russia (1), Ukraine (1).

**First record**: 1959-1964 (Rasnitsyn (1964), Russia).

**Hibernacula**: DT (1), U (1).

**Sources**: Rasnitsyn (1964), Varga et al. (2020).

#### Ichneumon caloscelis

Wesmael, 1845

071895A3-010A-5F3D-8EA5-D212469E28D2

##### Notes

None.

**Status**: Confirmed.

**Records**: Belgium (1)*, France (2), Germany (5).

**First record**: Ca. 1863 (Tischbein (1863), Germany).

**Hibernacula**: DT (2), M (4), S (1), U (3).

**Sources**: Tischbein (1863) and Tischbein (1871), Berthoumieu (1894), Seyrig (1923), Heinrich 1949a[*Pterocormus caloscelis* Wesm.], Bauer (1984); Unpublished: Coll. RBINS, GBIF.

**Also mentioned in**: Tischbein (1873b), Morley (1903), Constantineanu (1959).

#### Ichneumon capriolus

Hilpert, 1992

6994021A-3617-575C-B6AE-67FEBF545B75

##### Notes

Sebald et al. (2000) include it, based on their own findings and an older specimen from Bauer, so at least two unique records exist. The specimen from Bauer likely refers to the third paratype, collected on 10.XI.1989 near Kallmünz (Hilpert 1992).

**Status**: Confirmed.

**Records**: Germany, 1980-2000 (2 records).

**Sources**: Sebald et al. (2000).

#### Ichneumon cerebrosus

Wesmael, 1859

00CCEA7B-2630-5BC2-965E-BCA8E1E80B5C

##### Notes

None.

**Status**: Confirmed.

**Records**: Germany, ca. 1984.

**Hibernacula**: S (1).

**Sources**: Bauer (1984).

#### Ichneumon cessator

Muller, 1776

E2EB6DDF-770A-56B4-8F7A-EC800CF6D08B

##### Notes

On pictures of lower quality, it could be confused with similarly-coloured species, for example, *Hoplismenus terrificus* or *Thyrateles camelinus*. Females of *I. cessator* can be recognised by their rather conical habitus (similar to *H. terrificus*), some orange-red flagellomeres (not white) and especially the two small white spots at the apical segments of the metasoma.

**Status**: Confirmed.

**Records**: Belgium (1)*, Czechia (1)*, France (1), Germany (6), Russia (1), Sweden (1)*, Ukraine (1)*.

**First record**: 1915-1922 (Seyrig (1923), France).

**Hibernacula**: DT (9), DTCL (15), M (2), S (1).

**Sources**: Seyrig (1923), Bauer (1984), Hilpert (1987a); Unpublished: Pers. obs.

**Also mentioned in**: Bauer (1999), Rasnitsyn (1964).

#### Ichneumon cinxiae

Kriechbaumer, 1890

EE9BA1C5-9B5B-5B8F-BFC9-D212CCE5D2F7

##### Notes

Found also near Berchtesgaden, just like *I. buryas*. The holotype of the junior synonym *I. tenuicornutus* was probably also found hibernating in May 1947 (Hilpert 1992).

**Status**: Confirmed.

**Records**: Germany (2).

**First record**: Ca. 1947-1949 (Heinrich 1949b, Germany).

**Sources**: Heinrich (1949b) [*Ichneumon tenuicornutus*], Sebald et al. (2000).

#### Ichneumon computatorius

Muller, 1776

887A8DD3-96DC-5BED-9249-16DB139DEF40

##### Notes

Similar to *I. inquinatus* and *I. magistratus*, but the scutellum is white and the apical flagellomeres are strongly widened behind the white antennal ring (Hilpert 1992). The records of Constantineanu (1929b) and Constantineanu (1959) possibly concern the same specimens.

**Status**: Confirmed.

**Records**: Austria (11), Czechia (1)*, France (3), Germany (8), Romania (6), Russia (2).

**First record**: Ca. 1863 (Tischbein (1863), Germany).

**Hibernacula**: C (1), DT (33), DTCL (3), M (2), U (4).

**Sources**: Tischbein (1863) and Tischbein (1876), Pic (1917), Constantineanu (1929b), Heinrich (1949b), Aerts (1957), Constantineanu (1959), Rasnitsyn (1964), Weber (1989), Gokhman (1990a) [*Ichneumon croceipes* Wesm.], Hinz and Kreissl (1992) and Hinz and Kreissl (1993), Schmidt and Zmudzinski (2005), Valemberg and Vago (2013); Unpublished: GBIF, Pers. obs.

**Also mentioned in**: Tischbein (1871) and Tischbein (1873b), Berthoumieu (1894), Morley (1903), Gokhman (2003).

#### Ichneumon confusor

Gravenhorst, 1820

F7795936-4F15-5701-9BD4-46F8BD902763

##### Notes

Similar to *I. albiger* (more specifically the form with yellow tibia), *I. melanotis* and red forms of *I. molitorius*. It is difficult to identify *I. confusor* from pictures, but the pre-apical flagellomeres are strongly transverse and the scopa is clearly present (Hilpert 1992). Some additional, but uncertain reports in the winter months exist for Austria, Belgium and Sweden.

**Status**: Confirmed.

**Records**: Austria (9), Belarus (4), Belgium (6), Czechia (1)*, France (27), Germany (17), Norway (1)*, Poland (2), Romania (6), Russia (3), Sweden (1), Switzerland (1)*, the Netherlands (2), Ukraine (2), United Kingdom (33).

**First record**: 10.III.1835 (Tischbein (1873b), Germany).

**Hibernacula**: DT (253), DTCL (42), LV (43), M (10), U (41).

**Sources**: Shuckard (1838) [*Ichneumon confusorius*], Holmgren (1864) [*I. confusorius* Grav.], Tischbein (1873b) [*I. crassicornis* Tischb.; *I. confusorius* Gr.], Berthoumieu (1894) [*I. confusorius* Grav.], Morley (1903) [*I. confusorius* Grav.], Pic (1916), Pic (1917) and Pic (1919) [*I. confusorius* Grav.], Smits van Burgst (1918) [*I. confusorius* Grv.], Hancock (1923) [*I. confusorius* Grav.], Seyrig (1923) and Seyrig (1926a) [*I. confusorius* Gr.], Hedwig (1927) [*I. confusorius* Grv.], Constantineanu (1929b) [*I. confusorius* Grav.], Heinrich (1935), Heinrich (1949a) and Heinrich (1951) [*Pterocormus confusorius*; *I. confusorius* Grav.], Renken (1956) [*I. confusorius* Grav.], Constantineanu (1959) [*I. confusorius* Grav.], Rasnitsyn (1959) and Rasnitsyn (1964) [*I. confusorius* Grav.], Constantineanu (1969) [*I. confusorius* Grav.], Valemberg and Vago (1974a)/Valemberg and Vago (1974b) and Valemberg and Vago (1975) [*I. confusorius* Grav.], Valemberg (1976b), Valemberg (1978) and Valemberg (1982), Bauer (1984), Gokhman (1985), Hinz and Kreissl (1992) and Hinz and Kreissl (1993), Sebald et al. (2001), Schmidt and Zmudzinski (2005), Valemberg and Vago (2013), Varga et al. (2020), Verheyde and Quicke (2022); Unpublished: Coll. RBINS, Observation.org, Pers. obs., Pers. obs. (G. Artmann), UKRbin, Tereshkin (2014).

**Also mentioned in**: Tischbein (1863) and Tischbein (1871) [*Ichneumon confusorius* Gr.], Tereshkin (2002), Gokhman (2003).

#### Ichneumon coniger

Tischbein, 1876

04AB8A09-124F-5054-999B-270D64CBEB20

##### Notes

This species is mainly black with orange legs, has a white scutellum and the two apical segments are covered with white spots. More importantly, it has a tubercle on the hind coxa. The distribution seems to be slightly more eastern. The holotype of *I. pentaleucus*, described by Kriechbaumer (1895) and later synonymised (Hilpert 1992), was found hibernating below moss in Germany. One uncertain hibernation record for Austria (GBIF).

**Status**: Confirmed.

**Records**: Bulgaria (2), Czechia (3), Germany (1), Hungary (1)*, Romania (4), Russia (2), Ukraine (1)*.

**First record**: March 1894 (Kriechbaumer (1895), Germany).

**Hibernacula**: DT (24), M (1), U (3).

**Sources**: Kriechbaumer (1895) [*Ichneumon pentaleucus*], Constantineanu (1959), Rasnitsyn (1964), Constantineanu (1969), Kolarov (1992), Valemberg and Fiala (2009), Lungu-Constantineanu and Constantineanu (2014); Unpublished: GBIF, iNaturalist, Pers. obs.

#### Ichneumon crassifemur

Thomson, 1886

AC3E5BA8-74DC-525E-AEE8-CDDECA14C3D1

##### Notes

Similar to *I. melanotis* and *I. molitorius* when the second and third tergites are reddish, but the sculpture on the femora is distinctive (Hilpert 1992). Uncertain records exist for Italy (iNaturalist) and Latvia (Dabasdati).

**Status**: Confirmed.

**Records**: Belarus (1), Belgium (4), Bulgaria (1), Czechia (7)*, France (21), Germany (5), Russia (3), United Kingdom (1).

**First record**: 13.III.1898 (Morley (1903), United Kingdom).

**Hibernacula**: DT (85), DTCL (1), U (9).

**Sources**: Morley (1903), Pic (1917), Valemberg and Vago (1974a), Valemberg (1975), Valemberg (1978) and Valemberg (1982), Bauer (1984), Gokhman (1985), Hilpert (1989), Kolarov (1992), Sebald et al. (2001), Schmidt and Zmudzinski (2005), Valemberg and Vago (2013), Verheyde and Quicke (2022); Unpublished: Coll. RBINS, Observation.org, Tereshkin (2014).

**Also mentioned in**: Tereshkin (2002), Gokhman (2003).

#### Ichneumon crassigena

Kriechbaumer, 1890

B49FD9AA-750E-5B23-9A86-7FC92EBBAAC1

##### Notes

This species has a complex taxonomic history, see also *Ichneumon haemorrhoicus. I. albicollis* Wesmael, 1857 is considered a synonym [as a junior homonym of *Ichneumon albicollis* Schiodte, 1839] (Yu et al. 2016).

**Status**: Confirmed.

**Records**: Romania, 1929-1959 (Constantineanu 1959).

**Hibernacula**: DT (1).

**Sources**: Constantineanu (1959).

**Also mentioned in**: Morley (1903).

#### Ichneumon decurtatus

Wesmael, 1845

ED6F3174-78DC-5147-8299-3053AE4CAA1B

##### Notes

None.

**Status**: Confirmed.

**Records**: Germany (1), Romania (4).

**First record**: Ca. 1863 - February (Tischbein (1863), Germany)

**Hibernacula**: DT (5), M (2).

**Sources**: Tischbein (1863), Constantineanu (1956) and Constantineanu (1969).

**Also mentioned in**: Tischbein (1871), Constantineanu (1959).

#### Ichneumon deliratorius

Linnaeus, 1758

570B01A8-EF14-53EA-8F36-3C85A85179C2

##### Notes

Hinz (1983) argues this species hibernates in the host cocoon as a pharate adult, which renders the records from Romania unlikely. From Sweden (Artportalen) and the United Kingdom (iRecord), there are two incorrect records referring to individuals of the *Ichneumon molitorius*/*melanotis*-complex.

**Status**: Unverified.

**Records**: Romania (2).

**First record**: 1964-1967 (Constantineanu (1969), Romania).

**Hibernacula**: DT (2).

**Sources**: Constantineanu (1969), Lungu-Constantineanu and Constantineanu (2014).

**Also mentioned in**: Hinz (1983).

#### Ichneumon didymus

Gravenhorst, 1829

A6867C12-3F30-5C8A-BDAF-436DEAE0A8AF

##### Notes

None.

**Status**: Confirmed.

**Records**: France (2), Germany (4), Russia (1).

**First record**: Ca. 1913 - December (Pfeffer (1913), Germany).

**Hibernacula**: DT (5), DTCL (1), M (11), U (1).

**Sources**: Pfeffer (1913), Seyrig (1923), Rasnitsyn (1959), Valemberg (1976a), Hinz (1981), Bauer (1984); Unpublished: GBIF.

#### Ichneumon dilleri

Heinrich, 1980

B4089518-B8D9-5D33-A5AA-CF7B7EC67E0A

##### Notes

None.

**Status**: Confirmed.

**Records**: Germany (3).

**First record**: 12.V.1947 (Heinrich (1949b), Germany).

**Hibernacula**: S (1), U (2).

**Sources**: Heinrich (1949b) [*Ichneumon sulcatus* Berth.], Bauer (1984), Sebald et al. (2000).

#### Ichneumon diversor

Wesmael, 1855

1574B29E-242F-5383-91F4-948D730BEE73

##### Notes

None.

**Status**: Confirmed.

**Records**: Austria (1), Germany (2).

**First record**: Ca. 1984 (Bauer (1984), Germany).

**Hibernacula**: DT (1), DTCL (1), U (1).

**Sources**: Bauer (1984), Hinz and Kreissl (1992), Sebald et al. (2000).

#### Ichneumon dolosus

Wesmael, 1855

A0043B23-D157-5271-AF28-0630EE101F4B

##### Notes

While the hibernation records are clear, the holotype of this species is probably lost and the taxonomic status is thus unclear. Very similar to *I. ignobilis* and *I. stigmatorius*. The species is not included in Hilpert (1992).

**Status**: Taxon requiring further verification.

**Records**: France (1), Germany (1).

**First record**: Ca. 1873 (Tischbein (1873b), Germany).

**Hibernacula**: M (4).

**Sources**: Tischbein (1873b) [*Ichneumon perhiematus* Tischb.], Seyrig (1923) [I. stigmatorius var. dolosus Wsm.].

**Also mentioned in**: Sebald et al. (2000).

#### Ichneumon emancipatus

Wesmael, 1845

A41BAA16-9EBE-5789-B56B-DA6843DB88C3

##### Notes

Similar to *I. gracilicornis*, identification from pictures in the field is hardly possible. The main differences lie in the presence/absence of the scopa and the width of the middle flagellomeres (Hilpert 1992). In specimens from Belgium and the Netherlands, it appears that the proximal flagellomeres tend to be orange in *I. gracilicornis* and black in *I. emancipatus*. However, more research is necessary to see if this is really indicative. About 10 specimens could not be identified to species level. Records from the complex contain new hibernation records for Italy, Lithuania and the Netherlands.

**Status**: Confirmed.

**Records**: Belgium (2), France (2), Germany (3).

**First record**: Ca. 1894 (Berthoumieu (1894), France).

**Hibernacula**: DT (1), M (3), S (1), U (3).

**Sources**: Berthoumieu (1894), Athimus (1901a), Seyrig (1926a), Heinrich (1949b), Aerts (1957), Constantineanu (1959), Bauer (1984); Unpublished: Coll. RBINS.

**Also mentioned in**: Morley (1903), Constantineanu (1959).

#### Ichneumon eremitatorius

Zetterstedt, 1838

CD76FF3C-C0C7-5940-B29B-40182B53FF5C

##### Notes

The sole finding was found hibernating in May, at a height of 1200 metres in the Alps (Heinrich 1949b). Heinrich considers his own identification as doubtful.

**Status**: Unverified.

**Records**: Germany, V.1947-1948.

**Sources**: Heinrich (1949b).

**Also mentioned in**: Constantineanu (1959).

#### Ichneumon erythromerus

Wesmael, 1857

D0E5E225-7FF4-5948-A4F2-06FFE9A7604B

##### Notes

Similar to *I. insidiosus* (Horstmann 2003).

**Status**: Confirmed.

**Records**: Germany (2).

**First record**: Ca. 1879 (Tischbein (1879), Germany).

**Sources**: Tischbein (1879) [*Ichneumon examinator* Tischb.], Sebald et al. (2000).

#### Ichneumon eumerus

Wesmael, 1857

40BB273C-1714-53FA-8C23-CE5436D03592

##### Notes

Similar to *I. exilicornis* (Hilpert 1992). Sebald et al. (2000) include it, stating that the label in Bauer’s collection records the specimen as having been collected while overwintering in situ.

**Status**: Confirmed.

**Records**: Germany, 1980-2000.

**Sources**: Sebald et al. (2000).

#### Ichneumon exilicornis

Wesmael, 1857

C1FB2BD5-DC18-57C5-9CAC-F3A647803120

##### Notes

Similar to *I. eumerus* (Hilpert 1992) There is one additional uncertain record for Germany (GBIF). An alpine species was split off by Bauer (1999); *I. centralpinicola* Bauer, 1999. Both records we have, however, are not alpine and therefore refer to *I. exilicornis* sensu stricto.

**Status**: Confirmed.

**Records**: France (1), Germany (1)*.

**First record**: 20.II.1977 (Valemberg (1976b), France).

**Hibernacula**: LV (1), U (1).

**Sources**: Valemberg (1976b) [*Ichneumon caproni* Pk.]; Unpublished: GBIF.

#### Ichneumon extensorius

Linnaeus, 1758

75A2FF5A-E7DE-51FA-BE1B-1ED74EC332AA

##### Notes

Similar to *I. albiger* and *I. gracilentus*. This species tends to be slightly more uncommon. Exceptionally, it has also been observed in caves (Seyrig 1926b). Kolarov (1992) mentions 242 individuals below the bark of an oak, one of the highest records of clustered ichneumonids ever reported. It is also one of the first ichneumonids ever to be reported as a hibernating species, also below bark: “Mares plures (..) autumno, socialiter sub cortice quercus latentes, (...)” (Gravenhorst 1829).

**Status**: Confirmed.

**Records**: Austria (1), Belarus (3), Belgium (5), Bulgaria (1), Czechia (2)*, Denmark (7)**, France (26), Germany (19), Ireland (4), Norway (3)*, Poland (2), Romania (8), Russia (4), Sweden (4)*, Switzerland (3), the Netherlands (1), United Kingdom (27).

**First record**: Ca. 1829 (Gravenhorst (1829), Germany).

**Hibernacula**: C (2), DT (849), DTCL (56), DV (6), LV (13), M (22), S (6), U (13).

**Sources**: Gravenhorst (1829), Tischbein (1863) and Tischbein (1876) [*Ichneumon retractus* Tischb.], Berthoumieu (1894), Kriechbaumer (1896a), Morley (1903), Meyer (1913), Pfeffer (1913), Pic (1917) and Pic (1919), Seyrig (1923) and Seyrig (1926b), Hancock (1925), Pax and Maschke (1935), Heinrich (1949a)/Heinrich (1949b) and Heinrich (1951), Aerts (1957), Valemberg and Vago (1974a), Valemberg (1975), Valemberg (1976a), Valemberg (1978) and Valemberg (1982), Bauer (1984), Kolarov (1992), Gokhman (1993), Hinz and Kreissl (1993), Sebald et al. (2001), Schmidt and Zmudzinski (2005), Sebald (2007), Tschopp et al. (2012), Valemberg and Vago (2013), Verheyde and Quicke (2022); Unpublished: Artsobservasjoner, Artportalen, Coll. RBINS, iNaturalist, Insecte.org, iRecord, Naturbasen, Pers. obs., Pers. obs. (G. Artmann; P.-N. Libert), Tereshkin (2014).

**Also mentioned in**: Kriechbaumer (1896b), Tereshkin (2002), Gokhman (2003), Kocot-Zalewska and Domagała (2020).

#### Ichneumon factor

Dalla Torre, 1901

9277D2F5-93B4-5464-9349-FF69D564A398

##### Notes

The sole finding was found hibernating in May, at a height of 1200 metres in the Alps (Heinrich 1949b).

**Status**: Confirmed.

**Records**: Germany, V.1947-1948.

**Sources**: Heinrich (1949b).

#### Ichneumon filatus

(Tischbein, 1879)

EF56390A-39A8-5997-B39D-2F81D6E319AE

##### Notes

More common in eastern Europe. It was originally described from Ukraine (Tischbein 1879). Rasnitsyn reports having found three specimens hibernating (Rasnitsyn 1959).

**Status**: Confirmed.

**Records**: Russia, 1951-1958.

**Sources**: Rasnitsyn (1959) [*Amblyteles filatus* Tischb.].

#### Ichneumon formosus

Gravenhorst, 1829

F8BAB534-DD4D-518D-88CE-DC2C6BC62B1B

##### Notes

Similar to *I. haglundi* and *I. hinzi*, which are mostly alpine. The metasoma can be reddish in some specimens.

**Status**: Confirmed.

**Records**: Belarus (1), Belgium (4), Finland (1), France (4), Germany (7), Romania (1), Russia (4), United Kingdom (1).

**First record**: Ca. 1871 (Tischbein (1871), Germany).

**Hibernacula**: DT (17), , DV (1), M (8), U (6).

**Sources**: Tischbein (1871) and Tischbein (1873b) [*Ichneumon Mäklini* Holmg.; *I. obsessor* W.], Berthoumieu (1894) [*I. Maklini* Holm.], Athimus (1901a) [*I. Maklini* Holm.], Seyrig (1923) [*I. obsessor* Wsm.], Maneval (1925) [*I. Maklini* Hlm.], Seyrig (1926a) [*I. Maklini* Hlm.], Constantineanu (1929b) [*I. obsessor* Wesm.], Heinrich (1949a) [*Pterocormus obessor* Wesm.] and Heinrich (1951) [*I. obsessor* Wesm.], Rasnitsyn (1959) [*I. batis* Holmgr.; *I. mäklini* Holm.], Bauer (1961), Rasnitsyn (1964) [*I. mäklini* Holm.], Bauer (1984), Gokhman (1990b), Hinz and Horstmann (1998) [forma microcephalus Steph.], Sebald et al. (2001); Unpublished: Coll. RBINS, Observation.org, Tereshkin (2014).

**Also mentioned in**: Morley (1903), Constantineanu (1959) [*I. obsessor* Wesm.], Tereshkin (2002), Gokhman (2003).

#### Ichneumon freyi

Kriechbaumer, 1880

09E69B03-0CC9-5BAA-91BD-4EB603760F9A

##### Notes

In a paper on the life cycles of some ichneumonids in the Alps, Hinz (1991) mentions this species as hibernating. Although this is very likely, there are no field data known.

**Status**: Unverified.

**Sources**: Hinz (1991).

#### Ichneumon fulvicornis

Gravenhorst, 1829

17E43635-681C-5500-B048-2FB24680E1B9

##### Notes

Sebald et al. (2000) include it, stating that the label in Bauer’s collection records the specimen as having been collected while overwintering in situ.

**Status**: Confirmed.

**Records**: Germany, 1980-2000.

**Sources**: Sebald et al. (2000).

#### Ichneumon fuscatus

Gmelin, 1790

FD3D3D7F-DE88-5782-89E6-853D0A979EF2

##### Notes

Sebald et al. (2000) include it, stating that the label in Bauer’s collection records the specimen as having been collected while overwintering in situ. It is missing from Hilpert (1992) and no females are preserved (Horstmann 2003), so the taxonomic status remains unclear.

**Status**: Taxon requiring further verification.

**Records**: Germany, 1980-2000.

**Sources**: Sebald et al. (2000).

#### Ichneumon gracilentus

Wesmael, 1845

08EF9251-40C1-579F-96E6-3C9D675793BD

##### Notes

Similar to *I. albiger* and *I. extensorius*. From the records unassigned to species, the only potential new country record would be for Finland (iNaturalist). The holotype of *I. Wüstneii*, described by Kriechbaumer (1890) and later synonymised (Hilpert 1992), was found hibernating below moss in Denmark.

**Status**: Confirmed.

**Records**: Austria (17), Belarus (13), Belgium (21), Czechia (2)*, Denmark (5), France (19), Germany (17), Ireland (1), Latvia (1), Norway (15)*, Poland (1), Romania (4), Russia (6), Sweden (1)*, the Netherlands (4), Ukraine (7), United Kingdom (31)*.

**First record**: Ca. 1863 (Tischbein (1863), Germany).

**Hibernacula**: DT (555), DTCL (56), LT (1), LV (2), M (19), U (26).

**Sources**: Tischbein (1863) and Tischbein (1876), Kriechbaumer (1890) [*Ichneumon Wüstneii*], Kriechbaumer (1896b), Pfeffer (1913), Pic (1917), Johnson (1920), Seyrig (1923) and Seyrig (1926a), Heinrich (1926), Ozols (1941), Heinrich (1949a)/Heinrich (1949b) and Heinrich (1951), Constantineanu (1959), Rasnitsyn (1959) and Rasnitsyn (1964), Constantineanu (1969), Valemberg (1982), Gokhman (1985), Hilpert (1989), Hinz and Kreissl (1992) and Hinz and Kreissl (1993), Gokhman 1993, Sebald et al. (2001), Schmidt and Zmudzinski (2005), Valemberg and Vago (2013), Varga et al. (2020), Verheyde and Quicke (2022); Unpublished: Artportalen, Artsobservasjoner, Coll. RBINS, Facebook (Årevinger i Norge), iNaturalist, Naturbasen, Observation.org, Pers. obs., Pers. obs. (H. Haraldseide), Tereshkin (2014).

**Also mentioned in**: Tischbein (1871) and Tischbein (1873b), Berthoumieu (1894) [incl. *I. Wustnei*], Morley (1903), Tereshkin (2002), Gokhman (2003).

#### Ichneumon gracilicornis

Gravenhorst, 1829

8C8138E8-67C4-59FA-971C-4B7A4C56D5F9

##### Notes

Similar to *I. emancipatus*.

**Status**: Confirmed.

**Records**: Belgium (2), France (6), Germany (10), Poland (1), Romania (4), Russia (2).

**First record**: Ca. 1863 (Tischbein (1863), Germany).

**Hibernacula**: C (1), DT (12), DV (1), M (17), S (1), U (8).

**Sources**: Tischbein (1863) and Tischbein (1873b), Berthoumieu (1894), Athimus (1901a), Pic (1917), Seyrig (1923) and Seyrig (1926b), Constantineanu (1929b), Pax and Maschke (1935), Heinrich (1949a)/Heinrich (1949b) [*Pterocormus gracilicornis* Grav.], Heinrich (1951), Constantineanu (1959), Rasnitsyn (1959), Bauer (1984), Gokhman (1990b), Bauer (1999); Unpublished: Coll. RBINS, GBIF.

**Also mentioned in**: Tischbein (1871), Morley (1903), Rasnitsyn (1964), Gokhman (2003), Kocot-Zalewska and Domagała (2020).

#### Ichneumon gratus

Wesmael, 1855

13625B30-260D-5D39-815C-DA2AC97D29E4

##### Notes

None.

**Status**: Confirmed.

**Records**: Czechia (1)*, Romania (1).

**First record**: 28.XI.1926 (Constantineanu (1929a), Romania).

**Hibernacula**: DT (3).

**Sources**: Constantineanu (1929a) [*Ichneumon Andrei* Berth.]; Unpublished: Pers. obs.

**Also mentioned in**: Constantineanu (1959) [*Ichneumon andrei* Berth.].

#### Ichneumon haemorrhoicus

Kriechbaumer, 1887

79A8E914-9CFA-5759-88AB-FD6F7AA52E38

##### Notes

This species has a complex taxonomic history, see *Ichneumon crassigena* (Yu et al. 2016).

**Status**: Confirmed.

**Records**: Bulgaria (1), France (1).

**First record**: 1977-1978 (France, Valemberg and Bosquit (1979)).

**Hibernacula**: DT (2).

**Sources**: Valemberg and Bosquit (1979), Kolarov (1992) [*I. corsiflavator* Aub.].

#### Ichneumon haglundi

Holmgren, 1864

5E84D063-838C-5004-A5A9-B5E1095A0D32

##### Notes

Similar to the more common *I. formosus* and *I. hinzi*, the latter species being alpine as well. The context of this finding is unclear. It is mentioned as hibernating in Constantineanu (1959), but no original records are included.

**Status**: Unverified.

**Records**: Romania, 1929-1959.

**Sources**: Constantineanu (1959).

#### Ichneumon hinzi

Heinrich, 1972

1F6C15C9-6808-5AB3-96E5-7C3D4AA3F58C

##### Notes

Similar to *I. formosus* and *I. haglundi* (Hilpert 1992). Sebald et al. (2000) include it, stating that the label in Bauer’s collection records the specimen as having been collected while overwintering in situ.

**Status**: Confirmed.

**Records**: Germany, 1980-2000.

**Sources**: Sebald et al. (2000).

#### Ichneumon ignobilis

Wesmael, 1855

E627B336-AB15-5109-A378-E8E0CE1F8F18

##### Notes

Very similar to *I. dolosus* and *I. stigmatorius*. Another species associated with mountainous regions; besides the Alps, also abundant in Scandinavia (GBIF, Hilpert (1992)). Some uncertain additional records exist from Germany (GBIF).

**Status**: Confirmed.

**Records**: France (1), Germany (2).

**First record**: 1915-1922 (France, Seyrig (1923)).

**Hibernacula**: M (1), S (1), U (1).

**Sources**: Seyrig (1923), Bauer (1984), Sebald et al. (2000).

#### Ichneumon ingratus

(Hellen, 1951)

F9518C5F-3CD7-561B-9D2F-F5C567EA22BB

##### Notes

None.

**Status**: Confirmed.

**Records**: Belarus (1), Germany (2), Russia (1).

**First record**: 22.IV.1984 (Belarus, Tereshkin (2014)).

**Hibernacula**: DT (8), U (1).

**Sources**: Hilpert (1987a), Gokhman (1990a), Sebald et al. (2000); Unpublished: Tereshkin (2014).

**Also mentioned in**: Tereshkin (2002), Gokhman (2003).

#### Ichneumon inops

Holmgren, 1880

C6FA13B6-3E06-5704-9012-8DCE26F64C42

##### Notes

The records mentioned in Constantineanu (1929b) and Constantineanu (1959) could concern the same individuals, but at least two records from the latter paper seem to be original.

**Status**: Confirmed.

**Records**: Romania (6).

**First record**: 8.XI.1926 (Romania, Constantineanu (1929b)).

**Hibernacula**: DT (6).

**Sources**: Constantineanu (1929b), Constantineanu (1959).

#### Ichneumon inquinatus

Wesmael, 1845

5B792368-6FA3-5221-A1A0-5CBD9143B03B

##### Notes

Often found in large aggregations below bark. Valemberg and Vago (2013) mention 287 individuals on a dead tree trunk. Some of the additional sources in Rasnitsyn (1964) were erroneously included.

**Status**: Confirmed.

**Records**: Austria (1), Belgium (125), Czechia (8)*, Denmark (1)**, France (45), Germany (19), Hungary (5), Italy (1)**, Poland (1)*, Romania (5), Russia (13), Switzerland (1)*, the Netherlands (11), Ukraine (6), United Kingdom (2)*.

**First record**: Ca. 1871 (Tischbein (1871), Germany).

**Hibernacula**: DT (1824), DTCL (33), DV (2), LV (7), M (23), U (31).

**Sources**: Tischbein (1871) and Tischbein (1876), Mocsáry (1885), Berthoumieu (1894), Athimus (1901a)/Athimus (1901b) [incl. *Ichneumon brevigena* Thoms.], Seyrig (1923) and Seyrig (1926a)/Seyrig (1926b), Györfi (1948), Leclercq (1953), Aubert (1960), Rasnitsyn (1959), Constantineanu (1959) [incl. *I. brevigena* Thoms.] and Constantineanu (1969), Györfi (1963), Valemberg (1975), Valemberg (1976b), Valemberg (1978) and Valemberg (1982), Bauer (1984), Hilpert (1989), Hinz and Kreissl (1992), Gokhman (1993), Sebald et al. (2001), Schmidt and Zmudzinski (2005), Valemberg and Vago (2013), Lungu-Constantineanu and Constantineanu (2014), Pénigot (2020), Varga et al. (2020), Verheyde and Quicke (2022); Unpublished: Coll. RBINS, iNaturalist, Insecte.org, Naturgucker, Observation.org, Pers. obs., Pers. obs. (G. Artmann).

**Also mentioned in**: Rasnitsyn (1964), Gokhman (2003).

#### Ichneumon insidiosus

Wesmael, 1845

85187274-6926-50B9-8E70-84C6A32F09D5

##### Notes

Similar in apperance to *I. erythromerus* (Horstmann 2003).

**Status**: Confirmed.

**Records**: France (4), Germany (7), Ireland (2), Russia (2), United Kingdom (1)*.

**First record**: Ca. 1871 (Tischbein (1871), Germany).

**Hibernacula**: DT (8), DV (1), LV (1), M (7), S (1), U (2).

**Sources**: Tischbein (1871) and Tischbein (1873b), Berthoumieu (1894), Seyrig (1923), Heinrich (1951), Aerts (1957), Rasnitsyn (1959), Bauer (1984), Gokhman (1990b), Sebald et al. (2001), Valemberg and Vago (2013); Unpublished: GBIF, Pers. obs.

**Also mentioned in**: Morley (1903), Constantineanu (1959), Gokhman (2003).

#### Ichneumon languidus

Wesmael, 1845

5A309478-244A-5B01-BB13-8ED5D5B4B576

##### Notes

In older literature, not to be confused with *Ichneumon pistorius* (var. luteoannulatus), which has been synonymised with *Stenichneumon militarius*. Specimens with a red metasoma (var. immisericors) can be confused with several other species (Hilpert 1992).

**Status**: Confirmed.

**Records**: Belarus (1), Belgium (1)*, France (8), Germany (2), Romania (12), the Netherlands (1).

**First record**: Ca. 1926 (Lauterborn (1926), Germany).

**Hibernacula**: DT (37), M (1), U (2).

**Sources**: Lauterborn (1926), Seyrig (1926b), Heinrich (1949a) [*Pterocormus languidus* Wesm.], Constantineanu (1959) and Constantineanu (1969), Valemberg (1976b) and Valemberg (1982) [incl. *Ichneumon immisericors*], Valemberg and Bosquit (1979), Valemberg and Vago (2013), Lungu-Constantineanu and Constantineanu (2014), Pénigot (2020), Verheyde et al. (2025a); Unpublished: Pers. obs., Pers. obs. (P.-N. Libert), Tereshkin (2014).

**Also mentioned in**: Hinz and Horstmann (1998), Tereshkin (2002).

#### Ichneumon lautatorius

Desvignes, 1856

40F05B8B-ED18-54E6-93EC-B271DCD98B11

##### Notes

Similar to *I. sarcitorius*, equally specialised in grass tussocks and vegetation as hibernaculum, but with two white bands on the metasoma. In rather low abundance present throughout Europe, but with regular observations in Belgium, France and the Netherlands.

**Status**: Confirmed.

**Records**: Germany (1), United Kingdom (5).

**First record**: 4.II.1923 (Hancock (1923), United Kingdom).

**Hibernacula**: LV (8), U (1).

**Sources**: Hancock (1923), Schmidt and Zmudzinski (2005); Unpublished: iRecord.

#### Ichneumon ligatorius

Thunberg, 1824

C07073FB-2F96-55D2-9D27-92B716B5F8D8

##### Notes

This species is easily recognised by the broadened hind tarsi (Hilpert 1992).

**Status**: Confirmed.

**Records**: Belgium (1)*, France (1), Germany (4), Ireland (1), Romania (2).

**First record**: Ca. 1873 (Tischbein (1873b), Germany).

**Hibernacula**: DT (3), M (2), S (1), U (4).

**Sources**: Tischbein (1873b) [*Ichneumon gradarius* W.], Berthoumieu (1894) [*I. gradarius* Wesm.], Johnson (1920) [*I. gradarius* Wesm.], Heinrich (1949a)/Heinrich (1949b), Constantineanu (1956) and Constantineanu (1959), Bauer (1984); Unpublished: Coll. RBINS.

**Also mentioned in**: Rasnitsyn (1964).

#### Ichneumon lugens

Gravenhorst, 1829

0719D0DD-BC9B-54B5-B1E6-026B45E510FF

##### Notes

One of the most common ichneumonids hibernating below tree bark in north-western Europe.

**Status**: Confirmed.

**Records**: Austria (9), Belarus (1)*, Belgium (176), Bulgaria (2), Czechia (10)*, Finland (1)*, France (41), Germany (58), Hungary (8), Ireland (1), Italy (7)*, Kosovo (1)**, Latvia (12)*, Lithuania (8)*, Norway (4)**, Poland (7)*, Romania (17), Russia (16), Slovakia (1)**, Spain (1)*, Sweden (7)*, Switzerland (1)*, the Netherlands (138), Ukraine (35), United Kingdom (2)*.

**First record**: Ca. 1873 (Tischbein (1873a), Germany).

**Hibernacula**: DT (934), DTCL (2), M (32), U (9).

**Sources**: Tischbein (1873a) [*Chasmodes lugens* Gr.], Mocsáry (1885) [*C. lugens* Grav.], Berthoumieu (1894) [*C. lugens* Grav.], Athimus (1901a) [*C. lugens* Gr.], Johnson (1912), Habermehl (1916) [*C. lugens* Grav.], Pic (1916), Pic (1917) and Pic (1919) [*C. lugens* Grav.], Seyrig (1923) [*C. lugens* Gr.], Constantineanu (1929b) and Constantineanu (1959) [*Chasmias lugens* Grav.], Györfi (1948), Rasnitsyn (1959) and Rasnitsyn (1964), Aubert (1960) [*C. lugens* Grav.], Györfi (1963) [*C. lugens* Grv.], Constantineanu (1969) [*C. lugens* Grav.], Valemberg and Vago (1974a), Valemberg (1976b) and Valemberg (1982), Valemberg and Bosquit (1979), Bauer (1984), Kolarov (1992), Hinz and Kreissl (1992) and Hinz and Kreissl (1993), Sebald et al. (2001), Schmidt and Zmudzinski (2005), Valemberg and Vago (2013), Lungu-Constantineanu and Constantineanu (2014) [*C. lugens*], Pénigot (2020), Varga et al. (2020), Verheyde and Quicke (2022); Unpublished: Artsobservasjoner, Artportalen, Coll. RBINS, Dabasdati, Facebook (Hymenopterists Forum), iNaturalist, Insecte.org, iRecord, Naturgucker, Pers. obs., UKRbin.

**Also mentioned in**: Morley (1903), Hinz and Horstmann (1999).

#### Ichneumon luteipes

Wesmael, 1855

EBCC29F0-F32B-587F-9B68-82DD00AE5AB7

##### Notes

No specimens have been reported in hibernation in the last hundred years. Although it seems to be mountainous, occurring in the Alps (Hilpert 1992), no data exist from the recent publications of Bauer and Sebald. Erroneously mentioned for Belgium in Verheyde and Quicke (2022) (*T. camelinus*, Valemberg misident.).

**Status**: Confirmed.

**Records**: France (2), Germany (2).

**First record**: 21.IV.1843 (Kriechbaumer (1881), Germany).

**Hibernacula**: DT (1), M (3).

**Sources**: Tischbein (1876), Kriechbaumer (1881) [*Ichneumon indiscretus*], Berthoumieu (1894), Seyrig (1923).

**Also mentioned in**: Constantineanu (1959), Verheyde and Quicke (2022).

#### Ichneumon magistratus

Hilpert, 1992

19C04500-03FC-597B-916C-F151B230A01E

##### Notes

Quite recently described in Hilpert (1992), where it is considered an eastern Palaearctic species. At least one paratype specimen was found ‘on hybernation’ in the eastern side of Russia near Vladivostok by A. Rasnitsyn. There are four other recent observations from Norway, most of them on birch.

**Status**: Confirmed.

**Records**: Norway (4), Russia (1) [eastern Palaearctic].

**First record**: 2.V.1962 (Hilpert (1992), Russia).

**Hibernacula**: DT (7), U (1).

**Sources**: Hilpert (1992), Mjøs et al. (2023).

#### Ichneumon melanobatus

Gravenhorst, 1829

D6BBDFDF-9E45-5DFB-94BC-2259D038681E

##### Notes

It is very likely this species can be found hibernating in all Scandinavian countries, where it is not uncommon (GBIF). There is one uncertain record from Sweden in 1927 (GBIF).

**Status**: Confirmed.

**Records**: Bulgaria (1), Norway (1)*.

**First record**: 18-26.II.1990 (Kolarov (1992), Bulgaria).

**Hibernacula**: DT (2), DTCL (1).

**Sources**: Kolarov (1992); Unpublished: Artsobservasjoner.

#### Ichneumon melanosomus

Wesmael, 1855

F0CA86F0-6C75-5908-ADD1-90228C7BA689

##### Notes

Another mountainous species (Hilpert 1992), not collected recently.

**Status**: Confirmed.

**Records**: Romania , 8.XI.1926.

**Hibernacula**: DT (1).

**Sources**: Constantineanu (1929b).

**Also mentioned in**: Constantineanu (1959).

#### Ichneumon melanotis

Holmgren, 1864

B0CDC6A5-77A3-5671-8F50-CC431EE95D9E

##### Notes

Very similar to darker forms of *I. molitorius*. The red forms can be confused with, for example, some varieties of *I. albiger*. The only distinctive differences between *I. melanotis* and *I. molitorius* are the width of the hindtarsus and the absence or presence of the scopa (Hilpert 1992). As a consequence, ca. 15 records could not be included, although it is very likely the majority are *I. melanotis*. These records would contain new data for Denmark and the Netherlands.

**Status**: Confirmed.

**Records**: Austria (5), Belarus (5), Belgium (10), France (9), Germany (10), Norway (4)*, Romania (3), Russia (2), Sweden (1), United Kingdom (2).

**First record**: Ca. 1850-1856 (Holmgren (1864), Sweden).

**Hibernacula**: DT (64), DTCL (2), DV (3), LV (4), M (3), S (2), U (12).

**Sources**: Holmgren (1864), Tischbein (1873b), Berthoumieu (1894), Meyer (1913) [*Ichneumon macrocerus* Thoms.], Pfeffer (1913), Pic (1916) and Pic (1917) [*I. macrocerus* Thoms.], Hancock (1923), Seyrig (1926a) [*I. macrocerus* Thoms.], Constantineanu (1929a), Habermehl (1929) [I. molitorius var. melanotis Holmgr.], Heinrich (1949a) [*Pterocormus melanotis* Holmgr.] and Heinrich (1951), Constantineanu (1959) [I. molitorius var. discolor Berth.], Valemberg and Vago (1974b) [I. molitorius var. discolor Berth.], Valemberg (1976a)/Valemberg (1976b) and Valemberg (1982) [I. molitorius var. discolor Bert.], Bauer (1984), Gokhman (1990b), Hinz and Kreissl (1992) and Hinz and Kreissl (1993), Sebald et al. (2001), Schmidt and Zmudzinski (2005), Gokhman (2014), Verheyde and Quicke (2022); Unpublished: Artsobservasjoner, Coll. RBINS, GBIF, Pers. obs., Tereshkin (2014).

**Also mentioned in**: Morley (1903), Rasnitsyn (1964) [*I. melanotus* Holm.], Hinz (1991), Tereshkin (2002), Gokhman (2003).

#### Ichneumon memorator

Wesmael, 1845

C4A3A12D-49CE-5CCD-888E-7495246C19C8

##### Notes

None.

**Status**: Confirmed.

**Records**: France (3), Germany (6), Romania (1), Russia (2).

**First record**: Ca. 1863 - February (Tischbein (1863), Germany).

**Hibernacula**: DT (208), DTCL (1), LV (1), M (2), S (1), U (1).

**Sources**: Tischbein (1863) [*Ichneumon nemorator* W.], Pfeffer (1913), Seyrig (1923) [*I. incomptus* Hlm.], Constantineanu (1959) [*I. incomptus* Holmgr.], Rasnitsyn (1959), Valemberg and Vago (1974a), Bauer (1984), Hilpert (1987a), Sebald et al. (2001), Gokhman and Mikhailenko (2008); Unpublished: GBIF

**Also mentioned in**: Tischbein (1871), Rasnitsyn (1964).

#### Ichneumon minutorius

Desvignes, 1856

5BE26361-63C1-5EFB-913C-F53A74E374F9

##### Notes

None.

**Status**: Confirmed.

**Records**: Austria (1), Belarus (4), France (7), Germany (9), Norway (1)*, Russia (1), Sweden (1)**, United Kingdom (3)*.

**First record**: 1915-1922 (Seyrig (1923), France).

**Hibernacula**: DT (29), DTCL (16), DV (1), LV (3), M (3), S (1), U (5).

**Sources**: Seyrig (1923) [*Ichneumon captorius* Thoms.], Heinrich (1949a)/Heinrich (1949b), Valemberg (1976a) [*I. xanthognathus* Thoms.], Valemberg (1982) [*I. xanthognathus* Thoms.], Bauer (1984), Gokhman (1987), Hilpert (1987a), Hinz and Kreissl (1993), Sebald et al. (2001), Schmidt and Zmudzinski (2005), Valemberg and Vago (2013); Unpublished: Artportalen, Artsobservasjoner, Tereshkin (2014).

**Also mentioned in**: Hinz and Horstmann (1998), Tereshkin (2002), Gokhman (2003).

#### Ichneumon molitorius

Linnaeus, 1761

B045E873-C5A4-5051-91A3-ABD81BD01427

##### Notes

Similar to *I. melanotis*. Rasnitsyn (1964) refers to his own publication from 1959, but, although the species is mentioned in the text, it is missing from his checklist.

**Status**: Confirmed.

**Records**: Belarus (4), Belgium (1), France (26), Germany (6), Hungary (1), Norway (2)*, Romania (5), Russia (3), United Kingdom (3).

**First record**: Ca. 1873 (Tischbein (1873b), Germany).

**Hibernacula**: DT (62), DTCL (1), LV (3), M (10), U (6).

**Sources**: Tischbein (1873b), Mocsáry (1885), Berthoumieu (1894), Athimus (1901b), Morley (1903), Hancock (1923), Seyrig (1923) and Seyrig (1926a)/Seyrig (1926b), Constantineanu (1929a), Heinrich (1949a) [*Pterocormus molitorius* L.], Aerts (1957), Constantineanu (1959), Rasnitsyn (1964), Constantineanu (1969), Bauer (1984), Gokhman (1990b), Sebald et al. (2001), Tereshkin (2002), Pénigot (2020); Unpublished: Artsobservasjoner, Insecte.org, GBIF, Pers. obs., Pers. obs. (H. Haraldseide), Tereshkin (2014).

**Also mentioned in**: Tereshkin (2002), Gokhman (2003).

#### Ichneumon mordax

Kriechbaumer, 1875

601ECA32-D7F7-5A90-AB3C-ABF4CB7C0943

##### Notes

Erroneously reported for France in Valemberg and Vago (1975), but corrected by the same author in Valemberg (1976b) as *Ichneumon albiger*.

**Status**: Confirmed.

**Records**: Romania (2).

**First record**: 19.III.1927 (Constantineanu (1956), Romania).

**Hibernacula**: DT (2).

**Sources**: Constantineanu (1956).

#### Ichneumon multipictus

Gravenhorst, 1820

EBFFE85A-F609-5398-AAAD-404479BAD10A

##### Notes

None.

**Status**: Confirmed.

**Records**: Germany (1), Poland (1), Russia (1), United Kingdom (1).

**First record**: Ca. 1903 - March (Morley (1903), United Kingdom).

**Hibernacula**: DT (1), LV (12), M (1), S (1).

**Sources**: Morley (1903), Heinrich (1935) [*Ichneumon suturalis* Holmgr.], Rasnitsyn (1959), Bauer (1984).

**Also mentioned in**: Constantineanu (1959), Rasnitsyn (1964).

#### Ichneumon nebulosae

Hinz, 1975

61F05D8D-AF43-5B39-A7BA-4004B85C5051

##### Notes

The holotype was caught hibernating in the French Jura. Experiments in the lab confirmed the diapause (Hinz 1975a). Since then, the species has also been found in Germany and Norway (GBIF), but none of the specimens was hibernating.

**Status**: Confirmed.

**Records**: France, 20.III.1972.

**Sources**: Hinz (1975a).

#### Ichneumon novemalbatus

Kriechbaumer, 1875

4CD72A98-EB10-5693-AFA4-6BA8B157475C

##### Notes

None.

**Status**: Confirmed.

**Records**: Germany (1), Romania (1).

**First record**: 14.XI.1926 (Constantineanu (1929b), Romania).

**Hibernacula**: DT (1), S (1).

**Sources**: Constantineanu (1929b), Bauer (1984).

**Also mentioned in**: Constantineanu (1959).

#### Ichneumon obliteratus

Wesmael, 1855

C752CCC6-0899-53C0-A92B-303354CDB232

##### Notes

Sebald et al. (2000) include it, stating that the label in Bauer’s collection records the specimen as having been collected while overwintering in situ. It is mainly known from the higher Alps (Hilpert 1992).

**Status**: Confirmed.

**Records**: Germany, 1980-2000.

**Sources**: Sebald et al. (2000).

#### Ichneumon oblongus

Schrank, 1802

1CEF326F-BCCA-5C8A-9FB5-A67BDEF2A05A

##### Notes

Resembling other smaller species in appearance, such as *I. simulans*. Some females are brachypterous. One of the more common species in grass tussocks and moss, but sometimes below bark too. Many records exist from the United Kingdom, which has a great tradition of ‘tussocking’. Additional uncertain records exist for Belgium and Germany (Coll. RBINS; GBIF).

**Status**: Confirmed.

**Records**: Belarus (1), Belgium (3), Czechia (3)**, Denmark (1)**, France (9), Germany (6), Ireland (4), Norway (4)*, Poland (1), Romania (3), Russia (1), Sweden (1)*, United Kingdom (50).

**First record**: Ca. 1863 (Tischbein (1863), Germany).

**Hibernacula**: DT (37), DV (4), LV (78), M (31), S (12), U (7).

**Sources**: Tischbein (1863) [*Ichneumon lutrator* W.], Tischbein (1873b) [*I. latrator* W.], Berthoumieu (1894) [*I. latrator* Fabr.], Athimus (1901a) [*I. latrator* Wesm.], Morley (1903) [*I. latrator* Fab.], Torka (1915) [*I. latrator* F.], Johnson (1920) [*I. latrator* Fab.], Hancock (1923) and Hancock (1925) [*I. latrator* Fabr.], Seyrig (1923) [*I. latrator* F.], Constantineanu (1929b) [*I. latrator* Fabr.], Heinrich (1949a) [*Pterocormus latrator* F.], Heinrich (1951) [*I. latrator* F.], Constantineanu (1959) [*I. latrator* Fabr.], Rasnitsyn (1959) [*I. latrator* F.], Valemberg and Vago (1974b) and Valemberg and Vago (1975) [*I. latrator* F.], Valemberg (1976b) [*I. latrator* F.], Bauer (1984) [*I. latrator* F.]; Unpublished: Artportalen, Coll. RBINS, iRecord, Naturbasen, Pers. obs., Tereshkin (2014).

**Also mentioned in**: Tischbein (1871) [*I. latrator* W.], Rasnitsyn (1964) [*I. latrator* F.], Tereshkin (2002) [*I. latrator* Fabr.].

#### Ichneumon oviventroides

Hinz, 1975

9DF6C717-DBFC-50A3-9773-E6D69FD9AA8B

##### Notes

All known specimens were collected hibernating, in moss on stones. The third specimen hibernating in the French Jura, was caught on the same day as *I. nebulosae*, which has a similar ecology (Hinz 1975a, Hilpert 1992). In contrast to this latter species, however, *I. oviventroides* is also mentioned to hibernate in Bauer's collection (Sebald et al. 2000).

**Status**: Confirmed.

**Records**: France (3), Germany (2).

**First record**: 30.X.1971 (Hinz (1975b), Germany).

**Hibernacula**: S (6), U (1).

**Sources**: Hinz (1975b), Sebald et al. (2000).

#### Ichneumon parengensis

Kiss, 1929

38AC2E1A-2241-5C8E-86FD-C606D1409594

##### Notes

Sebald et al. (2000) include it, stating that the label in Bauer’s collection records the specimen as having been collected while overwintering in situ.

**Status**: Confirmed.

**Records**: Germany, 1980-2000.

**Sources**: Sebald et al. (2000).

#### Ichneumon phaeostigmus

Wesmael, 1857

5DC1C151-88C2-50C6-9BD3-AC9640F9C185

##### Notes

Sebald et al. (2000) include it, stating that the label in Bauer’s collection records the specimen as having been collected while overwintering in situ.

**Status**: Confirmed.

**Records**: Germany, 1980-2000.

**Sources**: Sebald et al. (2000).

#### Ichneumon porcellus

Hilpert, 1992

268D6691-F389-5931-A0F9-0E5A1E79DF56

##### Notes

Sebald et al. (2000) include it, stating that the label in Bauer’s collection records the specimen as having been collected while overwintering in situ. The holotype was possibly hibernating too, as it was found on 31.X.1943 in Germany (Hilpert 1992).

**Status**: Confirmed.

**Records**: Germany, 1980-2000.

**Sources**: Sebald et al. (2000).

#### Ichneumon primatorius

Forster, 1771

F0774208-1C12-57E1-8AEF-12EDE770C83A

##### Notes

A species which is easily recognised by its size and white coxa. The historical data from north-western Europe seems to show it used to be more common in the first half of the 20^th^ century and earlier. In most countries, it is now confined to coastal dunes near the North Sea and Baltic Sea and/or inland calcareous grasslands where its main host, *Arctia caja* (L.) (Lepidoptera, Erebidae) still occurs. Mostly found under bark.

**Status**: Confirmed.

**Records**: Belarus (2), Belgium (4), Czechia (1)*, Denmark (2)*, France (12), Germany (5), Latvia (2)*, Poland (1)*, Russia (1), Sweden (1)*, the Netherlands (5).

**First record**: 1915-1922 (Seyrig (1923), France).

**Hibernacula**: DT (33), DTCL (1), M (4), U (7).

**Sources**: Seyrig (1923) and Seyrig (1926b), Heinrich (1949a) [*Pterocormus primatorius* Forst.], Heinrich (1951), Aerts (1957), Rasnitsyn (1959), Valemberg and Vago (1974b), Valemberg (1978) and Valemberg (1982), Bauer (1984), Sebald et al. (2001), Valemberg and Vago (2013), Verheyde and Quicke (2022); Unpublished: Artportalen, Coll. RBINS, Dabasdati, iNaturalist, Naturbasen, Observation.org, Pers. obs., Tereshkin (2014).

**Also mentioned in**: Rasnitsyn (1964), Hinz and Horstmann (1998), Tereshkin (2002).

#### Ichneumon proletarius

Wesmael, 1848

4F7548F2-569C-5FAA-A954-248C215EB359

##### Notes

Very common near the Mediterranean sea (Hilpert 1992). Constantineanu (1929b) says it is the most common species during his surveys, sometimes observing more than a hundred specimens on a dead tree trunk.

**Status**: Confirmed.

**Records**: France (4), Germany (2), Romania (14), Russia (2).

**First record**: Ca. 1879 (Tischbein (1879), Germany).

**Hibernacula**: DT (17), DTCL (1), LV (3), M (2), U (5).

**Sources**: Tischbein (1879) [*Ichneumon intermixtus* Tischb.], Berthoumieu (1894), Seyrig (1923), Constantineanu (1929a)/Constantineanu (1929b) and Constantineanu (1959), Rasnitsyn (1964) [*I. intermixtus* Tischb.], Constantineanu (1969), Sebald et al. (2001), Lungu-Constantineanu and Constantineanu (2014).

#### Ichneumon pseudocaloscelis

Heinrich, 1949

A590DB72-6A68-5192-8BA7-A79A82BE9BBD

##### Notes

The taxonomic status of some populations is unclear (Hilpert 1992). Hibernacula unknown.

**Status**: Confirmed.

**Records**: Austria (1)*, Germany (1).

**First record**: 25.V.1947 (Heinrich (1949b), Germany).

**Sources**: Heinrich (1949b); Unpublished: GBIF.

#### Ichneumon pygolissus

Heinrich, 1951

A16474B9-30B1-5E96-A4FF-1F8F568D3444

##### Notes

Sebald et al. (2000) include it, stating that the label in Bauer’s collection records the specimen as having been collected while overwintering in situ.

**Status**: Confirmed.

**Records**: Germany, 1980-2000.

**Sources**: Sebald et al. (2000).

#### Ichneumon quadrialbatus

Gravenhorst, 1820

AB0EB122-5650-5A74-BD18-06E7A0C9A27B

##### Notes

Berthoumieu (1894) reports the species as hibernating and refers to Tischbein, but this original record could not be found.

**Status**: Confirmed.

**Records**: France (1)* - Fig. 6a, Romania (4), Russia (2).

**First record**: 1951-1958 (Rasnitsyn (1959), Russia).

**Hibernacula**: DT (9), S (1), U (1).

**Sources**: Constantineanu (1959), Rasnitsyn (1959) and Rasnitsyn (1964), Constantineanu (1969); Unpublished: iNaturalist.

**Also mentioned in**: Berthoumieu (1894), Morley (1903).

#### Ichneumon quadriannellatus

Thomson, 1893

17178281-E2C6-5ABF-B01B-CDE11167BE4C

##### Notes

The context of this finding is unclear.

**Status**: Confirmed.

**Records**: Russia, 1959-1964.

**Sources**: Rasnitsyn (1964).

#### Ichneumon quaesitorius

Linnaeus, 1761

8DE2E1E7-5C56-5E78-A812-A5272004E403

##### Notes

None.

**Status**: Confirmed.

**Records**: France (2), Romania (5), Ukraine (1)*.

**First record**: 1924 (Seyrig (1926b), France).

**Hibernacula**: DT (7), M (9).

**Sources**: Seyrig (1926b), Constantineanu (1929a) and Constantineanu (1959) [incl. *Ichneumon insignis* Berth.], Valemberg (1982); Unpublished: Pers. obs.

#### Ichneumon rudolphi

Holmgren, 1884

215D7397-2378-54E1-9F80-3B38BFBC232A

##### Notes

This species was recently discovered hibernating below the bark of a *Pinus* sp. Only known from Scandinavia (GBIF, Hilpert (1992)).

**Status**: Confirmed*.

**Records**: Norway, 13.X.2022 - Fig. 6b.

**Hibernacula**: DT (1).

**Sources**: Artsobservasjoner.

#### Ichneumon rufigena

Kriechbaumer, 1875

C4D99ACF-0092-5358-B4E9-956B272D5533

##### Notes

The sole finding was found hibernating in June, at a height of 1200 metres in the Alps (Heinrich 1949b).

**Status**: Confirmed.

**Records**: Germany, V.1947-1948.

**Sources**: Heinrich (1949b) [*Ichneumon ruficollis* Hgn.].

#### Ichneumon sarcitorius

Linnaeus, 1758

2044C8B1-0C4A-515B-9883-FE2D5C1C5E51

##### Notes

Similar to *I. lautatorius*. Although this species is very common in many European countries, especially in summer months on umbellifers, hibernation records are relatively rare and mostly recent. This may suggest either facultative hibernation or a strict preference for using certain hibernacula (or a combination of both), in this case apparently grass tussocks and stones. This is reflected in the country records, with only one record for both France and Germany (little tradition in tussocking), but quite a lot of records for the United Kingdom (good tradition of tussocking). Very recently in 2024, two specimens were discovered under bark for the first time in Belgium and Germany.

**Status**: Confirmed.

**Records**: Belgium (7), Denmark (4)*, France (1), Germany (2), Russia (1), Spain (1)*, Sweden (1)*, the Netherlands (2), Ukraine (1)*, United Kingdom (12).

**First record**: Ca. 1894 (Berthoumieu (1894), France).

**Hibernacula**: DT (4), DV (3), LV (19), M (2), O (3), S (5), U (2).

**Sources**: Berthoumieu (1894), Morley (1903), Hancock (1923), Uffeln (1931), Leclercq (1942), Rasnitsyn (1964), Verheyde and Quicke (2022), Storey et al. (2025); Unpublished: Artportalen, Coll. RBINS, iNaturalist, iRecord, Naturbasen, Observation.org, Pers. obs.

**Also mentioned in**: Constantineanu (1959).

#### Ichneumon sculpturatus

Holmgren, 1864

D6674CEF-0514-5DD4-A548-0CD59FA3E9FD

##### Notes

None.

**Status**: Confirmed.

**Records**: Germany (1), Russia (1).

**First record**: Ca. 1984 (Bauer (1984), Germany).

**Hibernacula**: DT (1), S (1).

**Sources**: Bauer (1984) [*Ichneumon nereni* Thoms.], Gokhman (1990b) [*I. nereni* Thoms].

#### Ichneumon sexcinctus

Gravenhorst, 1829

11553C9F-6FEE-57ED-9F9A-6A9577ADAC7B

##### Notes

Sebald et al. (2000) include it, stating that the label in Bauer’s collection records the specimen as having been collected while overwintering in situ.

**Status**: Confirmed.

**Records**: Germany, 1980-2000.

**Sources**: Sebald et al. (2000).

#### Ichneumon silaceus

Gravenhorst, 1829

E26468D8-2DC2-58AA-A38E-A15CD2494669

##### Notes

Sebald et al. (2000) include it, stating that the label in Bauer’s collection records the specimen as having been collected while overwintering in situ.

**Status**: Confirmed.

**Records**: Germany, 1980-2000.

**Sources**: Sebald et al. (2000).

#### Ichneumon simulans

Tischbein, 1873

2A1EC6FA-FFA6-5DC0-8B44-50328E5AA918

##### Notes

Similar in appareance to other smaller Ichneumoninae, for example, *I. oblongus*.

**Status**: Confirmed.

**Records**: Austria (5), Belarus (3), Belgium (6)*, Czechia (1)*, Denmark (1)*, France (17), Germany (12), Ireland (3), Romania (12), Russia (3), Sweden (1)*, Switzerland (1)*, Ukraine (2)*, United Kingdom (13).

**First record**: Ca. 1903 (Morley (1903), United Kingdom).

**Hibernacula**: DT (125), DTCL (59), DV (5), LT (1), LV (25), M (17), U (6).

**Sources**: Pic (1917) [*Ichneumon subquadratus* Thoms.], Seyrig (1923) and Seyrig (1926a) [*I. subquadratus* Thoms.], Constantineanu (1929a)/Constantineanu (1929b) and Constantineanu (1959) [*I. subquadratus* Thoms.], Rasnitsyn (1959) and Rasnitsyn (1964) [*I. subquadratus* Thoms.], Constantineanu (1969) [*I. subquadratus* Thoms.], Valemberg and Vago (1975) [*I. subquadratus* Thoms.], Valemberg (1976b) and Valemberg (1982) [*I. subquadratus* Thoms.], Bauer (1984) [*I. subquadratus* Thoms.], Hilpert (1989) [*I. subquadratus* Ths.], Gokhman (1990a) [*I. subquadratus* Thoms.], Hinz and Kreissl (1992) and Hinz and Kreissl (1993) [*I. subquadratus* Thoms.], Sebald et al. (2001), Schmidt and Zmudzinski (2005), Valemberg and Vago (2013), Lungu-Constantineanu and Constantineanu (2014), Pénigot (2020); Unpublished: Artportalen, Coll. RBINS, iRecord, Naturbasen, Pers. obs., Pers. obs. (G. Artmann), Tereshkin (2014).

**Also mentioned in**: Tereshkin (2002), Gokhman (2003).

#### Ichneumon spurius

Wesmael, 1848

F9264BE0-5C03-541D-A3A6-6EE515FAAC65

##### Notes

None.

**Status**: Confirmed.

**Records**: Austria (1), Belgium (1)*, France (3), Germany (3), Romania (1), United Kingdom (1)*.

**First record**: Ca. 1863 (Tischbein (1863), Germany).

**Hibernacula**: DT (8), M (2), S (1), U (1).

**Sources**: Tischbein (1863), Berthoumieu (1894), Constantineanu (1929a), Heinrich (1949a) [*Barichneumon spurius* Wesm.], Bauer (1984), Hinz and Kreissl (1993), Valemberg and Vago (2013); Unpublished: Observation.org, Pers. obs.

**Also mentioned in**: Tischbein (1871) and Tischbein (1873b), Morley (1903), Constantineanu (1959).

#### Ichneumon stenocerus

Thomson, 1887

F3E0267A-3961-5D9A-8795-33C764DE6B3F

##### Notes

Very similar to *I. emancipatus* and *I. gracilicornis* (Hilpert 1992). According to Hilpert, it has a more southern distribution, occurring in the upland regions. The latest French specimen was found at a height of 860 metres at the Puy-de-Dôme. Its possible older records concern *I. gracilicornis* in reality.

**Status**: Confirmed.

**Records**: France (2).

**First record**: 17.II.1919 (Pic (1919), France).

**Hibernacula**: DT (1), U (1).

**Sources**: Pic (1919) [Ichneumon gracilicornis v. stenocerus Thms.], Valemberg (1976b) [I. gracilicornis var. stenocerus Thoms.].

#### Ichneumon stigmatorius

Zetterstedt, 1838

4C4561F0-5418-5F0F-9CE2-52DCE72E1C4D

##### Notes

Similar to *I. dolosus* and *I. ignobilis*. Berthoumieu (1894) reports the species as hibernating and refers to Tischbein, but this original record could not be found. Probably he refers to Tischbein's report of *I. perhiematus*, which was considered a variety at that time. One uncertain record exists of a specimen in Sweden (GBIF). Morley (1903) mentions it is common there and hibernates below moss, but this statement could not be found in the original description of Zetterstedt (1838), nor in the comments of Holmgren (1864).

**Status**: Confirmed.

**Records**: France (1), Germany (3), Russia (2).

**First record**: 1915-1922 (Seyrig (1923), France).

**Hibernacula**: DTCL (1), M (3), U (4).

**Sources**: Seyrig (1923), Bauer (1984) [*Ichneumon walkeri* W.], Sebald et al. (2000), Schmidt and Zmudzinski (2005).

**Also mentioned in**: Berthoumieu (1894), Morley (1903), Heinrich (1949b) [*I. eremitatorius* Zett.], Constantineanu (1959) [*I. eremitatorius* Zett.], Rasnitsyn (1964) [*I. eremitatorius* Zett.].

#### Ichneumon stramentarius

Gravenhorst, 1820

00537A4D-E285-5E4C-83DC-E478F14D8222

##### Notes

Similar to *I. suspiciosus*. Both species have the last three apical segments covered with a white spot, a yellow band on the hind tibia and T2-T3 orange to red. *I. stramentarius* usually has more flagellomeres, a narrow patch of hairs on the hind coxa, the hind tarsus broadened and also often has tinges of yellow shining through the second and third tergite. Usually, the number of specimens clustering together is lower. Records (126) of 232 specimens could not be included. These records possibly contain new hibernation records for the Czechia, Latvia, Lithuania, Norway and Poland. Both species are well distributed through Europe. In some countries (e.g. Belgium, France), *I. suspiciosus* seems to be more common, while in others (e.g. United Kingdom), this is vice-versa. The common continental subspecies is called ssp. stramentarius, in Scandinavia spp. *septentrionalis* occurs, having the legs much redder. Unfortunately, some of the older records possibly relate to *I. stramentor*, as this species was only described in 1981. For example, Valemberg uses *I. septentrionalis*-*atrifemur* to refer to *I. stramentarius* and *I. stramentarius* to refer to *I. stramentor*. For some authors, the species concept was unclear and the data were not included (e.g. Leclercq (1942), Aubert (1960)).

**Status**: Confirmed.

**Records**: Belarus (7), Belgium (10), Czechia (1)*, Denmark (2)**, Finland (2)*, France (12), Germany (6), Norway (12)*, Russia (3), Sweden (2)*, the Netherlands (1)*, United Kingdom (17).

**First record**: Ca. 1871 (Tischbein (1871), Germany).

**Hibernacula**: C (5), DT (88), DTCL (4), LV (2), M (4), S (1), U (6).

**Sources**: Tischbein (1871), Berthoumieu (1894), Pic (1916) and Pic (1917), Seyrig (1923) and Seyrig (1926b), Perkins (1952) [*I. septentrionalis scelestus*], Valemberg and Vago (1974a) [*I. macrocérophorus* D.T.], Valemberg (1982) [*I. septentrionalis*-*atrifemur* D.T.], Gokhman (1987), Sebald et al. (2001), Schmidt and Zmudzinski (2005), Valemberg and Vago (2013), Verheyde and Quicke (2022); Unpublished: Artportalen, Artsobservasjoner, Coll. RBINS, iNaturalist, iRecord, Naturbasen, Observation.org., Pers. obs., Tereshkin (2014).

**Also mentioned in**: Tischbein (1873b), Constantineanu (1959), Tereshkin (2002), Gokhman (2003).

#### Ichneumon stramentor

Rasnitsyn, 1981

E483CFAD-0625-55F7-8022-0D870394141D

##### Notes

Similar to *I. terminatorius*. One of the most common species in north-western Europe below bark. Very little certain historic data exist as the older specimens were often reported as *I. stramentarius* and it is not always possible to check which species was referred to with absolute certainty. We only included *I. stramentarius* as *I. stramentor* when in the same publication *I. macrocerus* was reported (now *I. stramentarius*).

**Status**: Confirmed.

**Records**: Armenia (1)**, Austria (7), Belgium (265), Bulgaria (4)**, Czechia (9)**, Denmark (37)**, France (30), Germany (30), Ireland (1)**, Poland (1)*, the Netherlands (146), United Kingdom (48).

**First record**: 18.II.1974 (Valemberg and Vago (1974a), France).

**Hibernacula**: DT (953), DTCL (6), LV (2), M (1), O (3), U (11).

**Sources**: Valemberg and Vago (1974a)/Valemberg and Vago (1974b), Valemberg and Vago 1975, Valemberg 1976a/Valemberg 1976b [*I. stramentarius* Wesm.], Valemberg and Bosquit 1979 [*I. stramentarius W*esm.], Valemberg (1982) [*I. stramentarius* Wesm.], Hinz and Kreissl (1992) and Hinz and Kreissl (1993), Sebald et al. (2001), Schmidt and Zmudzinski (2005), Valemberg and Vago (2013), Pénigot (2020), Verheyde and Quicke (2022); Unpublished: Arter DK, Coll. RBINS, iNaturalist, Insecte.org, iRecord, Naturbasen, Naturgucker, Observation.org, Pers. obs., Pers. obs. (P.N. Libert).

**Also mentioned in**: Sebald et al. (2000).

#### Ichneumon submarginatus

Gravenhorst, 1829

8E278D6F-8806-5BE6-9E91-A39AE47E90E8

##### Notes

Similar to *I. oviventroides* (Hinz 1975b, Hilpert 1992).

**Status**: Confirmed.

**Records**: France (2), Germany (3), Hungary (1), Russia (2).

**First record**: 19.IV.1941 (Györfi (1948), Hungary).

**Hibernacula**: DT (2), S (4), U (2).

**Sources**: Györfi (1948), Rasnitsyn (1964) Hinz (1975b) [*Ichneumon oviventris* Kriechb.], Bauer (1984) [*I. oviventris* Kr.], Gokhman (1990b).

**Also mentioned in**: Hinz and Horstmann (1998), Gokhman (2003).

#### Ichneumon sulcatoriops

Hilpert, 1992

37F074FA-2F04-5210-888A-1D7AEC92B282

##### Notes

The holotype was possibly found hibernating.

**Status**: Confirmed.

**Records**: Germany (3).

**First record**: May 1947 (Heinrich (1949b), Germany).

**Hibernacula**: DTCL (1), S (1), U (1).

**Sources**: Heinrich (1949b) [*Ichneumon sulcatorius*], Bauer (1984), Schmidt and Zmudzinski (2005).

**Also mentioned in**: Sebald et al. (2000).

#### Ichneumon suspiciosus

Wesmael, 1845

274F2F9F-B918-583F-85B3-42545F8482E3

##### Notes

Similar to *I. stramentarius*. Seems to be slightly more opportunistic in hibernacula. Has been found on all kinds of dead trees (also without bark), below stones and in grass tussocks, but also even in caves (Seyrig 1926b), in a buried chestnut (Verheyde and Quicke 2022) or below sand bags (Naturbasen).

**Status**: Confirmed.

**Records**: Belgium (131), Bulgaria (1)**, Denmark (20)**, France (38), Germany (14), Ireland (2), Italy (1)*, Latvia (1)*, Poland (3)*, Romania (2), Russia (1), Sweden (4)*, Switzerland (3)*, the Netherlands (5), Ukraine (1)*, United Kingdom (25).

**First record**: Ca. 1863 - May (Tischbein (1863), Germany).

**Hibernacula**: C (2), DT (692), DTCL (2), DV (2), LV (24), M (12), O (2), S (2), U (23).

**Sources**: Tischbein (1863), Berthoumieu (1894), Kriechbaumer (1896a), Athimus (1901b), Pic (1919), Johnson (1920), Hancock (1923) and Hancock (1925), Seyrig (1923) and Seyrig (1926a)/Seyrig (1926b), Heinrich (1949a)/Heinrich (1949b) and Heinrich (1951), Constantineanu (1959) [incl. *Ichneumon trispilus* Thoms.], Rasnitsyn (1959), Aubert (1960), Valemberg (1976b), Valemberg (1978) and Valemberg (1982), Bauer (1984), Hilpert (1989), Schmidt and Zmudzinski (2005), Valemberg and Vago (2013), Pénigot (2020), Verheyde and Quicke (2022); Unpublished: Artportalen, Coll. RBINS, Dabasdati, GBIF [I. macrocerus var. rufonotatus], iNaturalist, iRecord, Naturbasen, Observation.org, Pers. obs., Pers. obs. (G. Artmann).

**Also mentioned in**: Tischbein (1871) and Tischbein (1873b), Morley (1903), Rasnitsyn (1964) [incl. *I. trispilus* Thoms.].

#### Ichneumon terminatorius

Gravenhorst, 1820

86B35527-85DE-5AA2-8068-F19B24C24ED8

##### Notes

Superficially similar to *I. stramentor*, but with the hind tarsi paler and usually a narrow black band between T2 and T3 medially (Hilpert 1992).

**Status**: Confirmed.

**Records**: France (1), Germany (7), Ireland (2), Russia (2).

**First record**: Ca. 1863 (Tischbein (1863), Germany).

**Hibernacula**: DT (6), M (3), S (1), U (3).

**Sources**: Tischbein (1863), Berthoumieu (1894), Pfeffer (1913), Johnson (1920), Heinrich (1949b), Rasnitsyn (1959), Bauer (1984); Unpublished: GBIF, iNaturalist.

**Also mentioned in**: Tischbein (1871) and Tischbein (1873b), Morley (1903), Seyrig (1923), Constantineanu (1959), Rasnitsyn (1964).

#### Ichneumon tuberculipes

Wesmael, 1848

EB8FA477-8803-5202-BE75-9B1F9F51BB21

##### Notes

There is one uncertain hibernation record from Spain, 15.XII.1926 (GBIF).

**Status**: Confirmed.

**Records**: Denmark (3)**, France (2), Germany (3), Norway (2)*, Romania (4), Russia (1).

**First record**: Ca. 1873 - March (Tischbein (1873b), Germany).

**Hibernacula**: DT (9), DTCL (1), M (2), S (1), U (2).

**Sources**: Tischbein (1873b), Berthoumieu (1894), Constantineanu (1959), Rasnitsyn (1959), Constantineanu (1969), Valemberg (1982), Bauer (1984), Sebald et al. (2001); Unpublished: Naturbasen, Pers. obs.

**Also mentioned in**: Rasnitsyn (1964).

#### Ichneumon vafer

Tischbein, 1876

659EC1EE-BC9D-52BB-8B8C-26755B70893F

##### Notes

None.

**Status**: Confirmed.

**Records**: France (2), Germany (3).

**First record**: 1915-1922 (Seyrig (1923), France).

**Hibernacula**: DT (1), M (1), S (1), U (2).

**Sources**: Seyrig (1923) [*Ichneumon Rogenhoferi* Kr.], Heinrich (1949b) [*I. rogenhoferi* Kriechb.], Bauer (1984) [*I. rogenhoferi* Kr.], Sebald et al. (2000).

**Also mentioned in**: Hinz and Horstmann (1998).

#### Ichneumon validicornis

Holmgren, 1864

C81DC349-E682-5008-A183-AD8FEF8FD6E7

##### Notes

Some uncertain records from Germany exist (GBIF).

**Status**: Confirmed.

**Records**: Belgium (1), France (5), Germany (2), Russia (1).

**First record**: 1873 - Winter (Tischbein (1876), Germany).

**Hibernacula**: DT (2), M (42), U (1).

**Sources**: Tischbein (1876) [*Ichneumon vivacior* Tischb.], Berthoumieu (1894) [*I. vivacior* Tisch.], Athimus (1901b) [*I. vivacior* Fisch.], Seyrig (1923), Valemberg (1975) and Valemberg (1982), Bauer (1984), Gokhman and Quicke (1995).

**Also mentioned in**: Constantineanu (1959) [I. validicornis var. vivacior Tischb.], Gokhman (2003).

#### Ichneumon vorax

Geoffroy, 1785

9E4305BB-6CB4-553D-BAA8-387C863E92BB

##### Notes

None.

**Status**: Confirmed.

**Records**: Belarus (1), Germany (3), Russia (1).

**First record**: Ca. 1876 - 01 March (Tischbein (1876), Germany).

**Hibernacula**: DT (2), S (2), U (1).

**Sources**: Tischbein (1876) [*Ichneumon discriminator* W.], Bauer (1984) [*I. discriminator* W.], Sebald et al. (2000), Tereshkin (2002), Gokhman and Mikhailenko (2008).

#### Ichneumon vulneratorius

Zetterstedt, 1838

193318A5-3BCC-503A-AF30-BFFE4A7C36C7

##### Notes

None.

**Status**: Confirmed.

**Records**: Finland (1)*, Germany (1), Norway (1)*, Sweden (1), United Kingdom (1)*.

**First record**: Ca. 1838 (Zetterstedt (1838), Sweden).

**Hibernacula**: DT (2), S (2), U (1).

**Sources**: Zetterstedt (1838), Morley (1903), Heinrich (1949b) [*Ichneumon versutus* Holmgr.]; Unpublished: iNaturalist, Norway.

**Also mentioned in**: Holmgren (1864).

#### Ichneumon xanthorius

Forster, 1771

D279C18B-AD49-5CCC-A130-30CE6079F32D

##### Notes

A very common species in north-western Europe during the regular flying season, but rarely found hibernating (compare to *I. sarcitorius*). This may demonstrate hibernation is facultative or highly specialised. It has never been found directly below bark, all four reports on dead trees being below entire tree trunks (often highly rotten), stuck between the soil and the wood and, thus, similar to the niche below stones (where it has been reported the most). Some additional reports exist from litter and decaying vegetation. One report from grass tussocks by Morley (1903). It is noteworthy this is the sole report of this species in hibernation before 2017.

**Status**: Confirmed.

**Records**: Belgium (4), Germany (1)*, Italy (3)*, Sweden (1)**, the Netherlands (6), United Kingdom (1).

**First record**: Ca. 30.XI.1903 (Morley (1903), United Kingdom).

**Hibernacula**: DT (5), DV (4), LV (1), O (1), S (6), U (2).

**Sources**: Morley (1903), Verheyde and Quicke (2022); Unpublished: Artportalen, iNaturalist, Observation.org.

**Also mentioned in**: Constantineanu (1959), Rasnitsyn (1964).

#### Limerodops elongatus

(Brischke, 1865)

4B56189D-D7CD-508A-B485-D3EF9EAF8635

##### Notes

In Hinz and Horstmann (1999), hibernation is not considered an option, but there is at least one likely report of the species hibernating and three uncertain ones from Austria (GBIF), Finland and Hungary (iNaturalist). Hibernation is facultative or there was an issue with the identification.

**Status**: Unverified.

**Records**: Belarus, 29.X.1978.

**Hibernacula**: DT (1).

**Sources**: Tereshkin (2014).

#### Limerodops subsericans

(Gravenhorst, 1820)

247A9B1B-8029-53F1-82E4-40AF5A80DC01

##### Notes

In Hinz and Horstmann (1999), hibernation is not considered an option. All records come from caves. These findings could still be coincidental (indicated by the collection date in Dobat (1975) and Dobat (1978)) or the identification could be wrong.

**Status**: Unverified.

**Records**: Germany (2), Switzerland (1).

**First record**: 24.VI.1934 (Dobat (1978), Germany).

**Hibernacula**: C (3).

**Sources**: Aellen and Strinati (1962) [*Amblyteles subsericans* Grav.], Dobat (1975) & Dobat (1978) [*A. subsericans* Grav.].

**Also mentioned in**: Strinati (1966) [*A. subsericans*].

#### Limerodops unilineatus

Gravenhorst, 1829

7928DFD6-3BE1-5928-BD16-61E63A10A536

##### Notes

Presumed to hibernate as an adult (Hinz and Horstmann 1999), in contrast to the other species in the genus. However, no field data are known to us.

**Status**: Unverified.

**Sources**: Hinz and Horstmann (1999).

#### Lymantrichneumon disparis

(Poda, 1761)

7C5EB768-2EFB-5D84-930E-B0B1FCB5EDC2

##### Notes

Easily recognised, although specimens with a darker mesosoma are not uncommon and could be confusing. In several countries, the hibernation records (usually below bark) strongly exceed the number of observations during the rest of the year. Exceptionally, some records are known from buildings, caves (e.g. Schmitz (1909)) and cellars. It has also been found at more atypical structures, such as behind protective foil or in an empty beehive (Observation.org).

**Status**: Confirmed.

**Records**: Austria (14), Belgium (199), Croatia (1)*, Czechia (7)*, France (40), Germany (33), Hungary (7), Italy (14)*, Latvia (1)*, Lithuania (2), Poland (13), Romania (11), Russia (5), Serbia (1), Slovakia (1)*, Spain (4)*, Sweden (2)**, the Netherlands (49), Ukraine (13)*, United Kingdom (8)*.

**First record**: Ca. 1895 (Berthoumieu (1895a), France).

**Hibernacula**: B (3), C (7), DT (806), DTCL (15), M (7), O (4), U (16).

**Sources**: Berthoumieu (1895a) [*Ichneumon disparis* Poda], Athimus (1901b) [*I. disparis* Poda], Schmitz (1909) [*I. disparis* Poda], Pfeffer (1913) [*I. disparis* Poda], Seyrig (1923) [*Barichneumon disparis* Poda], Lauterborn (1926) [*I. disparis* Poda], Seyrig 1926b [*Protichneumon disparis* Poda], Constantineanu (1929b) and Constantineanu (1959) [*P. disparis* Poda], Heinrich (1930) [*P. disparis* Poda], Leclercq (1953) [*P. disparis* Poda], Györfi (1963) [*P. disparis* Poda], Rasnitsyn (1964) [*P. disparis* Poda], Constantineanu (1969) [*Melanichneumon disparis* Poda], Valemberg (1976b) and Valemberg (1982) [*I.* & *P. disparis* Poda], Jonaitis (1982), Bauer (1984), Hilpert (1989), Hinz and Kreissl (1992) and Hinz and Kreissl (1993), Sebald et al. (2001), Schmidt and Zmudzinski (2005), Gokhman and Mikhailenko (2008), Valemberg and Vago (2013), Lungu-Constantineanu and Constantineanu (2014), Pénigot (2020), Verheyde and Quicke (2022), Vesović et al. (2024); Unpublished: Coll. RBINS, Dabasdati, iNaturalist, Insecte.org, iRecord, Naturgucker, Observation.org, Pers. obs.

**Also mentioned in**: Morley (1903) [*P. disparis* Poda].

#### Melanichneumon albipictus

(Gravenhorst, 1820)

9025B3DB-1C0A-528F-9F54-A74C1CB7E409

##### Notes

Due to similar nomenclature, not to be confused with *Ichneumon multipictus*.

**Status**: Confirmed.

**Records**: France, ca. 1895.

**Sources**: Berthoumieu 1895a [*Ichneumon albipictus* Grav.].

**Also mentioned in**: Constantineanu (1959) [*Stenichneumon albipictus* Grav.].

#### Neotypus melanocephalus

(Gmelin, 1790)

624C9749-B588-502B-9BBF-9C5259116FC9

##### Notes

The lifecycle and phenology of *Neotypus* is well-known (Pfeifer 2016). It is unlikely this species hibernates.

**Status**: Incorrect.

**Records**: France, ca. 1896.

**Sources**: Berthoumieu (1896).

**Also mentioned in**: Constantineanu (1959).

#### Obtusodonta equitatoria

(Panzer, 1786)

8AE2E690-A36C-5C7A-9D58-ECB058B8FA01

##### Notes

The first hibernating specimen was found by Gravenhorst (1829) in Grunwitz (Poland) on pine. He mentions an additional record from Dahl, but the location is unclear. The distribution in Europe is mostly southern, with many records in Spain specifically. The species hibernates there as an adult below stones.

**Status**: Confirmed.

**Records**: France (1), Poland (1), Spain (2)*.

**First record**: Ca. 1829 - October (Gravenhorst (1829), Poland).

**Hibernacula**: DT (1), M (1), S (2).

**Sources**: Gravenhorst (1829) [*Ichneumon antennatorius*], Berthoumieu (1895b) [*Amblyteles equitatorius* Panz.]; Unpublished: Observation.org.

**Also mentioned in**: Morley (1903).

#### Orgichneumon calcatorius

(Thunberg, 1824)

0C3E6EB5-3AEA-5E71-83F4-A352B8225857

##### Notes

Not uncommon hibernating below bark, including the eastern Palaearctic (Riedel 2018). Similar to *Stenichneumon militarius* and *Ichneumon deliratorius*. However, the white colouration is more extensive, notably on the orbits and the mesopleuron. The apical segments of the metasoma do not have a white spot. Historical data are notably scarce, which translates to 10 new country records as hibernators (on 14 countries). One uncertain record exists from Switzerland (GBIF) on 20.XII.1990.

**Status**: Confirmed.

**Records**: Austria (10), Belarus (1)**, Belgium (2)*, Czechia (2)*, France (19), Germany (8)*, Hungary (1)**, Italy (2)**, Lithuania (1)**, Poland (1)*, Romania (3), Russia (3), Sweden (1)*, the Netherlands (1)*, Ukraine (1).

**First record**: 1951-1958 (Rasnitsyn (1959), Russia).

**Hibernacula**: DT (92), DTCL (1).

**Sources**: Constantineanu (1959) [*Stenichneumon calcatorius* Thunb.], Rasnitsyn (1959) [*S. calcatorius* Thunb.], Hinz and Kreissl (1992) and Hinz and Kreissl (1993), Valemberg and Vago (2013), Pénigot (2020), Varga et al. (2020); Unpublished: iNaturalist, Facebook, Observation.org, Pers. obs.

**Also mentioned in**: Rasnitsyn (1964).

#### Patrocloides ceaurei

(Heinrich, 1949)

16C89AED-EA4E-5CBF-B12F-ABD1AB4259B9

##### Notes

None.

**Status**: Confirmed.

**Records**: Germany, ca. 1999.

**Sources**: Bauer (1999).

**Also mentioned in**: Sebald et al. (2000).

#### Patrocloides dubitatorius

(Sulzer, 1776)

6995B75E-D35A-54EF-AE8B-35B173B7690F

##### Notes

There are additional uncertain records for Austria (GBIF).

**Status**: Confirmed.

**Records**: Austria (1), Germany (2), Russia (1).

**First record**: May 1947 or 1948 (Heinrich (1949b), Germany).

**Hibernacula**: DT (2), U (2).

**Sources**: Heinrich (1949b), Gokhman (1993), Hinz and Kreissl (1993), Sebald et al. (2000).

**Also mentioned in**: Gokhman (2003).

#### Patrocloides sputator

(Fabricius, 1793)

6EB7B607-4CE9-5105-B5F6-5E19CCF6F70C

##### Notes

Very similar to the common *Stenichneumon culpator*, but *P. sputator* lacks a tubercule on the hind coxa and is mostly confined to mountainous regions. Mentioned by Bauer (1984) as a hibernator, but not listed in his overview per hibernaculum.

**Status**: Confirmed.

**Records**: France (1), Germany (4), Romania (1).

**First record**: Ca. 1895 (Berthoumieu (1895b), France).

**Hibernacula**: DT (2), DTCL (16), M (1), U (2).

**Sources**: Berthoumieu (1895b) [*Amblyteles sputator* Fabr.], Lauterborn (1936) [*A. sputator*], Constantineanu (1959) [*A. sputator* Fabr.], Bauer (1984) [*Patroclus*], Hilpert (1987a), Schmidt and Zmudzinski (2005).

**Also mentioned in**: Morley (1903) [*Ctenichneumon sputator* Fab.], Bauer (1999).

#### Pseudoamblyteles homocerus

(Wesmael, 1854)

5B312D42-8C2E-5FF7-BB3F-8106D7B49BAD

##### Notes

There is one additional uncertain record from Germany, 9.I.1986 (GBIF) and one uncertain record from the United Kingdom, 16.II.2024 (Storey et al. 2025).

**Status**: Confirmed.

**Records**: Germany (1), Romania (3).

**First record**: May 1947 or 1948 (Heinrich (1949b), Germany).

**Hibernacula**: DT (2), DV (3), U (1).

**Sources**: Heinrich (1949b) [*Ctenamblyteles homocerus* Wesm.], Constantineanu (1959) [*C. homocerus* Wesm.].

**Also mentioned in**: Storey et al. (2025).

#### Pseudoplatylabus violentus

(Gravenhorst, 1829)

51D09978-12C4-5E13-ACB7-D479EDEAC890

##### Notes

Reported here for the first time as a hibernator. Both findings were made from the tussocks of *Molinia caerulea*.

**Status**: Confirmed*.

**Records**: The Netherlands (1), United Kingdom (1)**.

**First record**: 24.I.2022 (Observation.org, the Netherlands).

**Hibernacula**: LV (2).

**Sources**: Observation.org, Pers. obs.

#### Rhadinodonta flaviger

(Wesmael, 1845)

DF033B30-9056-59BF-8094-8A1D8CBBFE2A

##### Notes

With eight out of 13 countries newly reported as hibernating localities, this is another species with little historical data. It is moderately-sized and predominantly black, with the scutellum whitish laterally, but centrally black. This differs from *R. rufidens*, the other species in the genus in Europe, which has the scutellum completely black and the first three tergites red (Rasnitsyn and Siitan 1981).

**Status**: Confirmed.

**Records**: Austria (12), Belarus (1), France (15), Germany (3)*, Grand Duchy of Luxembourg (1)**, Hungary (3)*, Italy (1)** - Fig. 6c, Kosovo (1)**, Lithuania (1)**, Poland (1)*, Romania (2), Russia (2)*, Ukraine (1).

**First record**: 1915-1922 (Seyrig (1923), France).

**Hibernacula**: DT (56), DTCL (2), DV (1), LT (1), M (1).

**Sources**: Seyrig (1923) [*Anisobas flaviger* Wsm.], Hinz and Kreissl (1992) and Hinz and Kreissl (1993), Valemberg and Vago (2013), Lungu-Constantineanu and Constantineanu (2014), Varga et al. (2020); Unpublished: iNaturalist, Insecte.org, Observation.org, Pers. obs., Tereshkin (2014).

**Also mentioned in**: Tereshkin (2003).

#### Rhadinodonta rufidens

(Wesmael, 1845)

37C35499-480D-514B-B3C6-1D9A3C2ADCE1

##### Notes

None.

**Status**: Confirmed.

**Records**: France (1), Russia (2).

**First record**: 1951-1958 (Rasnitsyn (1959), Russia).

**Hibernacula**: DT (6), U (1).

**Sources**: Rasnitsyn (1959) and Rasnitsyn (1964) [*Ichneumon rufidens* Wesm.], Valemberg and Vago (2013).

#### Spilichneumon ammonius

(Gravenhorst, 1820)

4D13B577-7EBD-54AD-A1AD-113ED0FC042D

##### Notes

None.

**Status**: Confirmed.

**Records**: Germany (2), Russia (3).

**First record**: 1959-1964 (Rasnitsyn (1964), Russia).

**Hibernacula**: S (1), U (4).

**Sources**: Rasnitsyn (1964) [*Amblyteles ammonius* Grav.; *A. stagnicola* Thoms.], Bauer (1984); Unpublished: GBIF.

#### Spilichneumon celenae

Perkins, 1953

FBAE3F93-3B65-52F8-9680-3FE7C159F444

##### Notes

None.

**Status**: Confirmed*.

**Records**: Belarus, 3.XI.1987.

**Hibernacula**: DT (1).

**Sources**: Tereshkin (2014).

#### Spilichneumon johansoni

(Holmgren, 1871)

279915CD-F336-5799-BD3A-DF8014D49381

##### Notes

Similar to *S. occisorius*, but the subtegular ridge is black instead of white and the two first pair of lines of the apical metasomal segments have a different shape, narrowing laterally (Rasnitsyn and Siitan 1981).

**Status**: Confirmed.

**Records**: Germany (2), the Netherlands (1)*.

**First record**: May 1947 or 1948 (Heinrich (1949b), Germany).

**Hibernacula**: DT (1), U (2).

**Sources**: Heinrich (1949b), Sebald et al. (2000); Unpublished: Observation.org.

**Also mentioned in**: Bauer (2001).

#### Spilichneumon limnophilus

(Thomson, 1888)

6C8CC868-D99E-556A-A467-76F810448E7D

##### Notes

Sebald et al. (2000) include it, stating that the label in Bauer’s collection records the specimen as having been collected while overwintering in situ.

**Status**: Confirmed.

**Records**: Germany, 1980-2000.

**Sources**: Sebald et al. (2000).

**Also mentioned in**: Bauer (2001).

#### Spilichneumon occisorius

(Fabricius, 1793)

7ABFB3DC-7692-52E7-BDBB-543898B2CF88

##### Notes

Similar to *S. johansoni*.

**Status**: Confirmed.

**Records**: Belgium (2)*, France (1), Germany (2), Ukraine (1)*, United Kingdom (1).

**First record**: Ca. 1903 - April (Morley (1903), United Kingdom).

**Hibernacula**: DT (2), LV (1), M (2), S (2).

**Sources**: Morley (1903), Seyrig (1923) [*Spiloteles occisorius* F.], Bauer (2001); Unpublished: iNaturalist, Observation.org.

**Also mentioned in**: Constantineanu (1959) [*Amblyteles occisorius* Fabr.].

#### Spilichneumon podolicus

(Heinrich, 1936)

D52224B7-311E-5E9F-A41A-3B1695EA6E20

##### Notes

None.

**Status**: Confirmed.

**Records**: Germany, V.1947-1948.

**Sources**: Heinrich (1949b).

#### Spilothyrateles illuminatorius

(Gravenhorst, 1820)

3950B9D7-3471-5099-BC8F-4468887509AE

##### Notes

This species was only recently reported hibernating and seems to be expanding (Verheyde et al. 2021, Storey et al. 2025). Mostly found in grass tussocks, but occasionally also in evergreen trees, below stones or beneath the leaf rosettes of various plants, such as evening primrose and mullein (Observation.org).

**Status**: Confirmed.

**Records**: Belgium (1)*, Denmark (1)*, Germany (2), Italy (1)*, the Netherlands (4), Ukraine (1)*, United Kingdom (5).

**First record**: 1980-2000 (Sebald et al. (2000), Germany).

**Hibernacula**: DT (1), LT (1), LV (10), S (2), U (3).

**Sources**: Sebald et al. (2000), Schmidt and Zmudzinski (2005), Verheyde and Quicke (2022), Storey et al. (2025); Unpublished: iNaturalist, Observation.org, Pers. obs.

#### Spilothyrateles punctus

(Gravenhorst, 1829)

D6A2FE31-B8CC-5168-B823-E4F834EF2457

##### Notes

Similar to *S. nuptatorius* (not known to hibernate), but with a blackish scutellum and darker tergites, notably the first tergite (Rasnitsyn and Siitan 1981).

**Status**: Confirmed.

**Records**: Germany (2), Romania (1), Russia (2).

**First record**: 14.XI.1926 (Constantineanu (1929b), Romania).

**Hibernacula**: DT (1), DTCL (1), U (3).

**Sources**: Constantineanu (1929b) [*Amblyteles punctus* Grav.], Rasnitsyn (1964) [*A. punctus* Grav.], Bauer (1984) [*Ichneumon punctus* Grav.], Sebald et al. (2000).

**Also mentioned in**: Constantineanu (1959) [*Amblyteles punctus* Grav.].

#### Stenaoplus pictus

(Gravenhorst, 1829)

15767622-5778-5F8F-9446-8889E318EC4E

##### Notes

The context of the first record is unclear. The majority of the recent findings were from evergreen trees and shrubs. Its association with conifers and related ecology is well known (Tereshkin 2005).

**Status**: Confirmed.

**Records**: Germany (1), Norway (2)*, the Netherlands (4)*, United Kingdom (1)*.

**First record**: 1980-2000 (Sebald et al. (2000), Germany).

**Hibernacula**: DT (1), LT (4), LV (1), U (2).

**Sources**: Sebald et al. (2000); Unpublished: Artsobservasjoner, iNaturalist, Observation.org.

**Also mentioned in**: Tereshkin (2005).

#### Stenichneumon culpator

(Schrank, 1802)

4D407F59-F6D0-5F9B-BD72-9140EB6E2B09

##### Notes

Very similar to *Patrocloides sputator*, but with a tubercle on the hind coxa. Records (137) of 168 specimens could not be included as the essential features were not visible. One variety, var. adsentator, is common throughout Europe and has the abdomen completely black.

**Status**: Confirmed.

**Records**: Austria (6), Belarus (1)*, Belgium (342), Bulgaria (1), Croatia (1)**, Czechia (10)*, Denmark (28)**, Estonia (1)**, France (46), Germany (34), Grand Duchy of Luxembourg (1)*, Hungary (9), Ireland (1), Italy (1)*, Latvia (4)*, Lithuania (2)*, Norway (1)*, Poland (10)*, Romania (19), Russia (17), Sweden (6)*, the Netherlands (99), Ukraine (21), United Kingdom (39).

**First record**: Ca. 1871 (Tischbein (1871), Germany).

**Hibernacula**: DT (1326), DTCL (39), DV (2), LV (4), M (6), S (1), U (18).

**Sources**: Tischbein (1871) [*Ichneumon culpator* Schrank], Mocsáry (1885) [*I. culpator* Schrk.], Berthoumieu (1894) [*I. culpator* Schr.], Kriechbaumer (1899) [*I. culpator* Schr.], Habermehl (1916), Pic (1917) and Pic (1919) [*I. culpator* Sch.], Hancock (1923), Seyrig (1923) and Seyrig (1926a), Constantineanu (1929b), Heinrich (1951), Aerts (1957), Constantineanu (1959), Rasnitsyn (1959) and Rasnitsyn (1964), Györfi (1963), Constantineanu (1969), Valemberg and Vago (1974a)/Valemberg and Vago (1974b) and Valemberg and Vago (1975), Janssens (1976), Valemberg (1976a), Valemberg (1978) and Valemberg (1982), Bauer (1984), Gokhman (1985), Kolarov (1992), Hinz and Kreissl (1992) and Hinz and Kreissl (1993), Sebald et al. (2001), Schmidt and Zmudzinski (2005), Lungu-Constantineanu and Constantineanu (2014), Pénigot (2020), Varga et al. (2020), Verheyde and Quicke (2022); Unpublished: Arter DK, Artsobservasjoner, Coll. RBINS, Dabasdati, iNaturalist, Insecte.org, iRecord, Naturbasen, Naturgucker, Observation.org, Pers. obs., Pers. obs. (P.-N. Libert), UKRbin.

**Also mentioned in**: Tischbein (1873a) [*Ichneumon culpator* Schrank], Morley (1903), Gokhman (2003).

#### Stenichneumon inexpectatus

Heinrich, 1936

A2D03079-DB2E-5BAE-9F9E-48B1C55F11BD

##### Notes

One specimen was found hibernating in June, at a height of 1200 metres in the Alps.

**Status**: Confirmed.

**Records**: Germany, 25.V.1947.

**Sources**: Heinrich (1949b) [*Stenichneumon inexspectatus*].

#### Stenichneumon militarius

(Thunberg, 1824)

4456FDC9-7ABD-5818-97FA-81A4921E7190

##### Notes

Superficially similar to *Orgichneumon calcatorius*, but the white markings are less extensive. Remarkably common in Denmark compared to *S. culpator*, while this is usually vice-versa.

**Status**: Confirmed.

**Records**: Austria (20), Belgium (9), Denmark (43)**, France (23), Germany (14), Italy (1)*, Poland (2)*, Romania (1), the Netherlands (2)*, Ukraine (1)*.

**First record**: Ca. 1863 (Tischbein (1863), Germany).

**Hibernacula**: DT (214), DTCL (5), DV (1), M (7), S (1), U (14).

**Sources**: Tischbein (1863) [*Ichneumon pistorius* Gr.], Athimus (1901a) [*I. pistorius* Gr.], Pic (1917) [*I. pistorius* Grav.], Seyrig (1926a)/Seyrig (1926b), Heinrich (1949b) and Heinrich (1951), Renken (1956) [*I. militarius* Thunb.], Constantineanu (1959), Valemberg and Vago (1974a)/Valemberg and Vago (1974b) and Valemberg 1976a, Valemberg (1978) and Valemberg (1982), Bauer (1984), Hinz and Kreissl (1992) and Hinz and Kreissl (1993), Sebald et al. (2001), Schmidt and Zmudzinski (2005), Valemberg and Vago (2013), Verheyde and Quicke (2022); Unpublished: Coll. RBINS, iNaturalist, Insecte.org, Naturbasen, Naturgucker, Observation.org, Pers. obs.

**Also mentioned in**: Tischbein (1871) and Tischbein (1873a) [*Ichneumon pistorius* Gr.], Rasnitsyn (1964).

#### Syspasis albiguttata

(Gravenhorst, 1820)

0C059253-7B57-5D5E-B5A6-6D2891906F2A

##### Notes

Mostly found below bark. Incorrectly reported for Belgium in Verheyde and Quicke (2022).

**Status**: Confirmed.

**Records**: Austria (4), Czechia (1)*, France (8), Germany (5), Latvia (1)*, Lithuania (1)**, Poland (1), Romania (5), Russia (3), the Netherlands (1).

**First record**: 1870-1880 (Tischbein (1881), Germany).

**Hibernacula**: DT (38), DTCL (1), M (6).

**Sources**: Tischbein (1881) [*Amblyteles albostriatus* Tischb.], Seyrig (1923) and Seyrig (1926b) [*Stenichneumon multicinctus* Gr.], Heinrich (1927) [Coelichneumon multicinctus var. alboguttatus Grav.], Constantineanu (1929b) [*Ichneumon multicinctus* Grav.], Aerts (1957) [*I. alboguttatus* Gr.], Bauer (1984) [*Syspasis alboguttatus* Grav.], Gokhman (1985), Valemberg and Vago (2013), Verheyde and Quicke (2022); Unpublished: Dabasdati, iNaturalist, Pers. obs.

**Also mentioned in**: Constantineanu (1959) [*Stenichneumon alboguttatus* Grav.], Gokhman (2003).

#### Syspasis carinator

(Fabricius, 1798)

470726E4-D9BF-559C-BB56-9711A0D5ED45

##### Notes

Similar to *S. leucolomia*. One record from this complex exists from Spain (iNaturalist), consisting of three hibernating females.

**Status**: Confirmed.

**Records**: France (1), Germany (1), United Kingdom (2).

**First record**: Ca. 1894 (Berthoumieu 1894, France)

**Hibernacula**: LV (2), M (1), U (1).

**Sources**: Berthoumieu (1894) [*Ichneumon rufinus* Grav.], Bauer (1999) [*Syspasis helleri* Holmgr.]; Unpublished: Pers. obs.

**Also mentioned in**: Morley (1903) [*I. rufinus* Grav.], Sebald et al. (2000) [*S. helleri* Holmgr.].

#### Syspasis haesitator

Wesmael, 1845

B9C06ABD-B241-5144-AA56-46B195C4C777

##### Notes

Found hibernating below moss on a dead beech in Romania.

**Status**: Confirmed.

**Records**: Romania, ca. March 1959.

**Hibernacula**: DT (1).

**Sources**: Constantineanu (1959) [*Ichneumon haesitator* Wesm.].

#### Syspasis leucolomia

(Gravenhorst, 1829)

E1B81DA6-BC22-5446-9F1B-9D1BE16D179D

##### Notes

Similar to *S. carinator*. The recent observations are both from the southern part of France (iNaturalist).

**Status**: Confirmed.

**Records**: France (3).

**First record**: Ca. 1894 (Berthoumieu (1894), France)

**Hibernacula**: DT (8), LT (1), M (1).

**Sources**: Berthoumieu (1894) [*Ichneumon leucolomius* Grav.]; Unpublished: iNaturalist.

#### Syspasis lineator

(Fabricius, 1781)

178EB0C1-43D4-5DCE-8892-431C342CD900

##### Notes

One specimen was found under the bark of a living *Platanus* tree (Observation.org). This is certainly uncommon for larger Ichneumoninae. However, Morley (1903) also reports five specimens on a standing (dead) fir tree.

**Status**: Confirmed.

**Records**: Belgium (2)*, France (2), Germany (1), Russia (1), United Kingdom (4).

**First record**: Ca. 1903 (Morley (1903), United Kingdom).

**Hibernacula**: DT (11), LT (1), U (5).

**Sources**: Morley (1903) [*Stenichneumon trilineatus* Gmel.], Seyrig (1923) [*S. trilineatus* Gml.], Rasnitsyn (1964) [*S. trilineatus* Gmel.], Valemberg (1982) [*Stenichneumon lineator* Fab.]; Unpublished: Coll. RBINS, GBIF, Observation.org.

**Also mentioned in**: Constantineanu (1959) [*S. trilineatus* Gmel.].

#### Syspasis puerulus

(Kriechbaumer, 1890)

482A2A21-D9D1-57A5-BED5-284307DA93D0

##### Notes

Palaearctic (Riedel 2021a), but this species seems to be somewhat more common in (north)eastern Europe.

**Status**: Confirmed.

**Records**: Russia, 1951-1964 (2 records).

**Hibernacula**: DT (1), U (1).

**Sources**: Rasnitsyn (1959) and Rasnitsyn (1964) [*Stenichneumon puerulus* Kriechb.].

#### Syspasis scutellator

(Gravenhorst, 1829)

06E14093-0DF1-521F-AC8C-631DF99B4530

##### Notes

Very common species in south Germany, reaching up to 30% of the total hibernating fauna in the Black Forest Region (Hilpert 1987a).

**Status**: Confirmed.

**Records**: Belgium (1)*, Denmark (1)*, France (3), Germany (11), Romania (2), Russia (2), the Netherlands (2).

**First record**: 1915-1922 (Seyrig (1923), France).

**Hibernacula**: DT (216), DTCL (10), M (4), S (1), U (2).

**Sources**: Seyrig (1923) and Seyrig (1926b) [*Stenichneumon scutellator* Gr.], Constantineanu (1929a) [*Ichneumon scutellator* Grav.], Constantineanu (1959) [*S. scutellator* Grav.], Rasnitsyn (1959) [*S. scutellator* Grav.], Valemberg (1975) [*S. scutellator* Grav.], Bauer (1984) [*S. scutellator* Grav.], Hilpert (1987a), Schmidt and Zmudzinski (2005), Verheyde and Quicke (2022); Unpublished: iNaturalist, Naturbasen, Observation.org.

**Also mentioned in**: Rasnitsyn (1964) [*S. scutellator* Grav.].

#### Syspasis tauma

(Heinrich, 1951)

1047A22B-2B60-5AB1-B00C-AF586BD8FA8E

##### Notes

Uncommon species connected to highland regions. There is one uncertain record from Germany (GBIF), on 2.XII.1984, which is also very likely to be hibernating.

**Status**: Confirmed.

**Records**: Germany (2).

**First record**: 3.XII.1985 (Schmidt and Zmudzinski (2005), Germany).

**Hibernacula**: DT (1), U (1).

**Sources**: Schmidt and Zmudzinski (2005).

#### Thyrateles camelinus

Wesmael, 1845

9B39C5D4-EF4C-5FA0-AF7A-8203FCA46715

##### Notes

In contrast to *T. haereticus*, this species also occurs in lowlands near the Baltic and North Seas, although it is more common in mountainous regions (Bauer 1999). One uncertain record for Austria (GBIF). Mentioned in the descriptive part of Rasnitsyn (1959), but no original records were included.

**Status**: Confirmed.

**Records**: Belgium (4)*, Czechia (1)*, France (3), Germany (6), Latvia (1)**, Romania (4), Russia (1), the Netherlands (1)*, Ukraine (1)*.

**First record**: 1915-1922 (Seyrig (1923), France).

**Hibernacula**: B (1), DT (36), DTCL (11), M (3), U (2).

**Sources**: Seyrig (1923) and Seyrig (1926a) [*Dochyteles camelinus* Wsm.], Constantineanu (1959) [*Amblyteles camelinus* Wesm.], Valemberg (1975), Bauer (1984), Hilpert (1987a), Gokhman (1990a), Sebald et al. (2001), Schmidt and Zmudzinski (2005); Unpublished: Dabasdati, Observation.org, Pers. obs.

**Also mentioned in**: Rasnitsyn (1959) and Rasnitsyn (1964) [*Amblyteles camelinus* Wesm.], Bauer (1999), Gokhman (2003).

#### Thyrateles haereticus

(Wesmael, 1854)

93CFA249-B99B-5029-86C6-81676902C498

##### Notes

One uncertain record for Austria (GBIF).

**Status**: Confirmed.

**Records**: France (1), Germany (3), Romania (1).

**First record**: May 1947 or 1948 (Heinrich (1949b), Germany).

**Hibernacula**: DT (1), DTCL (1), U (3).

**Sources**: Heinrich (1949b) [*Ichneumon hereticus* Wesm.], Constantineanu (1969) [*Amblyteles binotatus* Kriechb.], Valemberg (1982) [*A. haereticus* Wesm.], Bauer (1984), Sebald et al. (2000).

**Also mentioned in**: Bauer (1999).

#### Triptognathops bicolor

(Kriechbaumer, 1882)

43230C14-AECB-5284-A7AD-0C4881B5B6C4

##### Notes

This species mainly occurs in Spain. It hibernates in caves (Selfa and Escolà 1991).

**Status**: Confirmed.

**Records**: Spain (8).

**First record**: 31.VII.1963 (Selfa and Escolà (1991), Spain).

**Hibernacula**: C (15).

**Sources**: Selfa and Escolà (1991).

#### Triptognathus atripes

(Gravenhorst, 1820)

59E1292B-1887-5E82-9BCA-685052FB6124

##### Notes

It was mentioned as occurring ‘below moss’ by Berthoumieu (1895b) and has only been confirmed by Bauer (2001), unfortunately without mentioning any original records.

**Status**: Confirmed.

**Records**: France (1), Germany (1).

**First record**: Ca. 1895 (Berthoumieu (1895b), France).

**Hibernacula**: M (1), U (1).

**Sources**: Berthoumieu (1895b) [Amblyteles uniguttatus var. atripes Grav.], Bauer (2001) [*Triptognathus uniguttatus* Grav.].

**Also mentioned in**: Morley (1903) [*A. uniguttatus* Grav.], Constantineanu (1959) [*A. uniguttatus* Grav.].

#### Triptognathus subalpinus

Heinrich, 1949

92650223-E672-5D30-9797-B904F2A79796

##### Notes

One specimen was found hibernating in May, at a height of 1200 metres in the Alps.

**Status**: Confirmed.

**Records**: Germany, VI.1947-1948.

**Sources**: Heinrich (1949b).

#### Triptognathus unifasciatus

(Spinola, 1843)

FB5C8367-4309-571A-847B-8E82AF598303

##### Notes

None.

**Status**: Confirmed.

**Records**: Spain, 4.VIII.1985.

**Hibernacula**: C (1).

**Sources**: Selfa and Escolà (1991) [*Triptognathops unidentatus* Berth.].

#### Ulesta perspicua

(Wesmael, 1857)

B44A0B40-7370-568A-8A36-7429255512C2

##### Notes

It is noteworthy that this species has no historic hibernation records, although it is widespread throughout Europe and fairly easy to recognise. Specialised in hibernating below tree bark.

**Status**: Confirmed

**Records**: Belgium (2)*, Czechia (1), France (2)*, Germany (1), Latvia (1)*, Russia (1)*.

**First record**: Ca. 1996-1997 (Sebald et al. (2001), Germany).

**Hibernacula**: DT (11).

**Sources**: Sebald et al. (2001), Valemberg and Fiala (2009); Unpublished: iNaturalist, Observation.org, Pers. obs.

**Also mentioned in**: Hinz (1991), Hinz and Kreissl (1993).

#### Virgichneumon callicerus

(Gravenhorst, 1820)

B2A54CC5-D8EA-57EC-8C7A-13D9F4F20C27

##### Notes

One specimen was found in the middle of the winter in grassland, both the hibernaculum and identification not being entirely clear.

**Status**: Unverified.

**Records**: Germany, 7.I.2024.

**Sources**: Observation.org.

#### Virgichneumon faunus

(Gravenhorst, 1829)

F878E836-E938-587F-8641-A75D3F626CAD

##### Notes

None.

**Status**: Confirmed.

**Records**: France (1), Romania (1).

**First record**: Ca. 1895 (Berthoumieu (1895a), France).

**Hibernacula**: M (1), U (1).

**Sources**: Berthoumieu (1895a) [*Ichneumon faunus* Grav.], Constantineanu (1959) [*Melanichneumon faunus* Grav.].

**Also mentioned in**: Morley (1903) [*M. faunus* Grav.].

#### Virgichneumon perversus

(Kriechbaumer, 1893)

B26F10D1-4645-54EE-BC33-42EFCD9A138F

##### Notes

The distribution of this species is unclear; it appears to be rare (GBIF). Rasnitsyn (1964) explicitly mentions it as a new hibernator, the specimen being found in the region of Moscow.

**Status**: Confirmed.

**Records**: Russia, 1959-1964.

**Sources**: Rasnitsyn (1964) [*Cratichneumon perversus* Kriechb.].

#### Virgichneumon tergenus

(Gravenhorst, 1820)

FC4B56D9-57CF-5AC5-B06E-BCC5705FC797

##### Notes

Only recently reported as hibernating by Pénigot (2020) in a stem of *Carex pendula*. Several records from the Netherlands, but some of them are uncertain as this species is similar to other *Barichneumon* and *Virgichneumon* spp. (esp. *B. derogator*). All specimens were found in moss, litter or in evergreen trees.

**Status**: Confirmed.

**Records**: France (1), the Netherlands (3)*.

**First record**: 5.I.2020 (Pénigot (2020), France) (unpublished, 30.I.2019 – the Netherlands).

**Hibernacula**: DV (1), LT (1), LV (1), M (1).

**Sources**: Pénigot (2020); Unpublished: Observation.org.

#### Vulgichneumon bimaculatus

(Schrank, 1776)

73125555-370D-5310-B300-145A6F1BB41A

##### Notes

As the ecology of this genus is not yet fully understood, more data are required.

**Status**: Unverified.

**Records**: France, ca. 1895.

**Sources**: Berthoumieu (1895a) [*Ichneumon bimaculatorius* Panz.].

**Also mentioned in**: Morley (1903) [*Melanichneumon bimaculatorius* Panz.], Constantineanu (1959) [*M. bimaculatus* Schrank].

#### Vulgichneumon deceptor

(Scopoli, 1763)

00BB1530-7C5E-5D63-A174-E7C59DABAC05

##### Notes

Schmidt and Zmudzinski (2005) mention one specimen, hibernating in November. One uncertain record from Belgium, 1.I.1924 (coll. RBINS).

**Status**: Confirmed.

**Records**: France (1), Germany (1).

**First record**: Ca. 1895 (Berthoumieu (1895a), France).

**Hibernacula**: M (1), U (1).

**Sources**: Berthoumieu (1895a) [*Ichneumon deceptor* Grav.], Schmidt and Zmudzinski (2005).

**Also mentioned in**: Morley (1903) [*Barichneumon vestigator* Wesm.], Constantineanu (1959) [*B. deceptor* Grav.].

#### Vulgichneumon saturatorius

(Linnaeus, 1758)

F990F653-6228-500F-8811-BF7FB481C6D3

##### Notes

None.

**Status**: Unverified.

**Records**: France, ca. 1895.

**Sources**: Berthoumieu (1895a) [*Ichneumon saturatorius* L.].

**Also mentioned in**: Morley (1903) [*Melanichneumon saturatorius* Linn.], Constantineanu (1959) [*M. saturatorius* Linn.].

#### Vulgichneumon suavis

(Gravenhorst, 1820)

83FE600F-C343-5538-877B-B09500DC5C27

##### Notes

Similar to *V. trifarius*. Differences are only related to the frons and collecting specimens is necessary (Rasnitsyn and Siitan 1981). Schmidt and Zmudzinski (2005) report it as hibernating in November and December. Two uncertain records from Norway (GBIF) and the Netherlands (either this species or *V. trifarius*) exist, the latter one collected by beating conifers.

**Status**: Confirmed.

**Records**: Germany, 1980-2006 (2 records).

**Sources**: Schmidt and Zmudzinski (2005).

#### Zanthojoppa lutea

(Gravenhorst, 1829)

F8BEE1A5-450E-5AEF-B9A7-DB74F0C2A99E

##### Notes

Specimens can have entirely orange hind legs or black(ish) legs. Not uncommon on dead trees in (north)western Europe, notably in Belgium and France.

**Status**: Confirmed.

**Records**: Austria (2), Belgium (34), Denmark (4)**, France (14), Germany (4), Grand Duchy of Luxembourg (1)**, Hungary (1)*, Poland (2), Romania (2), Russia (3), the Netherlands (4).

**First record**: Ca. 1894 (Berthoumieu (1894), France).

**Hibernacula**: DT (146), DTCL (1), M (3), S (1), U (2)

**Sources**: Berthoumieu (1894) [*Hoplismenus luteus* Grav.], Seyrig (1923) [*H. luteus* Gr.], Heinrich 1928 [*H. luteus* Grav.], Constantineanu (1929b) [*H. luteus* Grav.], Rasnitsyn (1959) [*H. luteus* Grav.], Bauer (1984) [*H. luteus* Grav.], Hinz and Kreissl (1992), Sebald et al. (2001), Valemberg and Vago (2013), Verheyde and Quicke (2022); Unpublished: Coll. RBINS, iNaturalist, Insecte.org, Facebook (Hymenopterists Forum), Observation.org, Pers. obs.

**Also mentioned in**: Constantineanu (1959) [*H. luteus* Grav.], Rasnitsyn (1964) [*H. luteus* Grav.].

#### Phaeogenini

69DD5B6E-2ED6-5DA1-821E-2C71C0E40A3F

##### Notes

Very much like Ichneumonini, some genera are probably obligate hibernators (e.g. *Aethecerus*, *Dicaelotus*, *Heterischnus*), while, for some other genera, the situation remains unclear. It is noteworthy, for example, that there are very few recent records of *Phaeogenes* spp., although several species are fairly easy to recognise.

#### Aethecerus discolor

Wesmael, 1845

27380F80-4215-5092-AF36-8BFCACB7E19F

##### Notes

Additionally, two uncertain records from Norway (pers. obs.).

**Status**: Confirmed.

**Records**: France (1), Russia (1).

**First record**: Ca. 1896 (Berthoumieu (1896), France).

**Sources**: Berthoumieu (1896), Gokhman (1988).

**Hibernacula**: LV (1), U (1).

**Also mentioned in**: Morley (1903).

#### Aethecerus dispar

Wesmael, 1845

39E8DC26-6CD4-5C55-8A67-6B235EBC36EE

##### Notes

None.

**Status**: Confirmed.

**Records**: France (1), United Kingdom (1).

**First record**: Ca. 1896 (Berthoumieu (1896), France).

**Hibernacula**: LV (1), M (1).

**Sources**: Berthoumieu (1896), Morley (1903).

#### Aethecerus nitidus

Wesmael, 1845

C914D591-86FF-55A6-B9F7-913DBB564382

##### Notes

This species had not been reported hibernating for over 160 years, but recently a specimen was beaten from branches in a pine tree, which had fallen off.

**Status**: Confirmed.

**Records**: Germany (1), the Netherlands (1)*.

**First record**: Ca. 1863 (Germany, Tischbein (1863)).

**Hibernacula**: LT (1), U (1)

**Sources**: Tischbein (1863); Unpublished: Observation.org.

**Also mentioned in**: Tischbein (1871).

#### Aethecerus ranini

Gokhman, 1991

DDAE2E80-6238-5233-B9E7-6A72572319FD

##### Notes

Most specimens were found hibernating in the empty stems of Apiaceae.

**Status**: Confirmed.

**Records**: Finland (1), Russia (1).

**First record**: 9.V.1981 (Russia, Gokhman (1991)).

**Hibernacula**: LV (42).

**Sources**: Gokhman (1991).

**Also mentioned in**: Gokhman (2003).

#### Auberteterus alternecoloratus

(Cushman, 1929)

C59CCEC6-3242-548D-A671-7D45F2A6CE9C

##### Notes

Hibernation in lab conditions had already been proved by Chacko and Rao (1966), but this was only recently confirmed in the field in Belgium, from a stem of *Heracleum sphondylium*.

**Status**: Confirmed.

**Records**: Belgium, 30.III.2021.

**Hibernacula**: LV (1).

**Sources**: Verheyde and Quicke (2022).

**Also mentioned in**: Chacko and Rao (1966) [*Centeterus alternecoloratus* Cushman?].

#### Baeosemus mitigosus

(Gravenhorst, 1829)

F5DC079F-4713-5638-9EBE-D605863A454D

##### Notes

One record of *Baeosemus* sp. is mentioned for Germany in Bauer (1984) on grass tussocks.

**Status**: Confirmed

**Records**: France (2), Germany (1), Romania (3).

**First record**: Ca. 1871 (Tischbein (1871), Germany).

**Hibernacula**: DT (2), LT (2), M (2).

**Sources**: Tischbein (1871) [*Herpestomus phaeocerus* W.], Berthoumieu (1896) [*H. phaeocerus* Wesm.], Seyrig (1923) [*H. phaeocerus* Wsm.], Constantineanu (1929a) [*H. phaeocerus* Wesm.], Constantineanu (1970) [*H. phaeocerus* Wesm.].

**Also mentioned in**: Bauer (1984).

#### Centeterus confector

(Gravenhorst, 1829)

27B6F249-A83A-58A4-A132-6C37D3B46DED

##### Notes

Similar to *C. rubiginosus*, but the temples are narrower than the eyes and specimens often (not always) have a white band on the antennae.

**Status**: Confirmed.

**Records**: Belgium (2), France (1), the Netherlands (1), United Kingdom (1)*.

**First record**: 1915-1922 (Seyrig (1923), France).

**Hibernacula**: DT (2), DV (1), LV (1), M (1).

**Sources**: Seyrig (1923), Verheyde and Quicke (2022); Unpublished: Coll. RBINS, iRecord.

#### Centeterus rubiginosus

(Gmelin, 1790)

D08F0692-C4B6-5F65-9C7E-87081C3E5882

##### Notes

Similar to *C. confector*.

**Status**: Confirmed.

**Records**: Ireland (1), United Kingdom (7).

**First record**: Ca. 1903 (Morley (1903), United Kingdom).

**Hibernacula**: DV (1), LV (7), M (1).

**Sources**: Morley (1903) [*Centeterus opprimator* Grav.], Johnson (1920) [*C. opprimator* Gr.], Hancock (1923) [*C. opprimator* Grav.]; Unpublished: Pers. obs.

#### Colpognathus celerator

(Gravenhorst, 1807)

177662F1-BE8C-5764-8503-5B85FDC612C2

##### Notes

Very similar to *C. divisus*, but the mandible and area superomedia are shaped differently (Perkins 1960). Several records could only be identified as species complex, with additional data for Belgium and the Netherlands. Erroneously reported at species level by Verheyde and Quicke (2022) for the Netherlands.

**Status**: Confirmed.

**Records**: Belgium (6)*, France (6), Germany (1), Hungary (1), Poland (1), Russia (1), United Kingdom (7)*.

**First record**: Ca. 23.XII.1885 (Mocsáry (1885), Hungary).

**Hibernacula**: DT (1), DV (7), LV (15), M (1), U (3).

**Sources**: Mocsáry (1885), Seyrig (1923), Hedwig (1927), Heinrich (1949a), Rasnitsyn (1964), Valemberg (1976b) Pénigot (2020); Unpublished: Coll. RBINS, iRecord, Pers. obs.

**Also mentioned in**: Verheyde and Quicke (2022).

#### Colpognathus divisus

Thomson, 1891

4693431C-9DFC-50B5-9C31-22BB22B493D8

##### Notes

Similar to *C. celerator*. One of the common species in grass tussocks in the United Kingdom.

**Status**: Confirmed.

**Records**: France (3), Germany (3), Ireland (1), Poland (1), United Kingdom (22).

**First record**: 23.III.1914 (Torka (1915), Poland).

**Hibernacula**: LV (48), M (2), U (1).

**Sources**: Torka (1915), Johnson (1920), Seyrig (1923), Hancock (1923) and Hancock (1925), Heinrich (1949a) and Heinrich (1951), Valemberg (1976b), Bauer (1984); Unpublished: GBIF, Pers. obs.

**Also mentioned in**: Morley (1903).

#### Diadromus arrisor

Wesmael, 1845

37E95905-5D6A-55FD-9E88-45D50D62D418

##### Notes

Both specimens from Romania were found near living trees, one in the moss bed at the foot of a tree, the second one between the cracks of tree bark. The specimen from Belgium was beaten from ivy. Two additional uncertain records for Finland on 6.XII.1942 and for the United Kingdom on 13.I.1979 (GBIF).

**Status**: Confirmed.

**Records**: Belgium (1)*, Romania (2).

**First record**: 24.XI.1926 (Constantineanu (1929b), Romania).

**Hibernacula**: LT (1), LV (1), M (1).

**Sources**: Constantineanu (1929b), Constantineanu (1970); Unpublished: Observation.org.

#### Diadromus candidatus

(Gravenhorst, 1829)

F315E91D-0932-5BF7-B6BA-D9C32B9F2C81

##### Notes

Sebald et al. (2000) include it, stating that the label in Bauer’s collection records the specimen as having been collected while overwintering in situ. Schmidt and Zmudzinski (2005) additionally report specimens late in the year, but without hibernation context.

**Status**: Confirmed.

**Records**: Germany, 1980-2000.

**Sources**: Sebald et al. (2000).

#### Diadromus collaris

(Gravenhorst, 1829)

E612B177-E872-5E2D-A112-3D1CAF94AE2A

##### Notes

None.

**Status**: Confirmed.

**Records**: Belgium (1)*, Denmark (1)**, France (1), Germany (1), Norway (2)*, the Netherlands (1), United Kingdom (5)*.

**First record**: I.1976 (Valemberg (1976a), France).

**Hibernacula**: DT (1), DV (2), LT (1), LV (8), U (1).

**Sources**: Valemberg (1976a) [*Thyraeella collaris* Grav.], Schmidt and Zmudzinski (2005), Verheyde and Quicke (2022); Unpublished: iNaturalist, Naturbasen, Observation.org, Pers. obs.

**Also mentioned in**: Valemberg (1978).

#### Diadromus heteroneurus

Holmgren, 1890

220B342C-EF87-5DF2-BE22-C5A47E23522E

##### Notes

None.

**Status**: Confirmed.

**Records**: Germany, 31.I.1987.

**Hibernacula**: DT (1).

**Sources**: Schmidt and Zmudzinski (2005).

#### Diadromus intermedius

Wesmael, 1845

EC3FA7AD-27D6-5AFC-88BC-93F57F8AF334

##### Notes

First reported by Rasnitsyn (1964) from the Moscow region. Bauer (2001) mentions this species as hibernating, but it is unclear if this is based on additional records.

**Status**: Confirmed.

**Records**: Russia, 1959-1964.

**Sources**: Rasnitsyn (1964).

**Also mentioned in**: Bauer (2001).

#### Diadromus pulchellus

Wesmael, 1845

D5378DB3-4D15-5CCD-BA45-65EDF1F2A066

##### Notes

Found while sieving.

**Status**: Confirmed*.

**Records**: Norway (2).

**First record**: 18.II.2015 (Pers. obs. - G. Engan, Norway).

**Hibernacula**: DV (2).

**Sources**: Pers. obs.

#### Diadromus subtilicornis

(Gravenhorst, 1829)

3AF65D4D-F73E-5AD8-9B70-87786D61E416

##### Notes

Recently found in the stem of *Carex paniculata*.

**Status**: Confirmed.

**Records**: Russia (1), United Kingdom (1)*.

**First record**: Around 1929 (Telenga (1929), Russia).

**Hibernacula**: DV (1), LV (1).

**Sources**: Telenga (1929); Unpublished: Pers. obs.

**Also mentioned in**: Gokhman (1988).

#### Diadromus troglodytes

(Gravenhorst, 1829)

45E25E63-83AB-5F8D-818C-2F94803B351F

##### Notes

Sebald et al. (2000) include it, but this was most likely based solely on the collection date.

**Status**: Confirmed.

**Records**: Norway (1)*, Romania (1), the Netherlands (2)*, United Kingdom (8).

**First record**: II.1894 (Morley (1903), United Kingdom).

**Hibernacula**: DV (3), LT (4), LV (4), M (1).

**Sources**: Morley (1903), Lungu-Constantineanu and Constantineanu (2014), Diller and Shaw (2014); Unpublished: Pers. obs.

**Also mentioned in**: Sebald et al. (2000).

#### Dicaelotus cameroni

Bridgman, 1881

463654B9-CED7-5C17-9D2C-45A4EB5B5846

##### Notes

Found in moss, at a height of 950 metres in Norway.

**Status**: Confirmed*.

**Records**: Norway, 12.IX.2021.

**Hibernacula**: M (2).

**Sources**: Artsobservasjoner.

#### Dicaelotus erythrogaster

(Holmgren, 1890)

0558B134-F027-52D0-8360-97CF0D8D6A13

##### Notes

Very similar to *D. montanus* and *D. pudibundus* and, therefore, considered as unverified. The first record from Romania mentions 16 specimens in the cracks of an old oak, covered with moss. The second report consisted of an individual specimen found on a stone covered with moss.

**Status**: Unverified.

**Records**: Romania (2).

**First record**: 14.XII.1964 (Constantineanu (1970), Romania).

**Hibernacula**: LT (16), S (1).

**Sources**: Constantineanu (1970) [*Cinxaelotus erythrogaster* Holmgr.], Lungu-Constantineanu and Constantineanu (2014) [*C. erythrogaster* Holmgr.].

#### Dicaelotus montanus

(de Stefani, 1885)

7D2D37FD-D84A-51DD-BD71-9D877E38B369

##### Notes

Very similar to *D. pudibundus*. Important characteristics are the meso- and metapleura which are red-marked (black in *D. pudibundus*) and the propodeum which has distinct horizontal and vertical parts (conspicuously rounded in *D. pudibundus*). The first specimen ever reported as a hibernator was found on an evergreen tree. There are additional uncertain records from Belgium, mainly specimens from ivy.

**Status**: Confirmed.

**Records**: Belgium (3), the Netherlands (2)*.

**First record**: 24.II.2021 (Observation.org, Belgium).

**Hibernacula**: LT (1), LV (4).

**Sources**: Verheyde and Quicke (2022); Unpublished: Observation.org.

#### Dicaelotus pictus

(Schmiedeknecht, 1903)

3001ACA7-20A6-5ADA-BFE1-142EB553D0E0

##### Notes

Solely found between the cracks and bark of living trees, notably *Platanus* and *Tilia* sp.

**Status**: Confirmed.

**Records**: Belgium (1), Romania (3).

**First record**: 1964-1967 (Constantineanu (1970), Romania).

**Hibernacula**: LT (4).

**Sources**: Constantineanu (1970) [*Deloglyptus pictus* Schm.], Lungu-Constantineanu and Constantineanu (2014) [*D. pictus* Schm.], Verheyde and Quicke (2022).

#### Dicaelotus pumilus

(Gravenhorst, 1829)

19EA1D4F-EF74-5E62-A377-29EF23F6C7B4

##### Notes

None.

**Status**: Confirmed.

**Records**: Belgium (1)*, France (1), Ireland (2), Poland (1), United Kingdom (10).

**First record**: 1890-1920 (Johnson (1920), Ireland).

**Hibernacula**: DT (1), DV (1), LT (1), LV (12), M (3), U (1).

**Sources**: Morley (1903), Johnson (1920), Seyrig (1923), Hedwig (1927), Storey et al. (2025); Unpublished: Coll. RBINS; Pers. obs.

#### Dicaelotus ruficoxatus

(Gravenhorst, 1829)

A9F9FDA9-7C04-587D-A416-6BF43EBF36E1

##### Notes

None.

**Status**: Confirmed.

**Records**: Belgium (1)*, Germany (1), United Kingdom (4)*.

**First record**: Ca. 1863 (Tischbein (1863), Germany).

**Hibernacula**: DT (1), DV (1), LV (10).

**Sources**: Tischbein (1863) [*Dicoelotus unipunctatus* W.]; Unpublished: Coll. RBINS; Pers. obs.

**Also mentioned in**: Tischbein (1871) [*Dicaelotus unipunctatus* W.].

#### Dicaelotus rufilimbatus

(Gravenhorst, 1820)

05BAEC30-44FD-5A03-B392-9FA2C96344DF

##### Notes

None.

**Status**: Confirmed.

**Records**: Ireland, 1890-1920 (2 records)

**Hibernacula**: M (2).

**Sources**: Johnson (1920).

#### Dicaelotus signatus

Roman, 1939

10729335-DF9F-5D3B-86E7-2902F2AA4B8A

##### Notes

None.

**Status**: Confirmed.

**Records**: Belgium, 21.III.1937.

**Hibernacula**: DT (1).

**Sources**: Leclercq (1942).

#### Dilleritomus filiformis

(Strobl, 1901)

0F46252A-38EB-5D91-A510-412D45657A31

##### Notes

Found dangling in the skirt of a tussock of *Carex paniculata*.

**Status**: Confirmed*.

**Records**: United Kingdom, 21.III.2021.

**Hibernacula**: LV (1).

**Sources**: Pers. obs.

#### Dirophanes callopus

(Wesmael, 1845)

1CAC3BBD-81AA-59C1-B113-A49F91B7C0D7

##### Notes

Beaten from *Quercus ilex* in the United Kingdom. The species probably also hibernates in tree cracks, as it has been observed in the vicinity of these microhabitats quite often very early or late in the year in, for example, Belgium and the Netherlands.

**Status**: Confirmed*.

**Records**: United Kingdom, 21.XI.2021.

**Hibernacula**: LT (1).

**Sources**: iNaturalist.

#### Dirophanes coryphaeus

(Wesmael, 1845)

61EC8EA8-5440-5330-A0FC-42770049FA4E

##### Notes

Found while sieving.

**Status**: Confirmed*.

**Records**: Norway, 1.IV.2018.

**Hibernacula**: DV (1).

**Sources**: Pers. obs.

#### Dirophanes fulvitarsis

(Wesmael, 1845)

DC7F99AB-76A2-531B-92FE-65FF8E726D8F

##### Notes

Only recently reported as hibernating by Pénigot (2020) in a stem of *Carex pendula*. This was confirmed three years later by a second finding in the United Kingdom, this time in a stem of *Juncus inflexus*.

**Status**: Confirmed.

**Records**: France (1), United Kingdom (1)*.

**First record**: 25.I.2020 (Pénigot (2020), France).

**Hibernacula**: LV (3).

**Sources**: Pénigot (2020); Unpublished: Pers. obs.

**Also mentioned in**: Valemberg (1978).

#### Dirophanes invisor

(Thunberg, 1824)

D1798B75-11FC-58D6-9C42-57291EE4FF1D

##### Notes

Well studied parasitoid of the green oak-leaf roller moth *Tortrix viridana*. Morley (1903) already thought females hibernated. Cole (Cole 1959, Cole 1967) and Bogenschütz (1965) demonstrated how females indeed hibernate, using phenology and lab conditions. Cole (1967) projected that the females survive in litter below oaks. This has been confirmed now by both Lungu-Constantineanu and Constantineanu (2014) and Diller and Shaw (2014).

**Status**: Confirmed.

**Records**: Romania (2), Russia (1), United Kingdom (2).

**First record**: 1951-1958 (Rasnitsyn (1959), Russia).

**Hibernacula**: DV (3), DT (1), LV (1).

**Sources**: Rasnitsyn (1959) [*Phaeogenes stimulator* Grav.], Cole (1967) [*P. invisor* Thunb.], Lungu-Constantineanu and Constantineanu (2014) [*P. invisor* Thunb.], Diller and Shaw (2014).

**Also mentioned in**: Morley (1903) [*P. stimulator* Grav.], Cole (1959) [*P. invisor* Thunb.], Rasnitsyn (1964) [*P. stimulator*], Bogenschütz (1965) [*P. invisor* Thunb.].

#### Dirophanes maculicornis

(Stephens, 1835)

505BA0C7-F9D6-5783-99FA-1B53CFF306FD

##### Notes

None.

**Status**: Confirmed.

**Records**: France (14), Germany (5), Norway (1)*, the Netherlands (1)*, Ukraine (1), United Kingdom (3).

**First record**: Ca. 1871 (Tischbein (1871), Germany).

**Hibernacula**: DT (15), LT (2), LV (1), M (26), U (1).

**Sources**: Tischbein (1871) [*Phaeogenes scutellaris* W.], Morley (1903) [*P. maculicornis* Steph.], Seyrig (1923) [*P. scutellaris* Wsm.], Heinrich (1949a) [*P. scutellaris* Wesm.], Starke (1956) [*P. scutellaris* Wesm.], Valemberg and Vago (1974a), Valemberg and Vago 1975, Valemberg 1976a/Valemberg 1976b [*P. maculicornis* Wesm.], Bauer (1984) [*P. maculicornis* Steph.], Schmidt and Zmudzinski (2005), Valemberg and Vago (2013), Varga et al. (2020); Unpublished: iRecord, Observation.org, Pers. obs. (H. Haraldseide).

#### Dirophanes regenerator

(Fabricius, 1804)

F7F4F562-94D9-5A0D-9900-E608C6C0C36B

##### Notes

None.

**Status**: Confirmed.

**Records**: France (1), Ireland (1), United Kingdom (5).

**First record**: Ca. 1903 (Morley (1903), United Kingdom).

**Hibernacula**: LV (7), M (1), U (1).

**Sources**: Morley (1903) [*Phaeogenes rusticatus* Wesm.], Johnson (1920) [*P. rusticatus* Wesm.], Seyrig (1923) [*P. rusticatus* Wsm.]; Unpublished: Pers. obs.

#### Eparces grandiceps

(Thomson, 1891)

B93509E9-F68F-5884-944E-05A7570720B1

##### Notes

None.

**Status**: Confirmed.

**Records**: Germany (2).

**First record**: Ca. 1984 (Bauer (1984), Germany).

**Hibernacula**: LV (1), U (1).

**Sources**: Bauer (1984), Sebald et al. (2000).

#### Epitomus infuscatus

(Gravenhorst, 1829)

15354C90-4534-5DC1-9847-4BCA8B385016

##### Notes

None.

**Status**: Confirmed.

**Records**: France (2), Norway (1)*, United Kingdom (4)*.

**First record**: 3.II.1974 (Valemberg and Vago (1974a), France).

**Hibernacula**: DT (1), DV (1), LV (11).

**Sources**: Valemberg and Vago (1974a)/Valemberg and Vago (1974b) [*Epitomus pygmaeus* Brischke]; Unpublished: Pers. obs.

#### Epitomus proximus

Perkins, 1953

46969E43-4807-5ECC-B992-D914838F301F

##### Notes

None.

**Status**: Confirmed.

**Records**: Romania, 1964-1967.

**Hibernacula**: LT (1).

**Sources**: Constantineanu (1970).

#### Eriplatys ardeicollis

(Wesmael, 1845)

9778D43C-BD24-5D07-B96F-352242026C86

##### Notes

Two uncertain records for Sweden (GBIF). It is unclear if the specimens were found hibernating, although the months December and January strongly suggest this.

**Status**: Confirmed.

**Records**: Germany (2), Romania (1), United Kingdom (1).

**First record**: 16.II.1899 (Morley (1903), United Kingdom).

**Hibernacula**: DT (1), LT (2), U (1).

**Sources**: Morley (1903) [*Melanomicrus Elliotti*], Constantineanu (1970), Bauer (1984), Sebald et al. (2000).

#### Herpestomus arridens

(Gravenhorst, 1829)

5917CBAA-FBD6-5958-93AE-F23E110F12C9

##### Notes

None.

**Status**: Confirmed.

**Records**: Germany (3).

**First record**: Ca. 1863 (Tischbein (1863), Germany).

**Hibernacula**: DT (2), LV (1).

**Sources**: Tischbein (1863) [*Herpestomus facialis* Gr.], Bauer (1984) [*H. xanthops* Grav.].

**Also mentioned in**: Tischbein (1871).

#### Herpestomus brunnicornis

(Gravenhorst, 1829)

41DEC242-ED10-599E-BF5A-8C1889064083

##### Notes

Leclercq (1953) mentions he caught a specimen in October 1944 which stayed alive during winter.

**Status**: Confirmed.

**Records**: Romania (1), the Netherlands (2).

**First record**: 2006-2010 (Lungu-Constantineanu and Constantineanu 2014, Romania).

**Hibernacula**: DV (1), LT (1), LV (1).

**Sources**: Lungu-Constantineanu and Constantineanu (2014), Verheyde and Quicke (2022); Unpublished: Observation.org.

**Also mentioned in**: Morley (1903), Leclercq (1953).

#### Herpestomus minimus

(Berthoumieu, 1901)

FF2BB3AD-D289-50C8-9D36-AF538364335F

##### Notes

This newly-reported hibernator was found below leaf litter at Mount Salviano in Italy.

**Status**: Confirmed*.

**Records**: Italy, 8.I.2025.

**Hibernacula**: DV (1).

**Sources**: iNaturalist.

#### Herpestomus nasutus

Wesmael, 1845

776F065E-FBEE-5CDF-B5E9-165310E514B7

##### Notes

The sole findings by Seyrig (1923)were from moss.

**Status**: Confirmed.

**Records**: France, 1915-1922.

**Hibernacula**: M (1).

**Sources**: Seyrig (1923) [*Herpestomus furunculus* Wsm.].

#### Heterischnus coxator

(Thomson, 1891)

ECA87378-44DD-5863-9D45-FAE0B9B89D92

##### Notes

One additional specimen is reported as found dead below bark, in June (Schmidt and Zmudzinski 2005). The species is also listed in Sebald et al. (2000), but this was based on the collection date only.

**Status**: Confirmed.

**Records**: Germany, 1980-2006.

**Hibernacula**: DT (6).

**Sources**: Schmidt and Zmudzinski (2005).

**Also mentioned in**: Sebald et al. (2000).

#### Heterischnus debilis

(Gravenhorst, 1829)

C86292EF-9658-537F-B2DA-160CEAEB969B

##### Notes

One specimen is reported as found dead below bark, in June 1985 (Schmidt and Zmudzinski 2005).

**Status**: Unverified.

**Records**: Germany, 5.VI.1985.

**Sources**: Schmidt and Zmudzinski (2005).

#### Heterischnus nigricollis

(Wesmael, 1845)

0403F55B-695D-501C-9021-6FC72A3F8B96

##### Notes

Most specimens were found hibernating in the empty stems of Apiaceae.

**Status**: Confirmed.

**Records**: France (4), Germany (2), Russia (1).

**First record**: Ca. 1896 (Berthoumieu (1896), France).

**Hibernacula**: DT (5), LV (12), M (3), U (1).

**Sources**: Berthoumieu (1896) [*Ischnus rufipes* Wesm.], Morley (1903) [*Heterischnus rufipes* Steph.], Seyrig (1923) [*H. rufipes* Wsm.], Hilpert (1987a), Gokhman (1988).

**Also mentioned in**: Gokhman (1990a), Gokhman (2003).

#### Heterischnus pulchellus

(Thomson, 1891)

FF72400C-BB2D-56B1-AF65-5955A0B13DC9

##### Notes

This species is also listed in Sebald et al. (2000), but this was based on the collection date only.

**Status**: Confirmed*.

**Records**: France, 15.I.2023 - Fig. 6d.

**Hibernacula**: LV (1).

**Sources**: iNaturalist.

**Also mentioned in**: Sebald et al. (2000).

#### Heterischnus pulex

(Muller, 1776)

7911CF65-DC9F-5140-81AE-A936D2034249

##### Notes

There is one additional record from a dead tree in Norway (Artsobservasjoner). Unfortunately, the typical pectinate claws for this species are not visible on the available pictures.

**Status**: Confirmed.

**Records**: Germany (2), Romania (1).

**First record**: Ca. 1871 (Tischbein (1871), Germany).

**Hibernacula**: DT (5).

**Sources**: Tischbein (1871), Constantineanu (1929b), Schmidt and Zmudzinski (2005).

#### Heterischnus truncator

(Fabricius, 1798)

03532A11-9EA7-54B6-B4DE-FE9D2E886A4B

##### Notes

The mesosoma very much varies in colour, from entirely red to black (Valemberg 2014). Uses human infrastructure to hibernate (e.g. fences, information panels), but also living trees (*Platanus* sp.) or dead trees.

**Status**: Confirmed.

**Records**: Belgium (9), Denmark (1)**, France (3), Germany (3), Romania (2), Russia (4), the Netherlands (7).

**First record**: Ca. 1829 (Gravenhorst (1829), Germany).

**Hibernacula**: DT (25), LT (5), LV (2), M (1), O (6), U (3).

**Sources**: Gravenhorst (1829) [*Ischnus truncator* Fabr.], Berthoumieu (1896) [*I. truncator* Fabr.], Seyrig (1923) [*I. truncator* F.], Constantineanu (1929b) [*I. truncator* Fabr.], Leclercq (1953) [*Ischnopsidea truncator* Fabr.], Hilpert (1987a), Gokhman (1988), Lungu-Constantineanu and Constantineanu (2014), Verheyde and Quicke (2022); Unpublished: Arter DK, iNaturalist, Insecte.org, Observation.org.

**Also mentioned in**: Sebald et al. (2000) [*Heterischnus thoracicus* Grav.].

#### Mevesia arguta

(Wesmael, 1845)

53F3748D-D9B8-5492-A037-924ECA50B416

##### Notes

As the original source for Bauer (2001) remains unclear, the only report is from Morley (1903). He reports to have found specimens several times during winter months at the roots of grass.

**Status**: Confirmed.

**Records**: United Kingdom, ca. 1903.

**Hibernacula**: LV (1).

**Sources**: Morley (1903) [*Phaeogenes argutus* Wesm.].

**Also mentioned in**: Valemberg (1978), Bauer (2001).

#### Oiorhinus pallipalpis

Wesmael, 1845

0FE90203-E60A-5799-ACA0-8D116D7E344B

##### Notes

Sebald et al. (2000) include it, stating that the label in Bauer’s collection records the specimen as having been collected while overwintering in situ. The second report, however, was from leaf litter (Diller and Shaw 2014).

**Status**: Confirmed.

**Records**: Germany (1), United Kingdom (1).

**First record**: 1980-2000 (Sebald et al. (2000), Germany).

**Hibernacula**: DV (1), U (1).

**Sources**: Sebald et al. (2000), Diller and Shaw (2014).

#### Oronotus binotatus

(Gravenhorst, 1829)

F2DA7C01-1C00-5688-B149-51EB82948AD0

##### Notes

Found at three different locations in three different hibernacula types in the Netherlands.

**Status**: Confirmed*.

**Records**: Belgium (3), the Netherlands (3).

**First record**: 8.II.2015 (Coll. RBINS, Belgium).

**Hibernacula**: DT (1), DV (1), LV (1), M (3).

**Sources**: Observation.org.

**Also mentioned in**: Sebald et al. (2000), Diller and Shaw (2014).

#### Phaeogenes melanogonos

(Gmelin, 1790)

DE5EBA0A-1E54-5084-9339-4C8494D36FF6

##### Notes

None.

**Status**: Confirmed.

**Records**: France, 1915-1922.

**Hibernacula**: LV (1).

**Sources**: Seyrig (1923) [*Phaeogenes melanogonus* Wsm.].

#### Phaeogenes planifrons

Wesmael, 1845

B331F839-68A0-53CB-859E-6F629A431879

##### Notes

None.

**Status**: Confirmed.

**Records**: France (1), Ireland (1).

**First record**: 1890-1920 (Johnson (1920), Ireland).

**Hibernacula**: LV (1), M (1).

**Sources**: Johnson (1920), Seyrig (1923).

#### Phaeogenes semivulpinus

(Gravenhorst, 1829)

A8626F62-D70D-5E8A-8FC5-72D6D5CE2552

##### Notes

Lungu-Constantineanu and Constantineanu (2014) erroneously call their finding a new hibernator for science.

**Status**: Confirmed.

**Records**: France (2), Romania (1).

**First record**: 1915-1922 (Seyrig (1923), France).

**Hibernacula**: LV (2), M (1).

**Sources**: Seyrig (1923), Lungu-Constantineanu and Constantineanu (2014).

#### Phaeogenes trepidus

Wesmael, 1845

8DC39B81-6E30-5357-8C9A-A1300DB5D55C

##### Notes

The hibernaculum type is not reported.

**Status**: Confirmed.

**Records**: United Kingdom, ca. 1903.

**Sources**: Morley (1903).

#### Stenodontus biguttatus

(Gravenhorst, 1829)

3999B24A-6C65-513A-9AC7-9F1F2138637A

##### Notes

This species was synonymised with *Stenodontus nasutus*.

**Status**: Confirmed.

**Records**: France (1), Germany (2), Romania (5).

**First record**: 1915-1922 (Seyrig (1923), France).

**Hibernacula**: DT (5), LT (1), M (1), U (1).

**Sources**: Seyrig (1923) [*Stenodontus nasutus* Wsm.], Constantineanu (1929b) [*S. nasutus* Wesm.], Constantineanu (1970) [*S. nasutus* Wesm.], Bauer (1984), Sebald et al. (2000).

#### Stenodontus marginellus

(Gravenhorst, 1829)

146FFEDC-469B-5521-A6C7-2F924BEF9035

##### Notes

None.

**Status**: Confirmed.

**Records**: France, ca. 1896.

**Hibernacula**: M (1).

**Sources**: Berthoumieu (1896).

**Also mentioned in**: Morley (1903).

#### Tycherus acutus

(Gravenhorst, 1829)

3DC197F7-957D-521D-8473-5B02C722656D

##### Notes

The context of this finding is unclear. It is mentioned as hibernating, based on museum material, which was included on the base of the date (Sebald et al. 2000).

**Status**: Unverified.

**Records**: Germany, 1980-2000.

**Sources**: Sebald et al. (2000).

#### Tycherus amaenus

(Wesmael, 1845)

F4BD382D-7423-555E-9B74-A780C6FDB6C4

##### Notes

One record from the personal collection of Bauer (undated) is included, the other one from museum material is of unclear origin (Sebald et al. 2000).

**Status**: Confirmed.

**Records**: Germany, 1980-2000.

**Sources**: Sebald et al. (2000).

#### Tycherus bellicornis

(Wesmael, 1845)

CB80AE46-D75F-544B-9478-809DC469B815

##### Notes

Sebald et al. (2001) mention this species is first reported as a hibernator in their paper, but both Ranin (1985) (without original data) and Gokhman (1989) already stated this species hibernated in hollow stems of umbellifers.

**Status**: Confirmed.

**Records**: Germany (3), Russia (1).

**First record**: 20.X.1980 (Gokhman (1988), Russia).

**Hibernacula**: LV (39), U (2).

**Sources**: Gokhman (1988), Ranin (1985), Sebald et al. (2000) and Sebald et al. (2001).

**Also mentioned in**: Gokhman (1989), Gokhman (2003).

#### Tycherus cephalotes

(Wesmael, 1845)

00A425E4-2530-5269-878B-4BE0AAEBA1F1

##### Notes

None.

**Status**: Confirmed.

**Records**: France (2), Romania (1).

**First record**: 2006-2010 (Lungu-Constantineanu and Constantineanu 2014, Romania).

**Hibernacula**: LT (1), LV (4).

**Sources**: Lungu-Constantineanu and Constantineanu (2014) [*Proscus cephalotes* Wesm.], Pénigot (2020).

#### Tycherus dilleri

Ranin, 1985

3467C719-85E3-563D-8641-07CAFE629972

##### Notes

Several paratypes of this species were collected hibernating, more specifically in hollow stems of umbellifers (Ranin 1985).

**Status**: Confirmed.

**Records**: Finland (13), Germany (1), Russia (1).

**First record**: 23.X.1977 (Ranin (1985), Finland).

**Hibernacula**: LV (26).

**Sources**: Ranin (1985), Gokhman (1988).

**Also mentioned in**: Gokhman (1989), Gokhman (2003).

#### Tycherus eques

(Wesmael, 1845)

9515DB02-8865-5324-B5BF-EAC3EE7A98B7

##### Notes

First reported by Rasnitsyn (1964) from the Province of Kalinin.

**Status**: Confirmed.

**Records**: Russia (1), United Kingdom (1)*.

**First record**: 1959-1964 (Rasnitsyn (1964), Russia).

**Hibernacula**: LV (1), U (1).

**Sources**: Rasnitsyn (1964) [*Phaeogenes eques* Wesm.]; Unpublished: Pers. obs.

#### Tycherus flavidens

(Wesmael, 1845)

BEDA731B-AFEF-5592-B330-9674F7602BAE

##### Notes

None.

**Status**: Confirmed.

**Records**: Belgium (1)*, France (2), Germany (1), Romania (1), Russia (1).

**First record**: Ca. 1871 (Tischbein (1871), Germany).

**Hibernacula**: DT (3), LT (1), LV (1), M (1).

**Sources**: Berthoumieu (1896) [*Phaeogenes flavidens* Wesm.], Constantineanu (1970), Valemberg (1976b) [*Proscus flavidens* Wesm.], Gokhman (1988); Unpublished: Coll. RBINS [*Phaeogenes flavidens*].

#### Tycherus fuscicornis

(Wesmael, 1845)

68C61317-B3DC-508C-9BFF-44296640D163

##### Notes

None.

**Status**: Confirmed.

**Records**: Germany (4), Russia (1), United Kingdom (1).

**First record**: 27.IX.1982 (Gokhman (1988), Russia).

**Hibernacula**: LV (16), U (2).

**Sources**: Bauer (1984) [*Phaeogenes fuscicornis* W.], Gokhman (1988), Sebald et al. (2000) and Sebald et al. (2001); Unpublished: GBIF.

**Also mentioned in**: Starke (1956) [*P. fuscicornis* Wesm.], Gokhman (1990a), Gokhman (2003).

#### Tycherus impiger

(Wesmael, 1845)

2D484920-A51F-5AE7-A05C-1112B544A330

##### Notes

None.

**Status**: Confirmed.

**Records**: France (1), Russia (1), United Kingdom (1).

**First record**: 1915-1922 (Seyrig (1923), France).

**Hibernacula**: LV (2), U (1).

**Sources**: Seyrig (1923) [*Phaeogenes impiger* Wsm.], Hancock (1925) [*P. impiger* Wesm.], Rasnitsyn (1964) [*P. impiger* Wesm.].

**Also mentioned in**: Bauer (2001).

#### Tycherus infimus

(Wesmael, 1845)

18A1776B-AE66-502F-91AF-BEA74B2AAE22

##### Notes

Morley found one specimen which was probably hibernating (Morley 1903) and with one additional uncertain record (GBIF), two uncertain records are known from the United Kingdom. For Germany, there is Sebald et al. (2000), which is uncertain as it is solely based on the collection date.

**Status**: Confirmed.

**Records**: Ireland, 1890-1920.

**Hibernacula**: M (1).

**Sources**: Johnson (1920).

**Also mentioned in**: Morley (1903) [*Phaeogenes infimus* Wesm.], Sebald et al. (2000).

#### Tycherus ischiomelinus

(Gravenhorst, 1829)

5F3AF10B-9328-5624-9910-B5F287C58526

##### Notes

The finding context of Bauer (2001) is unclear. There is one uncertain record from Sweden (GBIF).

**Status**: Confirmed.

**Records**: Germany (1), Russia (12).

**First record**: Ca. 1985-1988 (Gokhman (1988), Russia).

**Hibernacula**: LV (12), U (1).

**Sources**: Gokhman (1988), Bauer (2001).

**Also mentioned in**: Gokhman (1991), Gokhman (2003).

#### Tycherus nigridens

(Wesmael, 1845)

E4239F00-84F9-576D-80DF-E21A005BC0C1

##### Notes

This is one of the few species found in Romania in forest litter (Lungu-Constantineanu and Constantineanu 2014).

**Status**: Confirmed.

**Records**: Germany (1), Romania (1).

**First record**: Ca. 1871 (Tischbein (1871), Germany).

**Hibernacula**: DT (1), DV (1).

**Sources**: Tischbein (1871) [*Phaeogenes nigridens* W.], Lungu-Constantineanu and Constantineanu (2014).

#### Tycherus ophtalmicus

(Wesmael, 1845)

4947FDF5-5EC7-594A-BA96-90C8846533FA

##### Notes

None.

**Status**: Confirmed.

**Records**: Germany (2).

**First record**: Ca. 1984 (Bauer (1984), Germany).

**Hibernacula**: LV (1), U (1).

**Sources**: Bauer (1984) [*Dirophanes ophtalmicus* W.], Sebald et al. (2000).

#### Tycherus osculator

(Thunberg, 1824)

0897C897-544B-5619-A391-12A748E38592

##### Notes

During the 1980s and 1990s, this was by far the most common species in the Moscow region. It was not unusual to find several dozen females clustered inside the hollow stem of an umbellifer (Gokhman (1988) and pers. obs.).

**Status**: Confirmed.

**Records**: France (1), Germany (2), Poland (1), Russia (1).

**First record**: 26.XII.1925 (Seyrig (1926a), France).

**Hibernacula**: LV (350), U (4).

**Sources**: Seyrig (1926a) [*Phaeogenes oscultor* Thunb.], Hedwig (1927) [*P. osculator* Thunb.], Gokhman (1988), Sebald et al. (2000).

**Also mentioned in**: Ranin (1985), Gokhman (1989), Gokhman (2003).

#### Tycherus socialis

(Ratzeburg, 1852)

7A9A99AB-DE2B-534E-BF5B-BAF435DEDFA8

##### Notes

Unfortunately, the context of this finding is unclear.

**Status**: Confirmed.

**Records**: Germany, 1980-2001.

**Sources**: Sebald et al. (2000).

#### Tycherus stipator

(Wesmael, 1855)

5022B60D-92EB-516E-A3F2-A8F5C19BA410

##### Notes

Only one record from Morley (1903), who mentions he found it in the tufts of *Deschampsia cespitosa*.

**Status**: Confirmed.

**Records**: United Kingdom, ca. 1903.

**Hibernacula**: LV (1).

**Sources**: Morley (1903) [*Phaeogenes stipator* Wesm.].

#### Tycherus suspicax

(Wesmael, 1845)

5C7FCF33-C4D0-55FE-AC00-1B051CF8B91D

##### Notes

None.

**Status**: Confirmed.

**Records**: Germany (1), Russia (2), United Kingdom (8)*.

**First record**: 4.IV.1982 (Gokhman (1988), Russia).

**Hibernacula**: DT (2), LV (8), U (2).

**Sources**: Gokhman (1988), Sebald et al. (2000); Unpublished: Pers. obs.

#### Platylabini

DDE252C8-253C-5671-9337-5CE679A6B176

##### Notes

Reports of hibernation in this tribe are very scarce. At this moment, only tree species appear to be real hibernators (possibly facultative).

#### Apaeleticus mesostictus

(Gravenhorst, 1829)

7E492EA0-E7F2-5121-B24C-0E8FC69B1C8F

##### Notes

First reported by Lungu-Constantineanu and Constantineanu (2014) below cracks of a living tree, covered with moss. So far no other reports are, however, known for species in this genus.

**Status**: Unverified.

**Records**: Romania, 2006-2010.

**Hibernacula**: LT (1).

**Sources**: Lungu-Constantineanu and Constantineanu (2014).

#### Asthenolabus daemon

(Wesmael, 1845)

6DD54A32-14A6-514D-B64A-F3CAAD92DF00

##### Notes

The records by Sebald et al. (2000) and Sebald et al. (2001) may refer to the same specimen. Moreover, two additional records exist (hibernation uncertain), from 15.I.2008 and 15.I.2011 (GBIF).

**Status**: Confirmed.

**Records**: Germany, 1996-1997 (2 records).

**Hibernacula**: DT (1), U (1).

**Sources**: Sebald et al. (2000)/Sebald et al. (2001).

#### Linycus exhortator

(Fabricius, 1787)

F777DDBE-F763-5A3E-9C5A-7025DCFD9474

##### Notes

Although easily recognised and quite common, this species has not been observed in hibernation for over 125 years. Combining this with the known ecology of this species and the general ecology of Platylabini, we conclude more data are necessary to confirm this report.

**Status**: Unverified.

**Records**: France, ca. 1896.

**Hibernacula**: M (1).

**Sources**: Berthoumieu (1896) [*Platylabus dimidiatus* Grav.].

**Also mentioned in**: Morley (1903) [*P. dimidiatus* Grav.], Constantineanu (1959) [*P. exhortator* Fabr.].

#### Pristicerops infractorius

(Linnaeus, 1761)

FC8BE1EE-05BF-5674-8CA8-E39D91F0682D

##### Notes

Reported once by Heinrich (1949b)at a height of 1200 metres.

**Status**: Confirmed.

**Records**: Germany, V.1947-1948.

**Sources**: Heinrich (1949b) [*Pseudamblyteles infractorius* L.].

#### Probolus concinnus

Wesmael, 1853

7AE496AA-CEBC-5458-B3CB-FC1398338C51

##### Notes

The genus was recently revised by Horstmann (2000) and moved to Platylabini (Santos et al. 2021).

**Status**: Unverified.

**Records**: France, ca. 1896.

**Hibernacula**: M (1).

**Sources**: Berthoumieu (1896).

**Also mentioned in**: Morley (1903).

#### Probolus culpatorius

(Linnaeus, 1758)

2438DAEA-0C64-5259-AE2A-BF837E81613D

##### Notes

With a more clear taxonomic position (Horstmann 2000) and one recent record from Bauer (1984), we assume adult hibernation is likely here.

**Status**: Confirmed.

**Records**: France (1), Germany (1).

**First record**: Ca. 1896 (Berthoumieu (1896), France).

**Hibernacula**: M (1), S (1).

**Sources**: Berthoumieu (1896) [*Probolus alticola* Grav.], Bauer (1984) [*P. alticola* Grav.].

**Also mentioned in**: Morley (1903), Constantineanu (1959).

#### Mesochorinae

81ABD220-67F7-50F6-950A-6495456CC942

##### Notes

Mesochorinae are hyperparasitoids with a complex ecology and hibernation as a free-living adult is unlikely (Broad et al. 2018).

#### Mesochorus curvicauda

Thomson, 1886

F2A47D4E-D7E0-524A-8317-CEDC92822E19

##### Notes

This species is included in the list of Sebald et al. (2000), originating from the collection of Bauer. There is no context of this finding and it is likely this species was included, based on the collection date.

**Status**: Incorrect.

**Records**: Germany, 1980-2000.

**Sources**: Sebald et al. (2000).

#### Ophioninae

5F0475D3-C1A4-509D-8982-9CA7DFB71E80

##### Notes

The majority of species are koinobiont endoparasitoids of Lepidoptera larvae. They usually make their cocoon in the host’s pupation site, typically in the soil or litter layer (Broad et al. 2018).

#### Ophion obscuratus

Fabricius, 1798

5228AB40-BE0C-53F1-ABCE-688AE1D239AB

##### Notes

One specimen of *O. obscuratus* was caught using pitfall traps in a cave (not in hibernaculum). While this may indeed demonstrate the long phenology of this species (from autumn to winter), hibernation is unlikely knowing the ecology of the species in this genus (Johansson and Cederberg 2019).

**Status**: Incorrect.

**Records**: Hungary, 1.XII.2013.

**Sources**: Vas and Kutasi (2016).

#### Orthocentrinae

AAE61FF6-0884-586C-8E9F-339089DBDDAE

##### Notes

**Figure 7.**
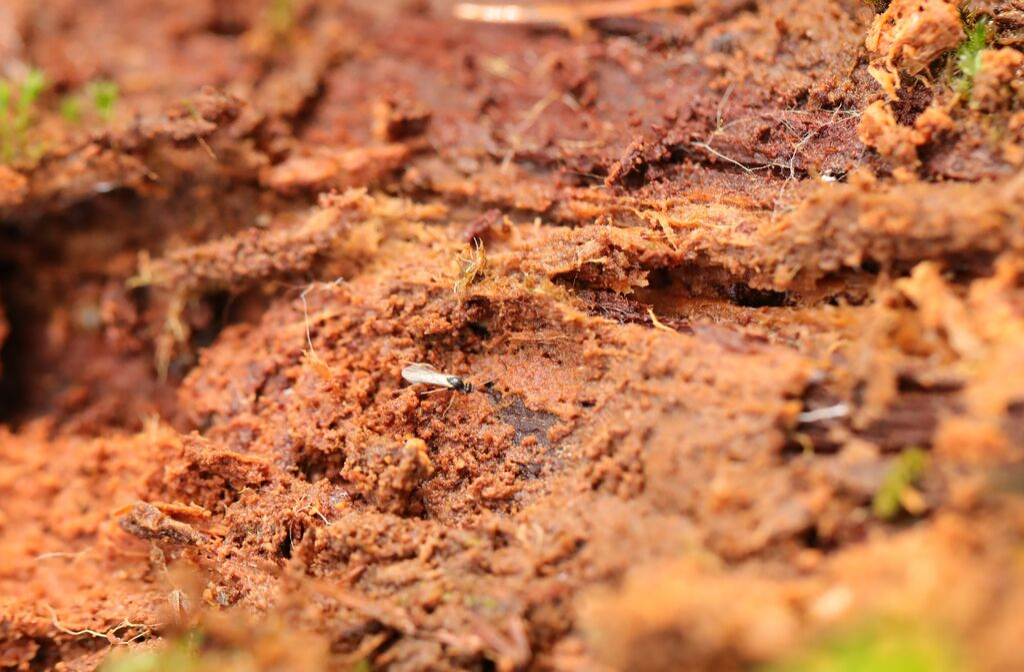
*Orthocentrus* spp. (here: *O. anomalus*) tend to hibernate more deeply in compact decaying dead wood, sometimes in larger aggregations - 28.XII.2021 (Belgium, Wijnendalebos), photograph by Patrick Debeuf.

The ecology of this subfamily is rather poorly known and taxonomic work is still needed. It bears similarities with Phygadeuontinae, as the hosts are also active during the entire year and the parasitoids, as a consequence, can be plurivoltine in some cases. Therefore, it is very important to see if species are found in hibernacula and results from beating trees can be more dubious as the species could be active at any moment during the year (males can sometimes be caught in January and February). At this moment, what seems clear is that several species in the *Orthocentrus*-group hibernate as free-living adults, preferring compact decaying wood (Fig. 7) or the soil around the tree’s root ball (Fig. 5b). Identification is hard and challenging, due to outdated keys and unresolved taxonomic issues. Additional records of *Orthocentrus* spp. exist from Belgium and the Netherlands (Observation.org), but also from Latvia, Lithuania and the United Kingdom (iNaturalist). Concerning other genera, nothing conclusive can be said at this stage. A record exists of an *Aperileptus* sp. beaten from ivy (Observation.org). There is one record of a *Helictes* sp. discovered while sieving the soil, in Norway (pers. obs.). Furthermore, there are records of two *Stenomacrus* spp., and one unidentified species from a cave (Vas and Kutasi 2016). *Megastylus cruentator* has also been reported from a cave.

#### Megastylus cruentator

Schiodte, 1838

4CBEEA0E-43EA-519A-95FA-BE986DD22936

##### Notes

The finding of this species in a cave is likely coincidental.

**Status**: Unverified.

**Records**: Poland, 1932-1934.

**Hibernacula**: C (1).

**Sources**: Pax and Maschke (1935) [*Megastylus cruentatus*].

**Also mentioned in**: Kocot-Zalewska and Domagała (2020).

#### Orthocentrus anomalus

Gravenhorst, 1829

B5D02D14-9205-5D57-B11F-0DC1D420866D

##### Notes

Similar to *O. fulvipes*, which has a shiny and punctate face and somewhat stouter flagellomeres, this species has been found in large quantities below bark and tree roots in Belgium and the Netherlands (five localities). It is by far the most common hibernating *Orthocentrus* sp. found. Typically it has ca. 26-29 flagellomeres, a black face and an orange posterior band on the second tergite. The specimens were identified by O. Varga as *O. petiolaris bicarinator*. This subspecies is a valid species already described as *O. anomalus*. Records of *O. petiolaris* sensu stricto could also refer to this subspecies, but we are not able to confirm this. *O. radialis*, another species mentioned in Sebald et al. (2000) and Sebald et al. (2001) will also be synonymised with *O. anomalus* (Varga, in prep.).

**Status**: Confirmed.

**Records**: Belgium (8)**, Germany (3), the Netherlands (2)**.

**First record**: Ca. 1996-1997 (Sebald et al. (2001), Germany).

**Hibernacula**: DT (61), DTCL (22), U (2).

**Sources**: Sebald et al. (2000)/Sebald et al. (2001) [*Orthocentrus radialis* Thoms.]; Unpublished: Pers. obs.

#### Orthocentrus asper

(Gravenhorst, 1829)

2CA2E72E-2E4D-55DC-BDA6-3A9F40C1DBD3

##### Notes

A species which appears to associated with pine forests. Up to 190 individuals were observed at once by Rasnitsyn (1959).

**Status**: Confirmed.

**Records**: Norway (1), Russia (1).

**First record**: 1951-1958 (Rasnitsyn (1959), Russia).

**Hibernacula**: DT (622).

**Sources**: Rasnitsyn (1959); Unpublished: Pers. obs.

#### Orthocentrus frontator

(Zetterstedt, 1838)

12416ACF-3C5C-5855-873D-28A2ABAF4A81

##### Notes

This species is difficult to interpret taxonomically and may be confused with *O. sannio*. One possible specimen from the Netherlands, below bark, could not be identified with certainty (Observation.org).

**Status**: Unverified.

**Records**: Germany, 1996-1997 (3 records).

**Hibernacula**: DT (5), U (2).

**Sources**: Sebald et al. (2000)/Sebald et al. (2001).

#### Orthocentrus fulvipes

Gravenhorst, 1829

78F89284-3AD0-5567-B3B1-DF23D736390F

##### Notes

Similar to *O. anomalus*. Two recent specimens were collected by beating trees; one in the Netherlands and one in the United Kingdom. The specimen from Johnson (1920) was found in moss in January.

**Status**: Confirmed.

**Records**: Ireland (1), Romania (1), the Netherlands (1)*, United Kingdom (1)*.

**First record**: 1890-1920 (Johnson (1920), Ireland).

**Hibernacula**: C (1), LV (2), M (1).

**Sources**: Johnson (1920), Decou and Decou (1961); Unpublished: GBIF, Observation.org.

#### Orthocentrus petiolaris

Thomson, 1897

6BD0F57C-AE3A-5C83-8EE7-06BCA100CB99

##### Notes

The *Orthocentrus* sp. mentioned in Bauer (1984) was later identified as *O. petiolaris* in Sebald et al. (2000). Due to the taxonomic changes related to the subspecies bicarinator (*O. anomalus*), we cannot be sure about the identity of these specimens.

**Status**: Unverified.

**Records**: Germany (1), Romania (2).

**First record**: 1964-1967 (Constantineanu (1969), Romania).

**Hibernacula**: DT (2), U (1).

**Sources**: Constantineanu (1969), Sebald et al. (2000), Lungu-Constantineanu and Constantineanu (2014).

#### Orthocentrus sannio

Holmgren, 1858

A416F7B2-6FBD-584E-8433-7603560C0D10

##### Notes

Several specimens were collected and investigated. They do not match completely with the current species concept of *O. sannio*. For example, the areolet is sessile (and not petiolate). This may lead to taxonomic changes (O. Varga, pers. comm.).

**Status**: Confirmed.

**Records**: Belgium, 18.I.2020.

**Hibernacula**: DT (20).

**Sources**: Verheyde and Quicke (2022).

#### Orthocentrus spurius

Gravenhorst, 1829

D53474FC-AF0E-5DAD-ADB8-EA16273B4B4E

##### Notes

A species group which needs to be revised and consists of multiple species (O. Varga pers. comm.). Sebald et al. (2000) include it, stating that the label in Bauer’s collection records the specimen as having been collected while overwintering in situ. There are several additional uncertain records of the *O. spurius*-group from beating trees in the Netherlands (Observation.org).

**Status**: Confirmed.

**Records**: Germany, ca. 2000.

**Sources**: Sebald et al. (2000) [*Orthocentrus spurious* Grav.].

#### Plectiscus impurator

Gravenhorst, 1829

781E92C5-5185-5643-B73C-10E6DCF84886

##### Notes

Most findings were made in hollow stems of umbellifers, one of them consisted of 25 individuals grouped together in the Netherlands (Observation.org). One uncertain record from Norway, February 2022 (GBIF).

**Status**: Confirmed.

**Records**: Belgium (1)*, Germany (4), the Netherlands (1)*.

**First record**: Ca. 1984 (Bauer (1984), Germany).

**Hibernacula**: LV (32), U (2).

**Sources**: Bauer (1984) [*Stenomacrus ventralis* Hlgr.], Sebald et al. (2000)/Sebald et al. (2001); Unpublished: Observation.org.

#### Stenomacrus caudatus

(Holmgren, 1858)

61E50D46-18F0-5A5A-B3B6-E029BBA7A6C2

##### Notes

This species is reported from a cave, but the date suggests this finding is likely to be coincidental.

**Status**: Unverified.

**Records**: Romania, 16.VII.1960.

**Sources**: Decou and Decou (1961).

#### Stenomacrus cubiceps

(Thomson, 1897)

CA672139-3842-5D84-BB93-50D926885B57

##### Notes

Sebald et al. (2000) include it, stating that the label in Bauer’s collection records the specimen as having been collected while overwintering in situ.

**Status**: Unverified.

**Records**: Germany, ca. 2000.

**Sources**: Sebald et al. (2000).

#### Phygadeuontinae

9AEAFE82-6F6A-5704-A1F4-1251BF61D896

##### Notes

One of the most diverse subfamilies with regards to host usage, which are more or less concealed hosts (Broad et al. 2018). Several species-rich genera contain species using multiple hibernation strategies (e.g. *Aclastus*, *Charitopes* and *Gelis*). More exceptionally, even at the species (or population) level, we have evidence that multiple mechanisms are used. This may be related to host usage (mainly Lepidoptera, although *Aclastus* and *Charitopes* spp. are associated with spiders and Neuroptera, respectively) and geography (see Discussion). It it is often not entirely clear if there is a period of quiescence or facultative or obligate diapause. To complicate things, identification is often difficult in this subfamily. There are several taxonomic issues and some genera need to be revised. As a consequence, we have been reviewing the reports of the genus *Phygadeuon* quite critically and classified most species as ‘Unverified’. For several other genera, we cannot say a lot at this stage. Some of these genera seem to be more likely to hibernate (e.g. *Arotrephes*, *Gnotus*) than others (e.g. *Dichrogaster*, *Endasys*, *Hemiteles* and *Lysibia*), but more data are needed.

#### Aclastus gracilis

(Thomson, 1884)

5366C31B-92D8-5596-AB69-6290E4D75F99

##### Notes

Although Horstmann (1970) and Horstmann (1980) do not mention any original data, it is stated the species is common below bark, also on living trees. Not included were several specimens from Finland in November, found by beating vegetation.

**Status**: Confirmed.

**Records**: Belgium (2), Germany (3).

**First record**: 16.II.1906 (Anonymous (1909), Belgium).

**Hibernacula**: M (2), S (1), U (2).

**Sources**: Anonymous (1909), Horstmann (1970) and Horstmann (1980), Bauer (1984).

#### Aclastus micator

(Gravenhorst, 1807)

0CDA5926-B526-50AD-93BE-7C8C8FC587AA

##### Notes

Hancock (1925) mentions he collected a specimen between the sheathing leaf-bases of *Carex pendula* in March. It is also mentioned as an adult hibernator in Ulrich (2001), but more context is missing here. The species is not reported to hibernate in the revisions of Horstmann (1970) and Horstmann (1980).

**Status**: Unverified.

**Records**: United Kingdom, 12.III.1924.

**Sources**: Hancock (1925) [*Hemiteles necator* Grav.].

**Also mentioned in**: Ulrich (2001).

#### Aclastus minutus

(Bridgman, 1886)

0E4D9529-BDA8-554B-BE7A-7A3406D56444

##### Notes

One specimen was found below the bark of a fence post (Horstmann 1980).

**Status**: Confirmed.

**Records**: Germany (2).

**First record**: Ca. 1970 (Horstmann (1970), Germany).

**Hibernacula**: O (1), U (1).

**Sources**: Horstmann (1970) and Horstmann (1980).

#### Aclastus pilosus

Horstmann, 1980

14C931CE-5FE5-593F-85E2-C243EB3E39D2

##### Notes

Similar to *A. micator* (Horstmann 1980). There is one uncertain record from Schwarz and Shaw (2000). One female emerged in February from a ‘witches broom’ in a birch, collected in December, but it is not clear whether the specimen overwintered as a cocoon or as a free-living adult.

**Status**: Unverified.

**Records**: United Kingdom, undated.

**Sources**: Schwarz and Shaw (2000).

#### Arotrephes perfusor

(Gravenhorst, 1829)

45B0A90E-4C38-5F70-9944-22BC2CEBDA8B

##### Notes

Schwarz and Shaw (2000) state it is very likely this species hibernates as it is active early in the year and almost certain univoltine (this could also be as a pharate adult).

**Status**: Unverified.

**Sources**: Schwarz and Shaw (2000).

#### Charitopes clausus

(Thomson, 1888)

5E3DECBD-B0D6-5FA3-A122-30806E2C1FB2

##### Notes

At least three original records are known as Schwarz and Shaw (2010) mention this species was frequently found hibernating under bark of beech, oak and pine between December and March. Two additional uncertain records exist from January 1925 in Sweden (GBIF).

**Status**: Confirmed.

**Records**: United Kingdom (3 records).

**Hibernacula**: DT (3).

**Sources**: Schwarz and Shaw (2010).

#### Charitopes gastricus

(Holmgren, 1868)

0003208B-135A-518A-B795-D7788CF0D252

##### Notes

Schwarz and Shaw (2010) reported on specimen labelled ‘winter’.

**Status**: Unverified.

**Records**: France or United Kingdom, undated.

**Sources**: Schwarz and Shaw (2010).

#### Dichrogaster longicaudata

(Thomson, 1884)

3E8A7CEA-B2B6-5901-B47B-206AFBF14385

##### Notes

One specimen was collected on 29.I.1976 in Finland, but the context of this finding is unclear.

**Status**: Unverified.

**Records**: Finland, 29.I.1976.

**Sources**: Pers. obs.

#### Endasys euxestus

(Speiser, 1908)

EFDC182E-EDC7-55E5-93A1-17F8B8240B87

##### Notes

Sebald et al. (2000) include it, stating that the label in Bauer’s collection records the specimen as having been collected while overwintering in situ. No other species of the genus are known hibernators and species are mainly parasitoids of sawfly cocoons (Broad et al. 2018).

**Status**: Unverified.

**Records**: Germany, 1980-2000.

**Sources**: Sebald et al. (2000).

#### Gelis acarorum

(Linnaeus, 1758)

ED3990EB-6B17-5D61-B469-21FA1E628738

##### Notes

Schwarz (2002) states this species probably hibernates. Two uncertain records exist from the Netherlands, found in natural beach wrack in February. One specimen in Bulgaria was discovered below the bark of a living tree (iNaturalist), 30.I.2024, but the identification remains uncertain.

**Status**: Unverified.

**Sources**: Schwarz (2002).

#### Gelis agilis

(Fabricius, 1775)

76FBEBAF-3C55-5FE7-AB46-6065F2E1CC2E

##### Notes

So far, this species has been mostly found in grass tussocks in the United Kingdom. Several uncertain hibernators were found by beating vegetation in Finland in November.

**Status**: Confirmed.

**Records**: The Netherlands (2)*, United Kingdom (6).

**First record**: 22.XI.1923 (Hancock (1925), United Kingdom).

**Hibernacula**: DV (2), LV (14).

**Sources**: Hancock (1925) [*Pezomachus instabilis* Forst.], Storey et al. (2025); Unpublished: Observation.org, Pers. obs.

**Also mentioned in**: Schwarz (1998).

#### Gelis anthracinus

(Förster, 1850)

0DBE088E-1CAF-5E6B-B5D4-047C7D2F6C7B

##### Notes

Schwarz and Shaw (1999) suggest it is likely that the species is univoltine and that females pass the winter as adults.

**Status**: Unverified.

**Sources**: Schwarz and Shaw (1999).

#### Gelis areator

(Panzer, 1804)

8B41DB63-7BD6-5B90-8D4C-3DDE0B832CB6

##### Notes

One of the most common species of Ichneumonidae in Europe. This species is one of the rare examples where it has been proven that different hibernation strategies are used, presumably depending on the hosts, which are very diverse in this species. More specifically, both hibernation in the host case/cocoon and hibernation as free-living adult are known (Schwarz and Shaw (1999); it is unclear from which country the valid records originate). Specimens have been found below bark, in fungi and in ‘burrows in holly' (Morley 1907).

**Status**: Confirmed.

**Records**: Belgium (1)*, Finland (1)*, the Netherlands (2)*, United Kingdom (3).

**First record**: II.1899 (Morley (1907), United Kingdom).

**Hibernacula**: DT (5), LV (1), O (3), U (2).

**Sources**: Morley (1907) [*Hemiteles aerator* Panz.], Schwarz and Shaw (1999); Unpublished: iNaturalist, Observation.org, Pers. obs.

#### Gelis bicolor

(Villers, 1789)

9C48A1F2-4588-551D-9874-6897A5B47C4D

##### Notes

Schwarz and Shaw (1999) and Schwarz (2002) hypothesised that the species hibernated. This is confirmed here by two independent findings in grass tussocks. Additionally, several specimens collected by beating vegetation in Finland and the United Kingdom were excluded due to difficulties in interpretation. Another uncertain record consists of a specimen in the Netherlands, found on a fence post in January.

**Status**: Confirmed*.

**Records**: United Kingdom (2).

**First record**: 18.X.2020 (Pers. obs. - M. Storey, United Kingdom).

**Hibernacula**: LV (2).

**Sources**: Pers. obs.

**Also mentioned in**: Schwarz and Shaw (1999), Schwarz (2002).

#### Gelis brevis

(Bridgman, 1883)

A85E3584-ADD5-5E4D-9427-1ADB499B418C

##### Notes

Based on the ecology and phenology, Schwarz (2002) suggests it is likely that females hibernate.

**Status**: Unverified.

**Sources**: Schwarz (2002).

#### Gelis brevithorax

Roman, 1936

0D8B6B39-5199-583A-B284-FAF5DAD7DA4B

##### Notes

Based on the ecology and phenology, Schwarz (2002) suggests it is likely that females hibernate.

**Status**: Unverified.

**Sources**: Schwarz (2002).

#### Gelis caudator

Horstmann, 1986

9DA4C0FF-12D3-5482-8703-5FD4605E4096

##### Notes

Schwarz (1994) described in detail how females hibernate in cracks of spruce trees and presumably birches, in Austria.

**Status**: Confirmed.

**Records**: Austria, ca. 1994.

**Hibernacula**: DT (1).

**Sources**: Schwarz (1994).

#### Gelis cursitans

(Fabricius, 1775)

44A45FD8-9125-5A37-871F-DD04A88F4C18

##### Notes

Based on the ecology and phenology, Schwarz (1998) suggests it is likely that females hibernate as an adult, although some specimens (e.g. those with the sawfly species *Diprion pini* as a host) have been found hibernating in the host cocoon.

**Status**: Unverified.

**Sources**: Schwarz (1998).

#### Gelis cyanurus

(Förster, 1850)

B9186FCC-D1B7-5A6A-98CB-5B05DEEB7002

##### Notes

One specimen was discovered below a stone in the south of France, but it is uncertain if this can be seen as real hibernation or if the species were already active.

**Status**: Unverified.

**Records**: France, 18.II.2024.

**Hibernaculum**: S (1).

**Sources**: iNaturalist.

#### Gelis declivis

(Förster, 1850)

5509058C-B2B0-5C16-8653-167ED5DA1F96

##### Notes

Schwarz (2002) mentions this species can be found hibernating in straw and detritus, but unfortunately mentions no original data. One recent finding from Sweden (January) was indeed discovered while sieving litter. One specimen from Finland (found in vegetation) was not included as the hibernation status is uncertain.

**Status**: Confirmed.

**Records**: Sweden (1)*, Unknown (1).

**First record**: Ca. 2002 (Schwarz (2002), unknown country).

**Hibernacula**: DV (2).

**Sources**: Schwarz (2002); Unpublished: GBIF.

#### Gelis discedens

(Förster, 1850)

E948C3C0-0D64-57E1-99D8-CC20D3EF7528

##### Notes

No specimens have been found in hibernacula so far, although it is predicted this species hibernates. In Finland, one specimen was found with a sweep net in vegetation, but as this was in November, hibernation is still not confirmed.

**Status**: Unverified.

**Sources**: Schwarz and Shaw (1999), Schwarz (2002).

#### Gelis fallax

(Förster, 1850)

F4DE717A-BA1E-5660-A253-48613A5A7EDB

##### Notes

The first finding of this species in hibernation was made by sieving hay from a hay ball in Sweden. The other observations consist of specimens hibernating in grass tussocks in the United Kingdom. Two specimens from Finland collected with a sweep net in November were not included.

**Status**: Confirmed*.

**Records**: Sweden (1), United Kingdom (3).

**First record**: 5.X.2016 (GBIF, Sweden).

**Hibernacula**: DV (1), LV (5).

**Sources**: GBIF, Pers. obs.

#### Gelis festinans

(Fabricius, 1798)

5B13531D-F1DD-5614-8099-CC90026F1B06

##### Notes

First reported by Rasnitsyn (1964) from the Moscow Province, matching the hypothesis from Schwarz (2002). Another recent observation originates from sieving soil in the Netherlands.

**Status**: Confirmed.

**Records**: Russia (1), the Netherlands (1)*.

**First record**: 1959-1964 (Rasnitsyn (1964), Russia).

**Hibernacula**: DV (1), U (1).

**Sources**: Rasnitsyn (1964) [*Gelis posthuma* Först.]; Unpublished: Coll. Naturalis.

**Also mentioned in**: Schwarz (2002).

#### Gelis fuscicornis

(Retzius, 1783)

E0F285E8-6EBC-5076-B91E-55E6F0764918

##### Notes

Schwarz and Shaw (1999) suggested the females hibernate. This is now confirmed by findings from Finland. Six specimens were discovered from beating trees, notably spruces, in December and March in Finland. Some additional records were not included, as these were mostly collected with a sweep net from lower vegetation in November.

**Status**: Confirmed*.

**Records**: Finland (4).

**First record**: 17.III.2007 (Pers. obs., Finland).

**Hibernacula**: LT (6).

**Sources**: Schwarz and Shaw (1999) [*Gelis longulus* Zett.], Pers. obs.

#### Gelis hortensis

(Christ, 1791)

E02641DE-584F-5217-8FB0-6EAC6997277E

##### Notes

Another species hypothesised to hibernate by Schwarz & Shaw and now confirmed from observations in several countries. Four of the six specimens were found in wetlands, hibernating in the hollow stems of *Carex* and *Typha*. One uncertain record exists from the Netherlands, found in natural beach wrack and there are several uncertain records from Finland, swept from vegetation in November.

**Status**: Confirmed*.

**Records**: Belgium (2)**, Finland (1), Ukraine (1), United Kingdom (1).

**First record**: 10.XII.2006 (Pers. obs., Finland).

**Hibernacula**: DV (2), LV (4).

**Sources**: iNaturalist, Pers. obs.

**Also mentioned in**: Schwarz and Shaw (1999), Schwarz (2002).

#### Gelis intermedius

(Förster, 1850)

B4C24686-CC10-5452-8731-254EB9AA6D21

##### Notes

Schwarz (2002) hypothesises that this species hibernates, but this in fact has already been reported by Morley (1907) from different hibernacula.

**Status**: Confirmed.

**Records**: United Kingdom, ca. 1907.

**Hibernacula**: LV (1), S (1), U (5).

**Sources**: Morley (1907) [*Pezomachus intermedius* Först.].

**Also mentioned in**: Schwarz (2002).

#### Gelis kiesenwetteri

(Förster, 1850)

AC74ACEA-CA11-5D8D-ACA9-4E8C62B4707F

##### Notes

First reported by Rasnitsyn (1964), but as the exact context of this finding is unclear, and as Schwarz (1998) mentions this species hibernates in the host cocoon, it is unclear how to interpret his results. Similar to *G. areator*, the use of multiple hibernation strategies could be possible, but this is speculative.

**Status**: Unverified.

**Records**: Russia, 1959-1964.

**Sources**: Rasnitsyn (1964) [*Gelis debei* Först.].

**Also mentioned in**: Schwarz (1998).

#### Gelis mangeri

(Gravenhorst, 1815)

B395E19C-DF05-5F9B-B174-686E89E40083

##### Notes

Clearly associated with reed-bed, and similar to, for example *G. hortensis*, most specimens were found in the hollow stems of *Carex* and *Typha* (also at the leaf sheaths).

**Status**: Confirmed.

**Records**: Belgium (4)*, the Netherlands (1)*, United Kingdom (2)*.

**First record**: Ca. 1999 (Schwarz and Shaw (1999), United Kingdom).

**Hibernacula**: LV (7), U (1).

**Sources**: Schwarz and Shaw (1999); Unpublished: iNaturalist, Observation.org, Pers. obs.

#### Gelis meigenii

(Forster, 1850)

BFD2E0CE-700F-5059-A857-D76424300E0E

##### Notes

Hypothesised to hibernate by Schwarz (1998), recently confirmed by one finding on the dangling skirt of a grass tussock. Two uncertain specimens exist from the Netherlands, one found in natural beach wrack and one beaten from vegetation in January.

**Status**: Confirmed*.

**Records**: United Kingdom, 17.II.2023.

**Hibernacula**: LV (1).

**Sources**: Pers. obs.

**Also mentioned in**: Schwarz (1998).

#### Gelis melanocephalus

(Schrank, 1781)

84FE5D30-7AAF-5ABC-B2DE-DAB98B6FF2B9

##### Notes

Schwarz and Shaw (1999) stated this species probably hibernates, but this was already observed by Johnson (1920). However, more recent observations have been added. Most specimens were found in decaying vegetation or beaten from shrubs. Some were found in grass tussocks. Several observations were not included as uncertain, including some from Finland in vegetation.

**Status**: Confirmed.

**Records**: Belgium (2)*, Denmark (2)*, Germany (2)*, Ireland (1), the Netherlands (6)*, United Kingdom (5).

**First record**: 1890-1920 (Johnson (1920), Ireland).

**Hibernacula**: DV (4), LT (1), LV (14), M (1).

**Sources**: Johnson (1920) [*Pezomachus fasciatus* Fub.], Storey et al. (2025); Unpublished: Arter DK, Coll. Naturalis, iNaturalist, Observation.org; Pers. obs.

**Also mentioned in**: Schwarz and Shaw (1999), Schwarz (2002).

#### Gelis melanophorus

(Förster, 1850)

550DA06D-3C8D-591D-917F-33043B01D51A

##### Notes

Hypothesised to hibernate by Schwarz and Shaw (1999), recently confirmed by a finding in a grass tussock.

**Status**: Confirmed*.

**Records**: United Kingdom, 4.I.2021.

**Hibernacula**: LV (1).

**Sources**: Pers. obs.

**Also mentioned in**: Schwarz and Shaw (1999), Schwarz (2002).

#### Gelis mutillatus

(Gmelin, 1790)

AA766B17-6726-5E7B-B416-355CD820DB06

##### Notes

Schwarz (1998) mentions females probably hibernate, but this was already observed by Johnson (1920). One more recent record from Bauer was added (Sebald et al. 2000).

**Status**: Confirmed.

**Records**: Germany (1), Ireland (1).

**First record**: 1890-1920 (Johnson (1920), Ireland).

**Hibernacula**: M (1), U (1).

**Sources**: Johnson (1920) [*Pezomachus vagans* Oliv.], Sebald et al. (2000).

**Also mentioned in**: Schwarz (1998).

#### Gelis nigritulus

(Zetterstedt, 1838)

B3427B97-6252-51D5-8816-828C03B1473D

##### Notes

Similar situation as with *Gelis kiesenwetteri*. Sebald et al. (2000) include it, stating that the label in Bauer’s collection records the specimen as having been collected while overwintering in situ, while Schwarz and Shaw (1999) state the species passes the winter in the host cocoon (possibly as pre-adult). Another uncertain record exists from Finland, where a specimen was found beneath a piece of wood on a sun-exposed house wall in March (Pers. obs.).

**Status**: Unverified.

**Records**: Germany, ca. 2000.

**Sources**: Sebald et al. (2000).

**Also mentioned in**: Schwarz and Shaw (1999).

#### Gelis proximus

(Förster, 1850)

695171A1-AC2B-584C-84A3-5CC7FE8F6487

##### Notes

Reported also hibernating in the host cocoon (Schwarz 2002). Five additional records from Finland beaten from vegetation in November (Pers. obs.) were not included.

**Status**: Confirmed.

**Records**: Austria (1), Belgium (5)*, Finland (1)*, France (1), Russia (1), the Netherlands (1)*, United Kingdom (8).

**First record**: 22.XI.1923 (Hancock (1925), United Kingdom).

**Hibernacula**: DV (8), LV (8), O (1), U (1).

**Sources**: Hancock (1925) [*Pezomachus corruptor* Forst.], Aubert (1959) [*Gelis corruptor*/*faunus*/*parisiensis*], Rasnitsyn (1964) [*G. corruptor* Först.], Schwarz (2002); Unpublished: Coll. RBINS, Pers. obs.

**Also mentioned in**: Schwarz and Shaw (1999).

#### Gelis recens

Schwarz, 2002

3001091E-7B1D-5229-ACD7-85CBCCA5AAA0

##### Notes

Based on the ecology and phenology, Schwarz (2002) suggests it is likely that females hibernate.

**Status**: Unverified.

**Sources**: Schwarz (2002).

#### Gelis rufogaster

Thunberg, 1827

A95D3A89-F266-5898-8D85-20EC934B6BB5

##### Notes

Thought to be hibernating by Schwarz and Shaw (1999), now confirmed from five countries. Most specimens were found on evergreen plants, notably ivy, but also common heather. Several specimens collected with a sweep net in vegetation in Belgium and Finland were not included.

**Status**: Confirmed*.

**Records**: Belgium (3), Finland (3), France (1), the Netherlands (4), United Kingdom (4).

**First record**: 10.XII.2006 (Pers. obs. - A. Albrecht & I. Österblad, Finland).

**Hibernacula**: DV (4), LT (2), LV (16), O (1), U (1).

**Sources**: Coll. RBINS, Coll. Naturalis, iNaturalist, Observation.org, Pers. obs.

**Also mentioned in**: Schwarz and Shaw (1999), Schwarz (2002).

#### Gelis spurius

(Förster, 1850)

B0F05CC3-4BD8-5431-8B05-50FEBBCFA5B4

##### Notes

One uncertain record from Germany in Förster (1850) (as *Pezomachus tristis*), who found a specimen on 21 December 1848 (see also Schwarz (1995)). Several uncertain records from Finland and the United Kingdom (found in vegetation).

**Status**: Confirmed.

**Records**: Belgium (6)*, Germany (1)*, United Kingdom (2)*, the Netherlands (4)*.

**First record**: Ca. 2002 (Schwarz (2002), unknown country).

**Hibernacula**: DV (9), LV (5), M (1).

**Sources**: Schwarz (2002); Unpublished: Coll. RBINS, Observation. org, Pers. obs.

#### Gelis tibiator

Schwarz, 2002

EA4CD3BC-15AF-54D5-98F8-3CF0B80994D4

##### Notes

Based on the ecology and phenology, Schwarz (2002) suggests it is likely that females hibernate.

**Status**: Unverified.

**Sources**: Schwarz (2002).

#### Gelis trux

(Förster, 1850)

6682A070-2EA4-56FA-B94F-8483548345D6

##### Notes

Hypothesised to hibernate by Schwarz (2002), recently confirmed by two findings in a grass tussock. Specimens from Finland, found in vegetation with a sweep net in November, were not included.

**Status**: Confirmed*.

**Records**: United Kingdom (2).

**First record**: 1.III.2020 (Pers. obs., United Kingdom).

**Hibernacula**: LV (2).

**Sources**: Pers. obs.

**Also mentioned in**: Schwarz (2002).

#### Gelis venatorius

(Förster, 1850)

0A7A4CF1-1278-51E8-A33B-A79A0A168C36

##### Notes

One specimen was found in a tree stump of *Populus tremula* in Finland.

**Status**: Confirmed.

**Records**: Finland, ca. 2002.

**Hibernacula**: DT (1).

**Sources**: Schwarz (2002).

#### Gelis viduus

(Förster, 1850)

0D878946-3E76-593A-B424-1F767347F16D

##### Notes

Schwarz and Shaw (1999) already hypothesised that this species hibernated. This is now confirmed. One additional uncertain record from Sweden, 22.XII.1993 (GBIF) and two uncertain records from Finland beaten from vegetation in November (Pers. obs.) have not been included.

**Status**: Confirmed*.

**Records**: Finland (1), Norway (1), United Kingdom (1).

**First record**: 17.XI.2012 (GBIF, Norway).

**Hibernacula**: LT (1), LV (1), M (1).

**Sources**: GBIF, Pers. obs.

**Also mentioned in**: Schwarz and Shaw (1999), Schwarz (2002).

#### Gelis vulnerans

(Förster, 1850)

BA626A92-826B-54E7-96B8-F778CCA43986

##### Notes

Specimens were found in moss and below snow in November and December (Schwarz 2002).

**Status**: Confirmed.

**Records**: Austria, Germany or Italy.

**Hibernacula**: M (2).

**Sources**: Schwarz (2002).

#### Gnotus chionops

(Gravenhorst, 1829)

A160FBF0-8DBA-5689-88FA-D4CB5360AA07

##### Notes

All specimens were found by beating *Quercus ilex* in the United Kingdom. One specimen was found in the middle of January. However, the identification needs to be verified. Some host records, which we have, suggest the species are associated with smaller Lepidoptera (Horstmann 1993, Schwarz and Shaw 2011).

**Status**: Unverified.

**Records**: United Kingdom (5).

**First record**: 20.XI.2020 (iNaturalist, United Kingdom).

**Hibernacula**: LT (5).

**Sources**: iNaturalist.

#### Hemiteles similis

(Gmelin, 1790)

F9315114-D764-5B06-85F0-40CB092007DF

##### Notes

Listed in Rasnitsyn (1964) as a hibernator, but the context of the finding is unclear. Specimens are often observed late in the year, also in houses or possibly caves (Decou and Decou 1961). However, it has been stated how this species overwinters in the host sac (Schwarz and Shaw 2000). In this case, it is less likely other hibernation strategies are used.

**Status**: Unverified.

**Records**: Russia, 1959-1964.

**Sources**: Rasnitsyn (1964).

**Also mentioned in**: Schwarz and Shaw (2000).

#### Lysibia nana

(Gravenhorst, 1829)

DED81FD2-7617-51A3-A885-E3EDFF25627B

##### Notes

One specimen reported in a cave from Pax and Maschke (1935). This will be a coincidental finding as this species is plurivoltine and has a long flight period. As far as known, it overwinters in the host cocoon and is a parasitoid of Microgastrinae (Schwarz and Shaw 2000).

**Status**: Unverified.

**Records**: Poland, 1932-1934.

**Sources**: Pax and Maschke (1935) [*Hemiteles fulvipes* Grav.].

**Also mentioned in**: Schwarz and Shaw (2000), Kocot-Zalewska and Domagała (2020).

#### Medophron afflictor

(Gravenhorst, 1829)

F0F33431-0654-5180-AB8F-8E6B63C7E6CE

##### Notes

At least three specimens were found in grass tussocks by Seyrig (1923) in France.

**Status**: Confirmed.

**Records**: France, 1915-1922.

**Hibernacula**: LV (3).

**Sources**: Seyrig (1923) [*Acanthocryptus nigritus* Gr.].

#### Medophron crassicornis

(Gravenhorst, 1829)

D34383B2-5564-5B7A-8496-0EFC56848FC3

##### Notes

One specimen was discovered in a rush tussock in the United Kingdom.

**Status**: Confirmed*.

**Records**: United Kingdom, 23.II.2021.

**Hibernacula**: LV (1).

**Sources**: Pers. obs.

#### Mesoleptus laevigatus

(Gravenhorst, 1820)

1398442F-1085-577B-A817-18F9FC95E263

##### Notes

One specimen was found in grass tussocks by Seyrig (1923) in France. Hibernation as an adult does not seem to match the known biology of the genus, which is mainly associated with Diptera (Jussila et al. 2010). As this species is also quite common and there are no recent records, new data are needed.

**Status**: Unverified.

**Records**: France, 1915-1922.

**Hibernacula**: LV (1).

**Sources**: Seyrig (1923) [*Exolytus laevigatus* Gr.].

**Also mentioned in**: Rasnitsyn (1964) [*E. laevigatus* Grav.].

#### Micromonodon tener

(Kriechbaumer, 1893)

CEA87DCE-228B-5A7E-B72E-E4B8C0743FBB

##### Notes

Active specimens have been observed as early as March (Observation.org), some of them ovipositing on living trees. It is unclear whether the species really hibernates, has a phase of quiescence or hibernates in the host cocoon (located in the direct vicinity of trees). Starke (1956) nonetheless reports his specimen in ‘winter rest’ below the bark of a maple tree.

**Status**: Unverified.

**Records**: Germany, 9.IV.1939.

**Sources**: Starke (1956).

#### Phygadeuon brevitarsis

Thomson, 1884

A64C0C28-7343-5432-9639-7E3B66FF7F65

##### Notes

This species was beaten from a pine tree, but as this is early spring, it is unclear if the species was really hibernating (Morley 1907).

**Status**: Unverified.

**Records**: United Kingdom, 2.IV.1899.

**Hibernacula**: LT (1).

**Sources**: Morley (1907).

#### Phygadeuon cylindraceus

Ruthe, 1859

B232D894-EAD2-501E-95CD-6ACB73F49A7B

##### Notes

Mentioned by Horstmann (1970) as a hibernator, but the context is unclear.

**Status**: Confirmed.

**Records**: Germany, ca. 1970.

**Sources**: Horstmann (1970).

#### Phygadeuon dimidiatus

Thomson, 1884

9F379361-1E02-5E23-BE51-1792656EB19B

##### Notes

Collected in the winter 1922-1923, the hibernaculum type is unclear.

**Status**: Unverified

**Records**: United Kingdom, 1922-1923.

**Sources**: Hancock (1925).

#### Phygadeuon dubius

Ruthe, 1859

3A18DBE5-D54A-5B68-9DAB-3F608FE1E727

##### Notes

Collected in grass tufts.

**Status**: Unverified.

**Records**: United Kingdom, 22.XI.1923.

**Hibernacula**: LV (1).

**Sources**: Hancock (1925).

#### Phygadeuon fumator

Gravenhorst, 1829

E4C5E58C-AEC8-5459-B1EB-A9B6A5F8F1CC

##### Notes

Morley (1907) mentions this species is very common at grass tussocks, having collected 20 specimens in one hour. Two additional uncertain records exist on GBIF, one from Germany (11.II.2014) and one from the United Kingdom (10.XI.1986).

**Status**: Confirmed.

**Records**: Ireland (1), United Kingdom (1).

**First record**: ca. 1904 (Morley (1907), United Kingdom).

**Hibernacula**: LV (20), M (1).

**Sources**: Morley (1907), Johnson (1920).

#### Phygadeuon infelix

Dalla Torre, 1901

37749482-904E-5A88-86BE-DE63A23B7131

##### Notes

None.

**Status**: Unverified.

**Records**: United Kingdom, 10.XI.1922.

**Hibernacula**: LV (1).

**Sources**: Hancock (1923) [*Phygadeuon inflatus* Thoms.].

#### Phygadeuon leucostigmus

Gravenhorst, 1829

F91912EA-406B-5DD9-B27C-564F89B2E56A

##### Notes

Originally found by Hancock (1923), confirmed in Germany by Bauer (1984) and more recently in the United Kingdom (Pers. obs). All specimens were found in grass tussocks.

**Status**: Confirmed.

**Records**: Germany (1), United Kingdom (2).

**First record**: 10.III.1923 (Hancock (1923), United Kingdom).

**Hibernacula**: LV (3).

**Sources**: Hancock (1923) [*Phygadeuon leucostigma* Grav.], Bauer (1984); Unpublished: Pers. obs.

#### Phygadeuon nanus

(Gravenhorst, 1829)

B0D4D4F4-0233-5481-83D3-3D6DE63540F4

##### Notes

The context of this finding is unclear, but it is first reported by Rasnitsyn (1964) from the Moscow Region.

**Status**: Unverified.

**Records**: Russia, 1959-1964.

**Sources**: Rasnitsyn (1964) [*Hemiteles nanus* Grav.].

#### Phygadeuon trichops

Thomson, 1884

950EC4B7-DE49-555B-A346-631E8647F9EE

##### Notes

The *Phygadeuon* species most often found in grass tussocks.

**Status**: Confirmed.

**Records**: Belgium (1)*, Germany (1), United Kingdom (35).

**First record**: 10.XI.1922 (Hancock (1923), United Kingdom).

**Hibernacula**: DV (1), LV (72).

**Sources**: Hancock (1923) and Hancock (1925) [*Phygadeuon ocularis* Thoms.], Bauer (1984); Unpublished: Coll. RBINS, Pers. obs.

#### Phygadeuon varicornis

(Gravenhorst, 1829)

73E74473-43DA-5BE4-9F1B-FEF5BDAB25B4

##### Notes

None.

**Status**: Unverified.

**Records**: United Kingdom, ca. 1908.

**Hibernacula**: LV (1).

**Sources**: Morley (1907).

#### Phygadeuon vexator

(Thunberg, 1824)

C32FD8EE-3590-5A41-B909-F4A8E322A676

##### Notes

This species has been reported below the bark of a fence post, but it is unclear when this happened and if hibernation would be a possibility.

**Status**: Unverified.

**Records**: Germany, undated.

**Sources**: Horstmann (1970).

#### Thaumatogelis audax

(Olivier, 1792)

58FFDEDE-E0FA-505C-A4C1-A711C2052720

##### Notes

Very similar to *T. sylvicola*, identification needs to be done carefully. The third antennal segment is usually longer and the frons has no distinct punctures (Schwarz 2001).

**Status**: Confirmed.

**Records**: Austria (1), Sweden (1)*, the Netherlands (2)*, United Kingdom (2).

**First record**: 22.I.1923 (Hancock (1923), United Kingdom).

**Hibernacula**: DV (1), LV (4), M (1).

**Sources**: Hancock (1923) and Hancock (1925) [*Pezomachus zonatus* Forst.], Schwarz (2001); Unpublished: Artportalen.

**Also mentioned in**: Schwarz and Shaw (2000).

#### Thaumatogelis femoralis

(Brischke, 1881)

B94A8441-2B72-5342-89F4-4AF8AE6A7D4F

##### Notes

Hypothesised to hibernate by Schwarz (2001), now confirmed by one finding in Denmark. It was found sieving litter.

**Status**: Confirmed*.

**Records**: Denmark, 7.I.2017**.

**Sources**: Naturbasen.

**Hibernacula**: DV (1).

**Also mentioned in**: Schwarz (2001).

#### Thaumatogelis innoxius

Schwarz, 2001

8DC258C4-E6CB-5877-9344-AD521FF937B4

##### Notes

Several other specimens in Schwarz (2001) were probably found hibernating, notably the specimen collected in Hungary (8.I.1927), but this remains uncertain.

**Status**: Confirmed.

**Records**: Austria (1), United Kingdom (2).

**First record**: 13.X.1972 (Schwarz (2001), Austria).

**Hibernacula**: DV (2), LV (1).

**Sources**: Schwarz (1998) [*Thaumatogelis mingetshauricus* Bogac.], Schwarz (2001); Unpublished: Pers. obs.

#### Thaumatogelis lichtensteini

(Pfankuch, 1913)

8EDD2402-C17F-5CCD-9855-A75522ECDB27

##### Notes

Based on the ecology and phenology, Schwarz (2001) suggests it is likely that females hibernate.

**Status**: Unverified.

**Sources**: Schwarz (2001).

#### Thaumatogelis neesii

(Förster, 1850)

DEAF3E97-411A-5A9B-BAF1-B9D0D2CDD448

##### Notes

None.

**Status**: Confirmed.

**Records**: Austria (2), Belgium (1)**, Sweden (1)*.

**First record**: Ca. 1998 (Schwarz (1998), Austria).

**Hibernacula**: DV (2), M (2).

**Sources**: Schwarz (1998); Unpublished: Artportalen, Coll. RBINS.

**Also mentioned in**: Schwarz (2001).

#### Thaumatogelis sylvicola

(Förster, 1850)

2B89DA72-31BF-55CE-85B6-218CAADB61B5

##### Notes

Similar to *T. audax*. Hypothesised to hibernate by Schwarz (2001), recently confirmed by one finding in the Netherlands, in litter. One type specimen from France (26.XII.1887) was possibly found hibernating, but this is unsure (GBIF).

**Status**: Confirmed*.

**Records**: The Netherlands, 7.I.2024**.

**Hibernacula**: DV (1).

**Sources**: Observation.org.

**Also mentioned in**: Schwarz (2001).

#### Thaumatogelis vulpinus

(Gravenhorst, 1815)

070810C2-A1E2-5860-B3D6-2CEFA514BEA6

##### Notes

One specimen was found in moss in February (Schwarz 2001), which confirms that females hibernate (Schwarz 1998).

**Status**: Confirmed.

**Records**: Austria, ca. 2001.

**Hibernacula**: M (1).

**Sources**: Schwarz (2001).

**Also mentioned in**: Schwarz (1998).

#### Theroscopus esenbeckii

(Gravenhorst, 1815)

60EED1DC-3903-5256-AAFC-534FB379D245

##### Notes

None.

**Status**: Confirmed.

**Records**: Ireland (2), Russia (2).

**Hibernacula**: M (2), U (2).

**Sources**: Johnson (1920) [*Hemiteles subzonatus* Gr.], Rasnitsyn (1964) [*H. inaequalis* Först.].

#### Theroscopus hemipteron

(Riche, 1791)

B6954AAE-E954-5FF0-BD67-CBB5B0081497

##### Notes

This species has been reported as found below the bark of a fence post, but it is unclear when in the season this happened and if hibernation would be a possibility.

**Status**: Unverified.

**Records**: Germany, undated.

**Sources**: Horstmann (1970) [*Theroscopus hemipterus*].

#### Theroscopus ochrogaster

(Thomson, 1888)

FFC6B169-A2C2-5F7E-99D8-1039C0062498

##### Notes

One specimen was discovered in a rush tussock in the United Kingdom.

**Status**: Confirmed*.

**Records**: United Kingdom, 26.VIII.2020.

**Hibernacula**: LV (1).

**Sources**: Pers. obs.

#### Theroscopus pedestris

(Fabricius, 1775)

D8F09FF1-63ED-5515-B17F-072E96665AB7

##### Notes

Present in a variety of hibernacula, mostly litter and grass tussocks. Remarkably common in Denmark.

**Status**: Confirmed.

**Records**: Belgium (2), Denmark (5)*, Germany (2), Norway (7)*, the Netherlands (1)*, United Kingdom (3).

**First record**: ca. 1904 (Morley (1907), United Kingdom).

**Hibernacula**: DV (8), LV (4), M (1), U (7).

**Sources**: Morley (1907), Anonymous 1909 [*Hemiteles pedestris* F.], Bauer (1984), Sebald et al. (2000); Unpublished: Naturbasen, Obsevation.org, Pers. obs.

#### Xenolytus bitinctus

(Gmelin, 1790)

7AE6795E-FABE-5F5D-B216-791BBDA46EF5

##### Notes

None.

**Status**: Unverified.

**Records**: Romania, 21.XI.1960.

**Hibernacula**: C (1).

**Sources**: Decou and Decou (1961).

#### Pimplinae

6C424A29-4B4D-59F0-B931-540B6A5D4AF6

##### Notes

Some species of Pimplinae, especially those associated with Coleoptera and Lepidoptera, are known (facultative) hibernators, although the majority still overwinter as prepupae (Broad et al. 2018). It is noteworthy that no certain hibernators are known from species with spiders as a host.

#### Ephialtini

42628397-1D21-5ADC-8FF8-39AF063971D8

#### Acrodactyla polita

(Förster, 1871)

C0DABA15-8A75-50D0-BB87-1C75A13B649A

##### Notes

As species in this genus are associated with spiders, this finding will be coincidental and will be more related to the presence of the host than to any form of diapause.

**Status**: Incorrect.

**Records**: Romania, 9.V.1960.

**Sources**: Decou and Decou (1961) [*Symphylus politus* Först.].

#### Endromopoda detrita

(Holmgren, 1860)

004ADEE8-31A7-51FB-8E11-328ACA5556F7

##### Notes

The biology of this species is well known. *Endromopoda* spp. are mostly associated with concealed hosts in Poaceae stems (Broad et al. 2018). This renders hibernation rather unlikely, even when Hinz and Kreissl (1993) allegedly found a specimen below bark (in May).

**Status**: Incorrect.

**Records**: Austria, 14.V.1992.

**Sources**: Hinz and Kreissl (1993) [*Scambus detritus* Hlgr.].

#### Exeristes ruficollis

(Gravenhorst, 1829)

2D0A9573-4001-5BBD-9A62-B96380485D40

##### Notes

Hancock (1925) mentions G.T. Lyle has personally told him he himself had beaten five specimens from conifers. However, there is no exact date available and more context is unknown. They are parasitoids of tortricids in pines (Fitton et al. 1988).

**Status**: Confirmed.

**Records**: United Kingdom, ca. 1925.

**Hibernacula**: LT (5).

**Sources**: Hancock (1925) [*Pimpla ruficollis* Grav.].

**Also mentioned in**: Fitton et al. (1988).

#### Fredegunda diluta

(Ratzeburg, 1852)

15778425-D2A5-5887-A77F-DCDC054D069A

##### Notes

Fitton et al. (1988) suppose this species hibernates, but until now, no field observations confirm this hypothesis. Very exceptionally, specimens have been observed late in the year on the leaf axil of reed stems, but never really in hibernaculum.

**Status**: Unverified.

**Records**: United Kingdom, ca. 1988.

**Sources**: Fitton et al. (1988).

#### Scambus calobatus

(Gravenhorst, 1829)

D2C98512-1545-5410-A91E-C119DF262CD3

##### Notes

This species is included in Rasnitsyn (1959), without further context. The ecology and taxonomy of the *Scambus calobatus*-gr. has been well studied in Shaw et al. (2011). Females oviposit in autumn and the new generation emerges early in the subsequent year (in acorns). Although it is possible that this species concept contains cryptic species, this record can no longer be checked.

**Status**: Incorrect.

**Records**: Russia, 1951-1958.

**Sources**: Rasnitsyn (1959) [*Epiurus ventricosa* Tschek].

#### Scambus elegans

(Woldstedt, 1877)

A2D43D8B-245E-536E-835C-F7406ACFFEEC

##### Notes

Similar to *S. pomorum*, but with the propodeum evenly rounded in profile and a higher ovipositor-hind tibia index (Fitton et al. 1988). This species is exclusively found in evergreen trees.

**Status**: Confirmed.

**Records**: Denmark (1)**, the Netherlands (1)*, United Kingdom (3).

**First record**: Ca. 1988 (Fitton et al. (1988), United Kingdom).

**Hibernacula**: LT (4), U (1).

**Sources**: Fitton et al. (1988); Unpublished: iRecord, Naturbasen, Observation.org

#### Scambus pomorum

(Ratzeburg, 1848)

3BB60C50-469D-5CAD-960F-DED6FD3A948C

##### Notes

Similar to *S. elegans*. The life cycle of this univoltine species is well described by Zijp and Blommers (2002). Found also exclusively hibernating on evergreen trees.

**Status**: Confirmed.

**Records**: The Netherlands (1), United Kingdom (7).

**First record**: 31.XII.1923 (Hancock (1925), United Kingdom).

**Hibernacula**: LT (9).

**Sources**: Hancock (1925) [*Pimpla pomorum* Ratz.], Zijp and Blommers (2002); Unpublished: iNaturalist.

**Also mentioned in**: Fitton et al. (1988), Shaw (2006), Verheyde and Quicke (2022).

#### Scambus vesicarius

(Ratzeburg, 1844)

C4D0A7DB-76EC-5084-8083-DBFC1CD81A81

##### Notes

It is unclear under which circumstances this species was listed by Rasnitsyn. The species is not easy to identify and no other reports are known (Shaw 2006).

**Status**: Unverified.

**Records**: Russia, 1951-1958.

**Sources**: Rasnitsyn (1959) [*Epiurus vesicaria* Rtzb.].

#### Schizopyga circulator

(Panzer, 1800)

F0400B14-A4B5-5B87-A264-6560A33B47A6

##### Notes

One remarkable finding of this species in a *Juncus effusus* tussock, October 2020. As this species lives in wetlands and has a plurivoltine phenology, being active throughout the entire year more or less, it is not clear if this species was still actively searching for hosts, was in a state of quiescence or, indeed, was looking for a hibernaculum. If this were the case, hibernation could be facultative, but at this stage, it is more likely the wasp was still searching for hosts. Fitton et al. (1988) mentions the larvae overwinter on the subadult hosts, which makes adult hibernation less likely.

**Status**: Unverified.

**Records**: United Kingdom, 18.X.2020.

**Sources**: Pers. obs.

#### Pimplini

93E2FAA7-2E16-5C5A-A996-418D07919E6B

#### Apechthis quadridentata

(Thomson, 1877)

3DF9C893-4D33-51B4-B689-38A04CBCD469

##### Notes

Mentioned between brackets. The context is otherwise unclear. No other hibernators in this genus are known.

**Status**: Incorrect.

**Records**: France, 20.XI.1976.

**Sources**: Valemberg (1976b).

#### Itoplectis maculator

(Fabricius, 1775)

3F66C194-42D8-5E92-8BF5-044EE298367C

##### Notes

Common species throughout Europe, often found hibernating in evergreen trees or shrubs. Although it is mainly specialised in this niche, sometimes it shows slightly more opportunistic behaviour. Hancock (1925) mentions that it can also be found on the dead leaves (hanging) of beech and oak trees. In Germany, one specimen was found hibernating on the pillar of a bridge (with a conifer nearby).

**Status**: Confirmed

**Records**: Belgium (3), Denmark (1)*, France (1)*, Germany (2), the Netherlands (31), United Kingdom (25).

**First record**: III.1923 (Hancock (1925), United Kingdom).

**Hibernacula**: B (1), LT (41), LV (28), U (3).

**Sources**: Hancock (1925) [*Pimpla maculator* Fabr.], Bogenschütz (1965), Cole (1967), Verheyde and Quicke (2022); Unpublished: iNaturalist, Naturbasen, Observation.org.

**Also mentioned in**: Fitton et al. (1988), Shaw (2009).

#### Pimpla contemplator

(Muller, 1776)

A4AB8A1E-4848-55A1-BCA2-C4BE340393F3

##### Notes

Similar to *P. spuria* (Fitton et al. 1988). It is unclear on what basis this species is included in the list of Rasnitsyn (1964), as he erroneously refers to Kulagin (1886).

**Status**: Unverified.

**Records**: Russia, ca. 1886.

**Sources**: Kulagin (1886), Rasnitsyn (1964).

#### Pimpla spuria

Gravenhorst, 1829

5D506BE4-C320-5EA1-965A-180361EBAF92

##### Notes

Similar to *P. contemplator*. Hancock (1925) mentions he found one specimen on dead oak leaves, hanging on a tree. As the hibernation strategies of this genus are complex, it is difficult to interpret this record conclusively. However, the ecology of this species is rather well known. Both *P. contemplator* and *P. spuria* are bivoltine and hibernate as prepupae (Fitton et al. 1988).

**Status**: Unverified.

**Records**: United Kingdom, 1.IV.1924.

**Sources**: Hancock (1925).

#### Pimpla turionellae

(Linnaeus, 1758)

54841655-35D1-5756-A218-5BD729D96455

##### Notes

*P. turionellae* has also been considered a hibernator, but some authors presumed that this behaviour only occurred facultatively (Fitton et al. 1988). There are several strong indications this indeed is the case. *P. turionellae* is bivoltine and it has a wide host range. It is common in some well-monitored countries like Belgium and the Netherlands, but has not or only very rarely been found in hibernation there. In other countries the opposite is true. The observations are remarkably high in countries with colder regions, for example, Finland and Russia. Hibernation possibly is a facultative strategy here, induced by temperature or host usage or a combination of both. One uncertain hibernation record exists for Sweden (Artportalen).

**Status**: Confirmed.

**Records**: Denmark (2)*, Finland (2)*, France (1), Germany (2), Russia (16), the Netherlands (4)*, United Kingdom (2).

**First record**: Ca. 1886 (Kulagin (1886), Russia).

**Hibernacula**: B (1), DT (101), LT (1), LV (1), O (1), U (3).

**Sources**: Kulagin (1886) [*Pimpla turionella* Lin.], Seyrig (1923) [*P. examinator* L.], Jackson (1937) [*P. examinator* F.], Rasnitsyn (1959) and Rasnitsyn (1964) [*P. turionella* L.], Glen and Curtis (1978), Verheyde and Quicke (2022); Unpublished: Laji.fi, iNaturalist, Naturbasen, Observation.org, Pers. obs. (J. Paappanen).

**Also mentioned in**: Constantineanu (1929b), Fitton et al. (1988).

#### Tryphoninae

0CCE8CC8-B890-596D-94FB-F5584C41D211

##### Notes

Similar to Ctenopelmatinae, the hosts are mainly sawflies in this subfamily (Broad et al. 2018). Equally, so far, no certain hibernation records exist. However, we have some uncertain hibernation records of species which use Lepidoptera as host. Not discussed below are several records of *Phytodietus* spp., reported from conifers in November and December.

#### Oedemopsini

9E4D3290-61E5-54FB-AA0B-7CEB2E4FFB00

#### Cladeutes discedens

(Woldstedt, 1874)

3B17A0FA-DE1B-586E-B7C0-2FC6B418AA47

##### Notes

There is one record of this species possibly in a hibernaculum, being beaten from shrubs in February. Another specimen was observed in December, although it disappeared a week later. So far, no certain records are known for the subfamily Tryphoninae and more evidence is needed here, even when both observations from the Netherlands make diapause likely.

**Status**: Unverified.

**Records**: The Netherlands, 25.II.2024**.

**Sources**: Observation.org.

**Hibernacula**: LV (1).

#### Thymaris niger

(Taschenberg, 1865)

94E95196-D373-5F8F-B101-5A6870843C2A

##### Notes

Morley (1907) mentions that he found a male ‘hibernating’ in moss. This is unlikely as males are unable to hibernate and rather demonstrates this individual just emerged.

**Status**: Incorrect.

**Records**: United Kingdom, 23.III.1895.

**Sources**: Morley (1907) [*Hemiteles niger* Tasch.].

**Hibernacula**: M (1).

#### Phytodietini

7B90AF36-CAD7-58A9-8EF7-710C3813554E

#### Netelia testacea

(Gravenhorst, 1829)

4FF421BE-3B29-5ED2-9D5D-0B9CDFA46B95

##### Notes

Sebald et al. (2000) include it, stating that the label in Bauer’s collection records the specimen as having been collected while overwintering in situ. Presumably, it was included solely on the base of collection date. The ecology of *Netelia* sp. is well known and, based on that, hibernation is very unlikely (Broad et al. 2018).

**Status**: Incorrect.

**Records**: Germany, 1980-2000.

**Sources**: Sebald et al. (2000).

## Discussion

Several authors have used the concepts of facultative and obligate diapause in the past (Zinnert 1969, Hinz 1983, Hinz 1991), while others appear to recognise these concepts without explicitly naming them (Fitton et al. 1988). While compiling this checklist, one of the challenges we encountered is determining how to distinguish between these types of diapause. A technical solution is provided by Hinz (1983), who observed how the ovivaria expand in non-hibernating species by artificially raising the temperature. In obligatory diapause, there will be no immediate reaction. Consequently, in the field, this means that individuals which spend a long period of time at their hibernaculum are more likely to be real hibernators. However, some species also appear to use facultative diapause (see below). Moreover, in plurivoltine species, there may be periods of quiescence, a very different phenomenon from a biochemical perspective, but similar in the field (Denlinger 2022). It is still not completely clear how these mechanisms function. Some researchers have demonstrated how diapause has a strong genetic basis and a high plasticity, at least for some species. It has been shown how, within populations of *Aphidius ervi* (Hymenoptera, Braconidae), living at the same longitude and latitude, there can be huge differences of individuals tending to hibernate or not (Tougeron et al. 2019).

By listing 340 confirmed species found in hibernacula from August to May in different countries in Europe, we demonstrated how duration can, indeed, be an important indication of obligate diapause. Another one can simply be the activity of the individual. While there can be a reaction (e.g. flying, crawling away very actively) in pre- and postdiapause phases, this reaction takes much more time during the deepest phase of diapause. Depending on the location, this deeper phase typically occurs between December and February and is clearly observable in the field. During this period, individuals tend to maintain more fixed positions, sometimes exhibiting specific physiological adaptations associated with this stage (Fig. 8). In a phase of quiescence (e.g. some Phygadeuontinae, questionably) tend to react more quickly and additionally, resume other mechanisms which are usually disabled in real diapause (e.g. feeding, mating, searching for a host etc.) (Denlinger 2022, Verheyde and Quicke 2022).

In the species' notes, we have discussed two plausible indicators of facultative diapause: the rarity of observing certain otherwise common species in hibernation and the occurence of multiple hibernation strategies within a single genus or even amongst populations of the same species. We now have sufficient evidence that the presence of multiple hibernation strategies within one genus is not uncommon in the subfamilies Cryptinae, Phygadeuontinae and Pimplinae, while it occurs more rarely at the species level - for example, in *Gelis areator* and possibly *Gelis kiesenwetteri* and *Gelis nigritulus* (Schwarz and Shaw 1999). There may also be a connection between this phenomenon and the use of Lepidoptera hosts, though this remains to be confirmed. Biogeography is another influencing factor, as noted by Tougeron et al. (2019). For instance, the Holarctic species *Enytus montanus* has been shown to hibernate in the Nearctic Region, but has not been observed doing so in the Western Palaearctic (Baltensweiler 1958). Similarly, *Pimpla turionellae* appears to hibernate as an adult much more frequently in (north)eastern Europe.

Regardless of the diapause state, the ongoing activity of many of these ichneumonids during the entire year (including winter) was one of the main reasons to refrain from using dates as criteria for diapause (Materials and Methods). Another reason is the fact that insects can be reared during winter months or may have been influenced in their growth by artificial conditions, for example, the heating of a building. In recent years, climate change has equally influenced the phenology of species (i.e. Haris et al. (2025)). The role of temperature is visible at height. While the (presumed) type specimens of *Bureschias balcanicus* were discovered hibernating in August 1935, at a height of over 1800 metres, an active female was observed nearby in October that same year, at a height of 1000 metres (Heinrich 1936). In the past, these factors all led to debatable presumptions, some of them contributing to the 18 species wrongly assumed to be hibernating and the 79 species yet to be verified.

**Figure 9.**
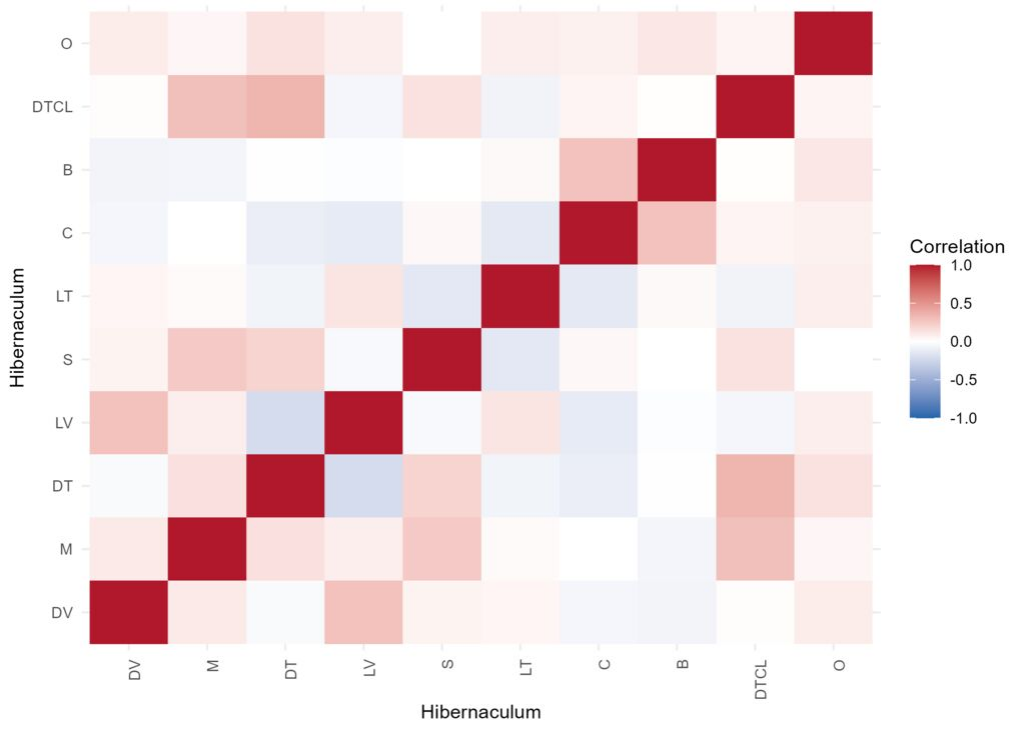
Correlation between the different hibernacula used by species.

Although it is obvious that hibernacula are vital to sustaining populations of hibernating species, the necessary size and extensiveness of these microhabitats at a local level are still very much unexplored, as is the limited distance individual species are able to cover in search of their hibernaculum. Our catalogue clearly demonstrated the presence of many individual specimens and species at different types of hibernating places. Some of them are highly specialised, such as those associated with caves. Up to 40% were found exclusively in this type of hibernaculum (Table 4). On the other hand, hibernaculum types that occur in close proximity tend to be used more frequently by the same species, as shown by the correlation analysis based on all records with known hibernacula (Fig. 9). For example, moss (M) and dead wood (DT), as well as dead vegetation (LV) and the litter layer (DV), show relatively high correlations, suggesting that these substrates are often used as substitutes by the same species. This is likely because such hibernacula frequently co-occur or share similar characteristics, for instance, moss growing on dead wood or dead pine branches associated with pine needles on the forest floor. In contrast, most hibernaculum types show little or no significant correlation, indicating that they are often used independently and, in many cases, may represent the primary or sole hibernaculum type for a given species. Lastly, specimens often cluster together and the number of individuals at one hibernaculum (e.g. a dead tree or cave) can be very high. This indeed demonstrates the importance of having a wide variety of hibernation resources. One tree more or less can make a great difference, especially in isolated places. Nature and forest management have been urged to take these different hibernation facilities into account when managing a certain area (Schebeck et al. 2024, Ferlauto and Burghardt 2025).

In the end, this all shows how a case-by-case biogeographical approach is essential to be able to say anything on the interesting biology and ecology of a certain species, which is very much entangled with its direct and indirect environment. It is clear how local research is still possible and interesting for faunistics, ecological (species) information and local nature management. We reported 29 species as hibernators for the first time. Moreover, 391 species were first reported in their given country as hibernators, of which 67 species are new to the local fauna altogether (Table 3). It is obvious that the biological mechanism of diapause is not uncommon. The 340 confirmed species represent approximately 4.75% of the total number of Darwin wasps occurring in the Western Palaearctic Region (Yu et al. 2016). There is a strong bias geographically (north-western Europe) and taxonomically (Phaeogenini and Phygadeuontinae with unresolved taxonomy). Many more hibernating species are expected to be found in the future.

## Figures and Tables

**Figure 2a.**
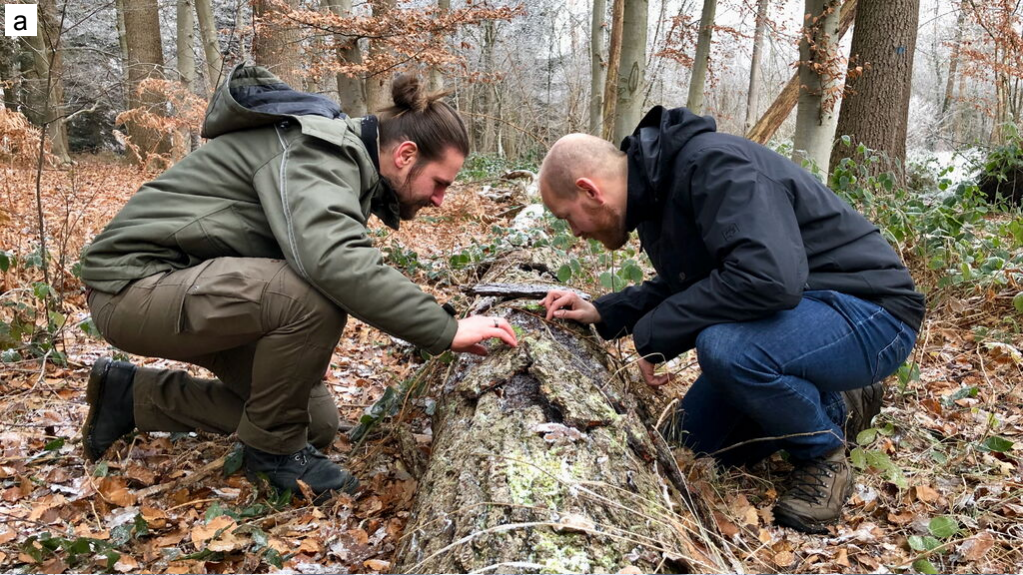
Searching below tree bark. 18.XII.2022, Ulvenhout (the Netherlands), photograph by Rob Buiter;

**Figure 2b.**
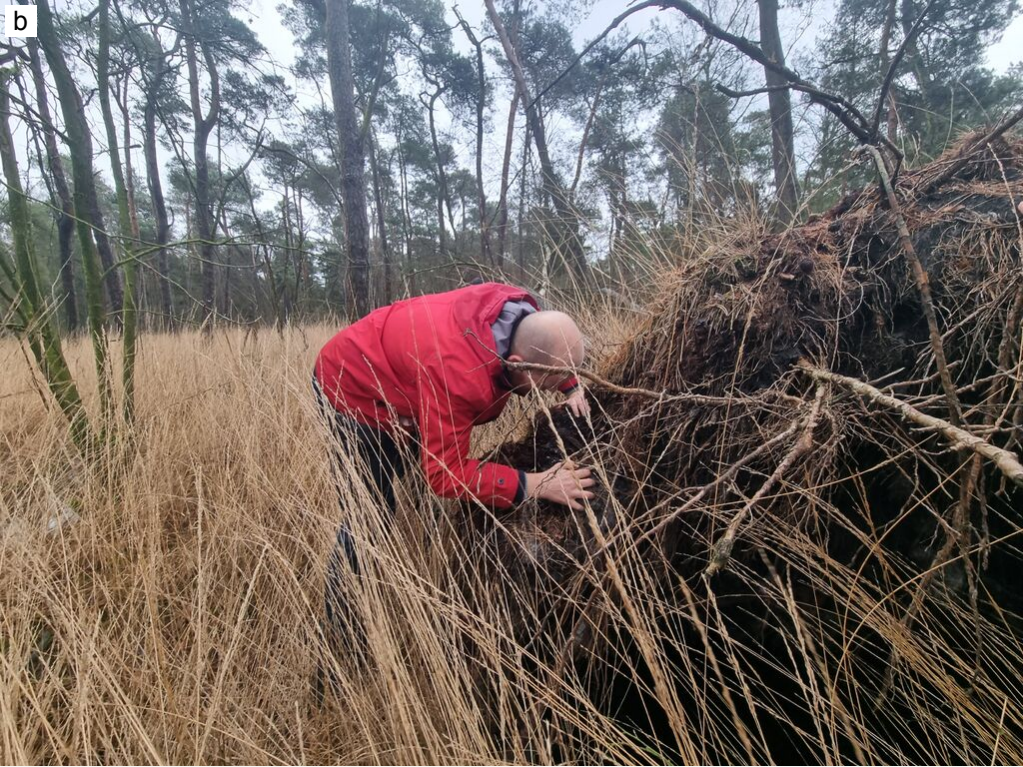
Searching between tree roots. 21.XII.2024, Kalmthout (Belgium), photograph by Karel Schoonvaere.

**Figure 3a.**
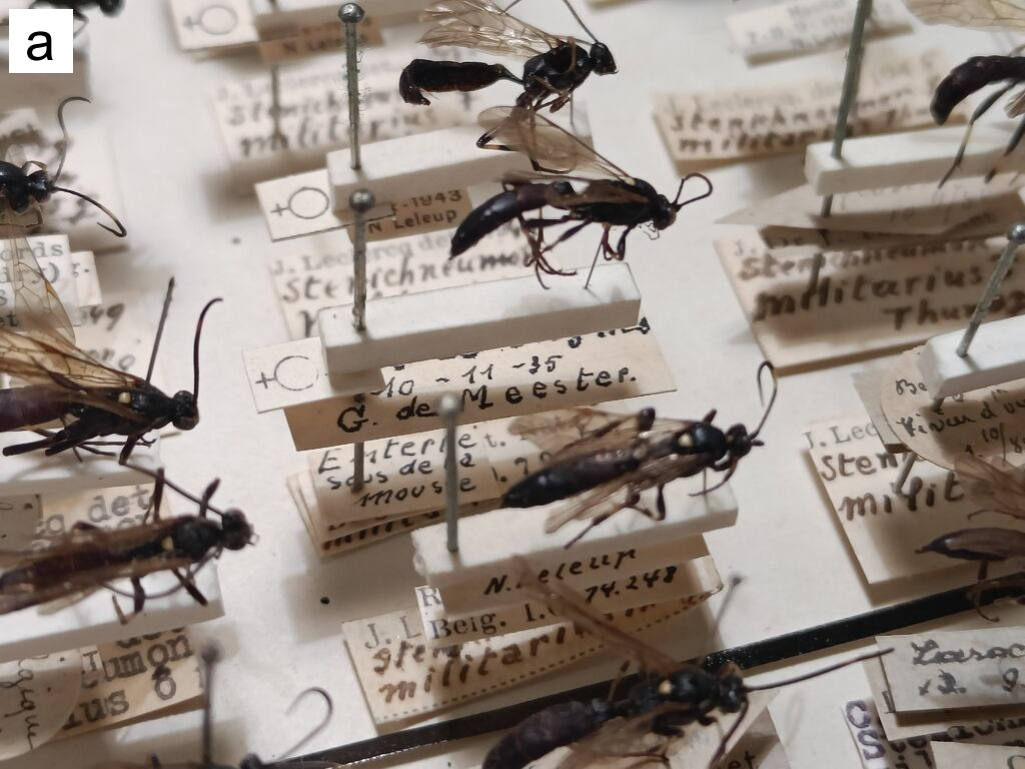
*Stenichneumon militarius*; 'sous de la mousse', collected in Belgium; collection RBINS, photograph by Fons Verheyde;

**Figure 3b.**
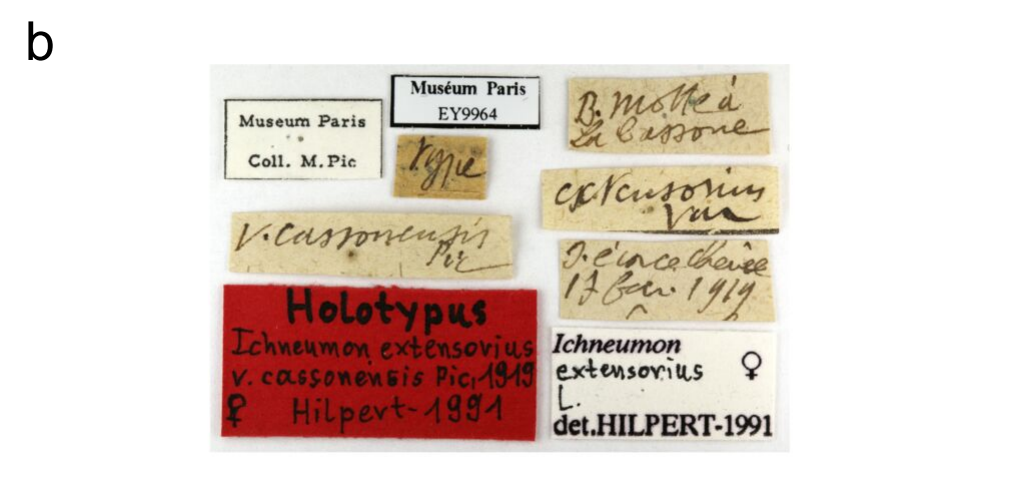
Holotype labels of Ichneumon extensorius subsp. cassonensis; 's[ous] écorce', collected in France; collection MNHN, photograph by Cristophe Hervé.

**Figure 5a.**
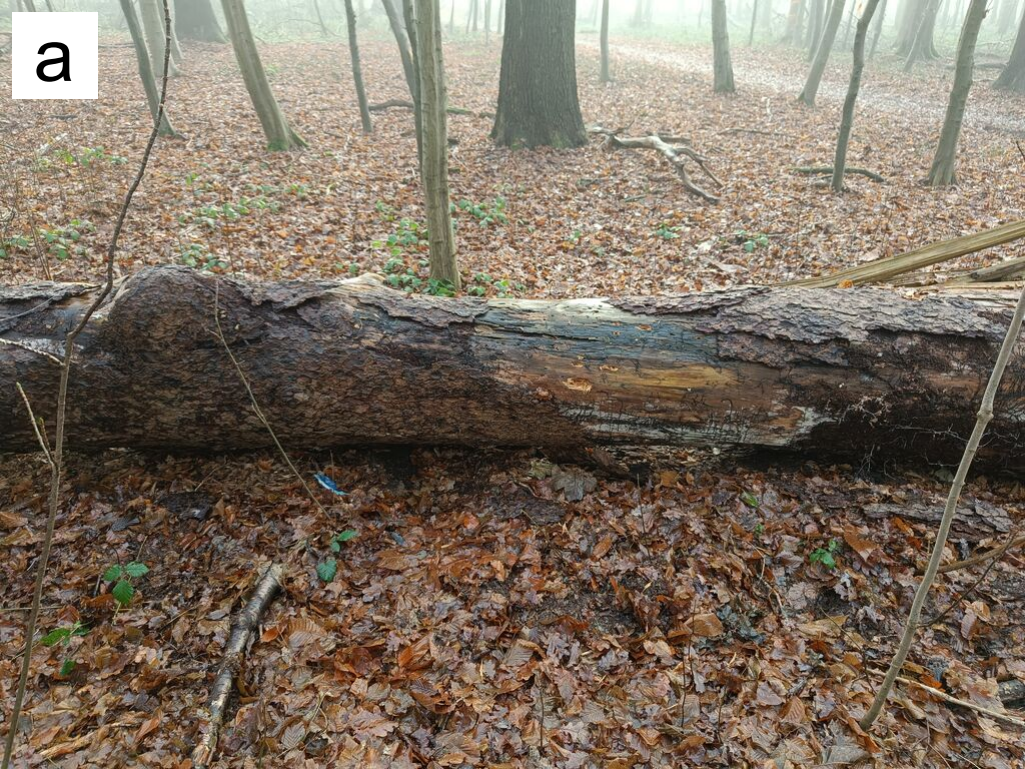
DT - 27.XII.2024, Valkenburg (the Netherlands), *Acer* sp., hibernaculum of *Ichneumon languidus*, photograph by Fons Verheyde;

**Figure 5b.**
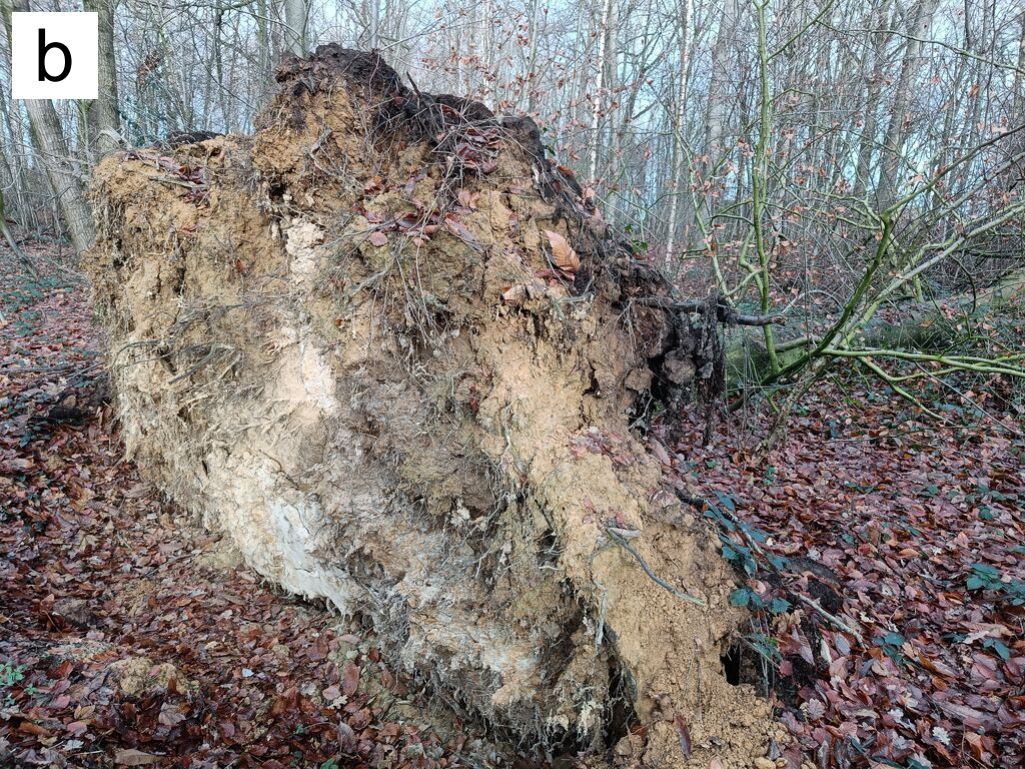
DTCL - 25.XII.2023, Elverdinge (Belgium), tree roots, hibernaculum of *Orthocentrus anomalus*, photograph by Fons Verheyde;

**Figure 5c.**
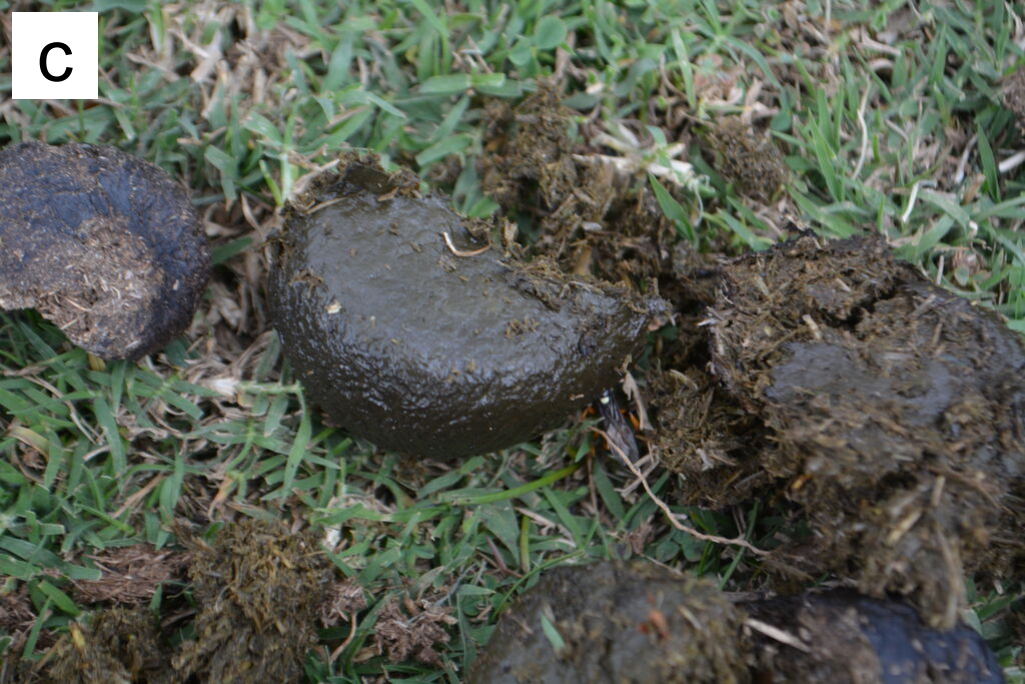
DV - 12.IX.2023, Nuro (Italy), dung balls, hibernaculum of *Eutanyacra picta*, photograph by Mario Oswald;

**Figure 5d.**
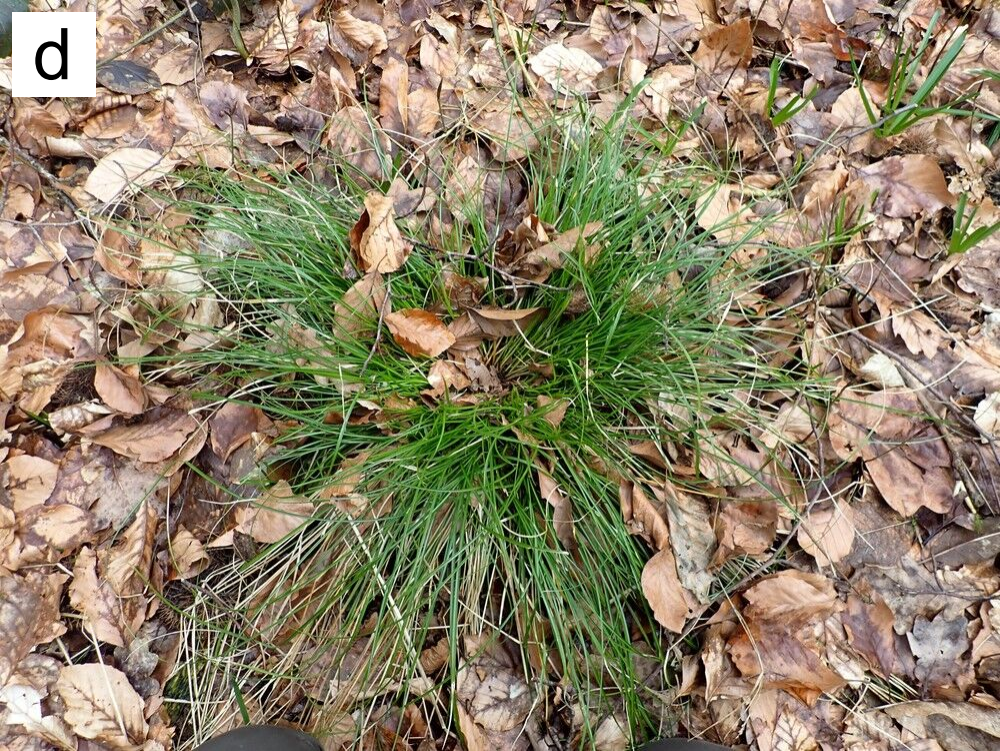
LV - 25.II.2024, Neigem (Belgium), *Deschampsia cespitosa*, hibernaculum of *Crytea erythraea*, photograph by Timon Boumon;

**Figure 5e.**
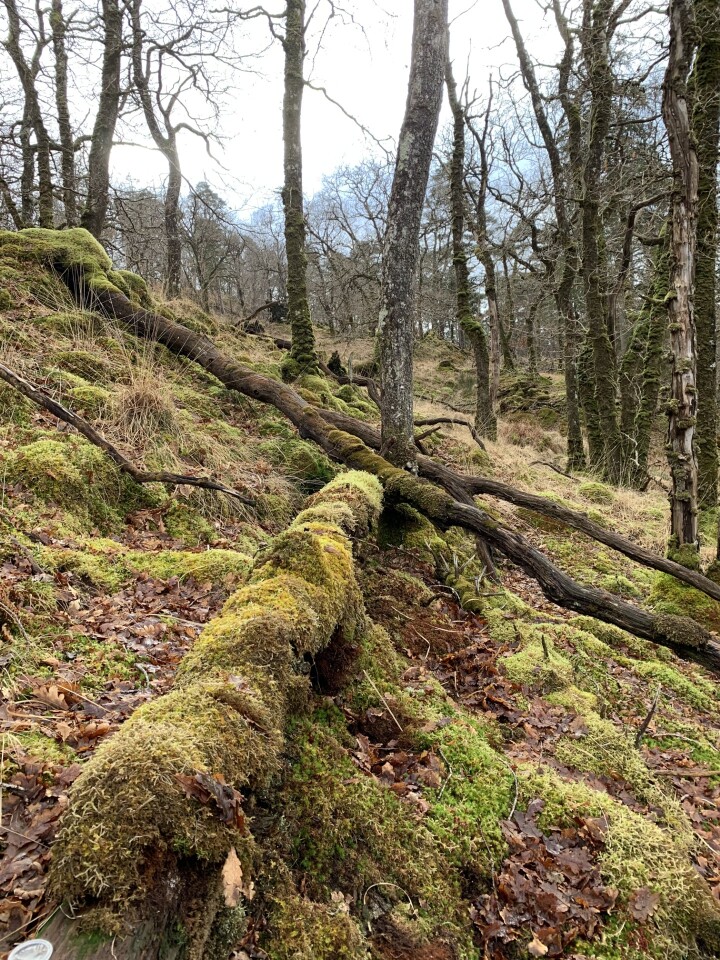
M - 7.X.2023, Cairngorm National Park (Scotland, United Kingdom), hibernaculum of *Ichneumon gracilentus* and *I. extensorius*, photograph by Al Cameron;

**Figure 5f.**
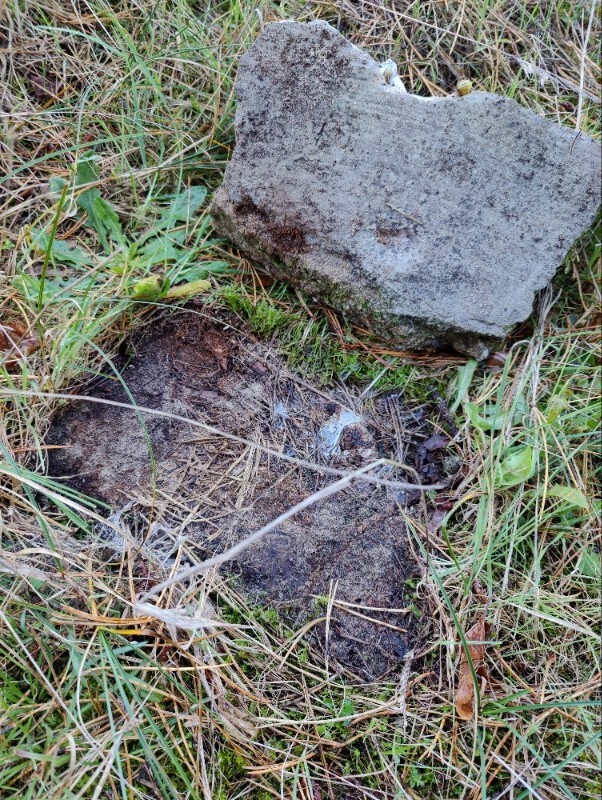
S - 15.X.2013, De Haan (Belgium), stone, hibernaculum of *Ichneumon sarcitorius*, photograph by Sebastiaan Stevens.

**Figure 6a.**
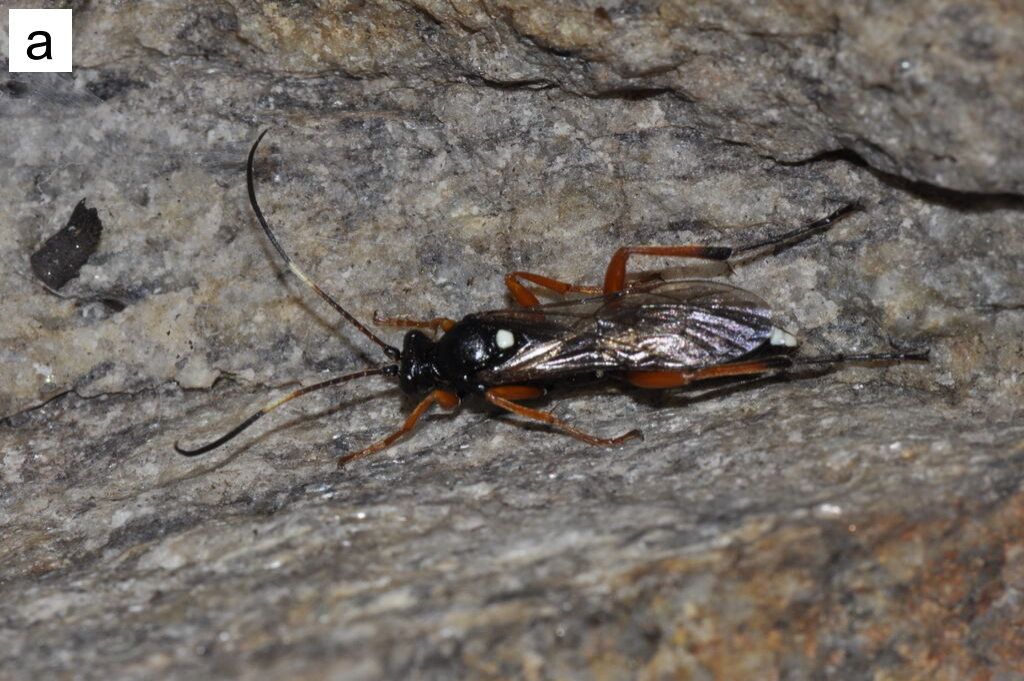
*Ichneumon quadrialbatus* - 9.XII.2012 (France), photograph by Pascal Dubois;

**Figure 6b.**
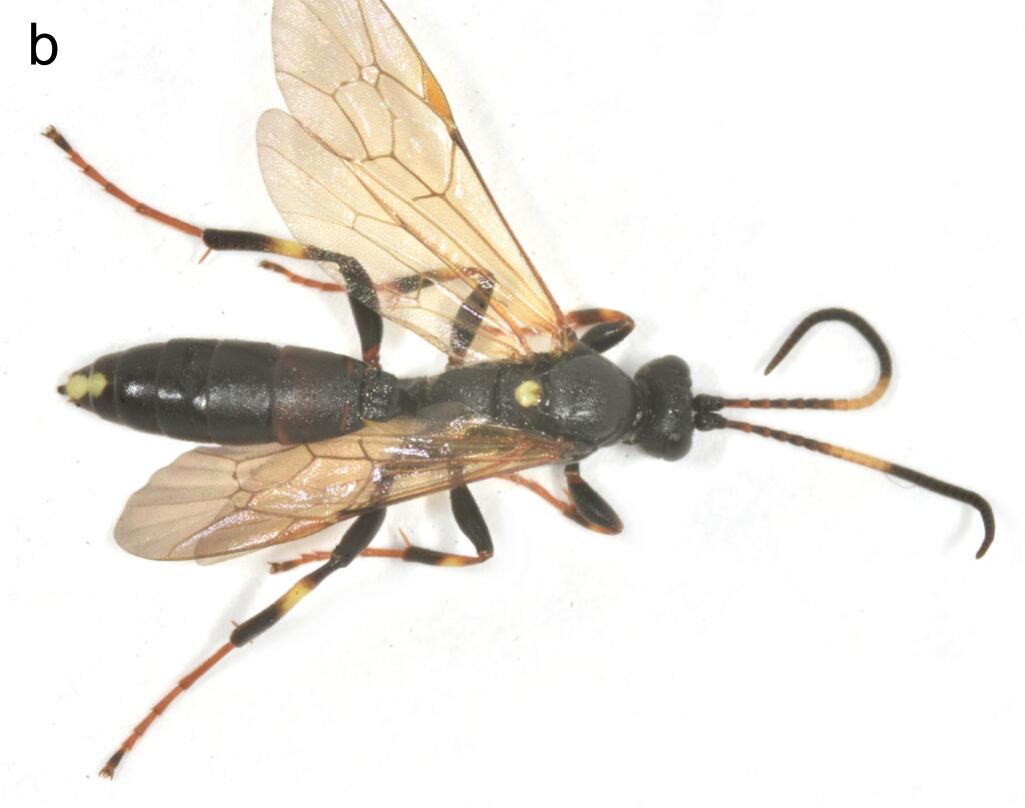
*Ichneumon rudolphi* - 13.X.2022 (Norway), photograph by Morten Angard Mjelde;

**Figure 6c.**
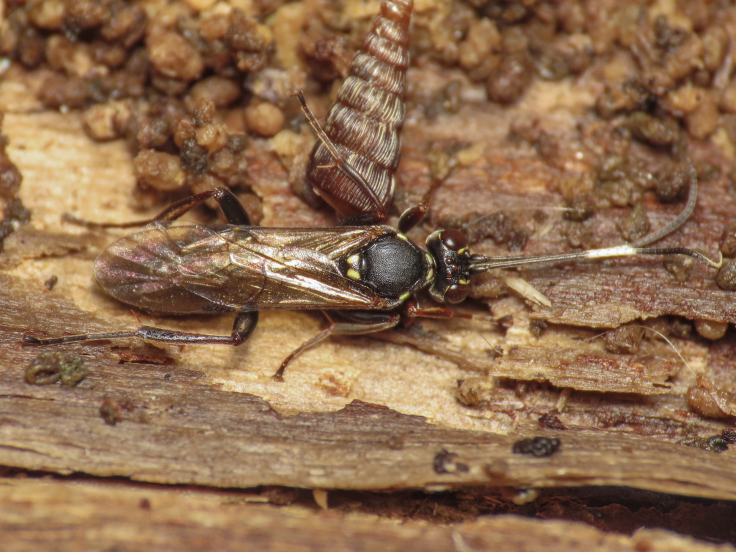
*Rhadinodonta flaviger* - 11.X.2024 (Italy), photograph by Emanuele Santarelli;

**Figure 6d.**
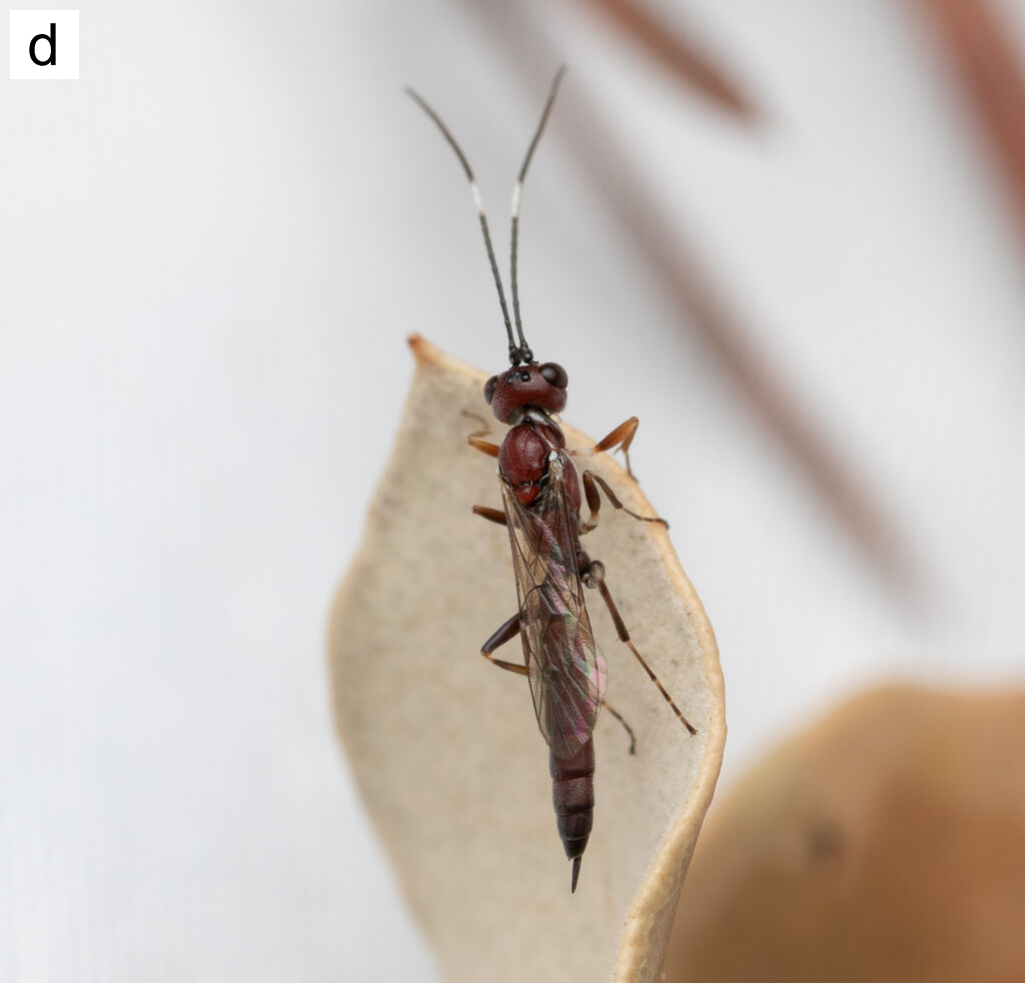
*Heterischnus pulchellus* - 15.I.2023 (France), photograph by Maxence Germain.

**Figure 8a.**
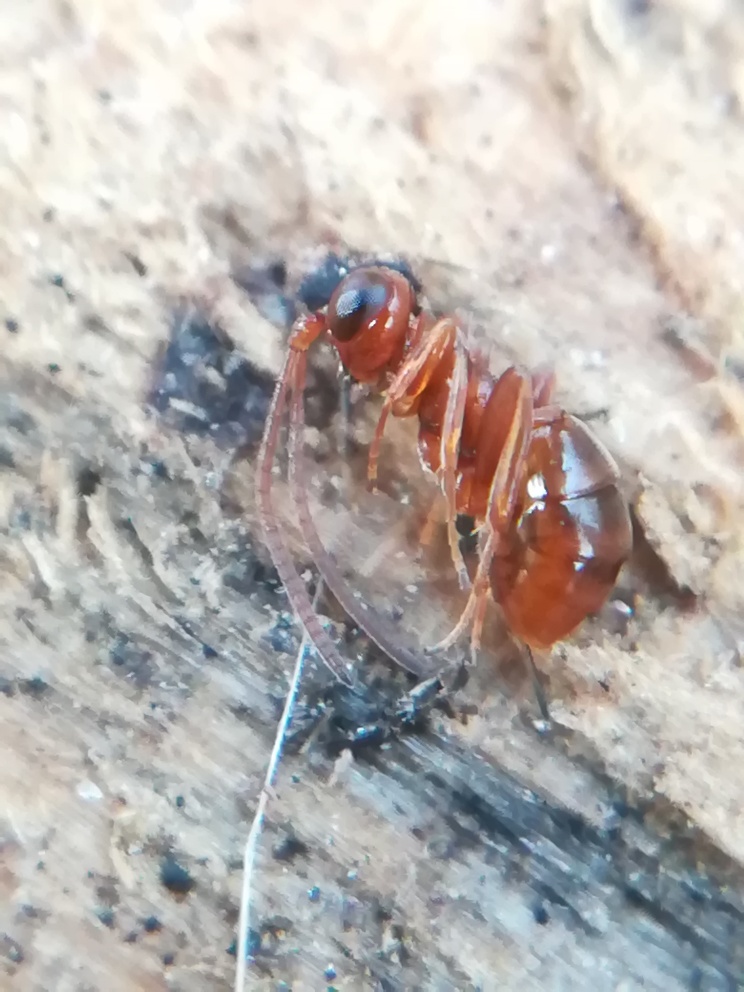
In hibernation, with the legs pulled up highly;

**Figure 8b.**
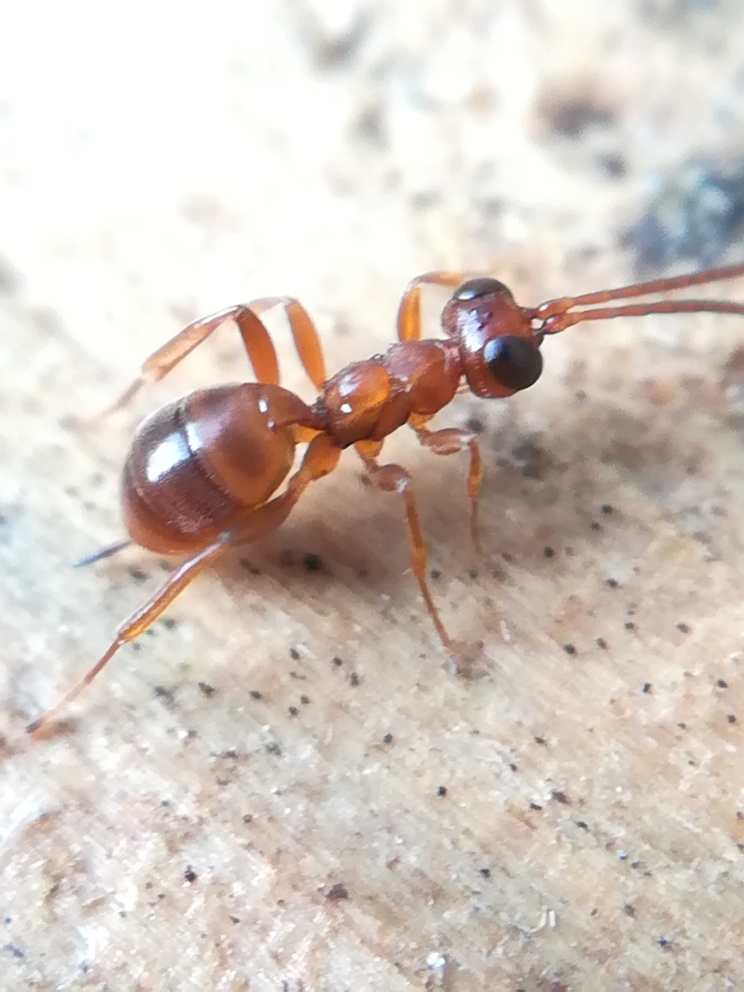
Awake after being disturbed (approx. 30 seconds later).

**Table 1.** Overview of data from citizen-science portals used in the catalogue.

**Name**	**Website link**	**Region**	**Number of records**
Arter DK	https://arter.dk/landing-page	Denmark	17
Artsobservasjoner	https://artsobservasjoner.no/	Norway	43
Artportalen	https://www.artportalen.se/	Sweden	43
BioPortal	https://www.bioportal.si/fotoseznam.php	Slovenia	2
Dabasdati	https://dabasdati.lv/lv	Latvia	21
Facebook (groups)	https://www.facebook.com/	Western Palaearctic	6
iNaturalist	https://www.inaturalist.org/	Western Palaearctic	861
insecte.org	https://insecte.org/	France	26
iRecord	https://irecord.org.uk/	United Kingdom	118
Laji.fi	https://laji.fi/en	Finland	3
Naturbasen	https://www.naturbasen.dk/	Denmark	163
Naturgucker	https://nabu-naturgucker.de/	Germany	40
Observation.org	https://observation.org/	Western Palaearctic	1168
UKRbin	https://www.ukrbin.com/	Ukraine	14

**Table 2.** Typology of hibernacula - as explained in Verheyde and Quicke (2022).

**Acronym**	**Examples of hibernacula niches mentioned in literature**	
B	Building, fortress, cellars	
C	Caves, limestone caves, quarries, grottoes, mines, holes, dens, Höhlen	
DT	Dead tree, below bark, sous écorce, sous arbre mort, unter Rinde, in Stubben, morschem Baumstumpf, Baumstämmen, onder schors, op dood hout, sub arborum cortice, veltet tre, fakéreg alatt	Fig. 5a
DTCL	Dead tree clay, roots, Wurzeltellern, wortelkluit, arbustorum radices	Fig. 5b
DV	Dead vegetation, tree litter, leaves, detritus, pine needles, sieving, in soil, balls of hay, compost, Komposstreu, strooisel, falevelek alatt, Laub	Fig. 5c
LT	Living tree, tree branches	
LV	Standing vegetation, shrubs, beating, grass tussock, Grasbüscheln, tarnisage, tussocking, bladrozet, hollow stem (standing)	Fig. 5d
M	Below moss, moss bed, sous mousse, unter Moos	Fig. 5e
O	Other (ant nest, fence posts, fungi, bee hotel, ..)	
S	Stone, Stein, Bajo una piedra	Fig. 5f
U	Unknown	
	Diapause, Overwintering, Überwinterung, hibernation, hivernation, зимовка	

**Table 3.** New country records of hibernating species mentioned in the catalogue.

Armenia	*Ichneumon stramentor*
Belarus	*Ichneumon bucculentus*, *Orgichneumon calcatorius*
Belgium	*Orthocentrus anomalus*, *Gelis hortensis*, *Thaumatogelis neesii*
Bulgaria	*Ichneumon stramentor*, *Ichneumon suspiciosus*
Croatia	*Stenichneumon culpator*
Czechia	*Ichneumon oblongus*, *Ichneumon str amentor*
Denmark	*Amblyteles armatorius*, *Aoplus defraudator*, *Chasmias paludator*, *Diphyus quadripunctorius*, *Hoplismenus terrificus*, *Ichneumon bucculentus*, *Ichneumon extensorius*, *Ichneumon gracilentus*, *Ichneumon inquinatus*, *Ichneumon oblongus*, *Ichneumon stramentarius*, *Ichneumon stramentor*, *Ichneumon suspiciosus*, *Ichneumon tuberculipes*, *Stenichneumon culpator*, *Stenichneumon militarius*, *Zanthojoppa lutea*, *Diadromus collaris*, *Heterischnus truncator*, *Thaumatogelis femoralis*, *Scambus elegans*
Estonia	*Stenichneumon culpator*
France	*Exephanes riesei*
Hungary	*Orgichneumon calcatorius*
Ireland	*Ichneumon stramentor*
Italy	*Hoplismenus pica*, *Ichneumon inquinatus*, *Orgichneumon calcatorius*, *Rhadinodonta flaviger*
Kosovo	*Ichneumon lugens*, *Rhadinodonta flaviger*
Latvia	*Thyrateles camelinus*
Lithuania	*Chasmias paludator*, *Diphyus raptorius*, *Diphyus salicatorius*, *Orgichneumon calcatorius*, *Rhadinodonta flaviger*, *Syspasis albiguttata*
Luxembourg	*Diphyus castanopyga*, *Diphyus raptorius*, *Eutanyacra crispatoria*, *Ichneumon bucculentus*, *Rhadinodonta flaviger*, *Zanthojoppa lutea*
Netherlands	*Cladeutes discedens*, *Orthocentrus anomalus*, *Thaumatogelis sylvicola*
Norway	*Ichneumon lugens*
Serbia	*Diphyus raptorius*
Slovakia	*Ichneumon lugens*
Sweden	*Chasmias paludator*, *Ichneumon minutorius*, *Ichneumon xanthorius*, *Lymantrichneumon disparis*
Ukraine	*Chasmias paludator*
UK	*Pseudoplatylabus violentus*

**Table 4.** Number of species recorded per hibernaculum and exclusivity per hibernaculum.

**Hibernaculum**	**Total species observed**	**Exclusive species (count)**	**Exclusive species (%)**
DT	159	40	25.16
M	124	15	12.10
LV	118	32	27.12
DV	67	9	13.43
S	55	4	7.27
DTCL	50	1	2.00
LT	40	9	22.50
C	31	12	38.71
O	16	1	6.25
B	11	1	9.09

## References

[B13364212] Aellen V., Strinati P. (1962). Nouveaux matériaux pour une faune cavernicole de la Suisse. Revue Suisse de Zoologie.

[B13364236] Aerts Wilhelm (1957). Die Schlupfwespen-(Ichneumoniden-) Fauna des Rheinlandes. Decheniana.

[B13364245] Arndt Walther (1921). Beitrag zur Kenntnis der Höhlenfauna: Ergebnis einer faunistischen Untersuchung der Höhlen Schlesiens. Zoologischer Anzeiger.

[B13364262] Athimus F. (1901). Beitrag zur Ichneumoniden-Fauna Belgiens. Allgemeine Zeitschrift für Entomologie.

[B13364337] Athimus F. (1901). Beitrag zur Ichneumoniden-Fauna Belgiens. Allgemene Zeitschrift für Entomologie.

[B13364347] Aubert Jacques-Félix (1959). Biologie d'un hyperparasite trimorphique du groupe de *Gelis
corruptor* Först. (Hym. Ichn.). Bulletin mensuel de la Société linnéenne de Lyon.

[B13364356] Aubert J. (1960). Les Ichneumonides des Pyrénées-Orientales. Vie et Milieu.

[B13364375] Baird Katty, Shaw Mark R. (2019). Overwintering behaviour of *Diphyus
quadripunctorius* (Müller) (Hymenoptera: Ichneumonidae, Ichneumoninae) in south-east Scotland. Entomologist's Monthly Magazine.

[B13364384] Baltensweiler Werner (1958). Zur Kenntnis der Parasiten des Grauen Lärchenwicklers (*Zeiraphera
griseana* Hübner) im Oberengadin: Ihre Biologie und Bedeutung während der Gradation von 1949 bis 195. Mitteilungen der Schweizerischen Anstalt für das forstliche Versuchswesen.

[B13364404] Bauer Rudolf (1961). Ichneumoniden aus Franken: Teil II (Hymenoptera: Ichneumonidae). Beiträge zur Entomologie = Contributions to Entomology.

[B13364447] Bauer Rudolf (1984). Die Überwinterung von Insekten mit besonderer Berücksichtigung der Ichneumoniden. Wissenschaftliche Beilage in Jahresbericht des Neuen Gymnasiums Nürnberg.

[B13364456] Bauer Rudolf (1999). Bemerkungen über die Ichneumoniden der Alpen mit einigen Neubeschreibungen (II. Teil) (Hymenoptera, Ichneumonidae, Ichneumoninae). Entomofauna: Zeitschrift für Entomologie.

[B13364465] Bauer Rudolf (2001). Bemerkungen über die Ichneumoniden der Alpen mit einigen Neubeschreibungen: Teil III (Hymenoptera, Ichneumonidae, Ichneumoninae). Entomofauna: Zeitschrift für Entomologie.

[B13719070] Bequaert Joseph (1909). Bouwstoffen voor de Hymenopteren-fauna van België [161-170]. Vlaamsch Natuur- en Geneeskundig Congres.

[B13364474] Beron Petar, Bechev Dimitar, Georgiev Dilian (2016). Faunistic diversity of Vrachanski Balkan Nature Park.

[B13366197] Berthoumieu Victor (1894). Ichneumonides d’Europe et de pays limitrophes: 1^er^ Tribu – Ichneumoniens, Section I [last part] + Section II. Annales de Société Entomologique de France.

[B13366206] Berthoumieu Victor (1895). Ichneumonides d’Europe et de pays limitrophes: 1^er^ Tribu – Ichneumoniens, Section II [last part] + Section III. Annales de Société Entomologique de France.

[B13366215] Berthoumieu Victor (1895). Ichneumonides d’Europe et de pays limitrophes: 1^er^ Tribu – Ichneumoniens, Section IV + [Remaining tribes and genera]. Annales de Société Entomologique de France.

[B13366224] Berthoumieu Victor (1896). Ichneumonides d’Europe et de pays limitrophes [Remaining tribes and genera]. Annales de Société Entomologique de France.

[B13364496] Bertolani Roberto, Manicardi Gian Carlo, Rebecchi Lorena (1994). Faunistic study in the karst complex of Frasassi (Genga, Italy). International Journal of Speleology.

[B13366233] Bogenschütz H. (1965). Untersuchungen über Parasiten des Eichenwicklers *Tortrix
viridana* L. III. Zur Phänologie einiger wichtiger Parasiten. Entomophaga.

[B13366242] Bokor Elemér (1921). A magyarhoni barlangok izeltlábui. Barlangkutatás.

[B13366251] Boyd D. O. (1935). *Chasmias
paludicola* Wesm. hibernating in burrow of *Megachile* (Hym.). Journal of the Society for British Entomology.

[B13398770] Broad Gavin, Shaw Mark, Fitton Michael (2018). Ichneumonid Wasps (Hymenoptera: Ichneumonidae): Their classification and biology.

[B13366350] Buresch I. (1934). Реферати и съобщения = Reports and announcements. Izvestiia na Bulgarskoto entomologichno druzhestvo = Mitteilungen der Bulgarischen Entomologischen Gesellschaft in Sofia.

[B13369367] Busch Theo (1951). *Anilasta
vulgaris* Tschek als Parasit des großen Kohlweißlings (*Pieris
brassicae* L.). Entomologische Zeitschrift.

[B13369376] Büttner Kurt (1932). Die Stollen, Bergwerke und Höhlen in der Umgebung von Zwickau und ihre Tierwelt: Nachtrag. Jahresbericht des Vereins für Naturkunde zu Zwickau i.S..

[B13369385] Canciani Giacomo, Cechini Riccardo (2023). Due nuove stazioni per *Diphyus
quadripunctorius* (O.F. Müller, 1776) (Hymenoptera, Ichneumonidae) nelle Prealpi Giulie e nella Tuscia viterbese. Atti del museo civico di storia naturale di Trieste.

[B13411272] Chacko M. J., Rao V. P. (1966). *Centeterus
alternecoloratus* Cushman? var., a pupal parasite of the graminaceous borers, *Chilo
partellus* (Swinhoe) and *Chilotraea
auricilia* (Dudgeon). Entomophaga.

[B13393722] Cole L. R. (1959). On a new species of *Syntretus* Förster (Hym., Braconidae) parasitic on an adult ichneumonid, with a description of the larva and notes on its life history and that of its host, *Phaeogenes
invisor* (Thunberg). Entomologist's Monthly Magazine.

[B13393731] Cole L. R. (1967). A study of the life‐cycles and hosts of some Ichneumonidae attacking pupae of the green oak‐leaf roller moth, *Tortrix
viridana* (L.) (Lepidoptera: Tortricidae) in England. Transactions of the Royal Entomological Society of London.

[B13449451] Cole L. R. (1979). Notes on the biology of *Ischnus
inquisitorius* (Müll.) (Hym, Ichneumonidae), an ectoparasitoid of tortricid pupae. Entomologists's Monthly Magazine.

[B13393740] Collart A. (1941). Études biospéologiques XXV (1). Ichneumonidae (Hymenoptera) de Transylvanie. Bulletin du Musée royal d'Histoire naturelle de Belgique.

[B13393762] Constantineanu Mihai I. (1929). Nouvelle contribution à la faune ichneumonologique de la Roumanie. Annales Scientifiques de l’Université de Jassy.

[B13393771] Constantineanu Mihai I. (1929). Contributions à l’étude des ichneumonides en Roumanie. Annales Scientifiques de l’Université de Jassy.

[B13394562] Constantineanu Mihai I. (1956). Specii de ichneumonide noi pentru fauna R.P.R.. Studii și cercetari științifice: Biologie și științe agricole.

[B13394587] Constantineanu Mihai I (1959). Familia Ichneumonidae, Subfamilia Ichneumoninae, Tribul Ichneumoninae Stenopneusticae. Fauna Republicii Populare Romîne Insecta.

[B13394633] Constantineanu RM (1969). Contribuţii la cunoaşterea hibernării ichneumonidelor adulte din arbori căzuţi din pădurea Birnova (jud. Iaşi). Studii si cercetari de biologie: Seria Zoologie = Romanian Journal of Biology: Zoology.

[B13394642] Constantineanu R. M. (1970). Hibernarea Ichneumonidelor adulte în arbori vii din pădurea Bârnova (jud. Iaşi). Studii şi Comunicări Ştiinţele Naturii.

[B13394669] Darvishnia Hamid, Esmaeili Hamid Reza, Sadeghi Saber, Riedel Matthias, Mohammadi-Khoramabadi Abbas (2018). *Exephanes
tauricus* Hinz, 2000 (Hym.: Ichneumonidae): A new record to Iranian cave fauna. Biharean Biologist.

[B13394681] Decou Anca, Decou Vasile (1961). Hyménoptères recueillis dans les grottes de Roumanie. Annales du Laboratoire souterrain de Han-sur-Lesse.

[B13397384] Denlinger David L. (2022). Insect diapause.

[B13394690] Dethier Michel (2007). Les invertébrés des carrières souterraines de craie du nord-est de la Belgique. Bulletin des Chercheurs de la Wallonie.

[B13394701] Diller Erich, Shaw Mark (2014). Western Palaearctic Oedicephalini and Phaeogenini (Hymenoptera: Ichneumonidae, Ichneumoninae) in the National Museums of Scotland, with distributional data including 28 species new to Britain, rearing records, and descriptions of two new species of *Aethecerus* Wesmael and one of *Diadromus* Wesmael. Entomologist's Gazette.

[B13394719] Dobat Klaus (1963). Beiträge zur Höhlenfauna der Provence. Die Höhle.

[B13394728] Dobat Klaus (1963). Die Fauna der Gutenberger Höhlen. Jahreshefte für Karst- und Höhlenkunde.

[B13394737] Dobat Klaus (1975). Die Höhlenfauna der Schwäbischen Alb mit Einschluß des Dinkelberges, des Schwarzwaldes und des Wutachgebietes. Jahreshefte der Gesellschaft für Naturkunde in Württemberg.

[B13394754] Dobat Klaus (1978). Die Höhlenfauna der Fränkischen Alb. Abhandlungen zur Karst- und Höhlenkunde.

[B13394773] Dundarova Heliana, Dedov Ivailo, Ljubomirov Toshko, Zhalov Alexey, Deltshev Christo, Ćalić J. (2018). Preliminary research on the cave fauna in three caves in Lurë Mountain (Albania) [139-144]. Proceedings of the 8th Symposium on karst protection.

[B13395545] Dvořák Libor (2002). Some invertebrates in cellars from the west Bohemia and Bohemian forest = Někteří bezobratlí živočichové sklepů na území západních Čech a Šumavy. Erica.

[B13395576] Fabbri Roberto, Poletti Katia (2015). Invertebrati delle cavità dei Gessi di Brisighella e Rontana. Memorie dell’Istituto Italiano di Speleologia.

[B13398723] Ferlauto Max, Burghardt Karin T. (2025). Removing autumn leaves in residential yards reduces the spring emergence of overwintering insects. Science of The Total Environment.

[B13395585] Fernández Toni Pérez, Ortíz-Sánchez F. Javier, Férnandez Toni Pérez, Ruiz Pérez Antonio (2013). Los invertebrados de hábitats subterráneos de Jaén.

[B13395609] Fernández Toni Pérez, Ruiz Antonio Pérez, Fernández Jesús Perez, Román Fátima García (2013). La cueva secreta del Sagreo (La Iruela, Jaén, Sur de Espana), una cavidad con historia en Jaén. Espeleo-Tema.

[B13395600] Fernández Toni Pérez, Ruiz Antonio Pérez (2014). Catálogo provisional de los invertebrados del sistema sima GESM-sima de la Luz (Tolox, Málaga). Gota a gota.

[B13401589] Fitton M. G., Shaw Mark, Gauld I. (1988). Pimpline Ichneumon-flies: Hymenoptera, Ichneumonidae (Pimplinae).

[B13546881] Institute Flanders Marine MarineRegions.org. www.marineregions.org.

[B13395618] Förster Arnold (1850). Monographie der Gattung *Pezomachus* Grv.. Archiv für Naturgeschichte.

[B13395627] Franciscolo Mario Enrico (1955). Fauna cavernicola del Savonese. Annali del Museo civico di storia naturale Giacomo Doria.

[B13395658] Frivaldszky Jánostól (1865). Adatok a magyarhoni barlangok faunájához. Mathematikai és Természettudományi Közlemények.

[B13395700] Glen D. M., Curtis D. E. (1978). Pupal parasites of codling moth. Mitteilungen aus der Biologischen Bundesanstalt für Land- und Forstwirtschaft.

[B13395728] Gokhman V. E. (1985). Chromosome sets in some Ichneumoninae (Hymenoptera, Ichneumonidae). Zoologichesky Zhurnal.

[B13395737] Gokhman V. E. (1987). Chromosomes in the Ichneumoninae (Hymenoptera, Ichneumonidae). Zoologichesky Zhurnal.

[B13454800] Gokhman V. E., Rasnitsyn A. (1988). Insects of the Moscow Province: Problems of Recording and Protection.

[B13395756] Gokhman V. E. (1989). Karyotypes of ichneumon flies of the *Tycherus
osculator* group (Hymenoptera, Ichneumonidae). Entomologicheskoe Obozrenie.

[B13395766] Gokhman V. E. (1990). Main trends of the karyotype evolution in Ichneumoninae (Hymenoptera, Ichneumonidae). Zoologichesky Zhurnal.

[B13717071] Gokhman V. E. (1990). Karyology and systematics of Ichneumoninae (Hymenoptera, Ichneumonidae).

[B13395775] Gokhman V. E. (1991). New species of Phaeogenini (Hymenoptera, Ichneumonidae) from the European part of the USSR. Zoologichesky Zhurnal.

[B13395784] Gokhman V. E. (1993). New data on the karyology of Ichneumonina (Hymenoptera, Ichneumonidae). Zoologichesky Zhurnal.

[B13400792] Gokhman V. E., Quicke D. (1995). The last twenty years of parasitic Hymenoptera karyology: An update and phylogenetic implications. Journal of Hymenoptera Research.

[B13395793] Gokhman V. E. (2003). Karyotypes of parasitic Hymenoptera: Evolution and implications for systematics and phylogeny.

[B13401561] Gokhman V. E., Mikhailenko A. P. (2008). Chromosomes of Ichneumonidae (Hymenoptera): New results and conclusions. Entomological Review.

[B13395801] Gokhman V. E. (2014). Overall results of the chromosomal study of parasitic waspsof the subfamily Ichneumoninae (Hymenoptera, Ichneumonidae). Proceedings of the Russian Entomological Society.

[B13395827] Gokhman V. E., Anokhin B. A., Kuznetsova V. G. (2014). Distribution of 18S rDNA sites and absence of the canonical TTAGG insect telomeric repeat in parasitoid Hymenoptera. Genetica.

[B13395845] Gravenhorst J. L.C. (1829). Ichneumonologia Europaea, Pars I.

[B13396193] Griepenburg W. (1933). Die Rentropshöhle bei Milspe in Westfalen. Mitteilungen über Höhlen- und Karstforschung.

[B13396231] Griepenburg Wiard (1934). Die Berghauser Höhle bei Schwelm i.W.. Mitteilungen zur Höhlen- und Karstforschung.

[B13396254] Griepenburg Wiard (1935). Kluterthöhle, Bismarck- und Rentropshöhle bei Milspe und ihre Tierwelt. Abhandlungen aus dem Westfälischen Provinzial-Museum für Naturkunde.

[B13396278] Guéorguiev V., Beron P. (1962). Essai sur la faune cavernicole de Bulgarie. Annales de Spéléologie: Revue trimestrielle.

[B13396328] Györfi J. (1948). Beiträge zur Kenntnis der Ichneumoniden Ungarns. V.. Fragmenta Faunistica Hungarica.

[B13396337] Györfi J. (1963). Beiträge zur Biologie und Ökologie der Schlupfwespen (Ichneumonidae). Zeitschrift für Angewandte Entomologie.

[B13396346] Habermehl Heinrich Friedrich (1896). Über die Lebensweise der Ichneumonen. Jahresbericht des Großherzoglichen Gymnasiums und der Großherzoglichen Realschule zu Worms.

[B13396357] Habermehl Heinrich Friedrich (1916). Beiträge zur Kenntnis der palaearktischen Ichneumonidenfauna. Zeitschrift für wissenschaftliche Insektenbiologie.

[B13396366] Habermehl Heinrich Friedrich (1929). Neue und wenig bekannte paläarktische Ichneumoniden. V. Nachtrag. Konowia.

[B13396386] Hadjuk Zdzisław Hadjuk, Orłoszek Antoni (1970). Wyniki badań faunistycznych Jaskini Niedźwiedziej = Les résultats des recherches sur la faune de la Grotte de L'ours. Acta Universitatis Wratislaviensis.

[B13577382] Hahn Daniel A., Denlinger David L. (2007). Meeting the energetic demands of insect diapause: Nutrient storage and utilization. Journal of Insect Physiology.

[B13396404] Hancock G. L.R. (1923). On some hibernating Ichneumonidae from the Cambridgeshire fens. Entomologist’s Monthly Magazine.

[B13396413] Hancock G. L.R. (1925). Notes on the hibernation of Ichneumonidae and on some parasites of *Tortrix
viridana* L.. Entomologist’s Monthly Magazine.

[B13499726] Haris Atilla, Józan Zsolt, Schmidt Péter, Glemba Gábor, Tomozii Bogdan, Csóka György, Hirka Anikó, Sima Peter, Tóth Sándor (2025). Climate Change Influences on Central European Insect Fauna over the Last 50 Years: Mediterranean Influx and Non-Native Species. Ecologies.

[B13396472] Hedwig Karl (1927). Verzeichnis der bisher in Schlesien aufgefundenen Hymenopteren. V. Ichneumonidae. Zeitschrift für Entomologie (Breslau).

[B13396481] Hedwig Karl (1943). Verzeichnis der bisher in Schlesien aufgefundenen Hymenopteren. V. Ichneumonidae: 6. Zugänge. Zeitschrift für Entomologie (Breslau).

[B13396490] Heinrich Gerd (1926). Beiträge zur Kenntnis der Ichneumonidenfauna Polens. Polskiego Pisma Entomologicznego.

[B13396499] Heinrich Gerd (1927). Beiträge zur Ichneumonidenfauna Polens: I. Nachtrag (Ichneumoninae u. Pimplinae). Polskiego Pisma Entomologicznego.

[B13581014] Heinrich G. (1928). Einige seltene, neue oder bisher in beiden Geschlechtern noch nicht bekannte Ichneumoniden (Hym.). Berliner entomologische Zeitschrift.

[B13400852] Heinrich Gerd (1930). Zur Sytematik der Ichneumoninae stenopneusticae II. Konowia.

[B13401544] Heinrich Gerd (1935). Zur Systematik der Ichneumoninae stenopneusticae VII. (Hym.). Berliner Entomologische Zeitschrift.

[B13396508] Heinrich Gerd (1936). Die von mir in Bulgarien gesammelten Ichneumoninae und Cryptinae (Insecta, Hymenoptera). Mitteilungen aus den Königlichen naturwissenschaftlichen Institute in Sofia = Известия на Царските природонаучни институти в София.

[B13396526] Heinrich Gerd (1949). (Hym. Ichneum.) Die Pterocorminae der Hahnheide (Fortsetzung). Bombus: Faunistische Mitteilungen aus Nordwestdeutschland.

[B13396517] Heinrich Gerd (1949). Ichneumoniden des Berchtesgadener Gebietes (Hym.). Mitteilungen der Münchner Entomologischen Gesellschaft.

[B13396535] Heinrich Gerd (1951). Beitrage zur Kenntnis der Ichneumoninae. Nachrichten des Naturwissenschaftlichen Museums der Stadt Aschaffenburg.

[B13396544] Hilpert Hilpert (1987). Schlupfwespen des Feldberggebietes (Hymenoptera, Ichneumonidae). Carolinea.

[B13396740] Hilpert Hubert (1987). Erster Beitrag zur Kenntnis der südbadischen Schlupfwespenfauna: Ichneumoniden des Feldberggebietes. I. Faunistik. Mitteilungen des Badischen Landesvereins für Naturkunde und Naturschutz (Freiburg).

[B13396749] Hilpert Hubert (1989). Zur Hautflügerfauna eines südbadischen Eichen-Hainbuchenmischwaldes. Spixiana.

[B13400656] Hilpert Hubert (1992). Zur Systematik der Gattung *Ichneumon* Linnaeus, 1758 in der Westpaläarktis (Hymenoptera, Ichneumonidae, Ichneumoninae). Entomofauna.

[B13396815] Hinz Rolf (1975). Beschreibung und Zucht von *Ichneumon
nebulosae* spec. nov. Hymenoptera: Ichneumonidae).. Beiträge zur Entomologie = Contributions to Entomology.

[B13396824] Hinz Rolf (1975). Vier Bemerkungen zur Systematik der Ichneumonidae (Hym.). Nachrichtenblatt der Bayerischen Entomologen.

[B13396833] Hinz Rolf (1981). Biologie und Zucht von *Ichneumon
didymus* Grav. (Hymenoptera: Ichneumonidae). Zeitschrift der Arbeitsgemeinschaft Österreichischer Entomologen.

[B13396842] Hinz Rolf (1983). The biology of the European species of the genus *Ichneumon* and related species (Hymenoptera: Ichneumonidae). Contributions of the American Entomological Institute.

[B13396861] Hinz Rolf (1991). Untersuchungen zur Lebensweise von Arten der Ichneumonini (Hymenoptera, Ichneumonidae). Mitteilungen der Schweizerischen Entomologischen Gesellschaft.

[B13396788] Hinz Rolf, Kreissl Erich (1992). Nachweise von überwinternden Schlupfwespen aus dem Grazer Bergland (Steiermark) (Hymenoptera, Ichneumonidae). Mitteilungen der Abteilung für Zoologie am Landesmuseum Joanneum Graz.

[B13396797] Hinz Rolf, Kreissl Erich (1993). Weitere Nachweise von Schlupfwespen aus der Steiermark (Hymenoptera, Ichneumonidae). Mitteilungen der Abteilung für Zoologie am Landesmuseum Joanneum Graz.

[B13396870] Hinz Rolf, Horstmann Klaus (1998). Zucht einiger *Ichneumon*-Arten aus ihren Wirten I (Hymenoptera, Ichneumonidae). Entomofauna: Zeitschrift für Entomologie.

[B13396758] Hinz Rolf, Horstmann Klaus (1999). Zur Lebensweise der europäischen Arten von *Chasmias* Ashmead, 1900 und *Limerodops* Heinrich, 1949 (Hymenoptera, Ichneumonidae, Ichneumoninae). Entomofauna: Zeitschrift für Entomologie.

[B13399816] Hinz Rolf, Horstmann Klaus (2000). Die westpaläarktischen Arten von *Exephanes* Wesmael (Insecta, Hymenoptera, Ichneumonidae, Ichneumoninae). Spixiana: Zeitschrift für Zoologie.

[B13396879] Holmgren August Emil (1864). Ichneumonologia Suecica: I. Ichneumonides oxypygi.

[B13396905] Horstmann Klaus (1970). Die Ichneumoniden (Hymenoptera) von der Nordseeküste Schleswig-Holsteins. Faunistisch-Ökologische Mitteilungen.

[B13396914] Horstmann Klaus (1980). Revision der europäischen Arten der Gattung *Aclastus* Förster (Hymenoptera, Ichneumonidae). Polskie Pismo Entomologiczne.

[B13401703] Horstmann Klaus (1993). Die europäischen Arten von *Gnotus* Förster und *Uchidella* Townes (Hymenoptera, Ichneumonidae, Cryptinae). Zeitschrift der Arbeitsgemeinschaft Österreichischer Entomologen.

[B13396941] Horstmann Klaus (2000). Die europäischen Arten von *Probolus* Wesmael, 1845 (Hymenoptera, Ichneumonidae). Entomofauna: Zeitschrift für Entomologie.

[B13442241] Horstmann Klaus (2003). Revision von Schlupfwespen-Arten VII (Hym. Ichneumonidae). Mitteilungen der Münchner Entomologischen Gesellschaft.

[B13411368] Jackson Dorothy J. (1937). Host-selection in *Pimpla
eximinator* F. (Hymenoptera). Proceedings of the Royal Entomological Society of London. Series A, General Entomology.

[B13397735] Janssens K. (1976). Enkele waarnemingen betreffende Ichneumonidae subfamilie Ichneumoninae. Phegea.

[B13396950] Jeannel René (1926). Faune cavernicole de la France.

[B13411341] Johansson Niklas, Cederberg Björn (2019). Review of the Swedish species of *Ophion* (Hymenoptera: Ichneumonidae: Ophioninae), with the description of 18 new species and an illustrated key to Swedish species. European Journal of Taxonomy.

[B13400884] Johansson Niklas (2024). Review of the Swedish species of *Lissonota* Gravenhorst (Hymenoptera: Ichneumonidae: Banchinae) with an illustrated key to the females of the Western Palaearctic.

[B13397128] Johnson W. F. (1912). *Ichneumon
lugens*, Grav., hibernating. The Irish Naturalist.

[B13397137] Johnson W. F. (1920). Hibernating ichneumon flies. The Irish Naturalist.

[B13397146] Jonaitis V. (1982). Стали известны девять новых видов паразитических наездников в Литовской ССР из подсемейства ихневмониды, обнаруженных между 1963 и 1980 годами в Ихневмонинае = 9 Ichneumonid species of the Ichneumoninae subfamily new to the Lithuanian SSR, found in 1969-1980. Miscellaneous: Novye i redkie dlya Litovskoi SSR vidy nasekomykh. Soobshcheniya i opisaniya.

[B13411350] Jussila Reijo, Sääksjärvi Ilari E., Bordera Santiago (2010). Revision of the western Palaearctic *Mesoleptus* (Hymenoptera: Ichneumonidae). Annales de la Société entomologique de France (N.S.).

[B13364715] Kocot-Zalewska Joanna, Domagała Paweł (2020). Terrestrial invertebrate fauna of Polish caves – a summary of 100 years of research. Subterranean Biology.

[B13397156] Kolarov J. A. (1992). On the hibernation and spreading of some ichneumonids (Hymenoptera, Ichneumonidae). Acta Zoologica Bulgarica.

[B13397196] Kovác L'ubomir, Hudec Igor, L'uptácik Peter, Mock Andrej, Kosel Vladimír, Fend'a Peter (2002). Spoločenstvá kavernikolných člankonožcov (Arthropoda) Demänovských jaskýň. Biospeleológia.

[B13397187] Kováč Ľubomír (2019). Ice Caves. Ecological Studies.

[B13397207] Kowalski Kazimierz (1955). The cave fauna of the Polish Tatra Mountains = Fauna Jaskin Tatr Polskich. Ochrona Przyrody.

[B13397216] Kriechbaumer Josef (1881). Ichneumoniden-Studien. Entomologische Nachrichten.

[B13397227] Kriechbaumer Josef (1888). Neue Ichneumoniden des Wiener Museums. Annalen des Naturhistorischen Museums in Wien.

[B13397236] Kriechbaumer Josef (1890). Neue Schlupfwespen aus Nord- und Mittel- Deutschland. Entomologische Nachrichten.

[B13397254] Kriechbaumer Josef (1895). Ichneumoniden-Studien. Entomologische Nachrichten.

[B13397263] Kriechbaumer Josef (1896). Neueste Studien über die Männchen des *lchn. extensorius* u. *suspiciosus*. Entomologische Nachrichten.

[B13397272] Kriechbaumer Josef (1896). Ichneumonologica varia. Entomologische Nachrichten.

[B13401035] Kriechbaumer Josef (1899). Ichneumonologica varia. Entomologische Nachrichten.

[B13397281] Kubátová Alena, Dvořák Libor (2005). Entomopathogenic fungi associated with insect hibernating in underground shelters. Czech Mycology.

[B13397290] Kulagin (1886). On the ichneumon-flies. Izvestija Imperatorskago Obscestva Ljubitelej Estestvoznanija, Antropologii i Etnografii.

[B13397308] Lana Enrico, Casale Achille, Giachino Pier Mauro (2008). Dodici anni di ricerche biospeleologiche nelle Alpi Occidentali: Risultati e prospettive. Memorie dell’Istituto italiano di Speleologia.

[B13397299] Lana Enrico, Sella Renato (2016). Le grotte del Monte Fenera e la loro fauna. Rivista Piemontese di Storia Naturale.

[B13400639] Lana E., Giachino P. M., Casale E. (2021). Fauna Hypogaea Pedemontana: Grotte e ambienti sotterraneidel Piemonte e della Valle d’Aosta.

[B13397326] Lauterborn Robert (1926). Faunistische Beobachtungen aus dem Gebiet des Oberrheins und des Bodensees. Mitteilungen des Badischen Landesvereins für Naturkunde und Naturschutz (Freiburg).

[B13397317] Lauterborn Robert (1936). Faunistische Beobachtungen aus dem Gebiete des Oberrheins und des Bodensees. Mitteilungen des Badischen Landesvereins für Naturkunde und Naturschutz (Freiburg).

[B13397335] Leclercq Jean (1942). Notes sur les Hyménoptères des environs de Liège. Bulletin du Musée royal d'Histoire Naturelle de Belgique.

[B13397344] Leclercq Jean (1953). Sur les ichneumonides (Hymenoptera) de la Belgique et des pays voisin. Bulletin du Musée royal d'Histoire naturelle de Belgique.

[B13397362] Lenart Jan, Schuchová Kristýna, Kasing Martin, Falteisek Lukás, Cimalová Sárka, Bílá Jana, Licbinská Monika, Kupka Jirí (2022). The abandoned underground mine as a semi-natural ecosystem: The story of Flaschar’s Mine (Czechia). Catena.

[B13397375] Leruth Robert (1939). La biologie du domaine souterrain et la faune cavernicole de la Belgique. Mémoires du Musée Royal d’Histoire Naturelle de Belgique.

[B13394660] Lungu-Constantineanu Camil Ştefan, Constantineanu Raoul (2014). New data on ichneumonid hibernation (Hymenoptera: Ichneumonidae) in the Bârnova forest massif (Iaşi county, Romania). Studii si Cercetari de Biologie: Seria Zoologie = Romanian Journal of Biology: Zoology.

[B13397540] Maneval Henri (1925). Capture d'Ichneumonides nouveaux pour la faune française et note biologique sur une espèce de la même famille. Bulletin de la Société Entomologique de France.

[B13397549] Meyer Heinrich (1913). Biologische Verhältnisse einheimischer Hymenopteren zur Winterzeit. Verhandlungen des Naturhistorischen Vereines der Preussischen Rheinlande.

[B13397558] Mjøs Alf Tore, Austevik Jostein, Birkeland Jarl, Engan Gunnar, Husdal Thomas, Mjølsnes Kjell, Ørsnes Geir (2023). New records of Darwin Wasps (Hymenoptera, Ichneumonidae) from Norway. Norwegian Journal of Entomology.

[B13397570] Mocsáry Sándor (1877). Bihar és Hajdu megyék hártya-, két-, reczés-, egyenes- és félröpűi. Mathematikai és Természettudományi Közlemények.

[B13397579] Mocsáry Sándor (1885). Adatok Magyarország fürkészdarázsainak ismeretéhez: Data ad cognitionem ichneumonidarum Hungariae. Mathematikai és Természettudományi Közlemények.

[B13397588] Mohr Erna (1938). Neue biologische Untersuchungen in der Segeberger Höhle. Schriften des Naturwissenschaftlichen Vereins für Schleswig-Holstein.

[B13397597] Morley Claude (1903). Ichneumonologia Brittannica: The ichneumons of Great Britain.

[B13397614] Morley Claude (1907). Ichneumonologia Brittannica: The ichneumons of Great Britain II. Cryptinae.

[B13397622] Novak Tone (2005). Terrestrial fauna from cavities in northern and central Slovenia, and a review of systematically ecologically investigated cavities. Acta Carsologica.

[B13397631] Novak T., Thirion C., Janžekovič F. (2010). Hypogean ecophase of three hymenopteran species in Central European caves. Italian Journal of Zoology.

[B13397640] Ortiz-Sánchez F. J., Fernández Toni Pérez (2008). Captura de Ichneumonidae (Insecta, Hymenoptera) en cuevas de Andalucía (España). Monografías Bioespeleológicas.

[B13397649] Ozimec Roman, Matocec Sanja Gottstein (2002). An overview of the cave and interstitial biota of Croatia.

[B13397662] Ozols E. (1941). Qualitative und quantitative Untersuchungen fiber die Ichneumonidenfauna eines Fichtenwaldes in Lettland. Folia Zoologica et Hydrobiologica.

[B13400631] Parenzan P., Belmonte G. (2002). Animalia speluncarum Italiae et omnis alii subterranei habitat terrae marisque.

[B13397671] Pax F., Maschke K. (1935). Die Höhlenfauna des Glatzer Schneeberges: 1. Die rezente Metazoenfauna. Beiträge zur Biologie des Glatzer Schneeberges.

[B13397689] Penado Andreia, Sampaio Marta, Madeira Madalena, Selfa Jesus, Rosa Gonçalo (2013). Where to spend the winter in Serra Da Estrela? The first record of *Diphyus
quadripunctorius* (Müller, 1776) overwintering in a subterranean habitat in Portugal. Entomological News.

[B13397699] Pénigot William (2020). Contribution à la connaissance des Ichneumonidae hivernants (Hymenoptera) de la forêt de Grésigne. Carnets Natures.

[B13397708] Perkins J. F. (1952). On some British Species of *Ichneumon* and *Alomyia* (Hym., Ichneumonidae). Bulletin of Entomological Research.

[B13411306] Perkins J. F. (1960). Hymenoptera: Ichneumonoidea: Ichneumonidae, subfamilies Ichneumoninae II, Alomyinae, Agriotypinae and Lycorininae.

[B13397717] Pfeffer W. (1913). Die Ichneumoniden Württembergs mit besonderer Berücksichtigung ihrer Lebensweise: I. Teil. Jahreshefte des Vereins für vaterländische Naturkunde in Württemberg.

[B13397726] Pfeifer Manfred Alban (2016). Phänologie von *Neotypus
melanocephalus* (Gmelin, 1790) (Hymenoptera: Ichneumonidae), eines Parasitoiden der Wiesenknopf-Ameisenbläulinge *Phengaris
nausithous* (Bergsträsser, 1779) et *Phengaris
teleius* (Bergsträsser, 1779) (Lepidoptera: Lycaenidae). Entomologische Zeitschrift.

[B13400696] Pic Maurice (1916). Notes de chasse. L'Échange: Revue Linnéenne.

[B13397744] Pic Maurice (1917). Hivernage des Ichneumoniens. L'Échange: Revue Linnéenne.

[B13397753] Pic Maurice (1919). Une chasse aux Ichneumonides. L'Échange: Revue Linnéenne.

[B13756786] Team Posit (2025). RStudio: Integrated development environment for R.

[B13397762] Pottler Benjamin, Coles L. W. (1962). Biology of *Biolysia
tristis* (Hymenoptera, Ichneumonidae) and its role as a parasite of the Clover Leaf Weevil (*Hypera
punctata*). Journal of Economic Entomology.

[B13397771] Presetnik Primož, Mulaomerovic Jasminko, Pašic Jasmin, Napotnik Ivan, Milanolo Simone, Budinski Ivana, Pejic Branka (2016). Rezultati pregleda potencijalnih zimskih skloništa šišmiša u Bosni i Hercegovini u zimu 2015/16 = Survey results of potential bat hibernacula in Bosnia and Herzegovina in winter 2015/16. Hypsugo.

[B13577409] Quicke Donald (2015). The Braconid and Ichneumonid Parasitoid Wasps: Biology, Systematics, Evolution and Ecology.

[B13397783] Ranin Olli (1985). Drei neue Arten der Gattung *Tycherus* (Hymenoptera, lchneumoninae: Phaeogenini). Notulae Entomologicae.

[B13397792] Rasnitsyn A. P. (1959). Hibernation sites of ichneumon flies (Hymenoptera, Ichneumonidae). Entomological Review.

[B13397801] Rasnitsyn A. P. (1964). Overwintering of ichneumon flies (Hymenoptera, Ichneumonidae). Entomogical Review.

[B13400991] Rasnitsyn A. P., Siitan U. V., Medvedev G. S. (1981). A guide to the insects of the European part of the USSR Hymenoptera, Ichneumonidae Vol. III..

[B13397810] Rathgeber Thomas (2004). Die Bärenhöhle bei Rohnbach (Kat.-Nr. 7316/1, Gemeinde Enzklösterle, Landkreis Calw), eine wenig bekannte Sandsteinhöhle im Nordschwarzwald. Beiträge zur Höhlen- und Karstkunde in Südwestdeutschland.

[B13756777] Team R Core (2024). R: A language and environment for statistical computing.

[B13397819] Renken W. (1956). Untersuchungen über Winterlager der Insekten. Zeitschrift für Morphologie und Ökologie der Tiere.

[B13397836] Riedel Matthias, Humala Andrei (2009). Faunistic notes on the Ichneumoninae (Hymenoptera: Ichneumonidae, excl. Phaeogenini) from the European part of Russia. Russian Entomological Journal.

[B13401685] Riedel Matthias (2012). Revision der westpaläarktischen Arten der Gattung *Coelichneumon* Thomson (Hymenoptera: Ichneumonidae: Ichneumoninae). Linzer Biologische Beiträge.

[B13397863] Riedel Matthias (2018). Contribution to the Siberian species of *Ichneumon* Linnaeus (Hymenoptera, Ichneumonidae, Ichneumoninae). Linzer biologische Beiträge.

[B13399736] Riedel Matthias (2021). Revision of the Palaearctic species of *Syspasis* Townes, 1965 (Hymenoptera, Ichneumonidae, Ichneumoninae). Linzer Biologische Beiträge.

[B13411262] Riedel Matthias (2021). The Western Palaearctic species of the subtribe Hoplismenina Heinrich (Hymenoptera, Ichneumonidae, Ichneumoninae). Linzer biologische Beiträge.

[B13397872] Rossius Petrus (1790). Fauna Etrusca: Tomus primus.

[B13402871] Santos Bernardo, Wahl David, Rousse Pascal, Bennett Andrew, Kula Robert, Brady Séan (2021). Phylogenomics of Ichneumoninae (Hymenoptera, Ichneumonidae) reveals pervasive morphological convergence and the shortcomings of previous classifications. Systematic Entomology.

[B13580201] Schebeck Martin, Lehmann Philipp, Laparie Mathieu, Bentz Barbara J., Ragland Gregory J., Battisti Andrea, Hahn Daniel A. (2024). Seasonality of forest insects: why diapause matters. Trends in Ecology & Evolution.

[B13397889] Schmidt Konrad, Zmudzinski Franz (2005). Beiträge zur Kenntnis der badischen Schlupfwespenfauna (Hymenoptera, Ichneumonidae): 5. Unterfamilie Ichneumoninae. Carolinea.

[B13397919] Schmitz H. (1909). Die Insektenfauna der. Höhlen von Maastricht und Umgegend. Tijdschrift voor Entomologie.

[B13397965] Schwarz Martin (1994). Beitrag zur Systematik und Taxonomie europäischer *Gelis*-Arten mit macropteren oder brachypteren Weibchen (Hymenoptera, Ichneumonidae). Linzer biologische Beiträge.

[B13397974] Schwarz Martin (1995). Revision der westpaläarktischen Arten der Gattung *Gelis* Thunberg mit apteren Weibchen und *Thaumatogelis* Schmiedeknecht (Hymenoptera, Ichneumonidae): Teil 1. Linzer Biologische Beiträge.

[B13397983] Schwarz Martin (1998). Revision der westpaläarktischen Arten der Gattungen *Gelis* Thunberg mit apteren Weibchen und *Thaumatogelis* Schmiedeknecht (Hymenoptera, Ichneumonidae): Teil 2. Linzer biologische Beiträge.

[B13400909] Schwarz Martin, Shaw Mark (1998). Western Palaearctic Cryptinae (Hymenoptera: Ichneumonidae) in the National Museums of Scotland, with nomenclatural changes, taxonomic notes, rearing records and special reference to the British check list: Part 1. Tribe Cryptini. Entomologist's Gazette.

[B13397928] Schwarz Martin, Shaw Mark (1999). Western Palaearctic Cyptinae (Hymenoptera: Ichneumonidae) in the National Museum of Scotland, with nomenclatural changes, taxonomic notes, rearing records and special reference to the British check list: Part 2. Genus *Gelis* Thunberg (Phygadeuontini: Gelina). Entomologist's Gazette.

[B13397946] Schwarz Martin, Shaw Mark (2000). Western Palaearctic Cryptinae (Hymenoptera: Ichneumonidae) in the National Museums of Scotland, with nomenclatural changes, taxonomic notes, rearing records and special reference to the British check list: Part 3. Tribe Phygadeuontini, subtribes Chiroticina, Acrolytina, Hemitelina and Gelina (excluding *Gelis*), with descriptions of new species. Entomologist's Gazette.

[B13397992] Schwarz Martin (2001). Revision der westpaläarktischen Arten der Gattungen *Gelis* Thunberg mit apteren Weibchen und *Thaumatogelis* Schwarz (Hymenoptera, Ichneumonidae): Teil 4. Linzer biologische Beiträge.

[B13398009] Schwarz Martin (2002). Revision der westpaläarktischen Arten der Gattungen *Gelis* Thunberg mit apteren Weibchen und *Thaumatogelis* Schwarz (Hymenoptera, Ichneumonidae): Teil 3. Linzer Biologische Beiträge.

[B13397956] Schwarz Martin, Shaw Mark (2010). Western Palaearctic Cryptinae (Hymenoptera: Ichneumonidae) in the National Museums of Scotland, with nomenclatural changes, taxonomic notes, rearing records and special reference to the British check list: Part 4. Tribe Phygadeuontini, subtribes Mastrina, Ethelurgina, Endaseina (excluding *Endasys*), Bathythrichina and Cremnodina. Entomologist's Gazette.

[B13401714] Schwarz Martin, Shaw Mark (2011). Western Palaearctic Cryptinae (Hymenoptera: Ichneumonidae) in the National Museums of Scotland, with nomenclatural changes, taxonomic notes, rearing records and special reference to the British check list: Part 5. Tribe Phygadeuontini, subtribe Phygadeuontina, with descriptions of new species. Entomologist's Gazette.

[B13398036] Sebald Helmut, Bauer Rudolf, Schönitzer Klaus, Diller Erich (2000). Ichneumonidae, die als Imagines überwintern (Insecta, Hymenoptera, Ichneumonidae). Entomofauna: Zeitschrift für Entomologie.

[B13398063] Sebald Helmut, Schönitzer Klaus, Diller Erich (2001). Überwinternde Ichneumoniden in Bayern (Hymenoptera, Ichneumonidae). Nachrichtenblatt der Bayerischen Entomologen.

[B13398046] Sebald Helmut (2007). Auswirkungen des milden Winters 2006/2007 auf die Diapause bei Ichneumonidae der Gattung *Ichneumon* (Insecta, Hymenoptera: Ichneumonidae). Nachrichtenblatt der Bayerischen Entomologen.

[B13398018] Sebald Helmut, Weber Dieter (2013). Schlupfwespen (Insecta, Hymenoptera, Ichneumonidae) aus Höhlen des Großherzogtums Luxemburg. Ferrantia.

[B13398081] Šedivý Josef (2001). Contribution to the taxonomy and knowledge of hosts of ichneumonids (Hymenoptera: Ichneumonidae). Klapalekiana.

[B13398255] Šedivý Josef, Dvořák Libor (2002). Přezimování lumků ve štolách, jeskyních a sklepech (Ichneumonidae, Hymenoptera) = Hibernation of Ichneumonids in mine galleries, caves and cellars (Ichneumonidae, Hymenoptera). Sborn. Přírodověd. Klubu v Uher. Hradišti.

[B13398273] Selfa Jésus, Escolà Oleguer (1991). Primeros datos sobre los Ichneumoninae (Hymenopetra, Ichneumonidae) encontrados en cuevas de la Península Ibérica = First data about the cave Ichneumoninae (Hymenoptera, Ichneumonidae) from the Iberian peninsula. Miscellània Zoològica.

[B13398264] Selfa Jésus, Bordera Santiago (1995). Ichneumonini and Protichneumonini (Hymenoptera, Ichneumonidae, Ichneumoninae) of the Museu de Zoologia of Barcelona. Miscellània Zoològica.

[B13398282] Seyrig André (1923). Observations sur la biologie des ichneumons. Annales de la Société Entomologique de France.

[B13398300] Seyrig André (1926). Ichneumonides récoltés par M. R. Benoist à Vendresse (Ardennes), le 26 décembre 1925. Bulletin de la Société Entomologique de France.

[B13398291] Seyrig André (1926). Observations sur les ichneumonides [1^re^ série]. Annales de la Société Entomologique de France.

[B13401597] Shaw Mark (2006). Notes on British Pimplinae and Poemeniinae (Hymenoptera: Ichneumonidae), with additions to the British list. British Journal of Entomology and Natural History.

[B13398309] Shaw Mark (2009). Notes on the host-feeding and hyperparasitic behaviours of *Itoplectis* species (Hymenoptera: Ichneumonidae, Pimplinae). Entomologist’s Gazette.

[B13411359] Shaw Mark, Jennings Malcolm, Quicke Donald (2011). The identity of *Scambus
planatus* (Hartig, 1838) and *Scambus
ventricosus* (Tschek, 1871) as seasonal forms of *Scambus
calobatus* (Gravenhorst, 1829) in Europe (Hymenoptera, Ichneumonidae, Pimplinae, Ephialtini). Journal of Hymenoptera Research.

[B13398747] Shaw Mark R., Horstmann Klaus, Whiffin Ashleigh (2016). Two hundred and twenty-five species of reared western Palaearctic Campopleginae (Hymenoptera: Ichneumonidae) in the National Museums of Scotland, with descriptions of new species of *Campoplex* and *Diadegma*, and records of fifty-five species new to Britain. Entomologist’s Gazette.

[B13398685] Shaw Mark R. (2017). Anatomy, reach and classification of the parasitoid complex of a common British moth, *Anthophila
fabriciana* (L.) (Choreutidae). Journal of Natural History.

[B13580215] Shen Zhen, Tang Liang-De, Desneux Nicolas, Zang Lian-Sheng (2025). Diapause in parasitoids: a systematic review. Journal of Pest Science.

[B13398318] Shuckard W. E. (1838). Notes on varous insects; with further explanatory observations. The Entomological Magazine.

[B13398327] Skalski A. W. (1969). Materiały do znajomości fauny jaskiń tatrzańskich. Speleologia.

[B13398336] Skalski A. W. (1973). Materiały do znajomości bezkręgowców jaskiń Wyżyny Krakowsko-Częstochowskiej. Rocznik Muzeum Częstochowskiego.

[B13398358] Skalski A. W. (1981). Charakterystyka fauny podziemnej Wyżyny Krakowsko-Częstochowskiej. Rocznik Muzeum Okręgowego w Częstochowie.

[B13457536] Smits van Burgst C. A.L. (1918). Naamlijst der in de Ichneumonen-collectie van het rijk aanwezige genera en specie der familie Ichneumonidae.

[B13398367] Starke Hermann (1956). Ichneumonidenfauna der sächsischen Oberlausitz. Natura Lusatica.

[B13400955] Storey Malcolm William, Ørsnes Geir, Pénigot William, Verheyde Fons, Broad Gavin (2025). Three Darwin wasps rediscovered or new to Britain from Portland, Dorset. British Journal of Entomology & Natural History.

[B13398376] Strinati Pierre (1966). Faune cavernicole de la Suisse. Annales de Spéléologie.

[B13398385] Strouhal Hans, Vornatscher Josef (1975). Katalog der rezenten Höhlentiere Österreichs. Annalen des Naturhistorischen Museums in Wien.

[B13398394] Szilády Zoltán (1914). Magyarországi rovargyjtésem jegyzéke: III. Hymenoptera. Rovartani Lapok.

[B13454813] Telenga N. A. (1929). Hymenopterous parasites of the family Ichneumonidae reared at Kuban Plant Protection Station in 1927. Newsletter of the Courses of Applied Zoology and Phytopathology.

[B13398412] Tereshkin A. M. (2002). Faunistic review of the genus *Ichneumon* Linnaeus, 1758 in Byelorussia. Entomofauna: Zeitschrift für Entomologie.

[B13398403] Tereshkin A. M. (2003). Notes on the ichneumon flies of the genera *Rictichneumon* Heinrich, 1961, *Rhadinodonta* Szepligeti, 1908, *Eristicus* Wesmael, 1844 and *Auritus* Constantineanu, 1969 (Hymenoptera, Ichneumonidae, Ichneumoninae). Euroasian Entomological Journal = Евразиатский энтомол. журнал.

[B13398421] Tereshkin A. M. (2004). Review of ichneumon flies of genus *Aoplus* (Hymenoptera, Ichneumonidae, Ichneumoninae) in Belarus. Proceedings of the National Academy of Sciences of Belarus: Biological Series.

[B13398430] Tereshkin A. M. (2005). Species of *Homotherus* and *Stenaoplus* genera (Hymenoptera, Ichneumonidae, Ichneumoninae) in Belarus. Proceedings of the National Academy of Sciences of Belarus: Biological Series.

[B13398439] Tereshkin A. M. (2011). Illustrated key to the genera of the subtribe Amblytelina of Palaearctic (Hymenoptera, Ichneumonidae, Ichneumoninae, Ichneumonini). Linzer biologische Beiträge.

[B13398703] Tereshkin A. M. (2014). Datasets on several subtribes of Ichneumoninae. http://tereshkin.info/Articles.htm.

[B13401615] Timuș Natalia, Constantineanu Raoul, Rákosy László (2014). *Ichneumon
balteatus* (Hymenoptera: Ichneumonidae) – a new parasitoid speciesof *Maculinea
alcon* butterflies (Lepidoptera: Lycaenidae). Entomologica Romanica.

[B13398448] Tischbein P. F.L. (1863). Befruchtete ichneumonen-weibchen im Winterquartier. Jahrbuch der Königl.-Sächs. Akademie für Forst- und Landwirthe zu Tharand.

[B13398458] Tischbein P. F. (1871). Hymenopterologische Beiträge. Entomologische Zeitung (Stettin).

[B13398467] Tischbein P. F. (1873). Uebersicht der europäischen Arten des genus *Ichneumon* (Wesmael) mit Angabe der bei Birkenfeld vorkommenden und Beschreibung neuer Arten. Entomologische Zeitung (Stettin).

[B13398476] Tischbein P. F. (1873). Uebersicht der europäischen Arten des genus *Ichneumon* (Wesmael) mit Angabe der bei Birkenfeld vorkommenden und Beschreibung neuer Arten: Fortsetzung. Entomologische Zeitung (Settin).

[B13398485] Tischbein P. F. (1874). Uebersicht der europäischen Arten des Genus *Ichneumon* (Wesmael) mit Angabe der bei Birkenfeld vorkommenden und Beschreibung neuer Arten: Fortsetzung. Entomologische Zeitung (Stettin).

[B13398503] Tischbein P. F. (1874). Uebersicht der europäischen Arten des Genus *Ichneumon* (Wesmael) mit Angabe der bei Birkenfeld vorkommenden und Beschreibung neuer Arten: Fortsetzung. Entomologische Zeitung (Stettin).

[B13398512] Tischbein P. F. (1876). Zusätze und Bemerkungen zu der Uebersicht der europäischen Arten des Genus *Ichneumon*. Entomologische Zeitung (Stettin).

[B13398521] Tischbein P. F. (1879). Zusätze und Bemerkungen zu der Uebersicht der europäischen Arten des Genus *Ichneumon*. Entomologische Zeitung (Stettin).

[B13398530] Tischbein P. F. (1881). Zusätze und Bemerkungen zu der Übersicht der europäischen Arten des Genus *Ichneumon* Gr.. Entomologische Zeitung (Stettin).

[B13398539] Torka Valentin (1915). Ichneumoniden der Provinz Posen. Deutsche Entomologische Zeitschrift (Berlin).

[B13577391] Tougeron Kévin (2019). Diapause research in insects: historical review and recent work perspectives. Entomologia Experimentalis et Applicata.

[B13401624] Tougeron Kévin, van Baaren Joan, Le Lann Cécile, Brodeur Jacques (2019). Diapause expression in a Québec, Canada population of the parasitoid *Aphidius
ervi* (Hymenoptera: Braconidae). The Canadian Entomologist.

[B13398548] Tsankov Georgi, Mirchev Plamen (1999). New Ichneumonidae (Hymenoptera) species for the Bulgarian fauna. Acta Entomologica Bulgaria.

[B13398557] Tschopp Andreas, Kropf Christian, Nentwig Wolfgang, Klopfstein Seraina (2012). Choosy polyphagous parasitoids: On the host preferences of *Ichneumon
albiger* and *Ichneumon
extensorius* (Hymenoptera: Ichneumonidae). Mitteilungen der Schweizerischen Entomologischen Gesellschaft.

[B13398566] Uffeln Karl (1931). Schlupfwespen. Entomologische Zeitschrift.

[B13398575] Ulrich Werner (2001). Differences in temporal variability and extinction probabilities between species of guilds of parasitic Hymenoptera. Polish Journal of Entomology.

[B13366260] Valemberg J;, Vago J. L. (1974). Nomenclature des Ichneumoninae hibernant du Cambresis. Bulletin de la Société Entomologique du Nord de la France.

[B13366269] Valemberg J., Vago J. L. (1974). Nomenclature des Ichneumoninae hibernant du Cambresis (2). Bulletin de la Société Entomologique du Nord de la France.

[B13366287] Valemberg J. (1975). Chasse aux ichneumons hibernants (suite n°4). Bulletin de la Société Entomologique du Nord de la France.

[B13366278] Valemberg J., Vago J. L. (1975). Chasse aux Ichneumoninae hibernants (suite III). Bulletin de la Société Entomologique du Nord de la France.

[B13366296] Valemberg J. (1976). Ichneumoninae hibernants (suite n°5) 120 capt.. Bulletin de la Société entomologique du Nord de la France.

[B13366305] Valemberg J. (1976). Ichneumoninae hibernants (suite n°6) 182 capt.. Bulletin de la Société entomologique du Nord de la France.

[B13366314] Valemberg J. (1978). Ichneumoninae hibernants (suite). Bulletin de la Société Entomologique du Nord de la France.

[B13366332] Valemberg J., Bosquit J. C. (1979). Ichneumoninae hibernants. Bulletin de la Société Entomologique du Nord de la France.

[B13366323] Valemberg J. (1982). Ichneumoninae hibernants. Bulletin de la Société Entomologique du Nord de la France.

[B13398584] Valemberg Joël, Fiala Miroslav (2009). Faunistic records from the Czech Republic - 296. Hymenoptera: Ichneumonidae: Ichneumoninae. Klapalekiana.

[B13366341] Valemberg J., Vago J. L. (2013). Ichneumoninae hibernant du Laonnois (Hymenoptera : Ichneumonoidea, Ichneumonidae). Bulletin de la Société Entomologique du Nord de la France.

[B13401606] Valemberg Joël (2014). Les *Heterischnus* Palearctiques (Hymenoptera: Ichneumonidae: Ichneumoninae: Phaeogenini: Heterischina): Clefs dichotomiques. Bulletin de la Societe Entomologique du Nord de la France.

[B13364724] Van Dinther J. B. M. (1951). Enkele observaties van *Barichneumon
peregrinator* L.. Entomologische Berichten.

[B13364202] Varga Oleksandr, Riedel Matthias, Diller Erich (2020). A pilot study of the Carpathian ichneumonine parasitoids (Hymenopera, Ichneumonidae: Ichneumoninae) reveals eighty-eight new records for Ukraine. Zootaxa.

[B13364169] Vas Zoltán, Kutasi Csaba (2016). Hymenoptera from caves of Bakony Mountains, Hungary – an overlooked taxon in hypogean research. Subterranean Biology.

[B13401444] Vergoossen Willem, Hageman John, Verheyde Fons (In press). Gewone sluipwespen (Hymenoptera, Ichneumonidae) in de onderaardse kalksteengroeven in Zuid-Limburg. Natuurhistorisch Maandblad.

[B13400978] Verheyde Fons, Hoekstra Paul, Libert Pierre-Nicolas, Meijer Hilco, De Ketelaere Augustijn, Vandaudenard Thibaud, Belgers Dick, Brosens Edwin (2021). Two hundred and five ichneumonid wasps reported for the first time in Belgium and the Netherlands (Hymenoptera: Ichneumonidae). Belgian Journal of Entomology.

[B13364151] Verheyde Fons, Quicke Donald L. J. (2022). Review of adult diapause in ichneumonid wasps (Hymenoptera, Ichneumonidae). Journal of Hymenoptera Research.

[B13399727] Verheyde Fons (2023). *Diphyus
salicatorius* (Gravenhorst, 1820), specialist van de Kempen in Vlaanderen. HymenoVaria.

[B13470384] Verheyde Fons, Lescroart Celie, Bracke Koenraad (2025). Twee nieuwe *Ichneumon*-soorten in België en Nederland, overwinterend aangetroffen (Hymenoptera: Ichneumonidae). Entomologische Berichten.

[B13559097] Verheyde Fons, De Ketelaere Augustijn, Ørsnes Geir, Pénigot William, Storey Malcolm, Österblad Ika, Cameron Al, Engan Gunnar, Fiala Miroslav, Lutz Jonas, Parkhomenko Maxim, Gokhman Vladimir E., Dekoninck Wouter, Cooleman Stijn (2025). Hibernating Ichneumonidae records in the Western Palearctic. Belgian Biodiversity Platform.

[B13364178] Vesović Nikola, Deltshev Christo, Mitov Plamen, Antić Dragan, Stojanović Dalibor Z., Stojanović Dejan V., Stojanović Katarina, Božanić Milenka, Ignjatović-Ćupina Aleksandra, Ćurčić Srećko (2024). The Diversity of Subterranean Terrestrial Arthropods in Resava Cave (Eastern Serbia). Diversity.

[B13398593] Weber Dieter, Weber Hans (1986). Höhlen, Felsdächer und künstliche Hohlräume im Gebiet des Kartenblattes Neustadt a. d. Weinstraße (TK 25 Bl. 6614 Neustadt a. d. Weinstr.). Mitteilungen der Pollichia.

[B13398602] Weber Dieter (1989). Die Höhlenfauna und -flora des Höhlenkatastergebietes Rheinland-Pfalz/Saarland, 2. Teil. Abhandlungen zur Karst-und Höhlenkunde.

[B13398620] Weber Dieter (2014). Die evertebrate Fauna des Varus-Tunnels bei Tholey. Abhandlungen der Delattinia.

[B13398637] Wesmael Constantin (1844). Tentamen dispositionis methodicae ichneumonum Belgii. Mémoires de l'Académie royale de Belgique.

[B13756794] Wickham Hadley (2016). ggplot2.

[B13398695] Yu D. S.K., van Achterberg C., Horstmann K. (2016). Taxapad 2016, Ichneumonoidea 2015.

[B13400775] Zetterstedt Johan Wilhelm (1838). Insecta Lapponica.

[B13398646] Zijp Jan-Piet, Blommers Leo H. M. (2002). Survival mode between the yearly reproduction periods, and reproductive biology of *Scambus
pomorum* (Hymenoptera: Ichneumonidae: Pimplinae), a parasitoid of the Apple Blossom Weevil *Anthonomus
pomorum* (Coleoptera: Curculionidae). Entomologia Generalis.

[B13400647] Žikić Vladimir, Ćurčić Srećko, Vesović Nikola (2020). *Diphyus
quadripunctorius* (Müller, 1776) (Hymenoptera: Ichneumonidae: Ichneumoninae): The first records from Serbian caves. Acta Entomologica Serbica.

[B13577517] Zinnert K. D. (1969). Vergleichende Untersuchungen zur Morphologie und Biologie der Larvenparasiten (Hymenoptera: Ichneumonidae und Braconidae) mitteleuropäischer Blattwespen aus der Subfamilie Nematinae (Hymenoptera: Tenthredinidae). Zeitschrift für Angewandte Entomologie.

[B13398673] Zwolfer H. (1962). Observations on the parasites of *Hydroecia
micacea* Esp. and *H.
petasitis* Dbl. Technical Bulletin of the Commonwealth Institute of Biological Control.

